# FMNL2 regulates gliovascular interactions and is associated with vascular risk factors and cerebrovascular pathology in Alzheimer’s disease

**DOI:** 10.1007/s00401-022-02431-6

**Published:** 2022-05-24

**Authors:** Annie J. Lee, Neha S. Raghavan, Prabesh Bhattarai, Tohid Siddiqui, Sanjeev Sariya, Dolly Reyes-Dumeyer, Xena E. Flowers, Sarah A. L. Cardoso, Philip L. De Jager, David A. Bennett, Julie A. Schneider, Vilas Menon, Yanling Wang, Rafael A. Lantigua, Martin Medrano, Diones Rivera, Ivonne Z. Jiménez-Velázquez, Walter A. Kukull, Adam M. Brickman, Jennifer J. Manly, Giuseppe Tosto, Caghan Kizil, Badri N. Vardarajan, Richard Mayeux

**Affiliations:** 1 Taub Institute for Research on Alzheimer’s Disease and the Aging Brain, College of Physicians and Surgeons, Columbia University, 630 West 168th Street, New York, NY 10032 USA; 2 The Gertrude H. Sergievsky Center, College of Physicians and Surgeons, Columbia University, 630 West 168th Street, New York, NY 10032 USA; 3 Department of Neurology, College of Physicians and Surgeons, Columbia University and the New York Presbyterian Hospital, 710 West 168th Street, New York, NY 10032 USA; 4 German Center for Neurodegenerative Diseases (DZNE), Helmholtz Association, Tatzberg 41, 01307 Dresden, Germany; 5 Rush Alzheimer’s Disease Center, Rush University Medical Center, Chicago, IL 60612 USA; 6 Department of Medicine, College of Physicians and Surgeons, Columbia University, and the New York Presbyterian Hospital, 630 West 168th Street, New York, NY 10032 USA; 7 Department of Psychiatry, College of Physicians and Surgeons, Columbia University, 1051 Riverside Drive, New York, NY 10032 USA; 8 Department of Epidemiology, School of Public Health, University of Washington, Seattle, WA 98195 USA; 9 Department of Medicine, Medical Sciences Campus, University of Puerto Rico School of Medicine, San Juan, Puerto Rico, 00936 USA; 10 School of Medicine, Pontificia Universidad Catolica Madre y Maestra (PUCMM), Santiago, Dominican Republic; 11 Department of Neurology, CEDIMAT, Plaza de la Salud, Santo Domingo, Dominican Republic; 12 School of Medicine, Universidad Pedro Henriquez Urena (UNPHU), Santo Domingo, Dominican Republic

**Keywords:** Alzheimer’s disease, Cerebrovascular risk factors, GWAS, FMNL2, Zebrafish, Human, Mouse, Gliovascular interaction, Neurovascular unit, Blood–brain-barrier

## Abstract

Alzheimer’s disease (AD) has been associated with cardiovascular and cerebrovascular risk factors (CVRFs) during middle age and later and is frequently accompanied by cerebrovascular pathology at death. An interaction between CVRFs and genetic variants might explain the pathogenesis. Genome-wide, gene by CVRF interaction analyses for AD, in 6568 patients and 8101 controls identified *FMNL2* (*p* = 6.6 × 10^–7^). A significant increase in *FMNL2* expression was observed in the brains of patients with brain infarcts and AD pathology and was associated with amyloid and phosphorylated tau deposition. FMNL2 was also prominent in astroglia in AD among those with cerebrovascular pathology. Amyloid toxicity in zebrafish increased *fmnl2a* expression in astroglia with detachment of astroglial end feet from blood vessels. Knockdown of *fmnl2a* prevented gliovascular remodeling, reduced microglial activity and enhanced amyloidosis. APP/PS1dE9 AD mice also displayed increased *Fmnl2* expression and reduced the gliovascular contacts independent of the gliotic response. Based on this work, we propose that FMNL2 regulates pathology-dependent plasticity of the blood–brain-barrier by controlling gliovascular interactions and stimulating the clearance of extracellular aggregates. Therefore, in AD cerebrovascular risk factors promote cerebrovascular pathology which in turn, interacts with *FMNL2* altering the normal astroglial-vascular mechanisms underlying the clearance of amyloid and tau increasing their deposition in brain.

**Supplementary Information:**

The online version contains supplementary material available at 10.1007/s00401-022-02431-6.

## Introduction

Alzheimer’s disease (AD) affects more than 6.2 million people in the United States and approximately 24 million worldwide. Neuropathological studies indicate that in AD, the hallmark findings of neuritic plaques and neurofibrillary tangles can frequently be accompanied by varying degrees of cerebrovascular disease in up to 70% of patients [4, 16, 44, 74]. Amyloid β in blood vessels, as in cerebral amyloid angiopathy, reduces cerebral blood flow and is present in most patients with AD. However, as cardio and cerebrovascular risk factors (CVRFs) increase in the elderly, accompanied by inflammation, cytokine release, endothelial dysfunction and arterial stiffening in brain [81]. Cholesterol laden macrophages accumulate in vessel walls also decreasing blood flow. The increase in atherosclerosis in the intracerebral arteries [55] and capillaries leads to microinfarcts in the hippocampus contributing to cognitive decline [34].

The relationship between CVRFs such as hypertension [78], body mass index [33, 57], diabetes [22, 59] and coronary heart disease [42, 65] and AD is well known, but there has been limited mechanistic evidence directly linking these vascular risk factors in AD to the presence of ischemic microvascular pathology. Each of these vascular factors has the capacity to impair the blood–brain barrier and glio-vascular units. Arterial pulses and flow are required for glymphatic clearance of molecules including amyloid β [42, 65, 86]. Jagust and colleagues argued that cerebrovascular disease contributed to AD by perturbing the amyloid β pathway in addition to causing neurodegeneration [38]. However, the effects reported in epidemiological studies of the association between CVRFs and AD are inconsistent, increasing AD risk in some studies [86], but in others showing protection against AD [64, 86]. The relationship between vascular risk factors and cerebrovascular pathology in AD could simply be the result of aging, the stage of the disease or a coincidental occurrence. An alternative explanation is that an unidentified interaction between genetic variants and vascular risk factors leading to cerebrovascular pathology in AD brain may contribute to disease pathogenesis.

*APOE*, *CLU*, *ABCA7*, and *SORL1*, typically associated with immune mechanisms in AD, are also part of the lipid metabolism pathway providing evidence for a putative molecular relationship. In fact, *ABCA7* and *SORL1* have been associated with brain infarcts [31]. Using summary statistics from multiple genome-wide associations studies (GWAS), a recent study detected evidence of pleiotropy between vascular risk factors and AD [17]. Another investigation [88] found shared genetic contribution to AD and small vessel disease. Despite the possible associations between genes, vascular factors and AD, there has been no unbiased genome-wide study of genes or genetic loci to investigate the interaction between cerebrovascular risk factors, cerebrovascular pathology, and AD.

Previously, we observed that the cumulative burden of vascular risk factors increased the association with AD risk [58, 70] compared with a single risk factor. Therefore, in this investigation, a dimensionality reduction of four common cardiovascular risk factors frequently associated with cerebrovascular disease and with AD: hypertension, heart disease, diabetes, and body mass index (BMI), was employed to create a vascular risk factor burden score. To augment the sample size, we included data from five different cohorts representing different ethnic groups, facilitating the interaction analyses.

## Materials and methods

### Clinical studies

#### Participants

Participants were from the following studies: Washington Heights–Inwood Columbia Aging Project (WHICAP), Estudio Familiar de Influencia Genetica en Alzheimer (EFIGA), the National Alzheimer’s Coordinating Center (NACC), and the Religious Orders Study and Rush Memory and Aging Project (ROSMAP) (Table 1). WHICAP is a multiethnic, prospective, community-based cohort study of aging and dementia in Medicare recipients 65 years and older residing in northern Manhattan. A detailed description of the study was previously published [83]. Participants are initially recruited as nondemented elderly and were non-Hispanic white, African American or Caribbean Hispanic. A consensus diagnosis was derived for each participant by experienced clinicians based on NINCDS‐ADRDA criteria for possible, probable, or definite AD (moderate or high likelihood of neuropathological criteria for AD) [61]. Recruitment for the EFIGA began in 1998, to study the genetic architecture of AD in the Caribbean Hispanic population. Patients with familial AD were recruited and if a sibling of the proband had dementia, all other living siblings and available relatives underwent evaluation. Cases were defined as any individual meeting NINCDS-ADRDA criteria for probable or possible AD. Details of the study have previously been reported [90]. NACC collects, organizes, and maintains phenotype information from National Institute on Aging (NIA) Alzheimer’s Disease Centers (ADCs). Genetic data were provided from wave 1–10 of the ADC genotyping. The Uniform Data Set (UDS) is made up of standardized clinical evaluations and diagnoses along with demographic information. Details on the UDS and NACC database can be found at http://www.alz.washington.edu/. ROSMAP are two harmonized longitudinal studies enrolling older adults without dementia: The Religious Orders Study and the Memory and Aging Project (ROSMAP). Details of the ROSMAP studies have been described elsewhere [9, 27]. Only participants 65 years of age or older, that had one or more in person clinical assessments and data concerning four vascular risk factors: any heart disease, hypertension, BMI, and diabetes were included.

**Table 1:** Participant demographics included

	ROSMAP	WHICAP African-Americans	WHICAP Whites	Caribbean Hispanics	NACC
*N*	1424	978	851	3404	8012
Ethnicity/race	Non-Hispanic whites	African–Americans	Non-Hispanic whites	Caribbean Hispanics	Non-Hispanic whites
% Women	70%	72%	60%	68%	56%
% AD	34%	33%	21%	51%	48%
Mean BMI (s.d.)	26.5 (5.51)	28.21 (6.17)	26.59 (5.07)	26.89 (5.26)	26.24 (4.69)
% hypertension	62%	82%	66%	76%	62%
% Heart disease	22%	39%	44%	23%	40%
% diabetes	21%	29%	15%	31%	12%
Mean age (s.d.)	85.93 (6.87)	80 (7.24)	81.01 (7.33)	75	78.42 (7.69)

*s.d.* standard deviation, *ROSMAP* Religious Orders Study and Rush Memory and Aging Project, *NACC* National Alzheimer’s Disease Coordinating Center, *WHICAP* Washington Heights, Hamilton Heights, Inwood Columbia Aging Project

### Cerebrovascular Risk Factors Score

Self-reported data from each participant were recorded as a binary indicator (Yes-Ever Had or No-Never Had) for heart disease, hypertension, and diabetes. The self-reported of vascular risk factors has reasonable reliability and validity [45]. Details on heart conditions varied between groups, so report of any heart disease qualified as presence of heart disease in this study. A quantitative variable was recorded for body mass index (BMI) from the last visit. The PCAMix package [20] computes principal components (PCs) in a mixture of quantitative and qualitative data, and was used to summarize the cardiovascular risk factors into one summary score by computing PCs from the four cerebrovascular variables. The goal of the dimensionality reduction was to capture the greatest amount of variance accounted for by the four cerebrovascular risk factors, and thus each participant’s values from the first principal component from each of the cohorts was used as their vascular risk factor score (CVRF score).

### Genotyping

Genotyping was performed on different platforms separately for each study cohort. Data from all cohorts underwent quality control (QC). For African American and white, non-Hispanics WHICAP participants, variants with missing rate greater than 5%, out of Hardy–Weinberg equilibrium (*p* < 1 × 10^–6^), or with less than 1% minor allele frequency (MAF) were removed. Participants were excluded if they were missing more than 2% of variants present in the overall cohort. Following quality control (QC), participants were imputed separately based on self-reported ethnicity. Imputation was performed using the HRC r1.1.2016 reference panel and SHAPEIT phasing on the Michigan Imputation Server [26]. Data from the WHICAP Caribbean Hispanics and EFIGA Caribbean Hispanics underwent QC and imputation in seven batches that included participants from both studies, consistent with the genotyping batch. Variants with missing rate greater than 5%, MAF ≤ 1%, out of Hardy–Weinberg equilibrium (*p* < 1 × 10^–6^) were removed. Samples with missing call rate greater than 5% were excluded. SHAPEIT2 [28] was used for phasing and IMPUTE2 was used for imputation with the HRC r1.1.2016 reference panel.

Quality control and imputation details for the ROSMAP have been reported previously [4]. Briefly, variants missing in more than 5%, MAF ≤ 1%, out of Hardy–Weinberg equilibrium (*p* > 0.001), and a *p* value of mishap test < 1 × 10^–9^ were removed. Imputation was performed using the HRC r1.1.2016 reference panel of Caucasian ancestry and Eagle v2.3 phasing. ADC genotype data from waves 1–10 underwent QC by the Alzheimer’s Disease Genetic Consortium as described previously [52]. Imputation was performed on the Michigan Imputation Server individually for each of the cohorts including all ethnicities of the Haplotype Reference Consortium (HRC) 1.1 reference panel. EAGLE was used for phasing and Minimac3 was used for imputation.

NACC and ROSMAP cohorts included only participants that self-reported as non-Hispanic White. For all cohorts, post-imputation genotype data were filtered for imputation quality (*R*^2^ > 0.8) and minor allele frequency (> 1%). KING[60] was used to perform multidimensional scaling (MDS) to identify population substructure within each cohort. Participants more than six standard deviations from the mean within cohort in the first three calculated MDS components were removed from analysis.

### Statistical Analyses

Study design and analyses are described in Supplementary Fig. 1, online resource. Differences in allele frequency were present across ethnic groups, thus we performed genome-wide gene-CVRF score interaction analysis independently in each cohort and summarized the results in a meta-analysis to improve analysis power and results interpretability. Gene-based tests were performed using the adaptive gene-environment interaction (aGE) test [20]. Independently, we validated the results using an alternative method implemented in the gene-environment set association test (GESAT) [56]. Both tests allowed for gene-based analyses with an interaction between the vascular risk factor variable and multiple variants within a gene. GESAT uses a variance component test while, aGE combines variance-component and burden tests to maximize power across broader association patterns. We observed that aGE controls the type I error rate in the presence of many neutral variants, and the power is resilient to the inclusion of neutral variants [20]. Therefore, we used the aGE to test for the genes that interacted with CVRFs to alter AD risk. Additionally, we used GESAT to confirm the robustness of statistically significant results reported from the aGE. Covariates included in the model were age at diagnosis for AD and age at last visit for the unaffected participants, sex, and the first three principal components to adjust for population substructure. We analyzed genes with a minimum of two variants with allele frequency greater than 0.01 in the gene-based test. The aGE R package and GESAT functions in the iSKAT R package were performed with default settings. Both tests only export *p* value of testing the gene-CVRF score interaction term. We combined the results in a meta-analysis using weighted sum of *Z*-scores method through METAL [96]. The *p* value of the interaction term in each cohort was first converted into signed *Z*-score and then calculated as the weighted mean of the *Z*-scores, with square-root of the sample size from each cohort as weights.

Individual SNPs were tested for CVRF interactions within each gene when a significant gene by vascular risk factors interaction was found. The SNP by CVRF interaction test was performed in each cohort for all SNPs within candidate genes by fitting a logistic regression for AD as the outcome and testing for the interaction term of SNP by CVRF score. The interaction tests were adjusted for the main genetic effect and the CVRF score as covariates in all models, in addition to age, sex, and the first three principal components. The models were tested with additive coding for the SNPs. For models used in the NACC cohort, batch effects were included as covariates because genotyping from ten waves were imputed separately. The results were then combined in a meta-analysis using inverse-variance weighted average method through METAL [96] by calculating the weighted mean of the SNP-CVRF interaction effect sizes, with the inverse variance from each cohort as weights.

Results were shown for SNPs and genes present in at least 10,000 participants. Statistical significance was set at *p* = 5 × 10^–6^ for the genome-wide gene-based tests and used the false discovery rate (FDR) adjusted *p* value or Bonferroni correction for the number of genes tested.

### RNA expression analysis

As shown in Table 3, we performed association analyses of pathologically diagnosed AD using the multi-region brain transcriptomes from ROSMAP [7–9] and a replication analysis was performed using the transcriptomes from Mount Sinai Medical Center [94] and Mayo Clinic Brain Banks [2]. A logistic regression was fitted for pathological AD as the outcome while adjusting for age, sex, and processing factors. The bulk RNA-sequencing (RNA-Seq) transcriptomic profiles in ROSMAP were accessed from three brain regions—the dorsolateral prefrontal cortex (DLPFC) in 1,092 individuals, the posterior cingulate cortex (PCC) in 661 individuals and the anterior caudate (AC) in 731 individuals. For the MSBB tissues, the transcriptomic profiles were accessed from four brain regions—the frontal pole (BM10) in 214 individuals, the superior temporal gyrus (BM22) in 191 individuals, the parahippocampal gyrus (BM36) in 161 individuals, the inferior frontal gyrus (BM44) in 186 individuals. For the Mayo Clinic brain tissues, the transcriptomic profiles were accessed from the temporal cortex (TCX) in 261 individuals and the cerebellum (CBE) in 262 individuals.

RNA sequencing data in ROSMAP was generated in multiple batches from different sequencing centers and experimental protocols. RNA sequencing of dorsolateral prefrontal cortex (DLPFC) was first done in 10 batches for 739 subjects at the Broad Institute. Subsequently, 124 subjects in 2 batches were sequenced at the New York Genome Center. Moreover, 229 samples in a single batch were sequenced at the Rush Alzheimer’s Disease Center. A detailed description of the study was previously published [101]. RNA sequencing of anterior caudate (AC) was done in 76 subjects in a single batch at the Broad Institute using the Illumina TruSeq method, as described above in DLPFC. Subsequently, 655 subjects in 2 batches were sequenced at the New York Genome Center using the same experimental protocols described in DLPFC. RNA sequencing of posterior cingulate cortex (PCC) was done in 79 subjects in a single batch at the Broad Institute using the Illumina TruSeq method. Subsequently, 487 subjects in 2 batches were sequenced at the New York Genome Center and 95 subjects in a single batch were sequenced at the Rush Alzheimer’s Disease Center. The experimental protocols of each sequencing center are same as described in DLPFC.

Each brain region was pre-processed separately. We used gene-level transcription values derived from the RNA sequencing data. The counts values were used to measure the gene expression levels and subsequently normalized using Trimmed means of M-values (TMM) to create a frozen dataset that are available on Synapse (Synapse: syn25741873). Outlier samples were removed based on quantified expression profiles and lowly expressed genes with a median Counts less than 10 were filtered out to reduce the influence of technical noise. A linear regression was fitted for each transcript with the log2-transformed normalized values as outcome adjusted for age, sex, and processing factors, and used residual from the transcript as our final transcription dataset. The residuals represent a quantitative trait capturing variability in transcripts outcome not captured by known demographics or technical factors. Technical factors for DLPFC include batch, library size, percentage of aligned reads, percentage of coding bases, percentage of intergenic bases, percentage of ribosomal bases, percentage of UTR base, percentage of duplication, median 3 prime bias, median 5 prime to 3 prime bias, median CV coverage, pmi, and study index of ROS or MAP. Technical factors for AC and PCC are very similar. A detailed description of outlier removal, normalization, and calculation of the residuals in MSBB and Mayo are described elsewhere [93].

We assumed that differences in candidate gene expression would be associated with brain infarcts. A logistic regression was fitted for brain infarcts as the outcome and tested for the candidate gene expression while adjusting for age, sex, and processing factors. Details of neuropathological evaluation and qualifying determinants of infarcts in the ROSMAP dataset have been reported previously [75].

We performed association analyses of pathologically diagnosed AD with expression level of candidate gene across cells of the dorsolateral prefrontal cortex in 24 individuals from the ROSMAP cohort. A logistic regression was fitted for pathological AD as the outcome while adjusting for age, sex. A detailed description of the ROSMAP single nucleus RNA sequencing data are described elsewhere [3].

### Causal mediation analysis

To quantify the involvement of gene expression in aging-related brain pathology in AD, we investigated whether the effect of gene expression on cerebrovascular pathology in AD brain would be mediated by AD specific pathology. We also investigated whether the effect of the AD pathology in brain infarcts would be mediated by candidate gene expression. We used a causal mediation analysis aimed at identifying whether the candidate expression resulted from amyloid or phosphorylated tau deposition or the reverse. In the mediation analysis, the mediated effect refers to an indirect effect of the exposure on outcome through a mediator. Direct effect refers to the effect of the exposure on the outcome after adjusting for the mediator. Total effect refers to the total effect of the exposure on the outcome, obtained by combining direct and indirect effects. Causal mediation modeling was performed using the R package mediation and confidence intervals were obtained by nonparametric bootstrap procedure with 1000 resamples [85]. In parallel, we performed cell type specific analyses of the expression of our gene of interest.

### Human brain sections and immunohistochemistry

Human brain sections from BA9 prefrontal cortex were also obtained from the New York Brain Bank at Columbia University (Table S14) and immunohistochemical staining for FMNL2 and GFAP were performed as described [92] with the following modifications: heat-induced antigen retrieval was performed with pressure cooker, and concomitant biotin-based immunodetection was performed. Nine random images per patient from the sections were acquired with identical acquisition parameters to control brains and Arivis-based quantification was performed (script available upon request). The analyses were based on the overlapping surface area of GFAP and FMNL2 that is normalized to the total GFAP surface area. Automated masking was quality controlled manually; images were excluded from the analyses if machine-predicted surfaces show substantial difference to manual selections. Analyses were performed in blind fashion: sample IDs were revealed after immunohistochemical stainings and quantifications for individual samples. Statistical comparisons were performed with Dunn’s Kruskal–Wallis test and linear mixed effects model.

### Mouse studies

#### Ethics statement and animal maintenance

All animal studies followed European animal regulations and were approved by the appropriate authority (Landesdirektion Sachsen, Germany) under license number TVV87/2016. To limit suffering and overall animal numbers, animals were treated with extraordinary caution. Mice were maintained on a 12-h alternating light and dark cycle, with free access to conventional mouse diet (chow) and water. Animals were housed in groups in normal ventilated cages, and after the surgeries, they were maintained in individual cages. Fixed gender mice aged between 52 and 54 weeks were used for analysis. Jackson Laboratories (Bar Harbor, ME, USA) provided the B6.Cg-Tg(APP695)3DboTG(PSEN1dE) mice, which were kept as a heterozygous breeding colony.

### Stereotaxic injection for mouse brain injury

The brain tissue injury was induced by stereotaxic injection. The mouse was anesthetized with a combination of oxygen and isoflurane (49:1) (Baxter–HDG9623) flow during the procedure and put on a pre-warmed heat-pad to avoid hypothermia. Ear bars were used to keep the head immobile, and a protective ointment was applied to the eyes to prevent cornea dehydration. An analgesic was administered subcutaneously before to the surgery to reduce any potential pain afterward. The injury was caused in the right hemisphere, coordinates were ± 1.6 mm mediolateral, − 1.9 mm anterior–posterior, and − 1.9 mm dorsoventral from the Bregma, where the PBS was dispensed at 200 nL/min speed. The left hemisphere was used as a control for the analysis. The capillary was progressively retracted after the injection, followed by the ear being released. Mouse brain was analyzed 3 days after the injury via immunohistochemistry.

### Tissue preparation

An overdose of Ketamine/Xylazin (0.25 mL per 25 g of body weight) was used to euthanize the mice, which were then transcardially perfused with NaCl (0.9 percent w/v) and 4% paraformaldehyde (PFA). Brains were extracted and post-fixed in 4% PFA overnight at 4 °C. Brains were placed in a 30% sucrose solution for 2–3 days to cryopreserve the fixed tissue. A sliding microtome (Leica SM2010) chilled with dry ice was used to cut coronal sections with a thickness of 40 μm. Free-floating sections were collected in six series and kept at − 20 °C in cryoprotection solution (CPS; 25% ethylene glycol, 25% glycerol in 0.1 M phosphate buffer pH 7.4). For immunohistochemistry, every sixth section series of each brain was used.

### Immunohistochemistry

Free-floating sections were rinsed three times in PBS before being blocked in a 10% Donkey Serum, 0.3% Tween 20, and 1 × PBS solution for 1 h at room temperature. Primary antibodies were diluted in PBS containing 3% donkey serum and 0.3% Tween-20 and sections were treated overnight at 4 °C. After 3 washes with PBS, secondary antibody conjugated with a chosen fluorophore was then incubated for four hours at room temperature. After a quick wash, samples are incubated for 15 min in 4,6-diamidino-2-phenylindole (DAPI) diluted in PBS. Additional washing steps were performed, and samples were mounted on glass slides.

### Imaging

Fluorescence images were acquired using a spinning disc Zeiss Axio Observer.Z1 microscope (Oberkochen, Germany) equipped with ZEN software (version blue edition, v3.2, company, Carl Zeiss, Jena, Germany).

### Zebrafish studies

#### Ethics statement

Animal experiments were carried out in accordance with the animal experimentation permits of Referate 24 (Veterinärwesen, Pharmazie, und GMP) of the state administration office of Saxony, Germany (Landesdirektion Sachsen), the ethical commission of TU Dresden (Kommission für Tierversuche), and the Institutional Animal Care and Use Committee (IACUC) at Columbia University (protocol number AC-AABN3554). Zebrafish handling and maintenance was according to the provided guidelines [1, 32, 51, 79] and EU Directive 2010/63 Article 33 and Annex III (permit numbers: TVV-35/2016, TVV-52/2015, TVV31/2019, and TVV39/2020). For zebrafish studies, 8–12 months old wild type AB strain, Tg(*her4.1*:GFP) [100] and Tg(*kdrl:*GFP) [40] reporter fish of both genders were used. In every experimental set, animals from the same fish clutch were randomly distributed for each experimental condition.

### Amyloid-β42 (Aβ42) and morpholino injection

Cerebroventricular microinjection of Amyloid-β42 peptides in adult zebrafish brain were performed as previously described [13]. Morpholinos were injected to embryos and adult zebrafish brains as described [11, 49]. For morpholino experiment, 10 μM concentration of control morpholinos, control morpholinos with Aβ42 or *fmnl2a* + *fmnl2b* morpholinos with Aβ42 were injected to adults. For embryos, 2 ng of morpholinos were injected. See Table S15 for more information on the reagents.

### Histological preparation of zebrafish tissue and immunohistochemistry

Euthanasia and tissue preparation were performed as described [13]. 12-µm thick cryo-sections were prepared from these brain samples using a cryostat and collected onto glass slides which were then stored at − 20 °C. Immunohistochemistry was performed as described [13].

### Imaging, quantifications, and statistical analyses

Images were acquired using ZEN software on a Zeiss fluorescent microscope with ApoTome or Zeiss Spinning Disc microscope. Images were analyzed using ZEN or Arivis Image processing software. For glial-vasculature interaction, automated quantification pipeline for at least 50% overlap between GFP (vasculature) and S100β (glia) was developed in Arivis software (script available upon request). Quantifications were performed on *z*-stacks images obtained from the telencephalon sections (region between caudal olfactory bulb until the rostral optic tectum). 6–8 histological sections per animal were used for stereological analyses and quantifications. For colocalization in Fig. 2, ImageJ version 2.1.0/1.53c was used with default settings. A two-tailed Student’s *t*-test was performed for comparison of two experimental groups, one-way ANOVA with Tukey’s multiple comparison test were performed for comparison of multiple experimental groups. Statistical analyses were performed in GraphPad Prism software. Bars in the graphs indicate the mean values and 95% CI. *p* values less than 0.05 were considered significant. Significance is indicated by ∗(*p* < 0.05), ∗∗(*p* < 0.01), ∗∗∗(*p* < 0.001) or n.s. (not significant, *p* > 0.05). No sample set was excluded from the analyses unless the histological sections were damaged severely during the acquisition of the sections (constitutes less than 5% of all sections analyzed).

## Results

### Genetic analyses

Genome-wide array data were available from 10,287 non-Hispanic whites, 3404 Caribbean Hispanics and 978 African–Americans (Table 1). The majority of the participants were women (65%) and had a history of hypertension (70%).

A history of heart disease, hypertension and diabetes and measured BMI in the different cohorts were modestly correlated (spearman correlation coefficient ranging from 0.03 to 0.26, Supplementary Fig. 2. Principal component (PC) analysis was performed to create the CVRF score, summarizing the joint effect of the four risk factors for each cohort. The first principal component was used to represent the CVRF score, Supplementary Fig. 1 shows the contribution of each risk factor to the score. In all cohorts, diabetes and hypertension were highly correlated and most strongly influenced the CVRF score. The percentage of variance explained by principal component 1 for each ethnic group were: African–Americans 34.7%, non-Hispanics Whites 35.5%, Caribbean Hispanics 38.6%. Variance explained by the first four principal components and contributions of the risk factors to each component is also described in Table S1.

In a gene by CVRF interaction analysis of AD, three genes met the false discovery rate (FDR) adjusted *p* value of 0.05 using the adaptive gene-environment interaction (aGE) test (Table 2). One gene located on chromosome 2, Formin-like protein 2 (*FMNL2*), also met the Bonferroni corrected *p* value threshold for significance (*p* = 6.6 × 10^–7^). Cohort-level results showed nominal significance in all ethnic groups except in African-Americans. QQ plots for each cohort are shown in Supplementary Fig. 4. There was no evidence of inflation in any group. This *FMNL2* gene by CVRF score interaction was subsequently validated using gene-environment set association test (GESAT) (*p* = 7.7 × 10^–7^). Other nominally significant genes are reported in Table S2. As an additional measure of *FMNL2* interaction with CVRF, we repeated the interaction test by constructing the CVRF as the sum of first two principal components. *FMNL2-*CVRF interaction was still robustly associated with AD (*p* = 3.47 × 10^–4^). Analyzing each risk factor of the CVRF separately, *FMNL2* interacts most strongly with history of hypertension (*p* = 7.53 × 10^–4^) and BMI (2.51 × 10^–3^) (Table S3).

**Table 2:** Gene-CVRF score interaction results in GWAS: Top ten genes interacted with the CVRF score to modify AD risk, sorted by meta-analysis *p* value

Gene	Chr	*p*_ROSMAP_	*p*_WHICAP AA_	*p*_WHICAP Whites_	*p*_CH_	*p*_NACC_	*p*_Meta_	*p*_FDR_
*FMNL2*	2	0.001	0.189	0.038	0.009	0.012	6.59 × 10^–7^	0.012
*LOC401357*	7	0.172	0.362	0.020	0.001	0.006	1.13 × 10^–6^	0.012
*AMMECR1L*	2	0.940	0.073	0.044	0.005	0.001	2.43 × 10^–6^	0.017
*CFAP99*	4	0.549	0.937	0.424	4.1 × 10^–4^	0.002	1.27 × 10^–5^	0.068
*SLC22A14*	3	0.294	0.425	0.307	0.293	4.90 × 10^–5^	1.81 × 10^–5^	0.070
*PRG3*	11	0.387	0.090	0.012	0.205	0.001	1.96 × 10^–5^	0.070
*PTPRF*	1	0.056	0.563	0.532	0.659	3.10 × 10^–5^	2.84 × 10^–5^	0.087
*PLA2G4E*	15	0.254	0.893	0.219	0.509	3.20 × 10^–5^	4.55 × 10^–5^	0.095
*ACACB*	12	NA	0.184	0.135	0.038	0.004	4.98 × 10^–5^	0.095
*LINC00353*	13	0.004	0.270	0.267	0.106	0.014	5.29 × 10^–5^	0.095

*p*_*cohort*_ *p* value of the gene-CVRF score interaction test in each cohort, *AA* African–Americans, *CH* WHICAP and EFIGA Caribbean Hispanics, *p*_*Meta*_ *p* value of the meta-analyzed gene-CVRF score interaction results from five cohorts, *p*_*FDR*_ FDR adjusted *p* value of the meta-analysis *p* value, *NA* not available

Meta-analysis of single nucleotide variants (SNP) within the *FMNL2* identified 130 nominally significant SNPs (*p* < 0.05) present in at least three cohorts and interacting with the CVRF score (Table S4). Two individual SNPs in *FMNL2* met the Bonferroni corrected *p* value in the interaction model rs57223657 (*p* = 3.61 × 10^–4^) and rs6760139 (*p* = 3.63 × 10^–4^). Random-effects model in meta-analysis was used as comparison and rs57223657 stayed similar (*p* = 3.61 × 10^–4^). Main genetic effect of rs57223657 was significant when adjusted for SNP–CVRF interaction effect and the CVRF score (*p* = 0.004).

### FMNL2 expression in autopsy brains

We assumed that any gene or genes interacting with vascular risk factors to modify AD risk would be directly related to molecular pathways in AD and cerebrovascular disease. Using autopsied brain tissue from the ROSMAP with multi-region brain transcriptomes, higher expression of *FMNL2* was associated with pathological AD in all three regions sampled: the dorsolateral prefrontal cortex (*p* = 0.004), the posterior cingulate cortex (*p* = 0.001) and in the anterior caudate (*p* = 0.012). In the ROSMAP cohort a small number of participants were considered cognitively normal but had autopsy findings confirming hallmarks of AD in brain. As a sensitivity analysis, we tested the association of *FMNL2* expression by restricting the analysis to individuals with both clinical and pathological AD compared them with healthy individuals without ante and post-mortem AD. The *FMNL2* association with AD was even stronger (*p* = 7.89E−08; Table S5). Available data from the Mount Sinai Brain Bank, also showed increase *FMNL2* expression in the parahippocampal gyrus (*p* = 0.0004) in pathological AD, and in data from the Mayo Clinical Brain Bank, *FMNL2* expression was increased in the temporal cortex (*p* = 0.011) in pathological AD (Table 3).

**Table 3:** Association of *FMNL2* expression with pathologic diagnosis of AD using the multi-region brain transcriptomes from three cohorts

Cohort	Brain region	*n*	*b*	SE	*p*
ROSMAP	Dorsolateral prefrontal cortex (DLPFC)	1092	0.582	0.200	0.004
	Posterior cingulate cortex (PCC)	661	1.040	0.316	0.001
	Anterior caudate (AC)	731	0.875	0.350	0.012
MSBB	Frontal pole (MB10)	214	0.369	0.623	0.554
	Superior temporal gyrus (BM22)	191	1.038	0.718	0.149
	Parahippocampal gyrus (BM36)	161	2.970	0.833	0.0004
	Inferior frontal gyrus (BM44)	186	1.235	0.652	0.058
Mayo	Temporal cortex (TCX)	261	1.204	0.473	0.011
	Cerebellum (CBE)	262	− 0.348	0.642	0.588

*n* sample size, *b* estimate, *SE* standard error, *p* *p* value

**Fig. 1:**
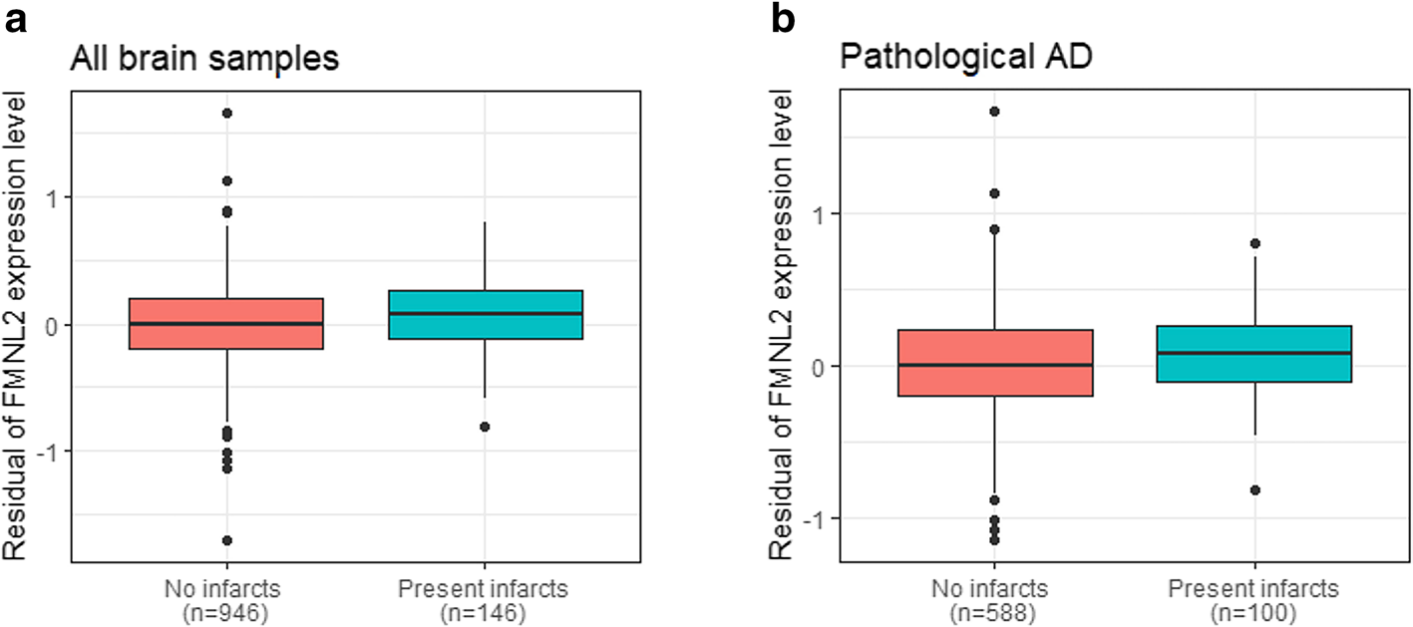
*FMNL2* expression level by brain infarcts. **a** Distribution of expression level of *FMNL2* in those with gross chronic infarction in cortex compared to those without infarction was statistically significant (adjusted for age, sex, and processing factors *b* = 1.024, *p*_FDR_ = 0.003). **b** Distribution of *FMNL2* expression in those with pathological AD and gross chronic infarction in cortex compared to those with pathological AD and no infarction, in the dorsolateral prefrontal cortex from ROSMAP was statistically significant (adjusted *b* = 0.792, *p* = 0.025). A logistic regression was fitted for the brain infarcts as outcome and *FMNL2* expression level (log2-tranformed normalized transcripts per million values) as exposure adjusted for age, sex, and processing factors. For visualization purposes, the residual of *FMNL2* expression level (y-axis) was used to represent the residual of transcripts from fitting the linear regression on the *FMNL2* expression level adjusted for age, sex, and processing factors, capturing variability in transcripts outcome not captured by known demographics or processing factors

Using data from ROSMAP, we found that an increased expression of *FMNL2* in AD brain was associated with Aβ (adjusted *β* = 0.188, *p* = 0.034) and tau deposition (adjusted *β* = 0.415, *p* = 0.011). The expression level of *FMNL2* was also significantly higher in those with gross chronic infarcts in cortex compared to those without infarctions (adjusted for age, sex, and processing factors *β* = 1.024, p _FDR_ = 0.003; Fig. 1a; Table 4). Restricted to pathological AD, *FMNL2* expression was higher in those with, compared to those without, gross chronic infarcts in cortex (adjusted *β* = 0.792, *p* = 0.025). A mediation model suggested that *FMNL2* expression mediates the association of Amyloid-β and phosphorylated tau deposition with brain infarcts, although no direct association was observed between them. The mediation analysis suggested an indirect effect, in which Aβ or tau deposition increased *FMNL2* expression, which was then associated with brain infarcts (mediation effect: *p* = 0.002 amyloid; *p* < 2 × 10^–16^ tau; Table S6). Of interest, the ante-mortem CVRF score was also modestly increased in those with gross chronic infarcts (detected post-mortem) compared to no infarcts (*β* = 0.21, *p* = 0.036).

**Table 4:** Association of expression level of *FMNL2* with brain infarcts in all brain samples in the dorsolateral prefrontal cortex from ROSMAP

Infarcts	*b*	SE	*p*	*p*_FDR_
Gross chronic infarctions in cortex	1.024	0.295	5.21 × 10^–4^	0.003
Gross subacute infarctions in cortex	0.697	0.467	0.135	0.181
Micro subacute infarctions in cortex	0.017	0.542	0.975	0.975
Chronic infarctions in cortex, regardless of size	0.525	0.226	0.020	0.060
Subacute infarctions in cortex, regardless of size	0.519	0.361	0.151	0.181
Infarctions in cortex, regardless of size/age	0.388	0.206	0.060	0.120

*b* estimate, *SE* standard error, *p* *p* value, *p*_*FDR*_ FDR adjusted *p* value. The expression of *FMNL2* was increased in pathological AD with gross chronic infarcts compared to pathological AD without gross chronic infarcts (*b* = 0.792, *p* = 0.025)

Rs77136812 in *FMNL2* was among 14 SNPs that were correlated with *FMNL2* expression (*β* = 0.118, *p* = 0.027) (Table S7). In addition, SNP rs4664586 was associated with hypomethylation of several CPG sites surrounding the *FMNL2* gene, the strongest having a *p* value of 6.75 × 10^–7^, indicated that one of these variants might also have regulatory effects on expression (Table S8).

We then tested the cell-specific expression of *FMNL2* using data from single nucleus RNA-sequencing of the frontal cortex in 24 individuals from the ROSMAP cohort. *FMNL2* was highly expressed (*p* = 0.024) in a subset of astrocytes enriched in interferon response genes, derived from AD brains compared to brain tissue from non-demented individuals.

### Zebrafish model

To determine the putative evolution of *FMNL2* expression changes in pathological AD, we used a previously described in vivo zebrafish model of amyloidosis [11–13, 24]. The zebrafish model of amyloid β pathology is an acute model where injection of amyloid peptides leads to an intracellular aggregation/polymerization of amyloid peptides (Supplementary Fig. 5), which lead to phenotypes reminiscent of the alterations in mammalian systems (e.g., cell death, synaptic degeneration, inflammation, glial hypertrophy and cognitive impairment) [12–14]. This model allows to mechanistically investigate the effects of amyloidosis in a vertebrate brain within a short time, and the molecular understanding from zebrafish model is relevant to mammalian models and human AD [48, 50, 66, 69, 77].

**Fig. 2:**
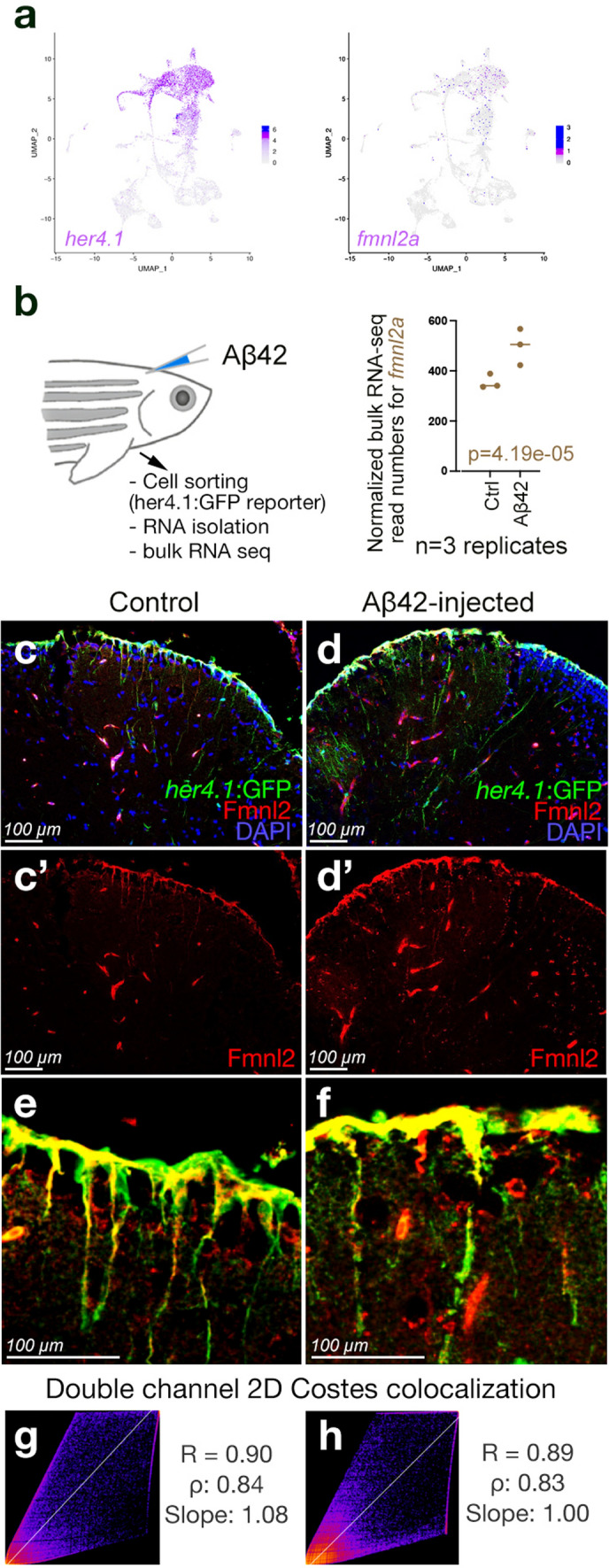
Expression of *fmnl2a* in zebrafish brain overlaps with astroglia. **a** Singe cell sequencing tSNE plots for *her4.1* and *fmnl2a* in adult zebrafish telencephalon. **b** Amyloid toxicity assay and bulk RNA sequencing results for *fmnl2a* gene expression*.* **c**, **d** Double immunohistochemical staining (dIHCs) for her4.1: GFP (astroglia) and Fmnl2 in control (**c**) and amyloid-injected (**d**) brains with DAPI counterstain. **c′**, **d′** individual fluorescent channels for Fmnl2. **e**, **f** Higher magnification image showing overlapping Fmnl2 and her4.1-driven GFP. **g**, **h** Double channel colocalization analyses of **e** and **f**. *R* Pearson correlation coefficient, *ρ* Spearmann’s rank coefficient. Scale bars as indicated

Based on our existing zebrafish single cell sequencing data, we determined that *fmnl2a,* a zebrafish ortholog of human *FMNL2,* is expressed in *her4.1*-positive glial cells (Fig. 2a). To confirm this, we analyzed the bulk RNA-seq data from sorted *her4.1*-positive glial in transgenic reporter line Tg(*her4.1*:GFP), which marked the astroglial cells in adult zebrafish in control and amyloid-β42-injected brains. We found that amyloid deposition significantly increased the expression of *fmnl2a* in astroglia (Fig. 2a)*.* Fmnl2 is localized to the cell bodies and the projections of the astroglia in the healthy adult zebrafish brain as determined by immunohistochemistry with an antibody cross-reactive to FMNL2 protein (Fig. 2c, c′). Amyloid β increased the FMNL2 reactivity levels in the astroglia consistent with the gliotic hypertrophy (Fig. 2d, d′). FMNL2 and her.1-driven GFP expression strongly correlates, indicating that these proteins co-localize in astroglial cells (Fig. 2g, h). We found that the other ortholog of human *FMNL2* gene in zebrafish, *fmnl2b,* is primarily expressed in neuronal populations as determined by single cell sequencing (Supplementary Fig. 6a). We determined the specificity of the FMNL2 antibody in zebrafish by performing the antibody staining only with the secondary antibody, which resulted in no signal in glial extensions and punctate staining in the parenchyma (Supplementary Fig. 6b), indicating that Fmnl2 immunohistochemistry in zebrafish is detecting true signals. Neuronal and glial Fmnl2 immunoreactivity can be distinguished in neurons and glia by the signal in the radial extensions (glial Fmnl2) and parenchymal perinuclear staining (neuronal Fmnl2) (Supplementary Fig. 6c).

**Fig. 3:**
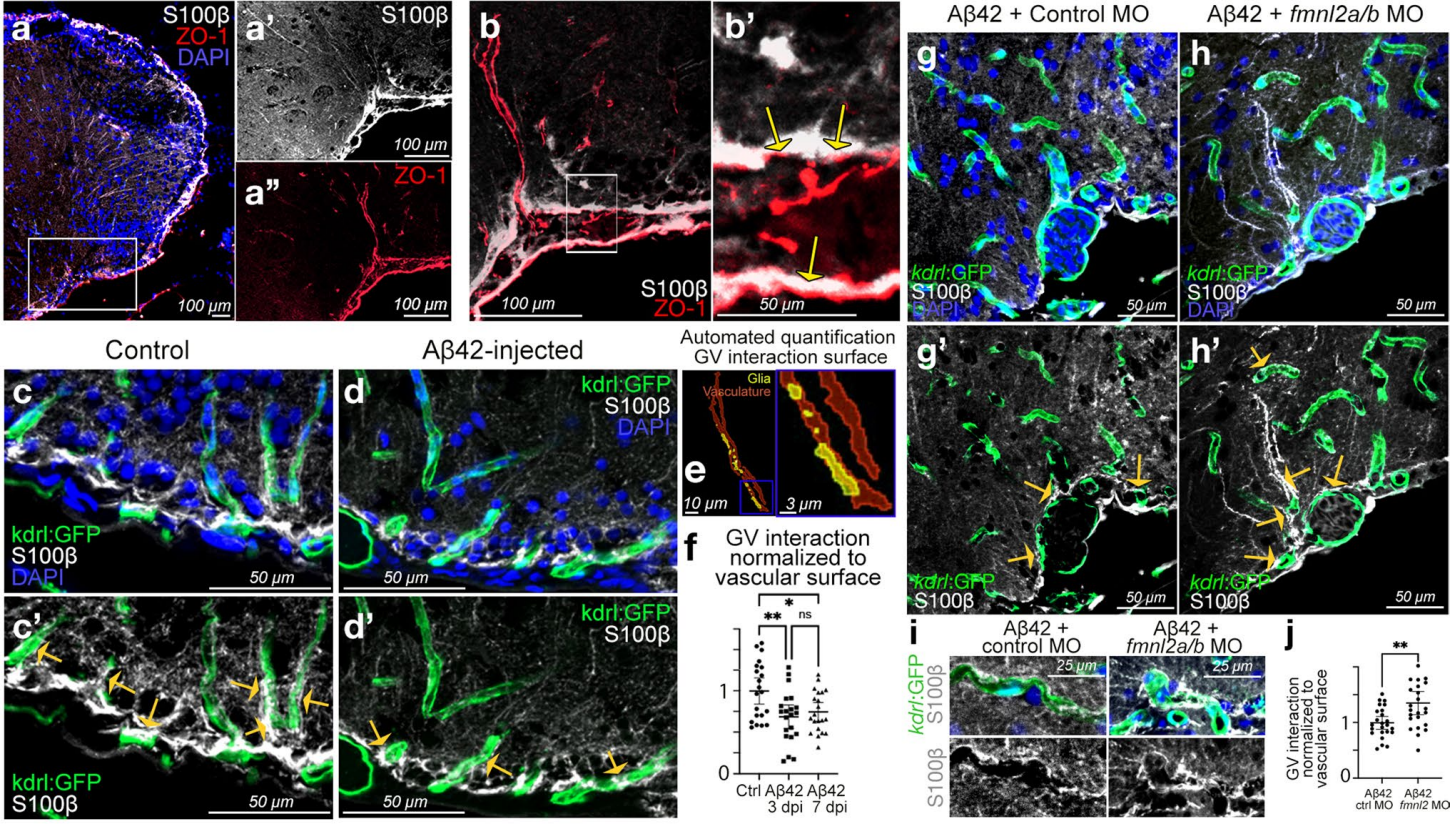
Fmnl2 is required for remodeling of gliovascular interactions. **a** dIHCs for S100β (astroglia) and ZO-1 (tight junctions/vessels) with DAPI counterstain. **a**′, **a**′′ Individual channels for the inset in **a**. **b** Higher magnification view of dorsoventral gliovascular (GV) junctions. **b**′ Close-up of inset in **b**. Arrows indicate overlaps. **c**, **d** dIHCs for kdrl:GFP (blood vessels) and S100β in control (**c**) and amyloid-injected (**d**) brains with DAPI counterstain. **c**′, **d**′ DAPI omitted from **c** and **d**. **e** Representative snapshot of the automated quantification of the surface of gliovascular interactions. **f** Quantification results of GV interactions. **g**, **h** dIHCs for kdrl:GFP and S100β in amyloid injected brains that were co-injected with control morpholino (**g**) and *fmnl2a/b* morpholino (**h**) with DAPI counterstain. **g**′, **h**′ DAPI omitted from **g** and **h**. **i** Close-up image of blood vessels interacting with glial endfeet. **j** Quantification results of GV interactions. Yellow arrows in **c**′, **d**′, **g**′ and **h**′ indicate exemplary gliovascular interactions. Scale bars as indicated

The radially elongated endfeet of astroglia (S100β) in zebrafish telencephalon coalesce at ventrolateral blood vessels (ZO-1) (Fig. 3a–a′′), which displayed colocalized astroglial endfeet and tight junctions of the blood vessels (Fig. 3b, b′). This interaction was verified using a transgenic zebrafish reporter line Tg(*kdrl*:GFP), which marked the endothelial cells of the blood vessels and S100β immunolabeling that marked the astrocytic endfeet (Fig. 3c, c′). To determine if these gliovascular interactions are affected in amyloid pathology, we performed immunohistochemical staining in amyloid β-injected animals and found that gliovascular interactions were less pronounced compared to the control (Fig. 3d, d′). To determine this change in a quantitative manner, we developed an automated image analyses pipeline that determines the colocalization of glial endfeet and blood vessels (Fig. 3e) and found that the surface area of gliovascular interactions reduced significantly upon amyloid β (Fig. 3f; Table S9). This result suggest that astroglial-vascular contacts undergo a relaxation upon amyloid β toxicity.

To determine the role of Fmnl2 in the dynamic regulation of gliovascular interactions and amyloid deposition, we knocked down Fmnl2 activity by cerebroventricular microinjection of *fmnl2*-targeting morpholinos, which were previously verified in zebrafish [91] and we also determined to be effective for reducing Fmnl2 expression in zebrafish (Supplementary Fig. 6d). Cerebroventricular microinjection targets mainly the ventricular cells and therefore would effectively knock-down Fmnl2 in astroglia. We found that relaxation of gliovascular interactions upon amyloid toxicity was negatively affected by *fmnl2* knock-down in zebrafish brains (Fig. 3g–j; Table S10), which suggested that gliovascular remodeling in response to amyloid toxicity requires Fmnl2 function.

**Fig. 4:**
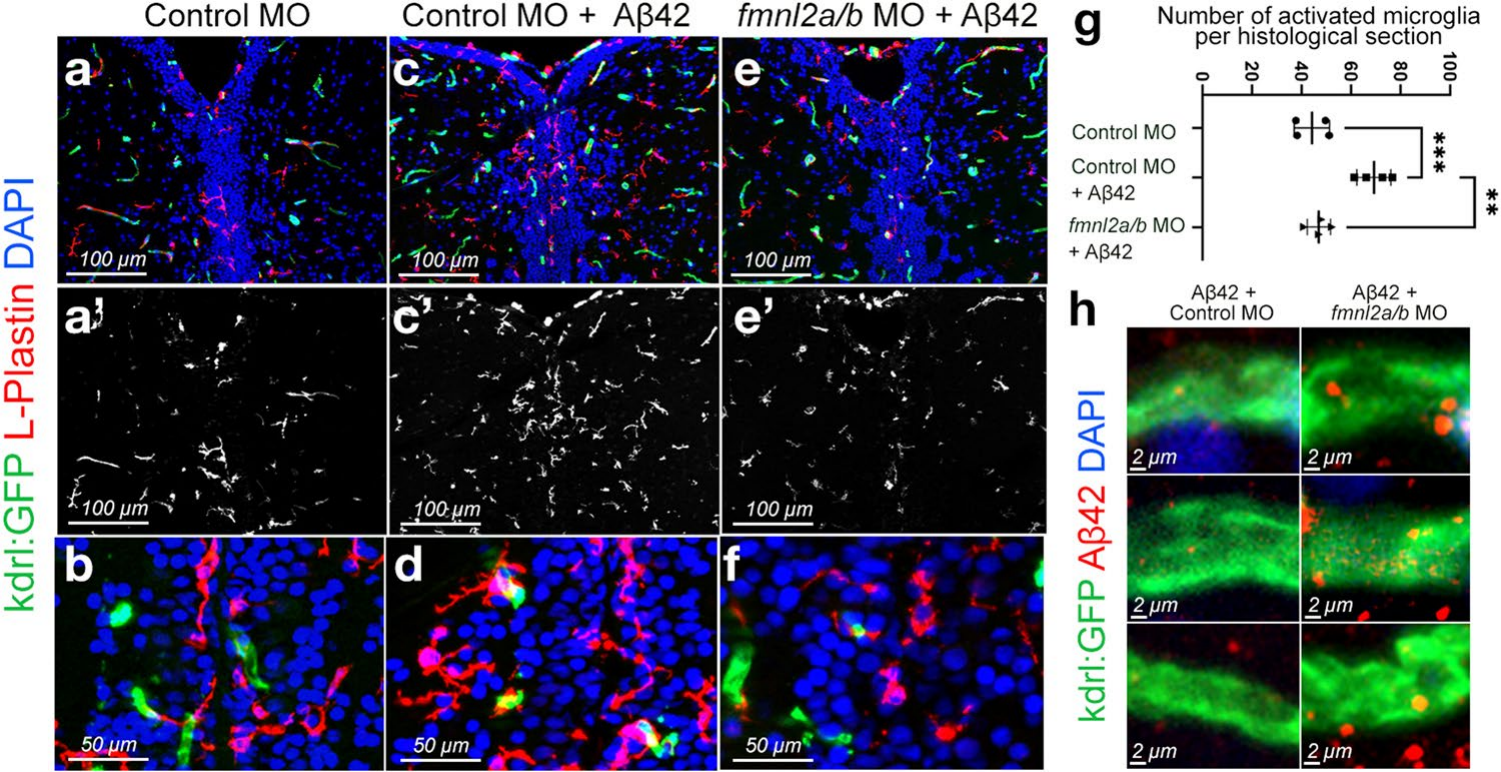
Fmnl2 knock-down reduces the number of activated microglia and alters the amyloid load. **a**–**f** dIHCs for L-plastin (microglia) and kdrl:GFP with DAPI counterstain on control morpholino (**a**, **b**), Aβ42+ control morpholino (**c**, **d**), and Aβ42+ *fmnl2a/b* morpholino (**e**, **f**) injected brains. **a**′, **b**′, **c**′ L-plastin channel alone. **g** Quantification graph for the number of activated microglia in the indicated conditions. **h** High magnification images from three blood vessels in Aβ42+ control morpholino-injected zebrafish brains (left column) and Aβ42+ *fmnl2a/b* morpholino-injected brains (right column). Scale bars as indicated

Defects in blood–brain barrier integrity are associated with several chronic diseases. An increase in blood–brain barrier permeability, required for the passage of toxic aggregates from brain to blood as well as for immune cells to infiltrate the brain to attack the toxic entities at earlier stages of disease progression [25], suggests that stage-specific regulation of gliovascular interactions modulates the cerebrovascular pathology in AD. To determine whether the microglial interactions with the blood vessels are affected by Fmnl2 function, we performed immunohistochemical staining for L-plastin (microglial marker) in relation to the blood vessels (Fig. 4a–f). We found that while amyloid β injection increased the microglia-vasculature contact compared to control animals, and *fmnl2* knock-down reduced the extent of this contact and the number of activated microglia (Fig. 4g; Table S11). These results suggest that the remodeling of the gliovascular interactions by *fmnl2* may be a critical factor for microglial dynamics or leukocyte extravasation from the blood vessels.

Microglial activity and vascular dynamics are important for clearance mechanisms in the brain. If Fmnl2 knock-down resulted in reduced number of activated microglia, it may have an impact on amyloid clearance as well. Therefore, we analyzed whether Fmnl2 knock-down could alter the clearance and vascular deposition of amyloid β. After acute amyloidosis through microinjection of amyloid, we performed immunostaining for amyloid β, and analyzed the extent of amyloid aggregation near the blood vessels with and without *fmnl2* knock-down (Fig. 4h). We observed that *fmnl2* knock-down zebrafish brains showed higher levels of amyloid aggregation around the blood vessels compared to control animals, which could be due to impaired amyloid clearance because of reduced number of activated microglia (Fig. 4h). These findings suggest that relaxed gliovascular interactions through the activity of *Fmnl2* is required for amyloid clearance through bloodstream and/or interaction of the immune cells with the vasculature, but not for blood vessel development (Supplementary Fig. 6d-f; Tables S9 and S12).

### Mouse model of AD recapitulates gliovascular remodeling and upregulation of Fmnl2

**Fig. 5:**
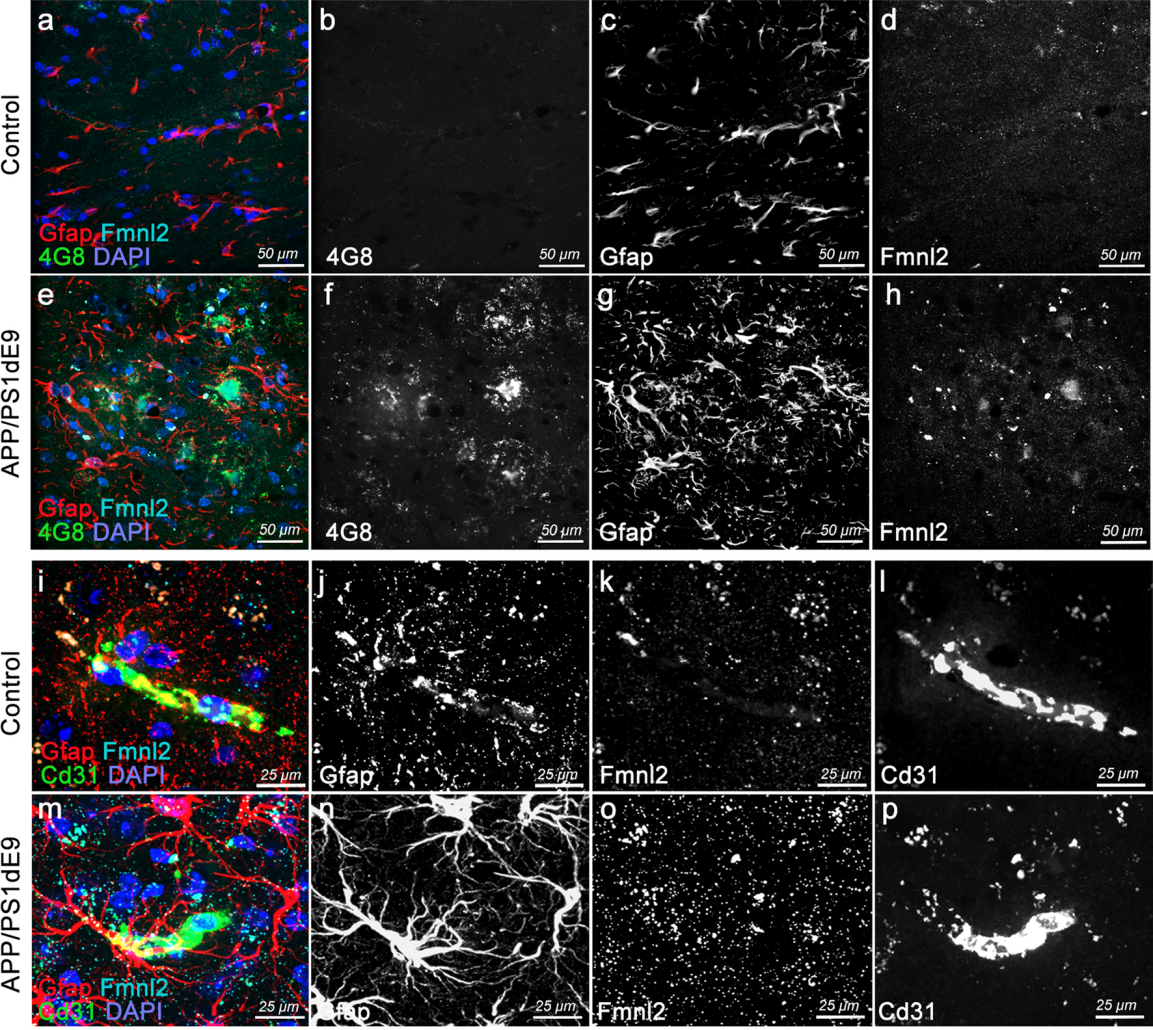
Upregulated Fmnl2 and altered gliovascular interactions observed in chronic APP/PS1dE9 AD model in mice. **a**, **h** Triple immunohistochemical stainings (tIHC) for Gfap (astroglia), 4G8 (amyloid plaques) and Fmnl2 with DAPI counterstain in control (**a**, **d**) and APP/PS1dE9 mice (**e**–**h**) at 12 months of age. Panels are from cerebral cortex. Black-white panels are individual fluorescence channels of the composite images in **a** and **e**. **i–p** tIHC for Gfap, Cd31 (blood vessel) and Fmnl2 with DAPI counterstain in control (**i**–**l**) and APP/PS1dE9 mice (**m**–**p**) at 12 months of age. Panels are from cerebral cortex. Black-white panels are individual fluorescence channels of the composite images in **i** and **m**. Scale bars as indicated

To determine whether chronic amyloid pathology in mammalian models of AD would alter Fmnl2 expression and gliovascular remodeling, we utilized a well-established AD model APP/PS1dE9 [39] (Fig. 5). Compared to age-matched control mice (12 months old) (Fig. 5a–d), APP/PS1dE9 mice shows extensive amyloid plaques (Fig. 5e, f), gliosis (Fig. 5g) and elevated levels of Fmnl2 (Fig. 5h), supporting our previous findings. To determine whether gliovascular interactions are also altered in mice upon AD pathology, we performed triple immunostaining for Gfap (astroglia), Fmnl2 and Cd31 (blood vessels) (Fig. 5i–p). We observed that concomitant to elevated levels of Fmnl2 and gliosis, astroglial interactions with the blood vessels are reduced with pathology (Fig. 5i, m). Fmnl2 staining was quality controlled by excluding the primary antibody, which confirmed that the signal for Fmnl2 is specific (Supplementary Fig. 7). These results indicate that in acute or chronic amyloid pathology models in zebrafish and mouse, upregulation of Fmnl2 and reduction in gliovascular interactions are consistent.

AD pathology is strongly associated with a gliotic response in astroglia, and the gliovascular remodeling response could be a generic outcome due to gliosis. To test whether Fmnl2 upregulation and relaxation of the gliovascular interactions are dependent on gliosis, we performed traumatic injury in the cerebral cortex of healthy mouse brains (Supplementary Fig. 8). Compared to control mice, injured brains showed extensive gliotic response; yet the gliovascular interactions and the levels of Fmnl2 were not significantly altered (Supplementary Fig. 8). These results suggest that the remodeling of gliovascular interactions and concomitant upregulation of Fmnl2 could be a specific response to amyloid pathology.

### Validation in post-mortem human brains

**Fig. 6:**
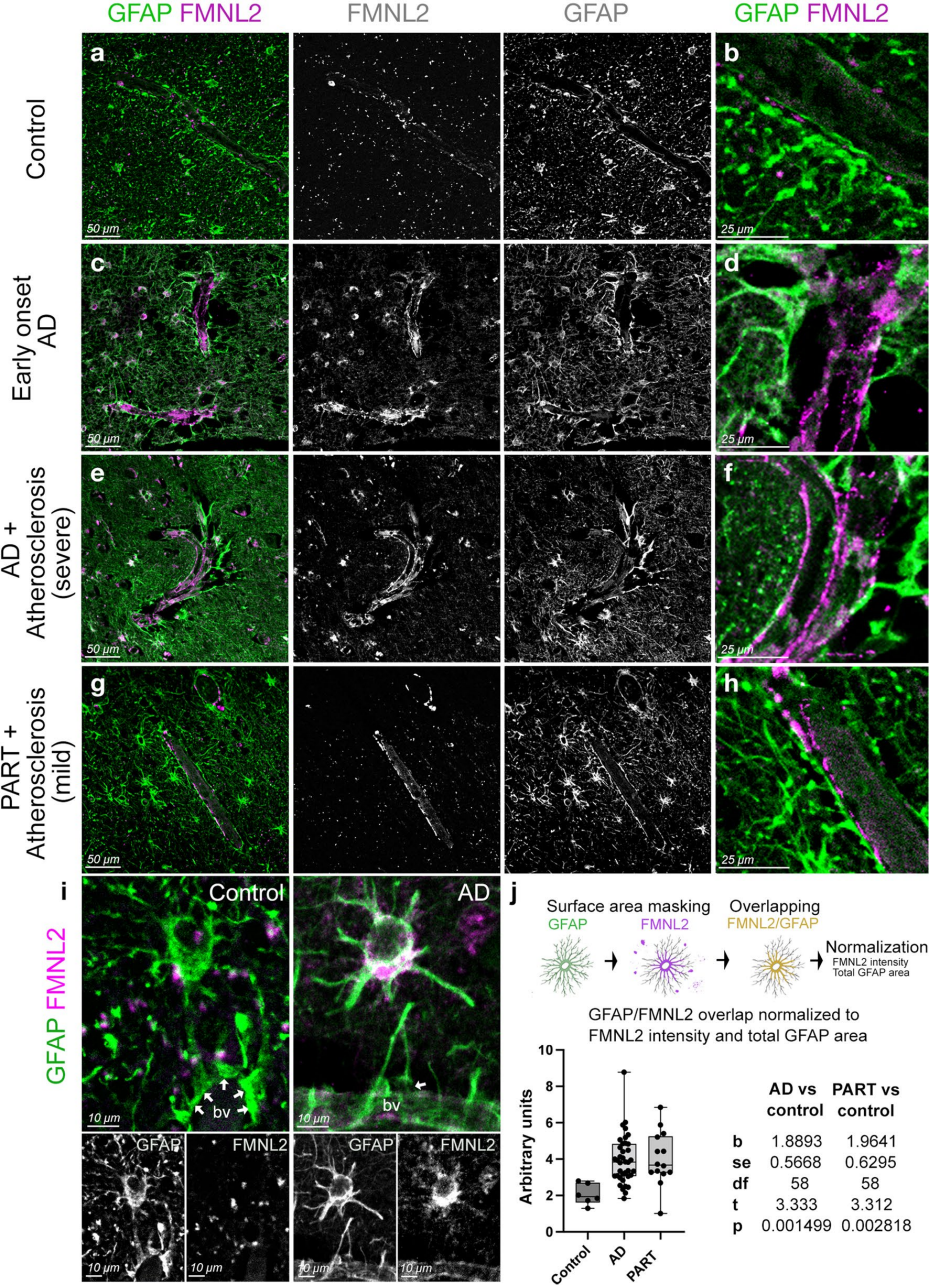
FMNL2 expression is upregulated at gliovascular junctions in AD patients. **a**–**h** dIHC for FMNL2 and GFAP in control (**a**, **b**), early onset AD patient (**c**, **d**), AD patient with severe atherosclerosis (**e**, **f**), and primary age-related Tauopathy (PART) with mild atherosclerosis patient (**g**, **h**). Black and white images are individual fluorescent channels of the leftmost panels for FMNL2 and GFAP. Rightmost panels (**b**, **d**, **f**, **h**) are higher-magnification composite images around a representative blood vessel. **i** Two examples of astroglia (GFAP+, green) contacting blood vessels (bv), and their expression of FMNL2 (violet). Arrows indicate contact with blood vessels. In AD, astroglia express FMNL2 and its endfeet is retracted from the blood vessel, while in controls tight association is observed. Black-white panels indicate individual fluorescence channels. **j** Arivis-based automated image quantification pipeline and the quantification graph for FMNL2 expression in human brains. Comparisons are for every diseased brain to control. with Dunn's Kruskal–Wallis test. Scale bars as indicated

Functional studies imply that vascular risk factors would increase the expression of *FMNL2* in humans with AD. To test this hypothesis, we performed immunohistochemical staining in post-mortem human brains for FMNL2 and GFAP (Fig. 6) (control, AD, Primary age-related Tauopathy [PART] with or without cardiovascular pathology, Tables S12 and S13). We observed that in control brains, FMNL2 expression was punctate and scarce around the blood vessels (Fig. 6a, b), while in early onset AD and in AD with severe atherosclerosis, there was significant FMNL2 expression that delineated the blood vessels (Fig. 6c–f). In these brains, astroglia were reactive and in some regions glial endfeet were detached from the vessel. In patients with primary age-related tauopathy, FMNL2 localizes around the blood vessels albeit less prominent than in AD (Fig. 6g, h). The tight associations of astroglia to the blood vessels inversely correlate with FMNL2 expression (Fig. 6i). Quantification of FMNL2 expression in control and patients showed a significant increase in FMNL2 in astroglial cells in disease conditions (Fig. 6j), supporting the findings that FMNL2 is a response to AD and is involved in regulating the gliovascular interactions.

## Discussion

A major effort to identify genes and loci associated with AD risk has been underway for several decades [41, 54]. Many of these genes have been replicated and confirmed in other studies [15, 53]. The focus has now shifted to fully understand mechanisms by which variants in genes lead to disease. Epidemiological and neuropathological research has established an intertwined relationship between AD and cerebrovascular disease and its antecedents. Yet, our understanding of the course of this inter-dependent relationship is unclear. To probe the interaction between CVRFs, cerebrovascular disease and AD based on the genotype–phenotype relationship, we used data from five cohorts of different ancestries to perform an unbiased genome-wide association study of the interaction effects of CVRFs and AD genetic variants. We found that *FMNL2*, formin-like protein 2, significantly interacted with the CVRF score to modify AD risk. We also found that the association between the CVRF scores and AD differed by SNP genotypes within *FMNL2.* FMNL2 brain expression was increased in pathological AD, with Aβ and tau deposition and with brain infarcts independently. The mediation analysis allowed us to hypothesize that the effects of FMNL2 expression on pathological AD were related to accumulation of Aβ and tau deposition. Furthermore, we found that FMNL2 to be highly expressed in astrocytes, a key cell type of interest in AD. SNPs in *FMNL2* interacting with CVRF are also correlated with gene expression and hypo-methylation levels suggesting a regulatory effect of common polymorphisms leading to increased FMNL2 expression.

A strength of the current study is the inclusion of three ethnic groups, which is particularly important in the study of genetics and cerebrovascular disease, for two reasons. First, specific variants associated with disease within a gene can differ between ethnic groups based on linkage disequilibrium (LD) structure. Identifying significant SNPs through a meta-analysis across cohorts and collapsing SNPs within gene for gene-based analyses increases the power of discovery and generalizability of the results. Second, there are differences in the frequency of cerebrovascular disease and associated CVRFs between non-Hispanic Whites, African–Americans, and Caribbean Hispanics. Clinically, these results could have important implications for personalized treatment plans, suggesting more aggressive treatments of cardiovascular and cerebrovascular risk factors in persons with specified genotypes. Third, we bring together data from three species—human, mouse, and zebrafish—and demonstrate a validation-oriented functional genomics pipeline to analyze the biological mechanisms of action for increasing number of AD-related genes [6] and their evolutionary conservation. Finally, we also propose zebrafish as a useful experimental model for amyloid β pathology, an important aspect of AD.

Previous studies of gene-environment interactions in AD have focused on candidate genes, stemming from prior genome-wide studies of AD or cerebrovascular disease (e.g., [19, 95]). Here we focused on a genome-wide, unbiased interaction approach to identify novel genes that interact with CVRFs in AD. Hypertension [29, 87], type II diabetes [89] and obesity [5] have each been associated with AD, cerebrovascular disease and alterations in the blood–brain-barrier integrity. The resulting microvascular dysfunction increases the permeability of the blood–brain barrier, oxidative stress, activation of immune mechanisms and leakage of proteins. Additionally, vascular defects generate hypoxic conditions that enhance the toxic protein aggregation [73, 80]. Therefore, these changes increase the demand for waste removal mechanisms in the brain.

### How do variants in FMNL2 contribute to the cerebrovascular pathology in AD?

*FMNL2* encodes a formin-related protein. Formins are important in regulating actin and microtubules, with cellular effects and consequences in cell morphology, cytoskeletal organization and cell–cell contact [30] and are implicated in AD pathology [35, 68]. Based on our results here, we propose that *FMNL2* regulates cell–cell interactions between glia and vasculature, and the clearance of extracellular aggregates. Astroglial endfeet form a perivascular space that is involved in arterial pulsation-dependent clearance [10]. The size of the astroglia-regulated interstitial space correlates with the drainage efficiency of the brain, for instance sleep-dependent adrenergic signaling expands the glympathic space [76, 98]. However, with a higher load of extracellular protein aggregation, more efficient mechanisms such as the clearance through blood–brain barrier [84], where major amyloid-binding efflux receptor complexes including CLU, LRP1/2 and VLDLR are present [97], is required. *FMNL2*-mediated gliovascular remodeling could be a mechanism to potentiate the clearance through blood–brain barrier. Notwithstanding the elevated protein aggregation, clearance mechanisms become less efficient with aging and the efflux receptor expression increases [67]. *FMNL2*-mediated enlargement of perivascular space can serve to facilitate both glympathic and blood–brain barrier clearance mechanisms. Recent findings confirm our hypothesis that perivascular remodeling is a critical response to amyloid pathology [21, 46] and could act as the interface between CVRFs and AD. A recent single cell transcriptomics analyses also confirmed our findings that FMNL2 is upregulated in astroglial cell populations in human AD brains [37]. Additionally, detached gliovascular interactions can lead to immune cell infiltration to the brain from the blood and may help the initial attempts of amyloid clearance [18, 36], which is supported by our findings that *fmnl2* knock-down reduces the microglia that contact the blood vessels in zebrafish.

The long-term consequence of gliovascular detachment is unclear [47, 82], as breached blood–brain-barrier could inadvertently lead to exacerbation of the disease pathology. Additionally, whether the gliovascular endfeet retraction is a consequence of a generic gliotic response or is part of a disease-associated mechanism is debated. Our results showed that amyloid aggregation but not the traumatic injury induced FMNL2 expression, suggesting that FMNL2-regulated astroglial endfeet remodeling could be preferentially related to AD pathophysiology. A recent study demonstrating that selective focal ablation of astroglia endfeet around the blood vessels does not cause blood–brain-barrier disruption and gliovascular contacts are efficiently replaced [62] supports our findings.

In this study, we experimentally showed that (1) FMNL2 is expressed in astroglial cells, (2) FMNL2 is upregulated with amyloid deposition in zebrafish, mouse, and human brains, (3) gliovascular interactions loosen with the disease pathology in three species, (4) this response requires FMNL2, and (5) loss of FMNL2 function leads to amyloid deposition and reduced immune response. Post-mortem human brain immunohistochemical staining also confirmed that compared to control individuals, patients with AD have a significant increase in the expression of FMNL2 near the glial endfeet at the blood vessels and in astrocytes that appear hypertrophic and reactive. We also observed that glial end feet were arrayed around the blood vessels in controls while this arrayed structure is impaired and show a dilated appearance concomitant to prominent FMNL2 expression. Based on these results, we propose that *FMNL2* functions in astroglial cells to regulate the extent of gliovascular interactions and toxic protein clearance from the brain on demand. The role of FMNL2 may also be required in other proteinopathies such as progressive age-related tauopathy, and FMNL2 is also upregulated and delineated in blood vessel structures.

Gliovascular contacts are important components of the blood–brain barrier, and they partake in sustained homeostasis and clearance of amyloid β [99, 102]. The role of *FMNL2* in amyloid clearance is supported by the findings where the knock-down of *FMNL2* protein resulted in the defective sorting into late endosomes and lysosomes [43]. Abnormalities in the endosomal-lysosomal network are known to be important to the mechanism of neurodegeneration and AD [63]. We do not exclude that FMNL2 could function in other cell types such as pericytes around the blood vessels to regulate the clearance and drainage mechanisms as well as to control the immune response either by altering microglia–blood vessel interactions or extravasation of immune cells into the brain. Future studies aiming at elucidating disease stage and cell-specific roles of FMNL2 will be instrumental to further delineate the role of this protein and to propose new drug candidates.

The animal models that we used in this study—zebrafish and mouse—reflect the acute and chronic pathological changes and cellular response to amyloidosis. We recognize that the complex late-stage cognitive decline in AD may not be fully represented in these model systems. However, the post-mortem human tissue analyses, AD cohort studies and single cell datasets are relevant to the cognitive decline in AD through clinical (e.g., cognitive assessment scores) and neuropathological (e.g., Braak staging and histological analyses) aspects. Our hypothesis favors for an FMNL2-related pathological mechanism that is likely to initiate during the early course of the disease and extend toward the later stages. In all our investigations, the dynamics of FMNL2 was consistent either in acute zebrafish model, chronic mouse model or post-mortem human brains with documented clinical and pathological AD. This suggests that the biological role of FMNL2 and the cellular interactions between neuro-glio-vascular niche cells could be an early response to the disease and extends toward later stages even when cognitive decline manifests. The relationship of clearance to the cognitive decline and brain atrophy is yet to be investigated.

FMNL2 is most highly expressed in the brain. In a study of ischemic stroke in young individuals (*n* = 1816), one SNP was found to be associated (rs2304556 *p* = 1.18 × 10^–7^) [23]. Interestingly, we found that increased FMNL2 expression (ENSG00000157827) was associated with brain infarcts and AD independently, suggesting that this gene may be involved in a shared molecular mechanism with cerebrovascular disease. The SNP with the highest significant association in our study, rs57223657, is a point mutation in the third intron of *FMNL2* gene, where a promoter regulatory flank region resides. Intronic regions that contain gene regulatory, or enhancer functions are known to be critical determinants of gene expression and function [71, 72]. Thus, it is possible that regulatory variants of *FMNL2* may also alter gene function and the extent of the AD pathology at least through the regulation of gliovascular interactions. The multidisciplinary work reported here demonstrates the unique involvement of genes, *FMNL2*, and antecedent CVRFs in AD with human GWAS and in vivo animal model data.

While this study has several strengths, we recognize that there are also some limitations. First, we relied on self-report of three of the four vascular risk factors. While this approach is reliable and valid [45] it is also possible some individuals responded incorrectly. However, we did measure the fourth, BMI, directly. Second, we did not consider the possible effects of medications used to treat vascular risk factors, such as hypertension or diabetes. We were unable to define more deeply some of the cardiovascular diseases. In some groups, investigators only reported “any heart disease”, while others included information on the specific heart condition. We used a transient knock-down of *FMNL2* function in an acute amyloidosis zebrafish model, and a model of chronic amyloid pathology in mouse. A gene editing approach coupled to chronic models to mimic human *FMNL2* SNP variants will help to capture long-term effects of *FMNL2* on vascular risk factors and AD pathology in the zebrafish or mouse experimental models.

Taken together, this investigation implies that hypertension, diabetes, heart disease and obesity are more likely to have acquired cerebrovascular disease in the brain and are at higher risk for AD. In these patients, FMNL2 expression resulting from the vascular risk factors and the resulting cerebrovascular pathology regulate the ongoing deposition of amyloid and tau proteins by altering the normal interplay between glia and the vasculature and ultimately the clearance of extracellular aggregates (Supplementary Fig. 9). Our results also indicate that *FMNL2* expression is upregulated in the presence of CVRFs among individuals who develop AD, and that having both conditions augment the effect of FMNL2 on AD pathology. The sequence of events—whether CVRF or AD comes first—will be better addressed with longitudinal experimental models for both diseases, yet our mediation analyses and functional experiments suggest that CVRF potentiates the AD pathology. Expanding on the relationship of genes and loci identified here, along with expanded basic molecular work will move us closer to conceptualizing the etiological interaction between AD and cerebrovascular disease and hopefully identifying therapeutic targets for AD.

## Supplementary Information

Below is the link to the electronic supplementary material.

Supplementary file1 (PDF 4859 KB)

Supplementary file2 (XLSX 566 KB)

## Data Availability

Genetic data for all cohorts is available at the National Institute on Aging Genetics of Alzheimer’s Disease Data Storage Site (NIAGADS; https://www.niagads.org). Primary clinical data for WHICAP and EFIGA data are available at https://www.neurology.columbia.edu/research/research-programs-and-partners/alzheimers-disease-research-center-adrc/investigators/investigator-resources. Clinical data from NACC data is accessible on request at https://naccdata.org. Clinical data from ROSMAP data is accessible on request at https://www.radc.rush.edu. For the RNA sequencing data, gene-level transcript values in counts and normalized expression data from ROSMAP are available on the AMP-AD Knowledge Portal at Synapse (https://adknowledgeportal.synapse.org) (Synapse: syn25741873). The residual expressions from gene transcripts are available at Synapse (ROSMAP: syn25741873, MSBB: syn8485027, and Mayo: syn8466826). The ROSMAP single nucleus RNA sequencing data and the description of the data are available at Synapse (Synapse ID: syn16780177). Expression datasets for zebrafish are publicly available as NCBI GEO accessions GSE74326 and GSE161834.

## Code availability

We used publicly available statistical software to perform analyses in the Methods section. Code to perform analyses in this manuscript are available from the author upon request (A.J.L.).
