# Supplementary material for: FMNL2 regulates gliovascular interactions and is associated with vascular risk factors and cerebrovascular pathology in Alzheimer’s disease
Source: Acta Neuropathol. 2022 May 24;144(1):59–79. doi: 10.1007/s00401-022-02431-6 (PMC9217776; doi:10.1007/s00401-022-02431-6)
Supplement: Supplementary file 1 — Supplementary file1 (PDF 4859 KB) [file 401_2022_2431_MOESM1_ESM.pdf]

FMNL2 regulates gliovascular interactions and is associated with vascular risk factors and cerebrovascular pathology in Alzheimer's disease

Annie J. Lee, Neha S. Raghavan, Prabesh Bhattarai[a], Tohid Siddiqui, Sanjeev Sariya, Dolly Reyes-Dumeyer, Xena E. Flowers, Sarah Anne Laurence Cardoso, Philip L. De Jager, David A. Bennett, Julie A. Schneider, Vilas Menon, Yanling Wang, Rafael A. Lantigua, Martin Medrano, Diones Rivera, Ivonne Z. Jiménez-Velázquez, Walter A. Kukull, Adam M. Brickman, Jennifer J Manly, Giuseppe Tosto, Caghan Kizil, Badri N. Vardarajan, Richard Mayeux

Supplementary Information

Supplementary Fig. 1 Study design and analysis workflow.

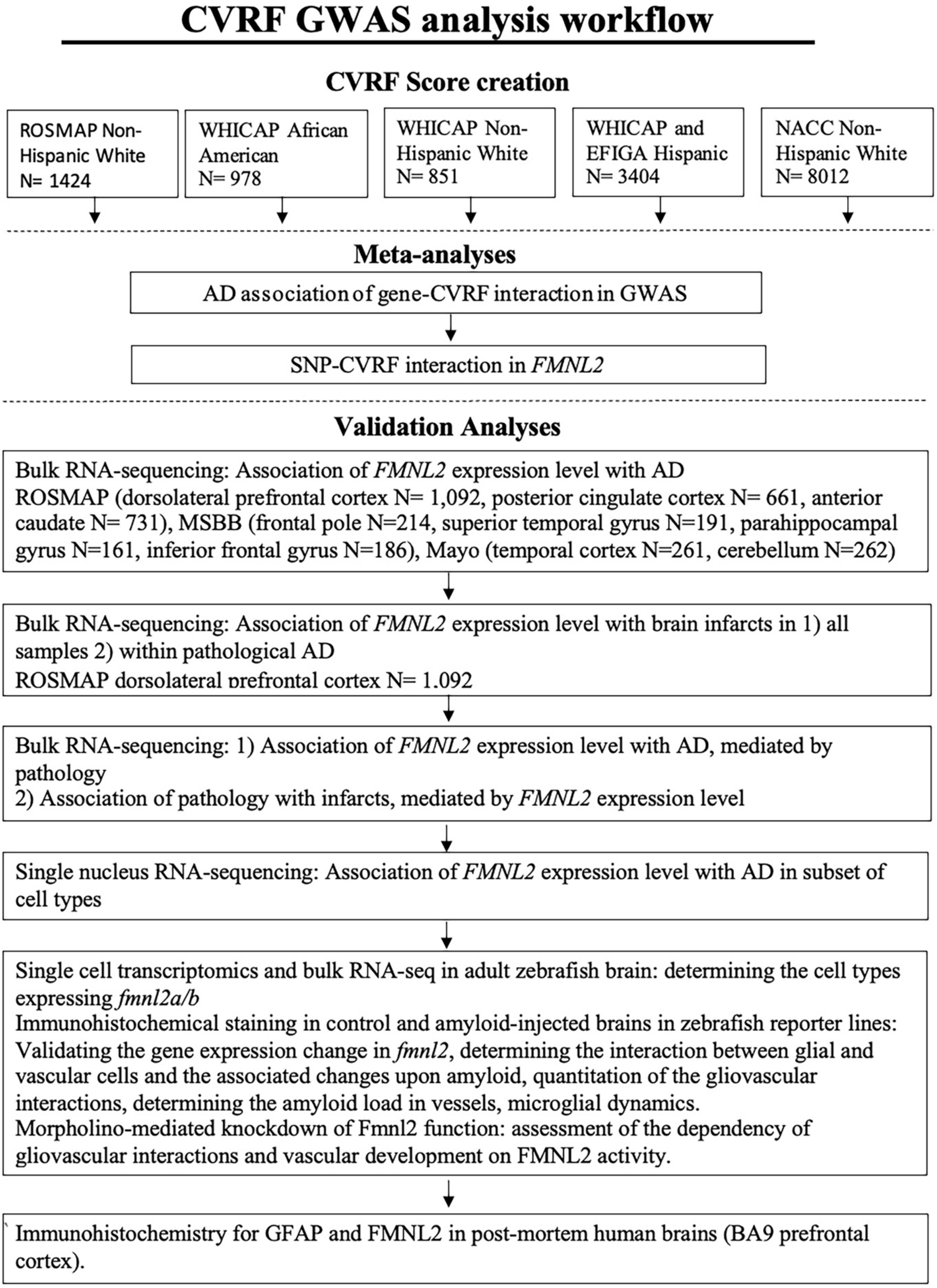

Supplementary Fig. 2 Correlation matrix of a history of heart disease, hypertension, diabetes, and BMI at last visit in each cohort.

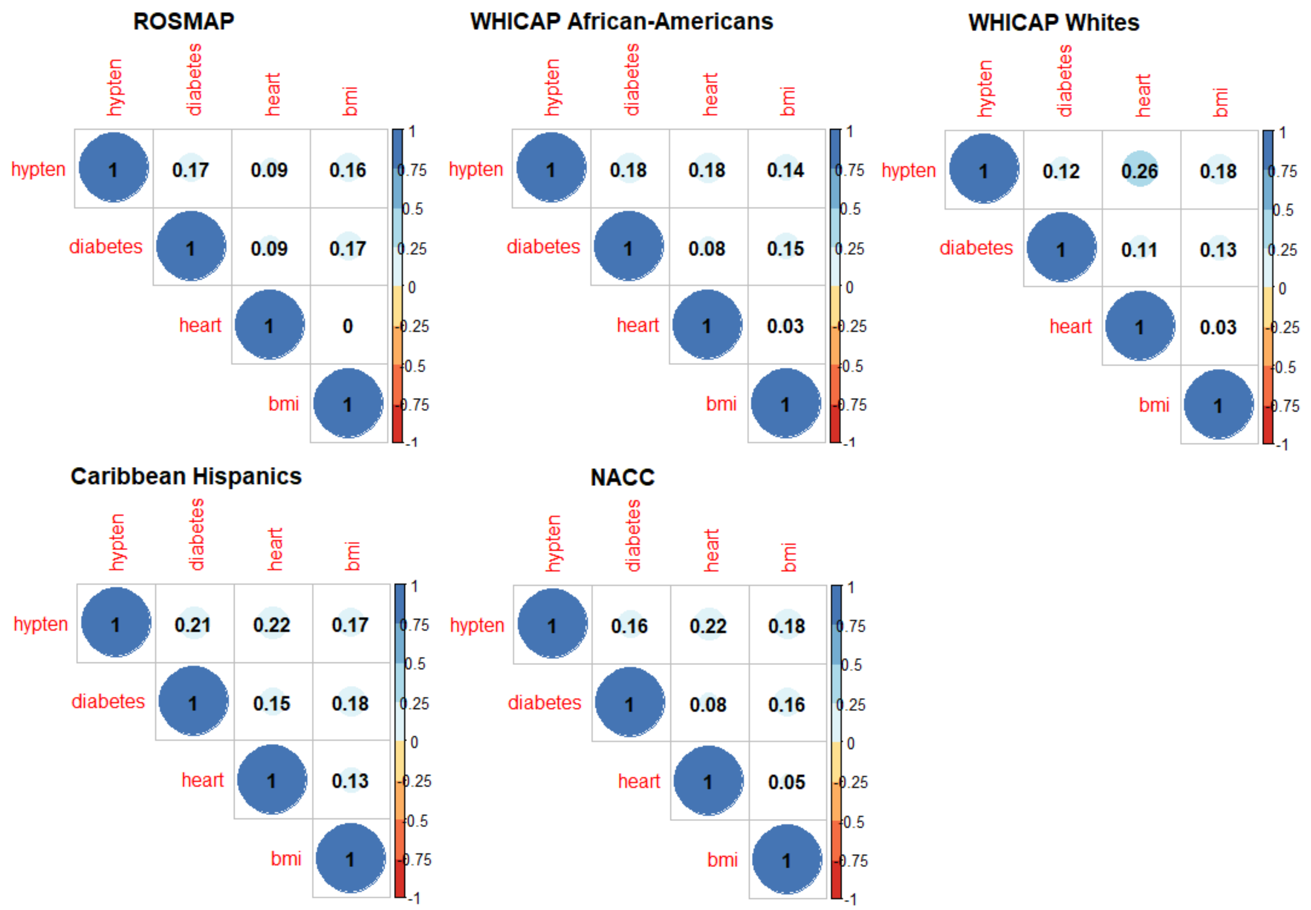

Supplementary Fig. 3 Principal component plots for history of heart disease, hypertension, diabetes, and BMI at last visit in each cohort.

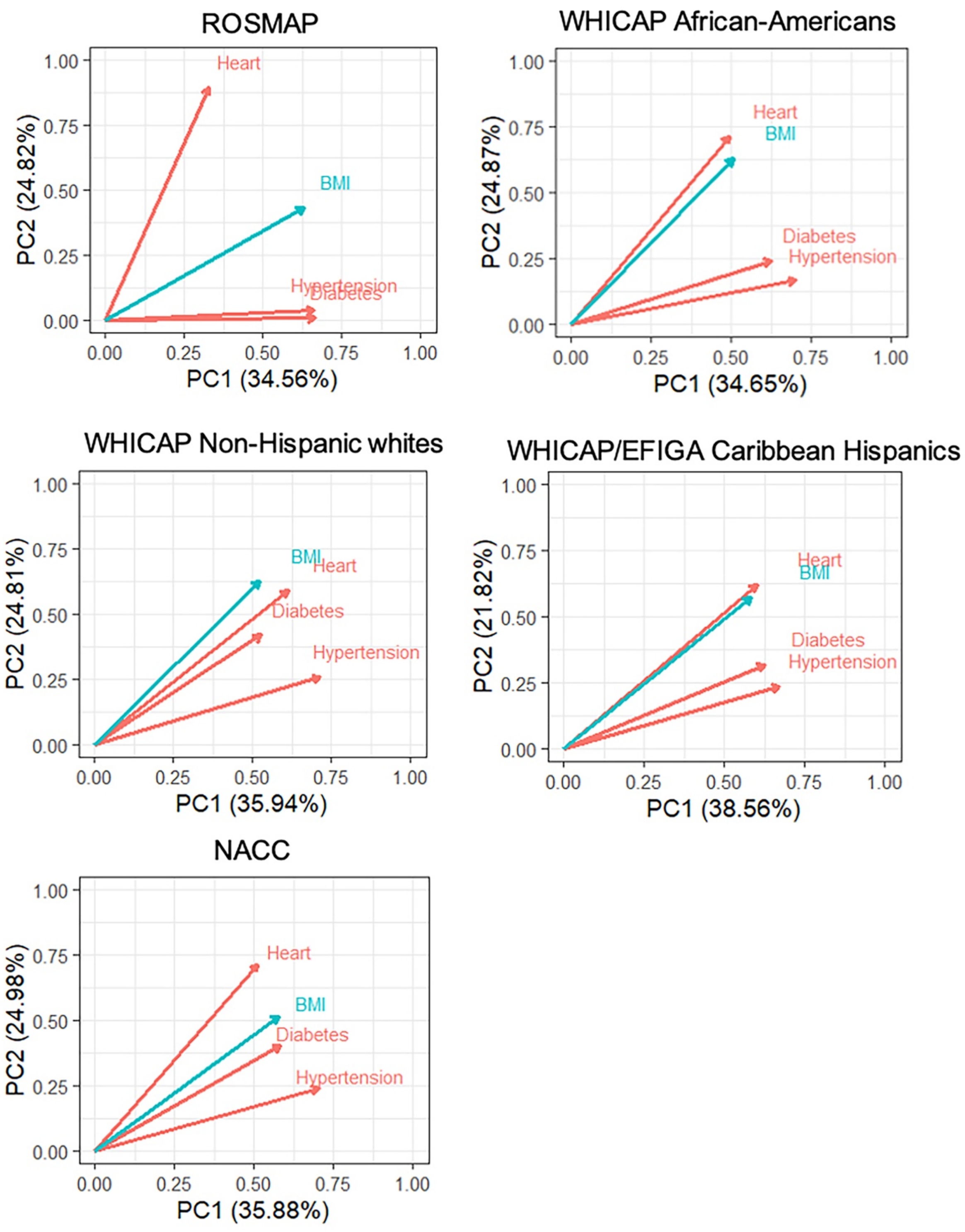

Supplementary Fig. 4 QQ plot of gene-CVRF interaction test in GWAS for each cohort.

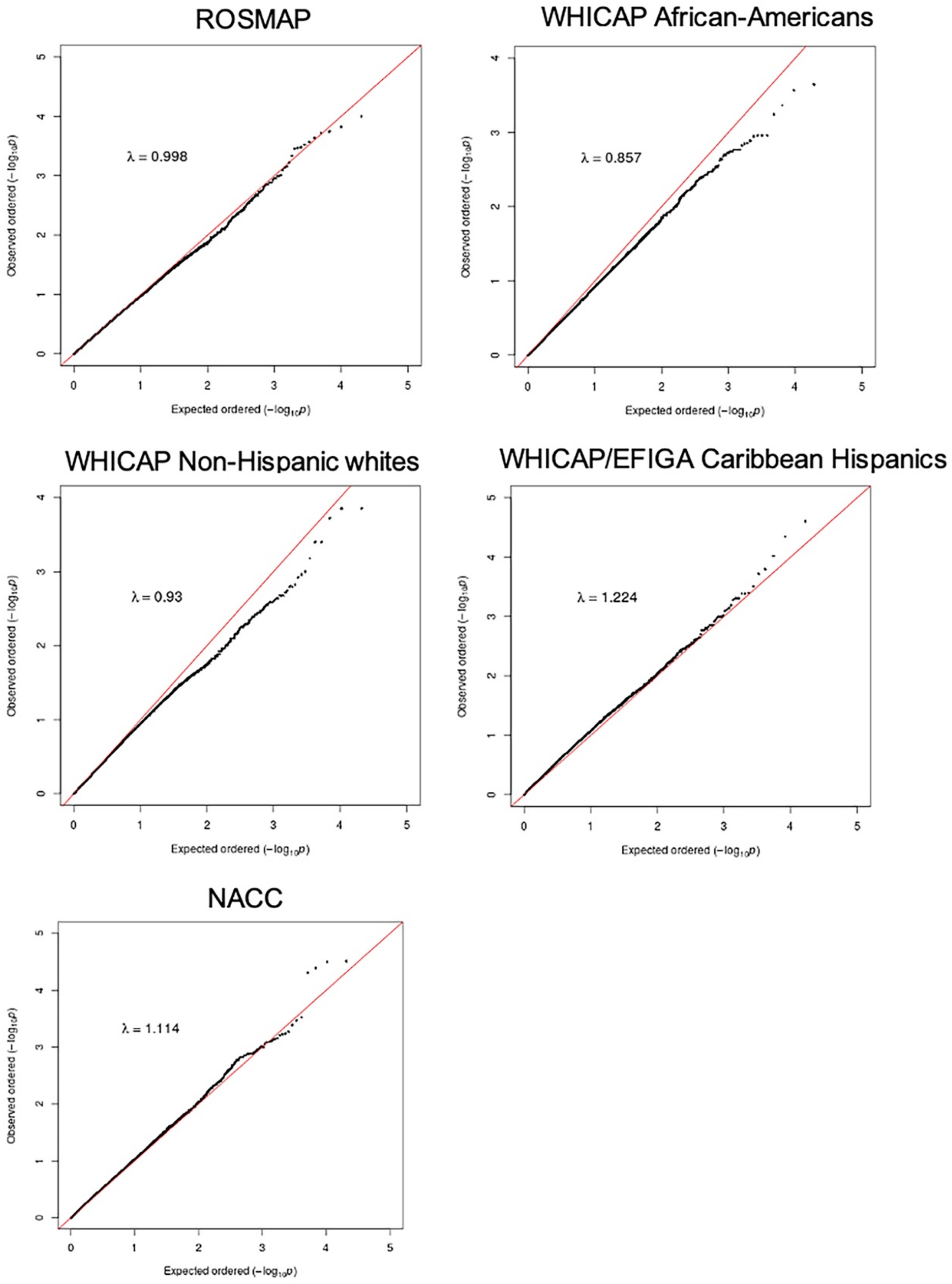

Supplementary Fig. 5 Transmission electron microscope images from control **(a)** and amyloid-injected **(b)** zebrafish brains. One neuron is shown. Nucleus is named, red asterisk denotes the amyloid aggregate in the membrane-bound compartment. Lower panel is a high magnification showing the beta-pleated striated aggregates. Scale bars as indicated.

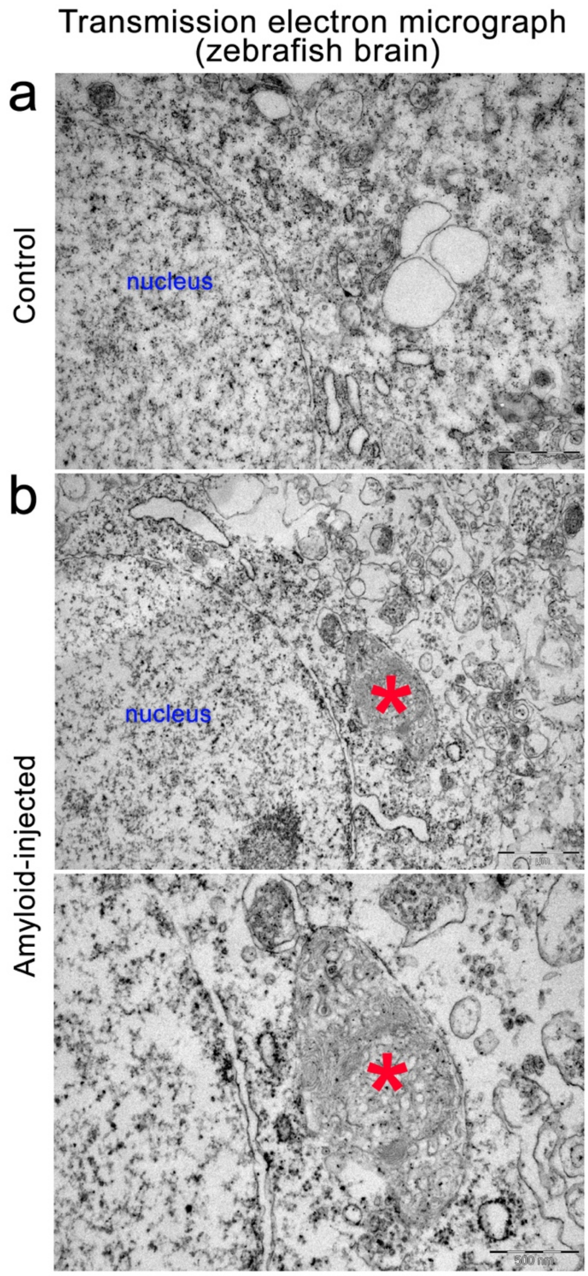

Supplementary Fig. 6 Fmnl2 antibody specificity, knockdown efficiency of *fmnl2a/b* morpholinos and *fmnl2a/b* in blood vessel development. **(a)** Single cell sequencing tSNE plots for *fmnl2b* and *sv2a (neuronal marker)* in adult zebrafish brain. *fmnl2b* is expressed in neurons. **(b)** Antibody specificity test for Fmnl2 antibody used in our study. Zebrafish brain sections were subjected to immunohistochemical staining without (upper panel) and with (lower panel) Fmnl2 primary antibody. In both samples, secondary antibody is used. Fmnl2 antibody generates a signal that is specific to the primary antibody. **(c)** dIHC for FMNL2 and her4.1-GFP counterstained with DAPI. Fmnl2 protein can be detected in astroglia and in neurons, and these expression patterns are discernible. **(d)** Efficiency of *fmnl2a/b* knockdown. Fmnl2 IHCs for 2-day old zebrafish embryos that are injected with control morpholino, and *fmnl2a/b* morpholinos with DAPI counterstain. Two Fmnl2 antibodies are used to test the reliability and specificity. Left: merge image. Middle: Fmnl2 staining with rabbit primary antibody. Right: Fmnl2 staining with mouse primary antibody. Both antibodies detect the floor plate expression of FMNL2, which is reduced upon injection of fmnl2 morpholinos. **(e)** Fmnl2 is not involved in blood vessel development. ZO-1 IHCs for 2-day old zebrafish embryos that are uninjected (left), injected with control morpholino (middle) or *fmnl2a/b* morpholino (right) at 1-cell stage. **(f)** Quantification of the relative length of intersegmental blood vessels in e. **(g)** Phalloidin-488 staining on 1 dpf zebrafish embryo in uninjected, control morpholino-injected and *fmnl2a/b* morpholino-injected animals. Scale bars as indicated.

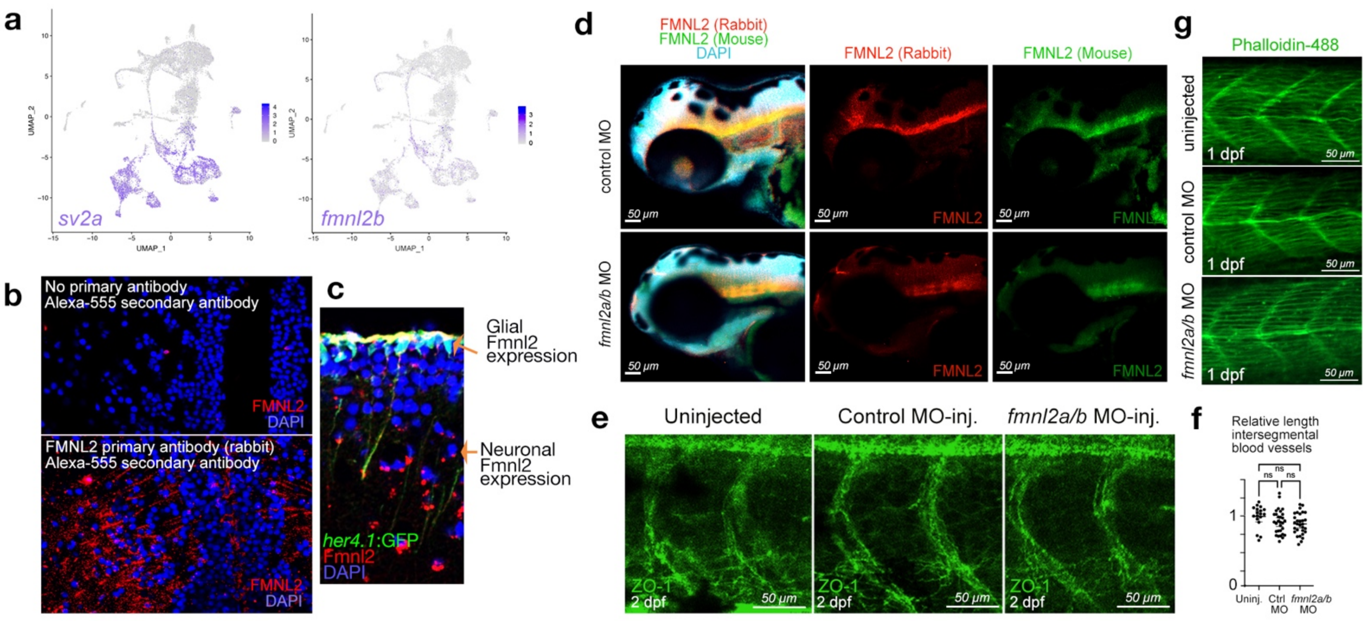

Supplementary Fig. 7 Antibody specificity test for Fmnl2. Immunohistochemical staining for Fmnl2 with DAPI counterstain on mouse cerebral cortex sections from APP/PS1dE9 mouse. **(a)** staining without the primary Fmnl2 antibody but with secondary antibody. Individual scarce spots are background staining**. (b, c)** Two separate mouse brain sections stained with primary and secondary antibodies show Fmnl2 staining. Scale bars as indicated.

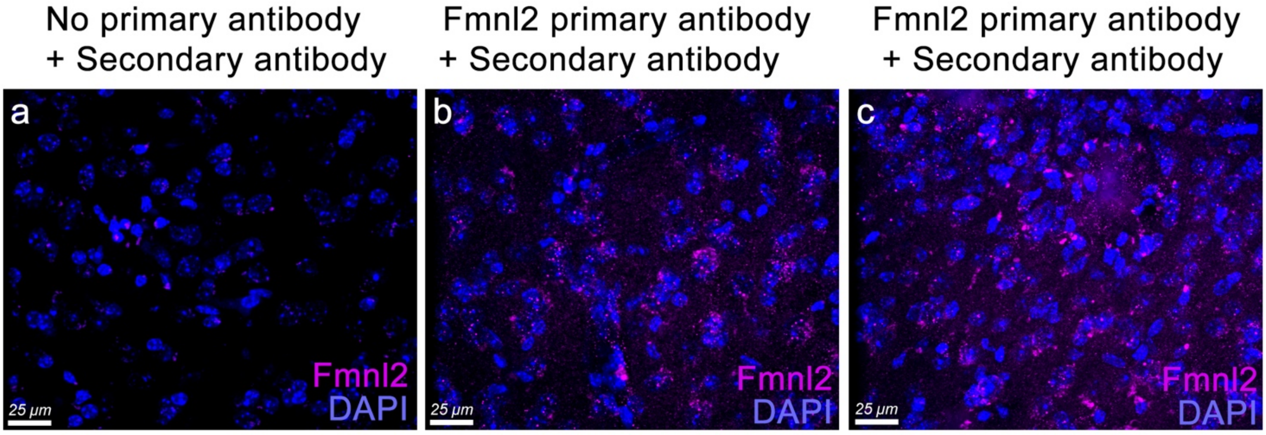

Supplementary Fig. 8 Injury in mouse brain does not lead to upregulation of Fmnl2 or gliovascular end feet retraction. **(a)** Gfap immunostaining on the uninjured hemisphere of the cerebral cortex of the mouse brain. **(b)** triple immunohistochemical staining (tIHC) for Gfap (astroglia), Cd31 (blood vessel) and Fmnl2 on control hemisphere. Individual fluorescent channels of b for Gfap **(c)**, Cd31 **(d)** and Fmnl2 **(e)**. **(f)** Gfap immunostaining on the injured hemisphere of the cerebral cortex of the same mouse brain in b. **(g)** tIHC for Gfap, Cd31 and Fmnl2 on injured hemisphere. Individual fluorescent channels of g for Gfap **(h)**, Cd31 **(i)** and Fmnl2 **(j)**. Scale bars as indicated.

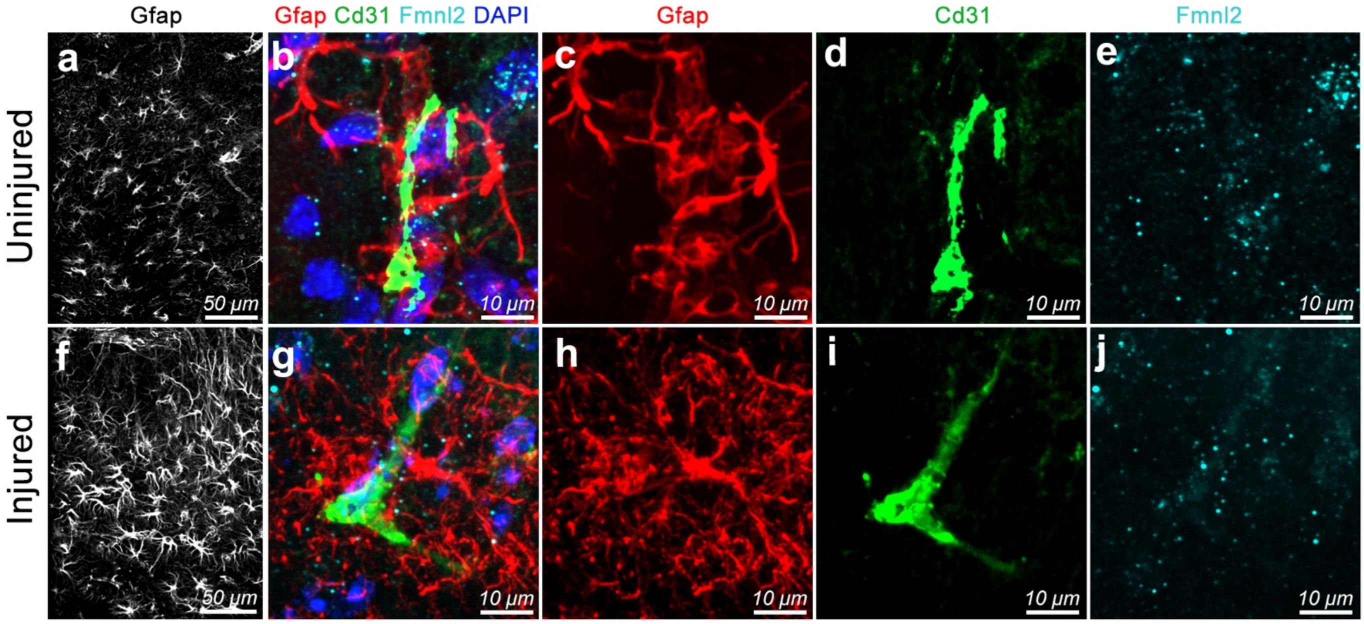

Supplementary Fig. 9 Working hypothesis on FMNL2 function.

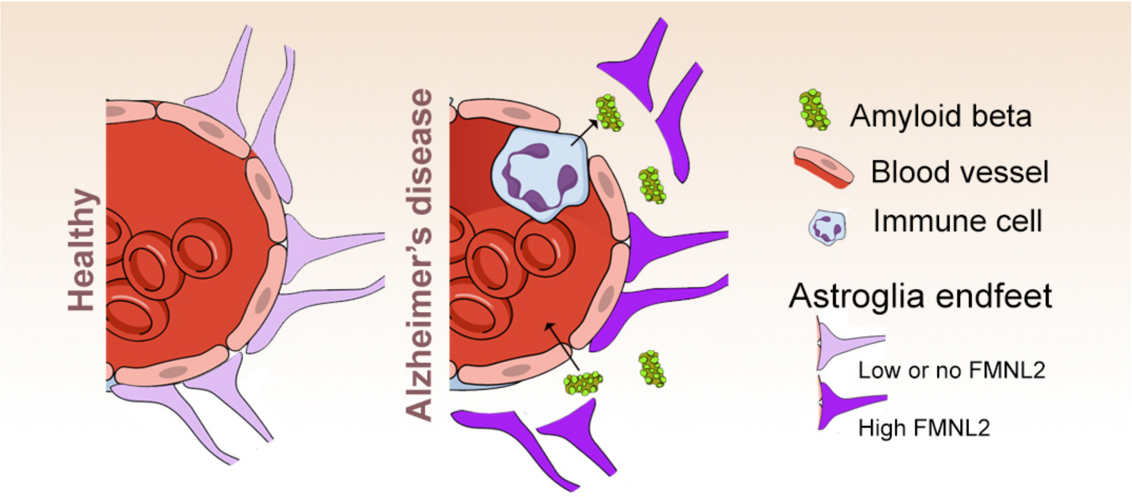

Table S1. Variance explained by the first four principal components and contributions of the risk factors to each component.

Cohort	PC	Eigenvalue	Proportion of variance	Cumulative proportion of variance	Squared loadings			
					Hypertension	Diabetes	Heart	BMI
ROSMAP	1	1.38	34.56	34.56	0.438	0.441	0.107	0.397
	2	0.99	24.82	59.37	0.001	6.35×10^{-5}	0.804	0.188
	3	0.83	20.81	80.18	0.427	0.404	4.03×10^{-4}	0.001
	4	0.79	19.82	100	0.134	0.155	0.09	0.414
WHICAP African-Americans	1	1.39	34.65	34.65	0.495	0.391	0.244	0.256
	2	0.99	24.87	59.52	0.028	0.059	0.511	0.397
	3	0.84	21.09	80.61	0.004	0.485	0.054	0.301
	4	0.78	19.39	100	0.473	0.065	0.191	0.047
WHICAP Whites	1	1.44	35.94	35.94	0.511	0.279	0.376	0.271
	2	0.99	24.81	60.76	0.067	0.177	0.353	0.395
	3	0.87	21.86	82.62	0.101	0.525	0.022	0.226
	4	0.7	17.38	100	0.321	0.018	0.248	0.108
Caribbean Hispanics	1	1.54	38.56	38.56	0.451	0.391	0.361	0.339
	2	0.87	21.82	60.37	0.056	0.101	0.386	0.329
	3	0.82	20.59	80.96	0.039	0.368	0.087	0.33
	4	0.76	19.04	100	0.453	0.14	0.166	0.002
NACC	1	1.44	35.88	35.88	0.497	0.341	0.263	0.334
	2	1	24.98	60.86	0.058	0.164	0.51	0.268
	3	0.83	20.68	81.54	0.049	0.495	0.009	0.275
	4	0.74	18.46	100	0.396	3.36E-06	0.218	0.124

Table S2. Top Gene x CVRF interactions (p<0.05) associated with LOAD, related to Table 2 and Supplementary Figure 4.

		ROSMAP	WHICAP African-Americans	WHICAP Whites	Caribbean Hispanics	NACC	Meta-analysis			
**Gene**	**Chr**	**p**	**p**	**p**	**p**	**p**	**n**	**p**	**FDR**	**Direction**
FMNL2	2	0.00110	0.18881	0.03796	0.00890	0.01199	14669	6.59E-07	0.012	.....
LOC401357	7	0.17183	0.36164	0.01998	0.00080	0.00599	14669	1.13E-06	0.012	.....
AMMECR1L	2	0.94006	0.07293	0.04396	0.00460	0.00130	14669	2.43E-06	0.017	.....
CFAP99	4	0.54945	0.93706	0.42358	0.00041	0.00220	14669	1.27E-05	0.068	.....
SLC22A14	3	0.29371	0.42458	0.30669	0.29271	0.00005	14669	1.81E-05	0.070	.....
PRG3	11	0.38661	0.08991	0.01199	0.20480	0.00150	14669	1.96E-05	0.070	.....
PTPRF	1	0.05594	0.56344	0.53247	0.65934	0.00003	14669	2.84E-05	0.087	.....
PLA2G4E	15	0.25375	0.89311	0.21878	0.50949	0.00003	14669	4.55E-05	0.095	.....
ACACB	12	NA	0.18382	0.13487	0.03796	0.00360	13245	4.98E-05	0.095	?....
LINC00353	13	0.00400	0.26973	0.26673	0.10589	0.01410	14669	5.29E-05	0.095	.....
IGFN1	1	0.42757	0.63137	0.40759	0.00590	0.00390	14669	5.58E-05	0.095	.....
LOC100129345	14	0.08791	0.78921	0.09191	0.01410	0.01399	14669	6.19E-05	0.095	.....
TPR	1	NA	0.12987	0.57243	0.60839	0.00004	13245	6.21E-05	0.095	?....
LANCL2	7	0.13986	0.30070	0.15584	NA	0.00100	11265	6.47E-05	0.095	...?.
HOXB4	17	0.09890	0.02697	0.33367	0.21479	0.00500	14669	6.60E-05	0.095	.....
EP400	12	0.83716	0.08691	0.05594	NA	0.00080	11265	8.48E-05	0.106	...?.
FAM195B	17	0.35564	NA	0.00699	0.06094	0.00899	13691	9.49E-05	0.106	.?...
RFC2	7	0.05594	NA	0.08192	0.06893	0.01099	13691	9.52E-05	0.106	.?...
HOXB3	17	0.00899	0.23377	0.30370	0.07892	0.02298	14669	9.78E-05	0.106	.....
SPPL2C	17	0.11888	0.07692	0.96903	0.00410	0.03796	14669	0.00011	0.106	.....
CD83	6	0.07393	0.21179	0.35165	NA	0.00200	11265	0.000111	0.106	...?.
POLR2D	2	0.98002	0.32667	0.76923	0.00390	0.00380	14669	0.000113	0.106	.....
FLJ31104	5	0.41059	0.62937	0.04096	NA	0.00070	11265	0.000116	0.106	...?.
CDK8	13	0.51748	0.07493	0.64336	0.14785	0.00130	14669	0.00012	0.106	.....
NNMT	11	0.35265	0.00730	0.07393	0.23576	0.01230	14669	0.000126	0.106	.....
FABP2	4	0.03497	0.16883	0.22877	0.07592	0.02398	14669	0.000131	0.106	.....
RAMP3	7	0.55744	0.12987	0.12088	0.03896	0.01099	14669	0.000133	0.106	.....
ARHGAP40	20	0.46254	0.13886	0.17283	0.34665	0.00112	14669	0.000144	0.110	.....
PABPC4L	4	0.03397	0.31968	0.91908	0.08392	0.00630	14669	0.000149	0.110	.....
DLK2	6	0.63337	0.02298	0.56244	0.01598	0.01798	14669	0.000154	0.110	.....
DAP3	1	0.14785	0.07493	0.97103	0.00004	0.23477	14669	0.000168	0.110	.....
FAM193A	4	0.13387	0.50849	0.40160	0.09491	0.00430	14669	0.000174	0.110	.....
CLEC6A	12	0.01598	0.59940	0.01099	0.77223	0.00420	14669	0.000174	0.110	.....
CALM3	19	0.24176	0.48052	0.08392	0.14785	0.00480	14669	0.000181	0.110	.....
WBP2NL	22	0.14785	0.00022	0.54945	0.21978	0.02997	14669	0.000182	0.110	.....
SRRD	22	0.21578	0.13586	0.32368	0.61139	0.00082	14669	0.000195	0.110	.....
C2orf27A	2	0.97602	0.28272	0.36963	0.06893	0.00150	14669	0.000195	0.110	.....
LINC01291	2	0.10889	0.44655	0.40759	0.03696	0.01399	14669	0.000202	0.110	.....
LRSAM1	9	0.00720	0.89111	0.76124	NA	0.00180	11265	0.000206	0.110	...?.
RALGPS1	9	0.54146	0.23676	0.71229	NA	0.00034	11265	0.000226	0.110	...?.
ARHGDIA	17	0.60739	NA	0.11089	NA	0.00060	10287	0.000235	0.110	.?.?.
SELENBP1	1	0.25674	0.67532	0.14286	0.10490	0.00490	14669	0.000238	0.110	.....
KATNBL1P6	6	NA	0.69830	0.02298	0.64835	0.00041	13245	0.000251	0.110	?....
C10orf82	10	0.04296	0.77323	0.14685	0.10989	0.01299	14669	0.000252	0.110	.....
MAPRE2	18	0.14186	0.59540	0.56843	0.38362	0.00072	14669	0.000261	0.110	.....
RAB3A	19	0.09191	0.03896	0.42957	0.11289	0.02697	14669	0.000266	0.110	.....
KCNAB1	3	0.00172	0.24476	0.97702	0.01099	0.12388	14669	0.000266	0.110	.....
MYCT1	6	NA	0.28971	0.01499	0.64136	0.00136	13245	0.000282	0.110	?....
S100A1	1	0.03896	NA	0.72428	0.62338	0.00058	13691	0.000283	0.110	.?...
SNX24	5	0.33467	0.33866	0.47053	NA	0.00090	11265	0.000291	0.110	...?.
TSPO	22	0.22877	0.20779	0.66134	0.30270	0.00170	14669	0.000293	0.110	.....
TMEM41B	11	0.29970	0.36464	0.31269	NA	0.00133	11265	0.000295	0.110	...?.
MIR9-3HG	15	0.54246	0.11289	0.39361	0.00940	0.03497	14669	0.000301	0.110	.....
TOX4	14	0.12388	0.85714	0.70929	0.00620	0.02398	14669	0.000315	0.110	.....
LINC01550	14	0.24975	0.30470	0.04396	0.04895	0.03696	14669	0.000319	0.110	.....
TXNDC8	9	0.04296	0.30470	0.67532	0.00009	0.33467	14669	0.000331	0.110	.....
ZNF876P	4	0.98402	0.11489	0.38462	0.00710	0.02398	14669	0.000333	0.110	.....
SCARA3	8	0.50050	0.05694	0.31269	0.17682	0.00710	14669	0.000337	0.110	.....
APOA5	11	0.26074	0.21379	0.92807	0.27273	0.00140	14669	0.00034	0.110	.....
PMP22	17	0.03197	0.24176	0.40859	0.64535	0.00310	14669	0.000347	0.110	.....
LRRC3C	17	0.39560	0.68232	0.27772	0.07992	0.00450	14669	0.00035	0.110	.....
RNF222	17	0.59041	0.15684	0.33866	0.10190	0.00620	14669	0.000351	0.110	.....
TXN	9	0.09491	0.35864	0.79021	0.00002	0.32867	14669	0.000352	0.110	.....
UBXN6	19	0.81119	0.15984	0.36464	0.00730	0.02797	14669	0.000354	0.110	.....
LINC01019	5	0.32767	0.01399	0.03896	0.26174	0.03097	14669	0.000354	0.110	.....

SLC38A6	14	0.40060	0.61039	0.06294	0.21578	0.00390	14669	0.000356	0.110	.....
IL6ST	5	0.12388	0.87712	0.09091	NA	0.00290	11265	0.000358	0.110	...?.
METTL3	14	0.09790	0.31469	0.99401	0.08591	0.00810	14669	0.000369	0.110	.....
LINC01036	1	0.37263	0.04096	NA	0.02198	0.03696	13818	0.000377	0.110	..?..
TTPAL	20	0.12388	0.00460	0.37962	0.01598	0.18881	14669	0.000379	0.110	.....
ZNF766	19	0.14585	0.94206	0.66633	0.01099	0.01798	14669	0.000386	0.110	.....
ASPHD2	22	0.54545	0.26074	0.09091	0.63736	0.00100	14669	0.000393	0.110	.....
C1orf21	1	0.32368	0.08691	0.29071	0.01698	0.05994	14669	0.000394	0.110	.....
MYO15B	17	0.17283	0.16184	0.00380	0.27972	0.03796	14669	0.000404	0.110	.....
KDM4A	1	0.03796	0.94306	0.28472	0.62238	0.00130	14669	0.000405	0.110	.....
BLACAT1	1	0.16683	0.06194	0.24875	0.09990	0.03596	14669	0.000411	0.110	.....
KCTD16	5	0.00799	0.67133	0.40160	0.01698	0.09291	14669	0.000416	0.110	.....
PHOX2B	4	0.31469	0.79920	0.43956	0.19980	0.00150	14669	0.000417	0.110	.....
MGAT4C	12	0.52148	0.07992	0.56044	NA	0.00190	11265	0.000427	0.110	...?.
C11orf40	11	0.01030	0.59640	0.08591	0.11588	0.05594	14669	0.000432	0.110	.....
SLC13A2	17	0.33067	0.53147	0.52747	0.07293	0.00580	14669	0.000432	0.110	.....
GPR37L1	1	0.48352	0.23477	0.11688	0.58442	0.00150	14669	0.000444	0.110	.....
SLC2A8	9	0.00410	0.92707	0.91608	NA	0.00390	11265	0.000447	0.110	...?.
ADCY6	12	0.75225	0.37163	0.96803	0.00999	0.00899	14669	0.000448	0.110	.....
PTPN7	1	0.06294	0.64635	0.41958	0.62038	0.00130	14669	0.000452	0.110	.....
KCNJ5	11	0.68931	0.35465	0.13187	NA	0.00150	11265	0.000454	0.110	...?.
FLJ33581	20	0.15684	0.94805	0.36364	0.53646	0.00062	14669	0.000458	0.110	.....
ZNF732	4	0.86114	0.09291	0.58741	0.01099	0.02498	14669	0.000464	0.110	.....
ZNF429	19	0.13187	0.55445	0.48851	0.14585	0.00660	14669	0.00047	0.110	.....
ADGRL3-AS1	4	0.13986	0.28272	0.25974	0.72727	0.00170	14669	0.000472	0.110	.....
TCF21	6	0.02498	0.05495	0.82717	NA	0.01399	11265	0.000474	0.110	...?.
LOC101929231	2	NA	0.19081	0.63137	0.00076	0.09191	13245	0.000474	0.110	?....
TP53BP1	15	0.12987	0.20380	0.38761	0.26274	0.00850	14669	0.000479	0.110	.....
MS4A12	11	0.13187	0.18881	0.28172	0.06593	0.03796	14669	0.000487	0.110	.....
ZNF593	1	0.54545	0.11089	0.02597	0.02697	0.08192	14669	0.000488	0.110	.....
GNPTG	16	0.39960	0.06593	0.46853	0.05395	0.02597	14669	0.000491	0.110	.....
LINC01551	14	0.20180	0.62537	0.37463	0.01299	0.03696	14669	0.000509	0.112	.....
P4HB	17	0.33566	NA	0.09291	0.29770	0.00360	13691	0.000509	0.112	.?...
RBFOX2	22	0.83117	0.04595	0.12887	0.01499	0.06793	14669	0.000523	0.113	.....
CYP26B1	2	0.06494	0.86014	0.62737	0.01199	0.03996	14669	0.000528	0.113	.....
PTPRJ	11	0.43257	0.20979	0.49850	0.03297	0.02098	14669	0.000531	0.113	.....
RAP1GDS1	4	0.00350	0.12787	0.46054	0.68631	0.01598	14669	0.000549	0.115	.....
DUBR	3	0.12388	0.43157	0.14186	0.07193	0.03596	14669	0.000554	0.115	.....
LOC100506730	1	0.61838	0.42258	0.59740	0.00490	0.02997	14669	0.000563	0.115	.....
CDC34	19	0.01698	0.79820	0.02198	0.00310	0.37063	14669	0.000565	0.115	.....
MSS51	10	0.09291	0.28571	0.79421	NA	0.00350	11265	0.000568	0.115	...?.
SERINC3	20	0.20979	0.02098	0.46553	0.00250	0.26374	14669	0.000571	0.115	.....
MATN1	1	0.07393	0.18282	0.32068	0.31568	0.01399	14669	0.000583	0.116	.....
TELO2	16	0.02198	0.75425	0.57043	0.35165	0.00550	14669	0.0006	0.117	.....
KRTAP9-1	17	0.10689	0.04695	0.00100	0.70030	0.05195	14669	0.000604	0.117	.....
TMEM200B	1	0.02897	NA	0.98002	0.11089	0.01190	13691	0.000605	0.117	.?...
SLC22A2	6	0.18082	0.33566	0.51249	0.05495	0.02298	14669	0.000607	0.117	.....
MRPS23	17	0.29171	0.17083	0.03796	0.04196	0.08791	14669	0.000619	0.117	.....
LINC00664	19	0.10589	0.46454	0.41059	0.09790	0.01898	14669	0.000622	0.117	.....
XIRP2	2	NA	0.05894	0.17782	0.16284	0.01698	13245	0.000629	0.118	?....
POLR2H	3	0.56843	0.63736	0.48651	0.00016	0.12687	14669	0.000641	0.119	.....
CDC20	1	0.10989	0.54046	0.73626	0.36064	0.00250	14669	0.000645	0.119	.....
EIF3E	8	0.25075	0.23576	0.94605	NA	0.00180	11265	0.000653	0.119	...?.
ZNF79	9	0.01399	0.65335	0.71528	NA	0.00640	11265	0.00066	0.119	...?.
MIR3134	9	0.49750	0.59441	0.31968	0.00100	0.09690	14669	0.000673	0.119	.....
LOC256880	4	0.28771	0.33367	0.05894	0.01898	0.09491	14669	0.000674	0.119	.....
TARID	6	0.30270	0.54845	0.20080	NA	0.00300	11265	0.00068	0.119	...?.
IL12B	5	0.12388	0.14486	0.12088	0.49650	0.01299	14669	0.000692	0.119	.....
GON4L	1	0.51748	0.34965	0.91808	0.00019	0.12787	14669	0.000697	0.119	.....
FOXO3	6	0.68931	0.55644	0.23077	0.06494	0.00880	14669	0.000698	0.119	.....
TPSG1	16	0.07293	0.28671	0.73926	0.08891	0.02498	14669	0.000698	0.119	.....
CYP11B2	8	0.82318	0.10889	0.09091	0.02298	0.05794	14669	0.000705	0.119	.....
ADPGK-AS1	15	0.13487	0.07592	0.13487	0.08591	0.08492	14669	0.000712	0.119	.....
CLCN1	7	0.19381	0.01299	0.55445	0.01998	0.14585	14669	0.000714	0.119	.....
CD82	11	0.83816	0.27772	0.96204	0.06094	0.00410	14669	0.000726	0.119	.....
SEPT3	22	0.12587	0.00170	0.90410	0.09291	0.08991	14669	0.000728	0.119	.....
SLC22A13	3	0.10090	0.22877	0.42657	0.57443	0.00460	14669	0.00073	0.119	.....
NFXL1	4	0.15185	0.27273	0.15584	0.13786	0.03197	14669	0.000748	0.120	.....
LOC101929570	2	0.17782	NA	0.37962	0.08591	0.01499	13691	0.000749	0.120	.?...

VCAN	5	0.25075	0.70929	0.65335	NA	0.00123	11265	0.000759	0.120	...?.
ST8SIA1	12	0.53746	0.80020	0.14985	NA	0.00150	11265	0.00076	0.120	...?.
PPP1R27	17	0.43357	NA	0.02498	NA	0.00599	10287	0.000775	0.121	.?.?.
LRFN5	14	0.40559	0.11089	0.14585	NA	0.00930	11265	0.000784	0.121	...?.
TTLL12	22	0.11588	0.64036	0.89710	0.33566	0.00230	14669	0.000784	0.121	.....
ALPL	1	0.48751	0.31568	0.38262	0.41558	0.00210	14669	0.000805	0.121	.....
FLJ16779	20	0.01299	0.14985	0.43856	0.00740	0.32567	14669	0.000811	0.121	.....
ADAL	15	0.09590	0.65934	0.29171	0.51648	0.00370	14669	0.000825	0.121	.....
SHF	15	0.56643	0.08791	0.28372	0.16683	0.01499	14669	0.000834	0.121	.....
LOC100130111	15	0.37463	NA	0.01998	0.17383	0.01898	13691	0.00084	0.121	.?...
TCHHL1	1	0.56244	0.22178	0.04695	0.01730	0.09990	14669	0.000847	0.121	.....
THPO	3	0.38961	0.06394	0.74226	0.02498	0.05295	14669	0.00085	0.121	.....
USP7	16	0.50450	0.08092	0.00920	NA	0.02697	11265	0.00086	0.121	...?.
LOC105374960	6	0.49850	0.35864	0.21778	0.01390	0.05794	14669	0.000865	0.121	.....
C8orf59	8	0.90410	0.12388	0.68032	0.45554	0.00100	14669	0.000883	0.121	.....
TNIP2	4	0.14186	0.40060	0.67732	0.12787	0.01399	14669	0.000885	0.121	.....
WNT11	11	0.68132	0.25175	0.30370	0.03297	0.02797	14669	0.00089	0.121	.....
LOC100131635	3	0.09590	0.54745	0.16384	0.46354	0.00799	14669	0.000891	0.121	.....
SNX2	5	0.45554	0.31668	0.54246	NA	0.00210	11265	0.000895	0.121	...?.
RTP2	3	0.11289	0.09690	0.17083	0.60140	0.01399	14669	0.000899	0.121	.....
PPIP5K1	15	0.03996	0.31968	0.44755	0.54446	0.00840	14669	0.000901	0.121	.....
CHRNA6	8	0.08691	0.52148	0.12687	0.03596	0.09291	14669	0.000904	0.121	.....
ACSM6	10	0.75624	0.67932	0.23776	0.04895	0.01099	14669	0.000914	0.121	.....
OXSR1	3	0.09990	0.79121	0.22977	0.72228	0.00210	14669	0.000918	0.121	.....
GPR3	20	NA	NA	0.14985	0.50749	0.00140	12267	0.000932	0.121	??...
CCT6A	7	0.33467	0.30969	0.17782	0.92607	0.00130	14669	0.000937	0.121	.....
UBE2O	17	0.68631	0.03896	0.23576	0.00850	0.13786	14669	0.000937	0.121	.....
BRINP1	9	0.09790	0.26074	0.29770	0.01499	NA	6657	0.000938	0.121	....?
RPS25	11	0.13986	0.06194	0.13487	0.45455	0.02597	14669	0.00094	0.121	.....
MAPT	17	0.26873	0.06194	0.93107	0.03397	0.05195	14669	0.000949	0.121	.....
MPG	16	0.15185	0.16484	0.41159	0.08591	0.04595	14669	0.000949	0.121	.....
ANXA4	2	0.52847	0.63936	0.20579	0.34366	0.00260	14669	0.000953	0.121	.....
ECHDC3	10	0.28472	0.62637	0.17582	0.00280	NA	6657	0.000958	0.121	....?
MAP1LC3B	16	0.36563	0.02797	0.11888	NA	0.02398	11265	0.000961	0.121	...?.
GCK	7	0.26074	0.44356	0.22977	0.16683	0.01499	14669	0.000962	0.121	.....
LOC101928441	12	0.13087	0.07992	0.19980	0.36763	0.02697	14669	0.000966	0.121	.....
DRG2	17	0.18881	0.99600	0.54146	0.00320	0.07393	14669	0.000972	0.121	.....
DEFB126	20	0.56543	0.51848	0.02697	0.18781	0.01570	14669	0.000972	0.121	.....
ENO4	10	0.15285	0.63936	0.59241	0.00150	0.14685	14669	0.000978	0.121	.....
LINC00271	6	NA	0.75524	0.86913	0.02897	0.00799	13245	0.000979	0.121	?....
ZNF512B	20	0.18382	0.79221	0.39960	0.14286	0.00999	14669	0.000985	0.121	.....
EZR-AS1	6	0.19381	0.02897	0.49750	0.00280	0.33067	14669	0.000999	0.122	.....
MAPT-AS1	17	0.20180	0.05095	0.97902	0.04496	0.05594	14669	0.001014	0.123	.....
ZNF30-AS1	19	0.22877	0.46054	0.17383	0.02797	0.07093	14669	0.001015	0.123	.....
CATSPER2P1	15	0.12188	0.53846	0.25974	0.24875	0.01399	14669	0.001023	0.123	.....
GML	8	0.34665	0.07493	0.47852	0.24376	0.01510	14669	0.001034	0.124	.....
ARHGAP15	2	NA	0.34965	0.31169	NA	0.00290	9841	0.00104	0.124	?..?.
SCIN	7	0.00900	0.27273	0.06494	NA	NA	3253	0.001062	0.125	...??
SCN2A	2	0.75524	0.91309	0.80919	0.07592	0.00250	14669	0.001065	0.125	.....
SPATA33	16	0.38062	0.30470	0.12887	NA	0.00790	11265	0.001069	0.125	...?.
TINCR	19	0.00860	0.70929	0.11089	NA	0.03397	11265	0.001073	0.125	...?.
KCP	7	0.58042	0.06793	0.02298	0.16983	0.05594	14669	0.001094	0.125	.....
SSFA2	2	0.02597	0.13886	0.92807	0.46753	0.01399	14669	0.001099	0.125	.....
OR10G9	11	0.94605	0.43856	0.92507	0.27572	0.00074	14669	0.001104	0.125	.....
LOC339166	17	0.34865	0.92907	0.07892	0.06194	0.02797	14669	0.001108	0.125	.....
USP25	21	0.04296	0.04096	0.87812	0.51149	0.01798	14669	0.001113	0.125	.....
ACHE	7	0.14286	0.29770	0.29970	0.17782	0.02697	14669	0.001118	0.125	.....
AGPAT4	6	0.10889	0.07592	0.38462	0.08891	0.08591	14669	0.00113	0.125	.....
SPRED1	15	0.10689	0.92208	0.97502	0.03796	0.01998	14669	0.001136	0.125	.....
OR10G4	11	0.47652	0.32867	0.78422	0.37163	0.00200	14669	0.001137	0.125	.....
CLEC12A	12	0.87313	0.07592	0.05295	0.01199	0.14785	14669	0.001138	0.125	.....
PLA2G4E-AS1	15	0.44456	0.08791	0.10090	0.86114	0.00460	14669	0.001144	0.125	.....
KBTBD8	3	0.28272	0.01199	0.03197	0.67532	0.03596	14669	0.001147	0.125	.....
MPP3	17	0.14386	0.58841	0.30669	0.15285	0.01998	14669	0.001156	0.125	.....
COMMD7	20	0.25075	0.43756	0.34865	0.61039	0.00270	14669	0.001168	0.125	.....
ZNF302	19	0.40260	0.51349	0.14985	0.24076	0.00999	14669	0.001173	0.125	.....
FLJ36000	17	0.76723	0.58541	0.59840	0.05495	0.00799	14669	0.001176	0.125	.....
RPAP3	12	0.18282	0.71528	0.62238	0.01299	0.05495	14669	0.001183	0.125	.....
HDC	15	0.00027	0.13487	0.00210	0.31568	0.50050	14669	0.001183	0.125	.....

HIST2H2BE	1	0.03696	NA	0.31369	0.01798	0.13686	13691	0.001188	0.125	.?...
VASH1	14	0.21778	0.43556	0.14486	NA	0.00999	11265	0.00119	0.125	...?.
SIN3B	19	0.01898	0.82318	0.13087	0.06394	0.10589	14669	0.001196	0.125	.....
MPND	19	0.25275	0.57542	0.42657	0.04895	0.03097	14669	0.001215	0.125	.....
LAT2	7	0.02498	0.65834	0.34865	0.45355	0.01299	14669	0.001215	0.125	.....
TBC1D32	6	NA	0.47253	0.46154	0.93706	0.00030	13245	0.001222	0.125	?....
FAM47E	4	0.00410	0.12787	0.37263	0.41658	0.06993	14669	0.001225	0.125	.....
GUSBP5	4	0.18581	0.43856	0.98601	0.01798	0.04695	14669	0.001262	0.128	.....
CNKSR1	1	0.96703	0.28671	0.02797	0.03696	0.05794	14669	0.001266	0.128	.....
UBLCP1	5	0.17083	0.10989	0.06194	0.58242	0.02398	14669	0.001271	0.128	.....
ERC1	12	0.17882	0.30869	0.02997	0.00799	0.31768	14669	0.001279	0.128	.....
PPIC	5	0.37363	0.79421	0.54046	NA	0.00162	11265	0.001282	0.128	...?.
CACNB2	10	0.24276	0.28472	0.02298	0.19680	0.05694	14669	0.001297	0.128	.....
KCNA3	1	0.03896	0.22478	0.49151	0.18981	0.04795	14669	0.001301	0.128	.....
ASPG	14	0.87912	0.15984	0.02697	0.66134	0.00530	14669	0.001306	0.128	.....
PGLYRP1	19	0.00610	NA	0.05594	0.12987	0.15185	13691	0.001316	0.128	.?...
TFIP11	22	0.30470	0.74725	0.14286	0.77722	0.00170	14669	0.00132	0.128	.....
LOC102477328	5	0.94905	0.01099	0.59640	0.20579	0.01499	14669	0.001321	0.128	.....
LINC00904	19	0.20080	0.33966	0.27173	0.14885	0.02997	14669	0.00133	0.128	.....
FANCM	14	0.13287	0.39560	0.06494	0.03596	0.14885	14669	0.00133	0.128	.....
PRKAG1	12	0.43357	0.53247	0.60040	0.00999	0.05195	14669	0.001333	0.128	.....
SNX27	1	0.06094	0.23377	0.23576	0.03497	0.16983	14669	0.00134	0.128	.....
MATN1-AS1	1	0.11688	0.05794	0.25275	0.57243	0.02298	14669	0.001346	0.128	.....
CPNE8	12	0.77423	0.06494	0.24575	NA	0.00790	11265	0.00135	0.128	...?.
ACSM4	12	0.01099	0.48851	0.06394	0.13786	0.14785	14669	0.001366	0.129	.....
EFCAB5	17	0.90210	0.07493	0.59341	0.03696	0.03397	14669	0.00138	0.129	.....
TRMT6	20	0.48651	0.07393	0.59341	0.13986	0.02298	14669	0.001382	0.129	.....
LINC00434	13	0.03796	0.69630	0.24975	0.15984	0.04296	14669	0.001386	0.129	.....
HOXD10	2	0.48551	0.92507	0.14985	0.02797	0.03596	14669	0.001388	0.129	.....
CSHL1	17	0.86214	0.71928	0.03297	0.02498	0.04895	14669	0.001395	0.129	.....
MROH1	8	0.28072	0.17083	0.02897	0.80719	0.01199	14669	0.001422	0.130	.....
GNPTAB	12	0.74126	0.09790	0.67932	0.13487	0.01299	14669	0.001443	0.130	.....
CHRNB1	17	0.72827	0.01580	0.60739	0.00770	0.15684	14669	0.001446	0.130	.....
CNGA1	4	0.19181	0.13087	0.41359	0.07093	0.07393	14669	0.001451	0.130	.....
LOC101929064	4	0.20180	0.07093	0.74525	0.07393	0.06194	14669	0.001459	0.130	.....
AHCTF1P1	2	0.07093	NA	0.08192	0.37962	0.02398	13691	0.001467	0.130	.?...
MBTPS1	16	0.25674	0.09690	0.49850	0.23876	0.02398	14669	0.001468	0.130	.....
UBTF	17	0.46553	0.17982	0.43656	0.44056	0.00560	14669	0.001473	0.130	.....
WISP2	20	0.90010	0.24675	0.11888	0.08891	0.02597	14669	0.001479	0.130	.....
STRC	15	0.05095	0.21179	0.42857	0.96603	0.00590	14669	0.00149	0.130	.....
CA13	8	0.49950	0.22777	0.42657	0.65035	0.00240	14669	0.001497	0.130	.....
TTLL5	14	0.18881	0.51548	0.53646	NA	0.00540	11265	0.001498	0.130	...?.
HPS4	22	0.28871	0.81319	0.18681	0.79620	0.00155	14669	0.001508	0.130	.....
ZNF579	19	0.17882	0.37962	0.07193	0.78821	0.00790	14669	0.001515	0.130	.....
FAM129A	1	0.04595	0.67932	0.65235	0.58342	0.00510	14669	0.001519	0.130	.....
STK32A	5	0.64336	0.08492	0.13087	0.06294	0.07393	14669	0.001527	0.130	.....
ARL8A	1	0.81618	0.29171	0.21878	0.91808	0.00080	14669	0.001533	0.130	.....
LOC344887	3	0.26474	0.30070	0.41259	0.58841	0.00470	14669	0.001567	0.130	.....
SYT11	1	0.61039	0.51249	0.86513	0.00550	0.04895	14669	0.001571	0.130	.....
EHD3	2	0.14186	0.63936	0.00260	0.48152	0.03996	14669	0.001571	0.130	.....
SIX4	14	0.40360	0.59740	0.15385	0.59441	0.00340	14669	0.001571	0.130	.....
LOC100505915	16	0.43556	NA	0.15385	NA	0.00530	10287	0.001573	0.130	.?.?.
KRTDAP	19	0.09990	0.75724	0.32268	0.07592	0.04595	14669	0.001574	0.130	.....
CASC23	11	0.10390	0.82118	0.08092	0.02398	0.14186	14669	0.001586	0.130	.....
ADGRA2	8	0.64436	0.04695	0.39161	NA	0.00999	11265	0.001591	0.130	...?.
OR10G7	11	0.56344	0.69630	0.42258	0.57343	0.00110	14669	0.001593	0.130	.....
IFI30	19	0.10190	0.52747	0.17682	0.00041	0.53746	14669	0.001599	0.130	.....
ZNF462	9	0.91009	0.71728	0.28072	0.03197	0.01898	14669	0.001603	0.130	.....
CDK10	16	0.24675	0.21778	0.07892	0.59940	0.01499	14669	0.001615	0.130	.....
BRD8	5	0.04595	0.59441	0.32867	0.10190	0.06394	14669	0.001623	0.130	.....
UGT2B15	4	0.97003	0.11988	0.31568	0.04096	0.04096	14669	0.001632	0.130	.....
PSMC5	17	0.93806	0.60539	0.18182	0.00072	0.15884	14669	0.001635	0.130	.....
LINC00392	13	0.12687	0.82218	0.08891	NA	0.01399	11265	0.001637	0.130	...?.
PRAC1	17	0.13586	0.60040	0.89011	0.22478	0.00910	14669	0.001657	0.130	.....
SYDE2	1	0.03397	0.07393	0.08691	0.00350	0.78322	14669	0.001664	0.130	.....
C3orf79	3	0.84515	0.03596	0.14386	0.05495	0.08691	14669	0.001666	0.130	.....
SLC25A13	7	0.37363	0.96104	0.00899	NA	0.01299	11265	0.001668	0.130	...?.
FBXL7	5	0.77822	0.54645	0.60739	0.52947	0.00082	14669	0.001673	0.130	.....
CASZ1	1	0.53447	0.00530	0.82917	0.16184	0.04196	14669	0.001675	0.130	.....

MS4A1	11	0.34066	0.10290	0.45854	0.01299	0.15584	14669	0.001678	0.130	.....
WDR92	2	0.16683	0.68032	0.31868	0.29371	0.01199	14669	0.00169	0.130	.....
ZFYVE21	14	0.07592	0.68432	0.91409	0.23177	0.01099	14669	0.001693	0.130	.....
LINC01270	20	0.15684	0.13387	0.64735	0.00065	0.45055	14669	0.001698	0.130	.....
USP14	18	0.21379	0.64535	0.46953	0.03297	0.05295	14669	0.001701	0.130	.....
CHAF1A	19	0.68032	0.36663	0.27872	0.03696	0.04096	14669	0.001702	0.130	.....
RPS6KA2	6	NA	0.04995	0.16384	0.25774	0.03097	13245	0.001705	0.130	?....
SLC39A10	2	0.48152	0.04595	0.77922	0.02697	0.08591	14669	0.001708	0.130	.....
HCRTR1	1	0.29970	0.06693	0.00014	0.15984	0.31269	14669	0.001709	0.130	.....
ADPGK	15	0.10090	0.15085	0.17782	0.15085	0.09391	14669	0.001709	0.130	.....
LAMTOR3	4	0.15385	0.55445	0.06494	0.02597	0.16783	14669	0.001728	0.130	.....
FAM174B	15	0.50350	0.52547	0.44555	0.09890	0.01598	14669	0.001738	0.130	.....
TRIP11	14	0.64635	0.45355	0.35764	0.16583	0.00999	14669	0.001751	0.130	.....
POMP	13	0.05594	0.72228	0.44056	0.07592	0.05794	14669	0.001752	0.130	.....
LMTK3	19	0.17582	0.78422	0.53846	0.11788	0.01898	14669	0.001762	0.130	.....
HOXB1	17	0.11089	NA	0.38362	0.01698	0.11489	13691	0.001762	0.130	.?...
LOC401554	9	0.77023	0.00340	0.61339	0.00360	0.30669	14669	0.001767	0.130	.....
LUNAR1	15	0.39361	0.00670	0.37063	NA	0.03497	11265	0.001768	0.130	...?.
LINC01039	13	0.04695	0.68132	0.12887	0.07193	0.11389	14669	0.001771	0.130	.....
MNX1-AS1	7	0.02198	0.83516	0.67532	0.02897	0.10290	14669	0.001774	0.130	.....
LINGO1	15	0.87013	0.09990	0.39461	0.20979	0.01290	14669	0.001792	0.130	.....
VTCN1	1	0.15285	0.55944	0.35664	0.22478	0.01998	14669	0.001793	0.130	.....
PUS3	11	0.14985	0.71828	0.63137	0.08891	0.02597	14669	0.001794	0.130	.....
ERBIN	5	0.05694	0.79221	0.63337	0.04595	0.06094	14669	0.001794	0.130	.....
LMO1	11	0.58142	0.08891	0.09191	0.31968	0.02797	14669	0.001805	0.131	.....
PPP2R1A	19	0.58541	0.29970	0.82817	0.04595	0.02398	14669	0.001809	0.131	.....
LOC101927869	21	0.04096	0.01199	0.86713	0.04895	0.25375	14669	0.001822	0.131	.....
FBN1	15	0.79820	0.76324	0.00410	0.08192	0.05295	14669	0.001826	0.131	.....
PUS1	12	0.42458	0.45355	0.41858	NA	0.00460	11265	0.001827	0.131	...?.
CCNA2	4	0.20180	NA	0.92607	0.00680	0.08192	13691	0.001838	0.131	.?...
DDX12P	12	0.34865	0.37063	0.00340	0.24276	0.07393	14669	0.001858	0.131	.....
PDIA3	15	0.10689	0.73427	0.36364	0.29770	0.01499	14669	0.001881	0.131	.....
LOC101928030	12	0.50450	0.24675	0.03197	0.67033	0.01099	14669	0.001883	0.131	.....
SNX8	7	0.09690	0.19880	0.42957	NA	0.02298	11265	0.001912	0.131	...?.
APPBP2	17	0.01798	NA	0.18781	0.00799	0.36763	13691	0.001915	0.131	.?...
PLEKHF2	8	0.84515	0.06893	0.20480	0.25574	0.01998	14669	0.001916	0.131	.....
SLC48A1	12	0.50250	0.94106	0.12488	0.03896	0.04096	14669	0.001917	0.131	.....
MAEA	4	0.70230	0.09191	0.49051	0.36064	0.00860	14669	0.001918	0.131	.....
DHX36	3	0.14685	0.31768	0.11788	0.13886	0.07792	14669	0.001919	0.131	.....
RPL12	9	0.06394	0.65934	0.77922	NA	0.00799	11265	0.001919	0.131	...?.
SCRN3	2	0.98801	0.21179	0.26773	0.11489	0.01798	14669	0.001922	0.131	.....
OR5AK4P	11	0.56743	0.00200	0.98501	0.06793	0.09291	14669	0.001924	0.131	.....
LOC730102	1	0.39161	0.64735	0.40859	0.07792	0.02398	14669	0.001924	0.131	.....
ZNF16	8	0.04995	0.29271	0.08691	NA	0.05495	11265	0.00196	0.131	...?.
NEURL1B	5	0.08691	0.04196	0.20480	NA	0.06793	11265	0.001962	0.131	...?.
TTLL1	22	0.57443	0.88611	0.26673	0.08292	0.01598	14669	0.001966	0.131	.....
LOC102723968	13	0.00019	0.98002	0.10689	0.49051	0.10290	14669	0.001968	0.131	.....
LMBR1L	12	0.83117	0.94306	0.36863	0.02398	0.02098	14669	0.001969	0.131	.....
LAIR1	19	0.77123	0.10390	0.28571	0.27473	0.01499	14669	0.00199	0.131	.....
LOC101928769	5	0.80519	0.74925	0.61339	0.08991	0.00699	14669	0.001993	0.131	.....
UCKL1	20	0.48951	0.89710	0.26973	0.15485	0.01050	14669	0.001995	0.131	.....
LOC101929709	8	0.16783	0.30270	0.01299	0.77223	0.02498	14669	0.001999	0.131	.....
LINC00520	14	0.16783	0.09790	0.84915	0.13586	0.04695	14669	0.002004	0.131	.....
CRYBB1	22	0.40959	0.35964	0.21079	0.41858	0.00999	14669	0.002012	0.131	.....
MIR600HG	9	0.07193	NA	0.09391	0.64535	0.01499	13691	0.002012	0.131	.?...
UNK	17	0.17882	0.33666	0.16184	0.17083	0.05395	14669	0.002015	0.131	.....
KMT2D	12	0.98501	0.29371	0.52747	0.01399	0.04595	14669	0.002015	0.131	.....
GLB1	3	0.17283	0.10789	0.62338	0.11688	0.06294	14669	0.002016	0.131	.....
ZSCAN29	15	0.12787	0.70529	0.30170	0.35065	0.01399	14669	0.002024	0.131	.....
CKMT1B	15	0.02498	NA	0.45754	0.90809	0.00560	13691	0.002035	0.131	.?...
SNORA29	6	0.24975	0.68232	0.27273	0.08791	0.03796	14669	0.002041	0.131	.....
LOC100507006	2	0.62138	0.00699	0.15385	0.02897	0.25674	14669	0.002042	0.131	.....
GALNT11	7	0.29870	NA	0.15684	0.34266	0.01199	13691	0.002047	0.131	.?...
UTP11	1	0.56843	0.80919	0.20080	0.06194	0.02697	14669	0.002055	0.131	.....
FBXO31	16	0.15684	0.05594	0.10490	NA	0.06294	11265	0.002066	0.131	...?.
GM140	1	0.02398	0.12188	0.31169	0.01199	0.47852	14669	0.002066	0.131	.....
CCNO	5	0.68631	0.10290	0.35964	NA	0.00899	11265	0.002076	0.131	...?.
AKAP1	17	0.27273	0.09990	0.50450	0.00940	0.22378	14669	0.002087	0.131	.....
CNTN3	3	0.02897	0.31768	0.35465	0.04496	0.19880	14669	0.002092	0.131	.....

GRPEL1	4	0.38262	0.28172	0.09690	0.02498	0.15684	14669	0.002098	0.131	.....
DNAH7	2	0.30969	0.06194	0.75225	0.02298	0.13487	14669	0.002106	0.131	.....
WDR76	15	0.21978	0.86513	0.37562	0.30070	0.00880	14669	0.00211	0.131	.....
CLEC4D	12	0.68831	NA	0.05694	0.31768	0.00999	13691	0.002119	0.131	.?...
LOC102724612	8	0.10789	0.31169	0.00066	0.21678	0.22577	14669	0.002122	0.131	.....
SHB	9	0.06893	0.18681	0.74625	NA	0.02098	11265	0.002131	0.131	...?.
REC114	15	0.34366	0.75924	0.52148	0.00550	0.10290	14669	0.002131	0.131	.....
CATSPER2	15	0.11588	0.44056	0.24076	0.46653	0.01798	14669	0.002134	0.131	.....
KAT6A	8	0.69131	0.22977	0.72228	0.00150	0.16683	14669	0.002137	0.131	.....
PRKCI	3	0.00480	0.94805	0.27273	0.05994	0.17483	14669	0.00215	0.131	.....
TLR4	9	0.15085	0.19980	0.02697	0.11688	NA	6657	0.002156	0.131	....?
MRPS18A	6	0.67133	0.05994	0.79321	0.01998	0.08691	14669	0.002161	0.131	.....
TRMT5	14	0.29371	0.84715	0.08891	0.69131	0.00470	14669	0.002161	0.131	.....
LOC101928514	17	0.75724	0.04895	0.60240	0.00599	0.17083	14669	0.002168	0.131	.....
C1orf229	1	0.54046	0.77123	0.04296	0.00310	0.23077	14669	0.002186	0.132	.....
CDC23	5	0.06494	0.57043	0.31469	0.08991	0.08292	14669	0.002197	0.132	.....
MMEL1	1	0.42058	0.06294	0.74725	0.17283	0.03097	14669	0.002214	0.132	.....
SH3TC1	4	0.03596	0.40460	0.00590	0.16883	0.24176	14669	0.002215	0.132	.....
KIAA0232	4	0.18482	0.48452	0.37163	0.03497	0.09491	14669	0.002221	0.132	.....
ABHD17C	15	0.90809	0.56144	0.67532	NA	0.00120	11265	0.002222	0.132	...?.
KDM4A-AS1	1	0.17383	0.67033	0.17283	NA	0.01399	11265	0.002241	0.132	...?.
PRDM15	21	0.00900	0.03896	0.87812	NA	0.07992	11265	0.002243	0.132	...?.
LOC105369860	12	0.00400	0.07393	0.07193	NA	0.23077	11265	0.00225	0.132	...?.
GPS1	17	0.65135	0.11289	0.92208	0.11389	0.01998	14669	0.002252	0.132	.....
B3GAT2	6	0.04496	0.20280	0.73926	0.37662	0.03097	14669	0.002257	0.132	.....
MIR548N	2	0.01998	0.99600	0.12887	0.01898	0.26074	14669	0.002264	0.132	.....
NXPH2	2	0.16983	0.46753	0.95005	0.33367	0.00810	14669	0.002266	0.132	.....
GHITM	10	0.19980	0.11988	0.50350	0.52048	0.01598	14669	0.002271	0.132	.....
GSDMA	17	0.83117	0.50749	0.11489	0.24975	0.01100	14669	0.002281	0.132	.....
OXA1L	14	0.25075	0.00999	0.11788	0.63836	0.05395	14669	0.002285	0.132	.....
KRT6C	12	0.90010	0.98002	0.05295	0.13387	0.01399	14669	0.002287	0.132	.....
TRIM17	1	0.12887	0.86414	0.45155	0.03397	0.07193	14669	0.002288	0.132	.....
RSPH9	6	0.70729	0.12088	0.58841	0.19980	0.01598	14669	0.002319	0.132	.....
GNAL	18	0.22478	0.57942	0.38362	0.02398	0.09690	14669	0.002323	0.132	.....
MCM8	20	0.41658	0.58042	0.21878	0.02498	0.08492	14669	0.002325	0.132	.....
TTN-AS1	2	0.23976	0.72228	0.10290	0.01798	0.15385	14669	0.00233	0.132	.....
TSR3	16	0.41459	NA	0.91908	0.26174	0.00410	13691	0.002336	0.132	.?...
MYD88	3	0.28671	0.74426	0.07093	0.22178	0.02997	14669	0.002336	0.132	.....
GARNL3	9	0.10290	0.42058	0.69930	NA	0.01199	11265	0.002348	0.132	...?.
TCL6	14	0.22777	0.16084	0.32967	NA	0.02198	11265	0.002352	0.132	...?.
DMKN	19	0.07692	0.74825	0.86613	0.04496	0.05794	14669	0.002352	0.132	.....
SERF2	15	0.16583	0.74026	0.27672	0.20080	0.02597	14669	0.002357	0.132	.....
CABP5	19	0.55644	0.07692	0.94805	0.15085	0.02198	14669	0.002361	0.132	.....
CHMP1A	16	0.47253	0.46753	0.08192	NA	0.01310	11265	0.002366	0.132	...?.
LOC101929771	1	0.13786	0.66034	0.15884	0.57942	0.01199	14669	0.002374	0.132	.....
PRAMENP	22	0.26973	0.61439	0.63137	0.01399	0.08691	14669	0.002377	0.132	.....
RPRD1B	20	0.67532	0.25974	0.43457	0.09091	0.02897	14669	0.002381	0.132	.....
CELF3	1	0.06094	0.37762	0.15684	0.05894	0.18681	14669	0.002381	0.132	.....
RPGRIP1	14	0.03197	0.17283	0.48352	0.61239	0.02997	14669	0.002388	0.132	.....
TMEM126B	11	0.71329	0.10490	0.49351	NA	0.00799	11265	0.002417	0.132	...?.
DNAJC25-GNG10	9	0.30569	0.33167	0.36164	0.00520	0.22478	14669	0.002423	0.132	.....
SLITRK5	13	0.13487	0.79720	0.53546	NA	0.00750	11265	0.002425	0.132	...?.
INTS10	8	0.24276	0.93107	0.09391	NA	0.01150	11265	0.002425	0.132	...?.
AHCYL2	7	0.70729	0.18082	0.97003	NA	0.00310	11265	0.002426	0.132	...?.
DPEP1	16	0.58242	0.49650	0.05295	NA	0.01260	11265	0.00243	0.132	...?.
RPL26	17	0.26773	0.56344	0.54146	0.10290	0.02997	14669	0.002435	0.132	.....
YAF2	12	0.42657	0.48352	0.00780	NA	0.03197	11265	0.002452	0.133	...?.
LSM5	7	0.26074	0.72328	0.58741	NA	0.00490	11265	0.002477	0.133	...?.
SURF1	9	0.05295	0.01798	0.22977	0.16683	0.24675	14669	0.002485	0.133	.....
LINC00264	10	0.01798	NA	0.23576	NA	0.04096	10287	0.002485	0.133	.?.?.
UBAP2	9	0.17183	0.10290	0.92208	NA	0.01598	11265	0.002486	0.133	...?.
RFNG	17	0.34665	0.02298	0.38561	0.02797	0.23576	14669	0.002493	0.133	.....
IFNA5	9	NA	0.00420	NA	0.05794	NA	4382	0.002498	0.133	?.?.?
OPA1-AS1	3	0.04296	0.82617	0.76823	0.07892	0.05495	14669	0.002501	0.133	.....
OCSTAMP	20	0.66533	0.27373	0.17383	0.07093	0.05694	14669	0.002512	0.133	.....
NLRX1	11	0.41259	0.36963	0.61938	0.08492	0.03197	14669	0.002514	0.133	.....
FRMD8	11	0.23676	0.51648	0.34965	0.43157	0.01099	14669	0.00253	0.133	.....
MORN1	1	0.48452	0.08891	0.49251	0.13786	0.04496	14669	0.002536	0.133	.....
TIAM2	6	0.17582	0.83516	0.44056	0.94006	0.00170	14669	0.002555	0.133	.....

IRF8	16	0.93407	0.40160	0.44955	0.29670	0.00470	14669	0.002558	0.133	.....
RAB12	18	0.76523	0.20879	0.35265	0.97702	0.00140	14669	0.002559	0.133	.....
EMILIN3	20	0.21379	0.31369	0.79421	0.05894	0.05894	14669	0.002562	0.133	.....
MAPKAPK3	3	0.60140	0.70829	0.65934	0.16883	0.00720	14669	0.002574	0.133	.....
CDCA7	2	0.55045	0.12787	0.53047	0.04995	0.06993	14669	0.002578	0.133	.....
PMEPA1	20	0.84915	0.82517	0.05395	0.47652	0.00470	14669	0.00259	0.133	.....
RBMS1	2	NA	0.33866	0.19281	0.10390	0.03996	13245	0.002594	0.133	?....
ACAA1	3	0.12687	0.04995	0.24975	0.45155	0.05994	14669	0.002595	0.133	.....
FAM27E5	17	0.85614	0.59840	0.98202	0.01998	0.02198	14669	0.002598	0.133	.....
PCAT18	18	0.26274	0.06593	0.36064	0.03596	0.19580	14669	0.00261	0.133	.....
PCNT	21	0.38362	0.01499	0.05594	0.34865	0.10490	14669	0.002613	0.133	.....
TGFB3	14	0.27173	0.35564	0.46254	NA	0.01099	11265	0.002617	0.133	...?.
LCOR	10	0.46553	0.72627	0.59740	0.01320	0.06394	14669	0.00263	0.133	.....
CRX	19	0.68032	0.39760	0.11389	0.35664	0.01299	14669	0.002634	0.133	.....
EIF4H	7	0.12188	0.59840	0.37962	0.35664	0.01898	14669	0.002635	0.133	.....
THSD1	13	0.01798	0.29271	0.17083	0.03097	0.39461	14669	0.002637	0.133	.....
CIR1	2	0.99201	0.83317	0.45754	0.03996	0.01598	14669	0.002642	0.133	.....
LLPH-AS1	12	0.57542	0.17383	0.23676	0.21279	0.03097	14669	0.002653	0.133	.....
FGF12	3	0.10589	0.26873	0.57642	NA	0.02098	11265	0.002691	0.134	...?.
TMEM261	9	0.32967	0.05095	0.02697	0.53846	0.06494	14669	0.002692	0.134	.....
CLEC4E	12	0.00900	0.19281	0.24675	0.49850	0.09191	14669	0.002693	0.134	.....
LOC284865	22	0.39161	0.90310	0.38661	0.36863	0.00530	14669	0.002694	0.134	.....
CCDC110	4	0.04595	0.11688	0.71928	0.30270	0.05994	14669	0.002699	0.134	.....
RAB3IL1	11	0.10390	0.31768	0.09291	0.81818	0.01998	14669	0.002702	0.134	.....
CYP8B1	3	0.15185	0.71329	0.90509	0.01399	0.09191	14669	0.002704	0.134	.....
C1orf106	1	0.02398	0.26274	0.49650	0.28072	0.07393	14669	0.002729	0.134	.....
MPV17L2	19	0.84316	0.15684	0.15984	0.08392	0.05894	14669	0.002752	0.134	.....
HOXD1	2	0.17682	0.40060	0.40160	0.01898	0.16583	14669	0.002753	0.134	.....
TEN1	17	0.37263	0.02398	0.88012	0.33766	0.02697	14669	0.002757	0.134	.....
ALDH1A2	15	0.22777	0.05095	0.05095	NA	0.08492	11265	0.002759	0.134	...?.
CDC42SE2	5	0.02897	0.57642	0.03297	0.37463	0.09690	14669	0.002764	0.134	.....
SYNE1	6	0.31668	0.50849	0.06394	NA	0.02198	11265	0.002772	0.134	...?.
PGBD5	1	NA	0.57842	0.07692	0.71828	0.00450	13245	0.002774	0.134	?....
RALGPS2	1	0.12587	0.65834	0.41858	0.02697	0.12288	14669	0.002775	0.134	.....
NME5	5	0.05395	0.69730	0.39960	0.09091	0.08492	14669	0.002776	0.134	.....
ECHS1	10	0.21379	0.18082	0.93806	0.07592	0.06094	14669	0.002776	0.134	.....
TNFSF11	13	0.12388	0.12887	0.04995	NA	0.08392	11265	0.002781	0.134	...?.
RAP1GAP2	17	0.00750	0.47652	NA	0.25674	0.06993	13818	0.002783	0.134	..?..
GPR4	19	0.21778	0.31568	0.24076	0.01798	0.21179	14669	0.002806	0.134	.....
FAM213A	10	0.89111	0.16683	0.01499	0.04096	0.16883	14669	0.002817	0.134	.....
KLK15	19	0.55844	0.08392	0.37562	0.17782	0.04296	14669	0.002817	0.134	.....
ITGAV	2	0.88312	0.11189	0.08392	0.00600	0.28472	14669	0.002818	0.134	.....
CNST	1	0.80619	0.02198	0.25974	0.05095	0.13487	14669	0.002839	0.135	.....
LCN8	9	0.36464	0.78821	0.65435	0.01598	0.06494	14669	0.002842	0.135	.....
NSMAF	8	0.48951	0.53247	0.52647	0.06294	0.03497	14669	0.002851	0.135	.....
PPARG	3	0.14386	0.00350	0.59740	0.17083	0.18182	14669	0.002854	0.135	.....
OR6T1	11	0.90909	0.86114	0.78521	0.56244	0.00054	14669	0.002858	0.135	.....
DUSP3	17	0.65634	0.40060	0.09491	0.30969	0.01898	14669	0.002873	0.135	.....
KIF20A	5	0.09291	0.77622	0.24575	0.13287	0.06194	14669	0.002882	0.135	.....
CENPF	1	0.90909	0.90609	0.02298	0.58741	0.00440	14669	0.002884	0.135	.....
USP54	10	0.21578	0.17383	0.78422	0.60939	0.00899	14669	0.002886	0.135	.....
HOXB-AS3	17	0.06693	0.18681	0.22178	0.24476	0.10090	14669	0.002895	0.135	.....
MOCS2	5	0.16783	0.85115	0.19281	NA	0.01399	11265	0.002918	0.135	...?.
SERPINA11	14	0.60739	0.40160	0.10390	NA	0.01290	11265	0.002941	0.135	...?.
PDE3A	12	0.09790	0.53147	0.30270	NA	0.02298	11265	0.002942	0.135	...?.
SPTSSA	14	0.07393	0.67732	0.42458	NA	0.01798	11265	0.002951	0.135	...?.
FAM71B	5	0.28172	0.93207	0.79620	0.37063	0.00410	14669	0.002956	0.135	.....
NDUFA6	22	0.05994	0.12587	0.11189	0.53846	0.07592	14669	0.002956	0.135	.....
LMNB1	5	0.45954	0.45854	0.22777	NA	0.01050	11265	0.002969	0.135	...?.
CGA	6	0.02398	0.88811	0.07692	0.04795	0.24875	14669	0.002969	0.135	.....
VEGFA	6	0.27373	0.14186	0.11988	0.02098	0.30170	14669	0.002976	0.135	.....
CALCB	11	0.22877	0.85015	0.07692	0.65335	0.00999	14669	0.002977	0.135	.....
SMDT1	22	0.04196	0.23277	0.12388	0.27073	0.12787	14669	0.002995	0.135	.....
FGF4	11	0.87912	0.93906	0.93906	0.14386	0.00320	14669	0.002995	0.135	.....
LOC100506858	5	0.27672	0.32068	0.02198	0.07692	NA	6657	0.003001	0.135	....?
OR52E4	11	0.72128	0.61538	0.28072	NA	0.00450	11265	0.003006	0.135	...?.
PRELID2	5	0.18382	0.08891	0.94505	0.43357	0.01998	14669	0.00301	0.135	.....
OR4C45	11	0.22478	0.85215	0.83516	0.14086	0.01598	14669	0.003013	0.135	.....
ZEB1	10	0.06793	0.26474	0.33566	NA	0.04096	11265	0.003017	0.135	...?.

ULK1	12	0.39261	0.36164	0.49051	NA	0.00899	11265	0.003023	0.135	...?.
BFSP1	20	0.15285	0.99401	0.63037	0.40859	0.00670	14669	0.003025	0.135	.....
BPIFB3	20	0.59141	0.63037	0.31668	0.15584	0.01798	14669	0.003028	0.135	.....
LOC100288866	17	0.39461	0.98901	0.17183	0.03397	0.06893	14669	0.003041	0.135	.....
MAML2	11	0.53447	0.34066	0.86713	0.88811	0.00108	14669	0.003042	0.135	.....
DDX5	17	0.50549	NA	0.06893	0.45455	0.01199	13691	0.003045	0.135	.?...
HIGD2B	15	0.11489	0.42657	0.12787	0.06394	0.17283	14669	0.003045	0.135	.....
RNASET2	6	0.48651	0.71329	0.34366	0.10190	0.02697	14669	0.003052	0.135	.....
C17orf98	17	0.62837	0.42058	0.20579	0.07592	0.05095	14669	0.003066	0.135	.....
LGALSL	2	0.27572	0.49351	0.00440	0.01499	0.42757	14669	0.00307	0.135	.....
OR2K2	9	0.22777	0.01798	0.69530	0.03397	0.24575	14669	0.003073	0.135	.....
DEFB127	20	0.16783	NA	0.01099	NA	0.05195	10287	0.003078	0.135	.?.?.
DNAJB14	4	0.26973	0.45155	0.06593	0.09391	0.11289	14669	0.003079	0.135	.....
EDDM3B	14	0.45155	0.00230	0.08492	0.96903	0.04196	14669	0.003091	0.135	.....
PIPSL	10	0.26673	NA	0.74226	NA	0.00550	10287	0.003098	0.135	.?.?.
C18orf25	18	0.08991	0.04496	0.41558	NA	0.06793	11265	0.003107	0.135	...?.
LINC00888	3	0.32068	0.53846	0.23477	0.02498	0.12887	14669	0.00311	0.135	.....
HIPK1-AS1	1	0.45654	0.79321	0.44456	0.15085	0.01598	14669	0.003113	0.135	.....
XRCC2	7	0.36663	0.31668	0.17582	0.77722	0.00820	14669	0.003116	0.135	.....
BTBD6	14	0.08991	0.40460	0.71429	0.78721	0.00699	14669	0.003125	0.135	.....
TMEM9	1	0.98302	0.01299	0.02498	0.12687	0.16284	14669	0.003129	0.135	.....
FAM160B2	8	0.38462	0.00660	0.78521	0.06194	0.16883	14669	0.003136	0.135	.....
ZNF276	16	0.57443	0.20579	0.53546	0.12687	0.03397	14669	0.003143	0.135	.....
CCND2-AS1	12	0.08791	0.10090	0.84615	NA	0.03197	11265	0.003155	0.135	...?.
EMP3	19	0.09590	0.06294	0.63237	0.06893	0.19381	14669	0.003171	0.135	.....
DPP10-AS1	2	0.63936	0.97003	0.52747	0.18482	0.00670	14669	0.003172	0.135	.....
METRN	16	0.36464	NA	0.78422	0.00140	0.19281	13691	0.003179	0.135	.?...
ITLN1	1	0.08192	0.98701	0.99800	0.22378	0.01399	14669	0.003186	0.135	.....
MAD2L1BP	6	0.80220	NA	0.53946	0.11489	0.01170	13691	0.003193	0.135	.?...
POLH	6	0.65235	0.41159	0.53347	0.07093	0.03297	14669	0.003195	0.135	.....
COMMD9	11	0.11289	0.63536	0.47253	0.08392	0.07293	14669	0.003205	0.135	.....
LRRC8C	1	0.14585	0.43457	0.15085	0.70529	0.01698	14669	0.003206	0.135	.....
SPG11	15	0.04396	0.75724	0.08891	0.43656	0.04895	14669	0.003207	0.135	.....
LINC01376	2	0.03097	0.52348	0.08492	0.00510	0.64036	14669	0.003216	0.135	.....
RASA2	3	0.10789	0.85714	0.47353	0.00170	0.33367	14669	0.003216	0.135	.....
PGGT1B	5	0.59041	0.16683	0.32068	NA	0.01399	11265	0.003237	0.135	...?.
SPATA2L	16	0.31768	0.24076	0.13487	0.67133	0.01698	14669	0.003251	0.136	.....
LAPTM5	1	0.35764	0.48851	0.22977	0.28372	0.02398	14669	0.003287	0.136	.....
MNAT1	14	0.60040	0.97003	0.18182	0.62438	0.00280	14669	0.003289	0.136	.....
OR9G4	11	0.07193	0.17982	0.94705	0.36064	0.03297	14669	0.003289	0.136	.....
MUT	6	0.18681	0.73626	0.00999	NA	0.04895	11265	0.003312	0.137	...?.
ALOX15B	17	0.15285	0.91508	0.59241	0.11089	0.03397	14669	0.003314	0.137	.....
TAF4	20	0.31269	0.03097	0.23976	NA	0.05495	11265	0.00332	0.137	...?.
SCAND1	20	0.39760	0.58541	0.18581	0.31968	0.01898	14669	0.003323	0.137	.....
SLC17A9	20	0.30769	0.57143	0.82418	0.06893	0.03696	14669	0.003331	0.137	.....
RCC2	1	0.19680	0.16683	0.87113	0.03097	0.13786	14669	0.003346	0.137	.....
RSPH6A	19	0.01898	0.87712	0.60739	0.14386	0.07093	14669	0.003354	0.137	.....
LINC01506	9	0.98901	NA	0.25874	0.70030	0.00133	13691	0.003356	0.137	.?...
TMEM5-AS1	12	0.06494	0.71129	0.02797	NA	0.06394	11265	0.003371	0.137	...?.
CEP19	3	0.14785	0.96304	0.11688	0.22278	0.04196	14669	0.003379	0.137	.....
AP1G1	16	0.15784	0.24376	0.05794	0.09091	0.21379	14669	0.003379	0.137	.....
NOX4	11	0.01698	0.34765	0.70030	NA	0.04396	11265	0.003388	0.137	...?.
LOC101927768	6	0.52947	0.16683	0.39960	0.22877	0.03097	14669	0.00339	0.137	.....
HRASLS5	11	0.00830	0.03796	0.04895	0.76623	0.19680	14669	0.003393	0.137	.....
TMEM145	19	0.03796	NA	0.55644	0.05994	0.12488	13691	0.003412	0.137	.?...
MAGI2-AS3	7	0.63037	0.56543	0.71129	0.03397	0.03996	14669	0.003422	0.137	.....
LACC1	13	0.10689	0.10390	0.24975	0.18082	0.14286	14669	0.003422	0.137	.....
C11orf65	11	0.77622	0.90709	0.73027	NA	0.00140	11265	0.003451	0.138	...?.
CREBZF	11	0.63137	0.13586	0.69231	NA	0.00899	11265	0.003479	0.139	...?.
TXNRD3NB	3	0.34965	0.41758	0.04895	0.97403	0.00899	14669	0.003485	0.139	.....
SFXN2	10	0.30470	0.99700	0.08192	0.32468	0.02098	14669	0.003498	0.139	.....
COL16A1	1	0.06793	0.49550	0.00120	0.10989	0.39760	14669	0.003499	0.139	.....
USP12-AS2	13	0.74925	0.31668	0.10090	0.01399	0.18382	14669	0.003507	0.139	.....
MAP2K3	17	0.07293	0.63437	0.95405	0.00310	0.27972	14669	0.003511	0.139	.....
FOXB1	15	0.13786	0.10689	0.08891	NA	0.08591	11265	0.003517	0.139	...?.
CLCA3P	1	0.63037	0.64735	0.59441	0.15485	0.01299	14669	0.003529	0.139	.....
OR10C1	6	0.08292	0.97702	0.24675	0.08891	0.08591	14669	0.003553	0.140	.....
NOP2	12	0.45754	0.38861	0.05694	0.13187	0.08492	14669	0.003594	0.140	.....
HLA-F-AS1	6	0.46853	0.83716	0.09690	NA	0.01130	11265	0.003603	0.140	...?.

EEF2K	16	0.14685	0.22877	0.68432	0.26274	0.04096	14669	0.003606	0.140	.....
PYGL	14	0.08492	0.90110	0.41359	0.32268	0.02398	14669	0.003607	0.140	.....
CCNT2-AS1	2	0.02697	0.62837	0.35465	0.11089	0.13487	14669	0.003614	0.140	.....
LOC90784	2	0.07293	0.22078	0.37962	0.41459	0.05295	14669	0.003616	0.140	.....
WDR78	1	0.70629	0.89610	0.60839	0.61638	0.00120	14669	0.003619	0.140	.....
CKMT1A	15	0.06893	0.76124	0.46254	0.50350	0.01698	14669	0.003631	0.140	.....
LOC643542	18	0.28172	0.20080	0.30270	NA	0.02697	11265	0.003633	0.140	...?.
NIN	14	0.71528	0.22977	0.07992	0.05395	0.12488	14669	0.003636	0.140	.....
LOC101929745	5	0.29570	0.92308	0.73726	0.03097	0.05195	14669	0.003647	0.140	.....
LOC257396	5	0.21678	0.48152	0.23177	NA	0.02198	11265	0.003648	0.140	...?.
ZYG11A	1	0.25075	0.36464	0.15584	0.68931	0.01598	14669	0.00365	0.140	.....
KIAA1683	19	0.42058	0.64835	0.04496	0.52448	0.01798	14669	0.003651	0.140	.....
CASP2	7	0.06294	NA	0.56144	0.39760	0.02298	13691	0.003664	0.140	.?...
NXT1	20	0.25075	0.11489	0.00450	0.29071	0.19980	14669	0.003664	0.140	.....
STAM2	2	0.08991	0.92707	0.12388	0.04595	0.16683	14669	0.003664	0.140	.....
MAPK1IP1L	14	0.56044	0.11588	0.56344	0.04895	0.09590	14669	0.003666	0.140	.....
KAZALD1	10	0.77223	0.20979	0.64036	0.22777	0.01499	14669	0.003667	0.140	.....
KRT6A	12	0.90310	0.98901	0.02797	0.31169	0.01250	14669	0.003683	0.140	.....
LINC00668	18	0.94006	NA	0.71129	NA	0.00170	10287	0.003685	0.140	.?.?.
TUBGCP4	15	0.17782	0.67632	0.39361	0.34965	0.01998	14669	0.003694	0.140	.....
OR2T8	1	0.33866	0.09590	NA	0.01698	0.20380	13818	0.003701	0.140	..?..
NINJ1	9	0.02098	0.13487	0.53846	0.29271	0.12288	14669	0.003725	0.141	.....
TMEM71	8	0.62537	0.00620	0.55245	0.83816	0.01499	14669	0.003753	0.141	.....
LINC01063	3	0.86513	NA	0.84815	0.00350	0.07992	13691	0.003754	0.141	.?...
ARL3	10	0.58042	0.99301	0.07692	0.83017	0.00300	14669	0.003766	0.141	.....
CA8	8	0.06793	0.64935	0.35964	0.09590	0.10789	14669	0.003767	0.141	.....
TLDC2	20	0.45954	0.02398	0.10689	0.24276	0.12587	14669	0.003785	0.142	.....
ACMSD	2	0.01798	0.69031	0.16983	0.19680	0.13586	14669	0.003796	0.142	.....
SRD5A1	5	0.21079	0.11089	0.29371	NA	0.04496	11265	0.003801	0.142	...?.
KCNAB1-AS2	3	0.04296	0.84316	0.77822	0.03097	0.13487	14669	0.003805	0.142	.....
CECR2	22	0.39161	0.15684	0.09990	0.50450	0.03696	14669	0.003827	0.142	.....
GABRA5	15	0.08891	0.08691	0.60739	0.62238	0.03497	14669	0.003834	0.142	.....
NMNAT3	3	0.10989	0.21279	0.11588	0.96004	0.02398	14669	0.00384	0.142	.....
LOC101930452	12	0.43057	0.05994	0.00360	0.76823	0.07493	14669	0.003844	0.142	.....
GRIN2D	19	0.25774	0.06693	0.11588	0.23277	0.13287	14669	0.003854	0.142	.....
DGCR10	22	0.14785	NA	0.21479	0.11888	0.08092	13691	0.003867	0.142	.?...
STK3	8	0.03596	0.63636	0.58541	NA	0.02797	11265	0.003873	0.142	...?.
SYNRG	17	0.47652	0.88911	0.14785	0.25674	0.01898	14669	0.003896	0.143	.....
HOXB9	17	0.17782	0.68531	0.20480	0.17383	0.05794	14669	0.003897	0.143	.....
ZNF610	19	0.02398	0.66833	0.21179	0.64635	0.03596	14669	0.003904	0.143	.....
MTRNR2L1	17	0.64635	0.95804	0.08092	0.13886	0.03097	14669	0.003922	0.143	.....
KPNA7	7	0.25774	0.47153	0.10490	0.16783	0.08092	14669	0.003934	0.143	.....
HHIPL1	14	0.15285	0.25475	0.05894	0.09391	0.23277	14669	0.003937	0.143	.....
PLA2R1	2	0.03397	0.18282	0.56144	0.99401	0.01898	14669	0.003953	0.144	.....
SLC37A4	11	0.37263	0.09590	0.13487	0.43357	0.05195	14669	0.003958	0.144	.....
OR52K2	11	0.22078	NA	0.25375	0.00105	0.45854	13691	0.003972	0.144	.?...
PTPN14	1	NA	0.16284	0.53447	0.22777	0.02597	13245	0.003978	0.144	?....
SIRPB2	20	0.00102	0.96903	0.66334	0.03497	0.32667	14669	0.003988	0.144	.....
CD59	11	0.06294	0.03297	0.15385	0.82418	0.07892	14669	0.003989	0.144	.....
YBEY	21	0.61838	0.86214	0.01299	0.02797	0.16683	14669	0.003993	0.144	.....
ZRANB3	2	0.03497	0.00290	0.98202	0.22777	0.24176	14669	0.004007	0.144	.....
NPRL3	16	0.50649	0.61039	0.41359	0.02597	0.08591	14669	0.004011	0.144	.....
OR8D4	11	0.87812	0.90809	0.46953	0.34366	0.00340	14669	0.00407	0.146	.....
YY1AP1	1	0.07193	0.28971	0.92807	0.00049	0.64835	14669	0.004076	0.146	.....
FMNL1	17	0.89710	0.85315	0.25574	0.08991	0.02198	14669	0.004082	0.146	.....
CHD8	14	0.41159	0.29670	0.38961	0.15984	0.04795	14669	0.004088	0.146	.....
PRAC2	17	0.12787	NA	0.33566	0.19481	0.05095	13691	0.004095	0.146	.?...
EID2	19	0.61239	0.27373	0.73726	0.09091	0.03796	14669	0.004113	0.146	.....
ATM	11	0.90509	0.45854	0.77522	NA	0.00270	11265	0.004114	0.146	...?.
SULT4A1	22	0.55944	0.82817	0.32767	0.31269	0.00980	14669	0.00412	0.146	.....
HDAC5	17	0.37363	0.27872	0.76324	0.08192	0.05794	14669	0.00412	0.146	.....
PYM1	12	0.04995	NA	0.07592	0.02997	0.35265	13691	0.00413	0.146	.?...
ITGAE	17	0.19081	0.38362	0.40859	0.14885	0.06993	14669	0.004151	0.146	.....
RALA	7	0.55345	0.01598	0.22677	0.39660	0.06593	14669	0.004167	0.146	.....
CDC42EP1	22	0.55644	0.04296	0.08292	0.19680	0.12987	14669	0.004174	0.146	.....
PRR23C	3	0.68631	0.69031	0.57043	0.36264	0.00540	14669	0.004236	0.146	.....
MYOZ2	4	0.20879	0.01698	0.45854	0.16583	0.17383	14669	0.004248	0.146	.....
RNF112	17	0.21479	0.52248	0.42957	0.00550	0.29171	14669	0.004257	0.146	.....
GABRR3	3	0.42757	0.86913	0.33367	0.85415	0.00236	14669	0.004258	0.146	.....

ICOS	2	0.09790	0.33866	0.72128	0.61039	0.01698	14669	0.00426	0.146	.....
HDAC2	6	0.34865	0.60240	0.12488	0.28072	0.03696	14669	0.004269	0.146	.....
TMEM143	19	0.60240	0.84216	0.73926	0.02298	0.04695	14669	0.00427	0.146	.....
SP9	2	0.97502	0.23576	0.81119	0.09091	0.02398	14669	0.004287	0.146	.....
SLU7	5	0.76424	0.04795	0.67932	0.89610	0.00470	14669	0.004292	0.146	.....
ZSCAN2	15	0.04995	0.93506	0.40060	NA	0.02398	11265	0.004292	0.146	...?.
CNIH4	1	0.05095	0.25075	0.75924	0.03696	0.23876	14669	0.004315	0.146	.....
NUCB2	11	0.37463	0.46553	0.36264	0.22577	0.03197	14669	0.004332	0.146	.....
CCDC144NL-AS1	17	0.13986	0.60340	0.20380	0.69830	0.01698	14669	0.004357	0.146	.....
ELL3	15	0.13287	0.59640	0.59640	0.53047	0.01399	14669	0.004358	0.146	.....
MAP1A	15	0.14685	0.49650	0.32967	0.36264	0.03596	14669	0.004359	0.146	.....
YIPF3	6	0.57243	0.42557	0.93706	0.09491	0.02597	14669	0.004362	0.146	.....
NAGA	22	0.05694	0.06094	0.10789	0.46753	0.16084	14669	0.004364	0.146	.....
ECD	10	0.56144	0.57942	0.37562	NA	0.00799	11265	0.00437	0.146	...?.
ERVK13-1	16	0.02298	0.94306	0.21279	0.08591	0.17782	14669	0.00437	0.146	.....
TOMM5	9	0.18082	0.77423	0.70629	0.03896	0.08492	14669	0.004377	0.146	.....
C20orf96	20	0.60539	0.79021	0.31668	0.55644	0.00460	14669	0.004393	0.146	.....
SH3BP5	3	0.11888	0.19880	0.06294	0.64236	0.06593	14669	0.004395	0.146	.....
LOC100506937	7	NA	0.00440	NA	NA	NA	978	0.0044	0.146	?.???
ZMYM1	1	0.76923	0.16983	0.04296	0.05195	0.18581	14669	0.004407	0.146	.....
LOC100130417	20	NA	0.26873	0.24076	NA	0.01698	9841	0.004407	0.146	?..?.
DDX52	17	0.43057	0.89211	0.06593	0.57942	0.01199	14669	0.004409	0.146	.....
RHPN1-AS1	8	0.18881	0.56044	0.01598	0.18382	0.14885	14669	0.004411	0.146	.....
BBS4	15	0.11389	0.18981	0.12587	0.25874	0.13586	14669	0.004427	0.146	.....
KCNK10	14	0.15984	0.94306	0.04296	NA	0.03596	11265	0.004429	0.146	...?.
CD5	11	0.22378	0.03297	0.27173	0.38661	0.09491	14669	0.004431	0.146	.....
LHX4	1	0.03796	0.20280	0.59940	0.83916	0.02597	14669	0.004437	0.146	.....
MRPS31P5	13	0.00410	0.33566	0.24476	0.07193	0.45255	14669	0.004439	0.146	.....
DPT	1	0.40659	0.85814	0.18781	0.00052	0.45554	14669	0.00444	0.146	.....
GREB1	2	0.42557	0.21978	0.31269	0.13487	0.07493	14669	0.004445	0.146	.....
TANK	2	0.00450	0.38661	0.18881	0.42957	0.15984	14669	0.004448	0.146	.....
LOC100505918	1	0.61239	NA	0.51548	0.04096	0.04995	13691	0.00445	0.146	.?...
SPATA25	20	NA	0.13686	0.11289	0.03497	0.21279	13245	0.004453	0.146	?....
ANTXR1	2	NA	0.00670	0.95604	0.05095	0.15584	13245	0.004454	0.146	?....
LOC101928162	12	0.70729	0.63337	0.07992	0.27572	0.02498	14669	0.004458	0.146	.....
OR8J3	11	0.91608	0.34066	0.58841	0.52348	0.00400	14669	0.00446	0.146	.....
AKR1C4	10	0.40260	0.87812	0.45355	0.05295	0.05295	14669	0.004462	0.146	.....
EIF3K	19	0.63936	0.21978	0.57143	0.05295	0.07592	14669	0.004467	0.146	.....
VIP	6	0.27772	0.45455	0.00400	0.67832	0.05495	14669	0.004472	0.146	.....
LOC101927230	17	0.39660	NA	0.01998	0.69730	0.01898	13691	0.004483	0.146	.?...
SLC19A1	21	0.19281	0.26074	0.32667	0.05894	0.17682	14669	0.004506	0.146	.....
IQCJ	3	0.79620	0.11289	0.01080	0.00670	0.56044	14669	0.004508	0.146	.....
GSG2	17	0.38661	NA	0.36464	0.22278	0.02398	13691	0.00451	0.146	.?...
NDUFA6-AS1	22	0.06893	0.15784	0.11788	0.56943	0.08891	14669	0.004521	0.146	.....
LOC101927620	14	0.09790	0.50649	0.55045	NA	0.02498	11265	0.004527	0.146	...?.
SLC35E3	12	0.27872	0.98701	0.08591	0.54845	0.01520	14669	0.004532	0.146	.....
EXOC7	17	0.56244	0.39461	0.51548	0.00730	0.18082	14669	0.004535	0.146	.....
MIRLET7DHG	9	0.45854	0.89710	0.40360	0.05195	0.05195	14669	0.004538	0.146	.....
CBX1	17	0.02498	0.20280	0.28571	0.06094	0.37862	14669	0.004542	0.146	.....
OR5D13	11	NA	0.67532	0.52847	0.29371	0.00899	13245	0.004544	0.146	?....
HSD11B1	1	0.42957	0.47253	0.67832	0.23477	0.01898	14669	0.004545	0.146	.....
MFAP1	15	0.14785	0.98801	0.33067	0.85814	0.00530	14669	0.004571	0.146	.....
HYI	1	0.02897	NA	0.56543	0.53846	0.02797	13691	0.004575	0.146	.?...
RPL37	5	0.33267	0.61738	0.45055	0.32567	0.01798	14669	0.004588	0.146	.....
PFN1	17	0.40759	0.82617	0.47253	0.47652	0.00670	14669	0.004589	0.146	.....
PRPH	12	0.12388	0.03796	0.12388	0.29670	0.20080	14669	0.004597	0.146	.....
PRSS54	16	0.22777	0.03097	0.25674	0.59241	0.06394	14669	0.00461	0.146	.....
CNN2	19	0.01898	0.44256	0.77423	0.01420	0.37662	14669	0.004612	0.146	.....
ZNF134	19	0.92008	0.21179	0.38262	0.00470	0.21978	14669	0.004616	0.146	.....
MCM6	2	0.00180	0.14386	0.71828	0.51948	0.14186	14669	0.004616	0.146	.....
MAP3K19	2	0.00260	0.13986	0.98601	0.85115	0.05495	14669	0.004618	0.146	.....
MRPL18	6	0.47353	0.99401	0.19381	0.03896	0.07892	14669	0.004636	0.146	.....
NUP50	22	0.24076	0.07493	0.27473	0.07393	0.23377	14669	0.004665	0.146	.....
LETM1	4	0.07193	NA	0.39660	0.11489	0.10190	13691	0.004667	0.146	.?...
DNMT3A	2	0.33866	0.00790	0.10490	0.41159	0.15185	14669	0.004667	0.146	.....
SPA17	11	0.11089	NA	0.30769	0.23976	0.05395	13691	0.00467	0.146	.?...
NUDT13	10	0.52747	0.55944	0.40460	NA	0.00899	11265	0.004671	0.146	...?.
TARP	7	0.01499	0.80819	0.21578	0.06593	0.26474	14669	0.004672	0.146	.....
PARTICL	2	0.01798	0.91309	0.02398	0.07892	0.36264	14669	0.00468	0.146	.....

GFRA3	5	0.29371	0.44456	0.47053	0.03197	0.13786	14669	0.004682	0.146	.....
OR5AK2	11	0.98901	0.03197	0.99600	0.01299	0.14685	14669	0.004682	0.146	.....
PNP	14	0.15784	0.00610	0.91409	NA	0.07792	11265	0.004705	0.146	...?.
PRMT6	1	0.20579	NA	0.63137	0.47852	0.01099	13691	0.004707	0.146	.?...
HSCB	22	0.76324	0.68631	0.27273	0.06294	0.04695	14669	0.004707	0.146	.....
SLC33A1	3	0.07692	0.21179	0.38561	0.06993	0.23876	14669	0.00471	0.146	.....
USF2	19	0.10290	0.26474	0.26374	0.23177	0.10889	14669	0.00471	0.146	.....
GRIP2	3	0.15085	0.69231	0.63337	0.00850	0.22677	14669	0.004719	0.146	.....
PGLYRP3	1	0.03996	0.21578	0.08392	0.23477	0.23576	14669	0.004735	0.146	.....
TCP11	6	0.09091	0.03297	0.16384	0.39860	0.17383	14669	0.004736	0.146	.....
SH3GL1	19	0.59141	0.66334	0.21279	0.02997	0.10490	14669	0.004747	0.146	.....
EPB41	1	0.35065	0.05594	0.77722	0.33966	0.04096	14669	0.004757	0.146	.....
CDC25C	5	0.60340	0.43956	0.44555	0.09291	0.04695	14669	0.00476	0.146	.....
BPIFB6	20	0.41059	0.51049	0.33267	0.42757	0.01598	14669	0.004767	0.146	.....
FTCDNL1	2	0.88811	0.64436	0.14386	0.12088	0.03497	14669	0.004792	0.147	.....
LOC101929715	2	0.51748	NA	0.59041	NA	0.00600	10287	0.004793	0.147	.?.?.
DARS2	1	0.34665	0.24775	0.29870	0.09291	0.11389	14669	0.004806	0.147	.....
BORCS5	12	0.34166	0.33367	0.32967	0.00920	0.28971	14669	0.004835	0.148	.....
KDM3B	5	0.37263	0.36364	0.67632	0.00820	0.20779	14669	0.004841	0.148	.....
GPX2	14	0.45754	0.80320	0.83516	0.03996	0.04496	14669	0.004843	0.148	.....
LINC00644	14	0.18282	0.08791	0.78721	0.78422	0.01698	14669	0.004851	0.148	.....
LOC101060385	2	0.03497	0.62238	0.48052	0.00820	0.42557	14669	0.004855	0.148	.....
CCDC84	11	0.21578	0.15584	0.11389	0.73227	0.03996	14669	0.004865	0.148	.....
IL19	1	0.09890	0.28372	0.36464	0.00390	0.57542	14669	0.0049	0.148	.....
LINC00637	14	0.02997	0.50250	0.83117	0.98601	0.00999	14669	0.004907	0.148	.....
RPRM	2	0.06494	NA	0.17982	0.04595	0.24575	13691	0.004912	0.148	.?...
FAM134B	5	0.06993	0.26374	0.50050	NA	0.04995	11265	0.004922	0.148	...?.
MCIDAS	5	0.67033	0.13886	0.52947	NA	0.01499	11265	0.004925	0.148	...?.
DNAL1	14	0.27073	0.27473	0.19381	NA	0.03896	11265	0.004928	0.148	...?.
RMST	12	0.05295	0.16983	0.22378	0.19381	0.20579	14669	0.004932	0.148	.....
VIM-AS1	10	0.00520	0.26374	0.49451	0.03696	0.51449	14669	0.004947	0.148	.....
ZNF541	19	0.60539	0.33766	0.13487	0.06993	0.11389	14669	0.004952	0.148	.....
ASIC3	7	0.49151	0.65834	0.55145	0.03097	0.07892	14669	0.004958	0.148	.....
IFNA6	9	NA	0.04895	NA	0.03297	NA	4382	0.004958	0.148	?.?.?
NBPF18P	1	0.37862	0.18781	0.10689	0.45854	0.04995	14669	0.004975	0.148	.....
PRDM2	1	0.75125	0.61239	0.23776	0.03896	0.07892	14669	0.005001	0.148	.....
LINC01336	5	0.00250	0.58242	0.98002	0.74226	0.03497	14669	0.005002	0.148	.....
RPL21	13	0.90909	0.76124	0.05994	0.03297	0.10090	14669	0.005003	0.148	.....
DLL4	15	0.67333	NA	0.72627	0.24076	0.00899	13691	0.005006	0.148	.?...
LOC644919	14	0.83916	0.12488	0.29670	NA	0.01798	11265	0.005008	0.148	...?.
ZNF30	19	0.40460	0.50949	0.41758	0.22378	0.03097	14669	0.00502	0.148	.....
PTGER4	5	0.17582	0.30969	0.59940	0.34765	0.03696	14669	0.005035	0.148	.....
DGCR5	22	0.28172	0.80919	0.61638	0.16284	0.02897	14669	0.005037	0.148	.....
CENPQ	6	0.21079	0.78122	0.02498	NA	0.04895	11265	0.005055	0.148	...?.
CCT7	2	0.01399	0.84416	0.52048	0.04196	0.24875	14669	0.005056	0.148	.....
C16orf90	16	0.41259	0.55345	0.51149	0.65135	0.00630	14669	0.005063	0.148	.....
LINC01624	6	0.08991	0.82018	0.05095	0.23377	0.11289	14669	0.005072	0.148	.....
C20orf27	20	0.00340	0.64136	0.49151	0.14486	0.22278	14669	0.00508	0.148	.....
COLEC12	18	0.01698	0.26873	NA	0.12787	NA	5806	0.00508	0.148	..?.?
PTPN5	11	0.67532	0.08092	0.62238	0.95504	0.00500	14669	0.005087	0.148	.....
PHLPP2	16	0.34266	0.25874	0.02797	0.07592	0.26174	14669	0.005094	0.148	.....
LOC101927267	12	0.14086	0.02498	0.39660	0.09291	0.31069	14669	0.005103	0.148	.....
RAI1-AS1	17	NA	0.64336	NA	0.00340	NA	4382	0.005105	0.148	?.?.?
BTD	3	0.25874	0.61439	0.01798	0.03696	0.31469	14669	0.005113	0.148	.....
CASC19	8	0.40659	0.35764	0.59940	0.37363	0.01798	14669	0.005114	0.148	.....
BLNK	10	0.79920	0.79021	0.42458	0.10589	0.02298	14669	0.005116	0.148	.....
CDK5	7	0.91508	0.75624	0.47253	0.07592	0.02498	14669	0.00514	0.148	.....
SYCE1	10	0.11489	0.99401	0.33766	0.01598	NA	6657	0.005147	0.148	....?
SIRPA	20	0.35265	0.38861	0.31069	0.67033	0.01299	14669	0.005153	0.148	.....
GTPBP2	6	0.64036	0.23277	0.31668	0.13087	0.06294	14669	0.005159	0.148	.....
ABRACL	6	0.24775	0.40160	0.87313	NA	0.01299	11265	0.005159	0.148	...?.
OR4B1	11	0.46953	0.64835	0.75724	0.24276	0.01399	14669	0.005165	0.148	.....
IGF2BP2	3	0.40360	0.26474	0.60839	0.31069	0.02697	14669	0.005195	0.148	.....
FCAMR	1	0.24875	0.10190	0.59141	0.04096	0.22378	14669	0.005195	0.148	.....
LOC101928435	21	0.10789	0.22278	0.84016	0.31169	0.05095	14669	0.005203	0.148	.....
PHF20	20	0.58941	0.44056	0.21179	0.26973	0.03097	14669	0.005203	0.148	.....
SFTPC	8	0.49950	0.01199	0.27373	0.64436	0.04995	14669	0.005207	0.148	.....
ATXN3	14	0.38861	0.83516	0.74226	0.23177	0.01399	14669	0.005208	0.148	.....
HSF2BP	21	0.13087	0.05594	0.04795	NA	0.17283	11265	0.005213	0.148	...?.

KANK3	19	0.35365	0.36563	0.81019	0.01020	0.18681	14669	0.005213	0.148	.....
CISH	3	0.17682	0.73726	0.77023	0.48651	0.01099	14669	0.005232	0.148	.....
LINC00842	20	NA	NA	0.80220	NA	0.00430	8863	0.005232	0.148	??.?.
GPR19	12	0.57742	0.11788	0.07193	0.08591	0.19680	14669	0.005239	0.148	.....
SZT2	1	0.03297	0.97802	0.56344	0.12288	0.09391	14669	0.00524	0.148	.....
JAKMIP1	4	0.92308	0.18382	0.35465	0.03097	0.11788	14669	0.005252	0.148	.....
WDR34	9	0.63237	0.11089	0.45654	0.13187	0.07293	14669	0.005252	0.148	.....
SLC22A3	6	0.02298	0.25475	0.26773	0.36563	0.14186	14669	0.005257	0.148	.....
TANC1	2	0.00430	0.52148	0.31668	0.21878	0.22278	14669	0.005275	0.148	.....
PRSS38	1	0.00830	0.06494	0.88112	0.86813	0.06294	14669	0.005281	0.148	.....
CCDC39	3	0.98601	0.12188	0.14186	0.01170	0.26973	14669	0.00529	0.149	.....
SGCZ	8	0.00799	0.10290	0.78621	NA	NA	3253	0.005305	0.149	...??
IL18R1	2	0.65435	0.05495	0.04496	0.04895	0.32967	14669	0.005321	0.149	.....
C17orf77	17	0.15385	0.47053	0.52847	0.69630	0.01399	14669	0.005323	0.149	.....
SRPK1	6	0.58342	0.77023	0.04595	0.02797	0.17582	14669	0.005334	0.149	.....
TMX2	11	0.12388	0.93906	0.54645	0.07892	0.07992	14669	0.005353	0.149	.....
CCNI2	5	0.05295	0.96204	0.97502	0.31768	0.02298	14669	0.005359	0.149	.....
LOC285593	5	0.01798	0.55045	0.01798	NA	0.18581	11265	0.005382	0.150	...?.
SLAIN2	4	0.75824	0.21778	0.03996	0.72927	0.02098	14669	0.005409	0.150	.....
ZFYVE9	1	0.01499	0.22378	0.13986	0.10889	0.43157	14669	0.005415	0.150	.....
IL1RL2	2	0.84316	0.17982	0.54545	0.12887	0.04296	14669	0.005418	0.150	.....
ZPR1	11	0.75524	0.60939	0.40959	0.17083	0.02198	14669	0.00543	0.150	.....
LOC339666	22	0.14286	0.66833	0.12488	0.35864	0.05794	14669	0.005431	0.150	.....
LOC101927179	4	0.24076	0.55944	0.07792	0.15385	0.11888	14669	0.005431	0.150	.....
AES	19	0.53646	0.38162	0.11788	0.13087	0.08891	14669	0.005434	0.150	.....
ECHDC2	1	0.57642	0.22577	0.14186	0.98601	0.00899	14669	0.00544	0.150	.....
ANKRD39	2	0.14585	0.91309	0.10789	0.11389	0.11988	14669	0.005443	0.150	.....
LOC103191607	13	0.01998	0.57642	0.31469	0.01299	0.52348	14669	0.005444	0.150	.....
IL1RL1	2	0.65734	0.18382	0.03397	0.04196	0.27473	14669	0.00545	0.150	.....
FAM171A2	17	0.14186	NA	0.21978	0.07892	0.14286	13691	0.005493	0.150	.?...
RMDN1	8	0.07093	0.26474	0.47952	0.11389	0.17882	14669	0.005502	0.150	.....
CLASP1	2	0.98202	0.06094	0.34066	0.02498	0.18681	14669	0.005505	0.150	.....
EIF4EBP1	8	0.06094	0.92507	0.35964	0.05894	0.16084	14669	0.005522	0.150	.....
LOC101928517	19	0.01299	0.75325	0.31868	0.45654	0.07393	14669	0.005525	0.150	.....
ACSS2	20	0.04096	0.42358	0.40559	0.10889	0.19481	14669	0.005533	0.150	.....
ZFPM2-AS1	8	0.20579	0.42158	0.07592	0.31169	0.08891	14669	0.005541	0.150	.....
NPAT	11	0.83117	0.84016	0.74725	NA	0.00250	11265	0.005545	0.150	...?.
CDKN2A	9	0.79221	0.56743	0.29570	0.06294	0.05894	14669	0.005551	0.150	.....
CHGB	20	0.11389	0.70430	0.53846	0.00430	0.37263	14669	0.005552	0.150	.....
KHNYN	14	0.80919	0.54845	0.07393	0.04795	0.11688	14669	0.005568	0.150	.....
SMTN	22	0.37063	0.15784	0.68132	0.43956	0.02498	14669	0.005577	0.150	.....
ROCK2	2	0.83816	0.32567	0.93506	0.23077	0.01199	14669	0.005585	0.150	.....
LOC100505984	2	0.64935	0.14685	0.15684	0.64735	0.02198	14669	0.005596	0.150	.....
S100A16	1	0.09291	0.92408	0.71728	0.23876	0.03397	14669	0.005606	0.150	.....
SULT1C2	2	0.02897	0.43556	0.77323	0.04995	0.23676	14669	0.005607	0.150	.....
GALNT8	12	0.30669	0.23576	0.31369	0.68332	0.02098	14669	0.005609	0.150	.....
SPHK2	19	NA	0.58342	0.44755	0.03796	0.07692	13245	0.005617	0.150	?....
FLJ46906	6	0.37962	0.06993	0.10090	NA	0.08092	11265	0.005619	0.150	...?.
POLDIP3	22	0.07193	0.74825	0.52547	0.13986	0.08791	14669	0.005625	0.150	.....
MPP4	2	NA	0.17982	0.14386	0.15385	0.09191	13245	0.005627	0.150	?....
MYO15A	17	0.45155	0.68531	0.69830	0.01099	0.13287	14669	0.005635	0.150	.....
FGB	4	0.54346	0.00380	0.14086	0.44855	0.13287	14669	0.005656	0.150	.....
LINC01068	13	0.75624	0.58042	0.55245	0.01698	0.09491	14669	0.005663	0.150	.....
BLOC1S2	10	0.20779	0.05994	0.31069	0.06993	0.29670	14669	0.005663	0.150	.....
LELP1	1	0.80120	NA	0.11788	0.31568	0.01898	13691	0.005676	0.150	.?...
NAPA-AS1	19	0.31169	NA	0.25874	0.13487	0.06494	13691	0.005687	0.150	.?...
EFCAB6-AS1	22	0.77622	0.78422	0.78621	0.02797	0.04496	14669	0.005691	0.150	.....
HCN1	5	0.12188	0.15285	0.95704	NA	0.03497	11265	0.005702	0.150	...?.
NIP7	16	0.14885	0.12288	0.60040	0.65734	0.03297	14669	0.00571	0.150	.....
TMEM136	11	0.10589	0.12687	0.91708	0.82418	0.01898	14669	0.005714	0.150	.....
LINC00634	22	0.28871	0.00180	0.50949	0.39560	0.15185	14669	0.005715	0.150	.....
LEF1-AS1	4	0.19281	0.38861	0.40759	0.14985	0.09291	14669	0.005722	0.150	.....
EPHA2	1	0.25075	0.71628	0.05495	0.76623	0.02098	14669	0.005724	0.150	.....
SEC23A	14	0.86114	0.18082	0.33167	NA	0.01560	11265	0.005733	0.150	...?.
LOC401127	4	0.35365	0.36963	0.51149	0.05095	0.12188	14669	0.005739	0.150	.....
OAS3	12	0.46254	0.39161	0.01598	0.46454	0.06194	14669	0.005739	0.150	.....
ZC3H7A	16	0.47353	0.07992	0.10889	NA	0.06593	11265	0.00574	0.150	...?.
ZBTB44	11	0.05994	0.38062	0.05694	0.14685	0.28372	14669	0.005742	0.150	.....
CCNI	4	0.26374	0.46454	0.45954	0.08591	0.09890	14669	0.005744	0.150	.....

KCNK15	20	0.74426	NA	0.23976	0.14186	0.03297	13691	0.005744	0.150	.?...
MRGPRX1	11	0.01998	NA	0.21179	0.49850	0.07493	13691	0.005752	0.150	.?...
ALPK1	4	0.63736	0.29670	0.13287	0.07692	0.12587	14669	0.005753	0.150	.....
ZFP36L2	2	0.00190	NA	0.99101	0.42358	0.07592	13691	0.005761	0.150	.?...
TIE1	1	0.07493	0.21379	0.82817	0.47652	0.04396	14669	0.005782	0.150	.....
HTR5A	7	0.80519	0.08292	0.74126	0.14985	0.04795	14669	0.005789	0.150	.....
PMS2P9	7	0.36663	NA	0.84715	NA	0.00730	10287	0.005797	0.150	.?.?.
NUP50-AS1	22	0.37662	0.04995	0.24575	0.07992	0.24775	14669	0.005798	0.150	.....
LOC105370306	13	0.01598	0.61439	0.15984	NA	0.10490	11265	0.005799	0.150	...?.
R3HDM1	2	0.00660	0.17483	0.96104	0.24975	0.17782	14669	0.005802	0.150	.....
HLA-H	6	0.10090	0.37862	0.96503	NA	0.02398	11265	0.005805	0.150	...?.
OR10P1	12	0.81818	0.96004	0.33566	0.00170	0.20879	14669	0.005832	0.150	.....
KDELC2	11	0.36663	0.97103	0.64735	NA	0.00640	11265	0.005836	0.150	...?.
C1S	12	0.10889	0.77023	0.54645	0.43057	0.02498	14669	0.005839	0.150	.....
CDK2AP1	12	0.19780	0.03297	0.47153	NA	0.08092	11265	0.00585	0.150	...?.
PARP14	3	0.68931	0.09590	0.45355	0.27672	0.04296	14669	0.005871	0.150	.....
RAB35	12	0.85315	0.69131	0.03097	NA	0.01898	11265	0.005884	0.150	...?.
LOC101927157	4	0.21479	0.47253	0.52448	0.12288	0.08192	14669	0.005884	0.150	.....
TJAP1	6	0.79620	0.25175	0.68631	0.08691	0.04895	14669	0.005896	0.150	.....
MCOLN3	1	0.05195	0.77123	0.03896	0.11389	0.27073	14669	0.005897	0.150	.....
PRELID3B	20	0.81818	0.33866	0.66533	0.01698	0.11089	14669	0.005942	0.150	.....
HPGD	4	0.70729	0.06194	0.71029	0.27273	0.03796	14669	0.00595	0.150	.....
CHAF1B	21	0.36364	0.14985	0.64436	0.18382	0.06893	14669	0.00595	0.150	.....
OTOF	2	0.31768	0.22977	0.14286	0.45055	0.05594	14669	0.005951	0.150	.....
ANKIB1	7	0.04595	0.94206	0.36264	0.05694	0.18781	14669	0.005954	0.150	.....
SEPN1	1	0.46553	0.31269	0.52448	0.13287	0.06094	14669	0.005955	0.150	.....
ZNF397	18	0.16883	0.40160	0.09990	0.14286	0.17483	14669	0.005956	0.150	.....
NBPF7	1	0.02198	0.61339	0.18382	0.30170	0.14086	14669	0.005957	0.150	.....
OR51V1	11	0.23177	0.20080	0.69530	0.08192	0.13187	14669	0.005981	0.150	.....
OR5D16	11	0.05495	0.51149	0.04895	0.30270	0.17183	14669	0.005995	0.150	.....
MMP27	11	0.67932	0.03896	0.24675	0.61838	0.03397	14669	0.005997	0.150	.....
PCBP4	3	0.16384	0.57642	0.89311	0.00800	0.24575	14669	0.006036	0.150	.....
TRIM8	10	0.36863	0.98801	0.54945	0.78521	0.00310	14669	0.006046	0.150	.....
RUNX1	21	0.08192	0.08492	0.84815	0.51149	0.05894	14669	0.00605	0.150	.....
FTSJ3	17	0.90609	NA	0.16384	0.07393	0.05495	13691	0.006061	0.150	.?...
JMJD1C	10	0.13287	0.53846	0.09391	NA	0.06294	11265	0.006064	0.150	...?.
PGLYRP4	1	0.21179	0.63836	0.16384	0.15285	0.10190	14669	0.006083	0.150	.....
FAM149B1	10	0.20380	0.68032	0.45055	NA	0.01998	11265	0.006091	0.150	...?.
EZR	6	0.35465	0.23676	0.49151	0.00260	0.47253	14669	0.006122	0.150	.....
ELF3	1	0.07592	0.37363	0.18881	0.07892	0.28172	14669	0.006122	0.150	.....
SMNDC1	10	0.12687	0.67832	0.27073	0.26773	0.06593	14669	0.006127	0.150	.....
ATF5	19	0.20480	0.24076	0.42158	0.03896	0.24775	14669	0.006138	0.150	.....
C6orf58	6	0.45155	0.07393	0.07892	0.84316	0.03896	14669	0.006138	0.150	.....
NDUFA7	19	0.41059	0.43656	0.75225	0.02298	0.13287	14669	0.006156	0.150	.....
DEFB115	20	0.05694	NA	0.13287	0.58841	0.05295	13691	0.006163	0.150	.?...
ELF1	13	0.94805	0.88112	0.68531	NA	0.00240	11265	0.006167	0.150	...?.
LOC100128398	19	0.56344	0.51049	0.54046	0.01698	0.13986	14669	0.006175	0.150	.....
XRCC3	14	0.03696	0.12687	0.48252	0.61339	0.08292	14669	0.006178	0.150	.....
OR4C16	11	0.04096	0.72627	0.66633	0.44855	0.03696	14669	0.006183	0.150	.....
SNTB1	8	0.38861	0.77722	0.91209	NA	0.00600	11265	0.006189	0.150	...?.
TRUB1	10	0.23077	0.36064	0.49950	0.31568	0.04496	14669	0.006196	0.150	.....
LOC101929199	4	0.40360	0.21778	0.83317	0.11289	0.06893	14669	0.006198	0.150	.....
RAB1A	2	0.26374	0.64535	0.01099	0.78521	0.03896	14669	0.006206	0.150	.....
PLEKHB2	2	0.13287	0.40060	0.41558	0.21778	0.08791	14669	0.006214	0.150	.....
PCBP3	21	0.02398	0.01698	0.15285	0.07293	0.77822	14669	0.006218	0.150	.....
IFI27	14	0.87612	0.13187	0.11089	NA	0.03297	11265	0.006219	0.150	...?.
TP53I13	17	0.51049	0.98102	0.47552	0.77822	0.00270	14669	0.006222	0.150	.....
ASAH1	8	0.07992	0.26773	0.02797	NA	0.16084	11265	0.006223	0.150	...?.
UBE2R2	9	0.14286	0.30070	0.90809	NA	0.02597	11265	0.00623	0.150	...?.
ESRRG	1	0.79121	0.04895	0.00380	NA	NA	3253	0.006235	0.150	...??
TRIM56	7	0.23676	0.40360	0.27972	0.41359	0.04296	14669	0.006249	0.150	.....
RDM1	17	0.27373	0.09790	0.39760	0.39161	0.06793	14669	0.006259	0.150	.....
PURG	8	0.50849	0.46454	0.12288	0.38661	0.03596	14669	0.006266	0.150	.....
PDE10A	6	0.65634	0.62637	0.70629	0.29570	0.01120	14669	0.006269	0.150	.....
TRIM31	6	0.82817	0.38861	1.00000	0.49750	0.00420	14669	0.006281	0.150	..0..
C10orf126	10	0.18282	0.56543	0.05794	NA	0.06294	11265	0.006286	0.150	...?.
LOC102724301	11	0.58242	0.03497	0.79520	0.14386	0.09091	14669	0.006298	0.150	.....
LHFPL5	6	0.41359	0.94106	0.03097	0.00999	0.34565	14669	0.006306	0.150	.....
EMX1	2	0.50749	NA	0.61738	0.19880	0.02198	13691	0.006307	0.150	.?...

TXNRD3	3	0.32368	0.56643	0.05295	0.92507	0.01698	14669	0.006312	0.150	.....
BCAR4	16	0.21079	0.95405	0.59640	0.00830	0.20979	14669	0.006319	0.150	.....
LINC00332	13	0.26174	0.18781	0.88511	NA	0.02398	11265	0.006321	0.150	...?.
ACTR1B	2	0.83417	0.67832	0.81918	0.13387	0.01598	14669	0.006332	0.150	.....
MIR5095	16	0.90010	0.20180	0.06593	0.15784	0.09391	14669	0.006334	0.150	.....
LINC00703	10	0.50350	0.35365	0.11688	0.02498	0.26474	14669	0.006338	0.150	.....
TGM7	15	0.23277	0.42358	0.26773	0.53447	0.03197	14669	0.006339	0.150	.....
SBSN	19	0.71828	0.39860	0.50649	0.20180	0.02797	14669	0.006344	0.150	.....
RASAL2	1	0.04396	0.12687	0.47253	0.05694	0.40360	14669	0.006349	0.150	.....
RAB32	6	0.25275	0.52947	0.09690	0.05295	0.23477	14669	0.006353	0.150	.....
SPATA4	4	0.14685	0.46653	0.16783	0.03197	0.32767	14669	0.006357	0.150	.....
NOL7	6	0.30170	0.70130	0.23876	0.26673	0.04396	14669	0.006364	0.150	.....
TRIML1	4	0.66334	0.31469	0.26374	0.47552	0.01998	14669	0.006378	0.150	.....
LOC100294145	6	0.57642	0.16384	0.38661	0.65035	0.01680	14669	0.006381	0.150	.....
ADAD2	16	0.19980	0.99900	0.60440	0.33067	0.01898	14669	0.006393	0.150	.....
DXO	6	0.55944	0.61339	0.71828	0.18781	0.02198	14669	0.006398	0.150	.....
DDX23	12	0.09391	0.85315	0.41459	0.20879	0.06693	14669	0.006416	0.150	.....
GS1-124K5.11	7	0.77323	0.13087	0.46753	NA	0.01898	11265	0.006417	0.150	...?.
NT5C3B	17	0.42358	0.96104	0.84316	0.01698	0.08691	14669	0.006436	0.150	.....
CYP4A11	1	0.63337	0.46354	0.83816	0.17283	0.02298	14669	0.006446	0.150	.....
POU6F2-AS1	7	0.70030	0.47453	0.89311	0.41359	0.00699	14669	0.006447	0.150	.....
CNTD2	19	0.71429	0.00440	0.83317	0.02198	0.32967	14669	0.006448	0.150	.....
HSH2D	19	0.43656	0.18881	0.04795	0.56743	0.05994	14669	0.006452	0.150	.....
CEP41	7	0.12887	0.43656	0.18681	0.23177	0.11788	14669	0.006456	0.150	.....
WTAP	6	0.12088	0.91409	0.27073	0.07992	0.13586	14669	0.006486	0.150	.....
IGFALS	16	0.71728	0.18581	0.84416	0.00610	0.22378	14669	0.00649	0.150	.....
TTC25	17	0.88511	0.53447	0.45055	0.00040	0.39461	14669	0.006496	0.150	.....
RASSF10	11	0.35764	0.20280	0.96404	0.25774	0.03596	14669	0.006498	0.150	.....
GSPT1	16	0.64635	0.38861	0.40160	0.04895	0.10290	14669	0.006499	0.150	.....
IGSF10	3	0.12288	0.57842	0.62537	0.48851	0.02597	14669	0.006508	0.150	.....
LOC440982	3	0.23077	0.54346	0.80619	0.06593	0.09191	14669	0.006513	0.150	.....
ITGA6	2	0.47053	0.55644	0.15784	0.29171	0.04296	14669	0.006514	0.150	.....
ATF6B	6	0.42957	0.48851	0.24476	0.53946	0.01998	14669	0.006523	0.150	.....
SOX14	3	0.46354	0.29371	0.18681	0.05495	0.18581	14669	0.006531	0.150	.....
DCDC5	11	0.41459	0.17283	0.81119	NA	0.01998	11265	0.006536	0.150	...?.
P3H2-AS1	3	0.52947	0.27872	0.10689	0.19481	0.09590	14669	0.006547	0.150	.....
KRTAP25-1	21	0.71229	0.20180	0.94705	NA	0.00930	11265	0.00655	0.150	...?.
CISD1	10	0.00899	0.05295	0.31369	0.03896	0.82018	14669	0.00655	0.150	.....
TRIM51	11	0.63536	0.93806	0.11089	0.28971	0.02498	14669	0.006556	0.150	.....
CYP2D6	22	0.46054	0.26374	0.11588	0.11089	0.15485	14669	0.006597	0.150	.....
TFCP2L1	2	0.48152	0.51149	0.40460	0.01798	0.18182	14669	0.006606	0.150	.....
C16orf78	16	0.68132	0.48651	0.03097	0.13487	0.11389	14669	0.006609	0.150	.....
DPPA4	3	0.63736	0.83417	0.07992	0.17283	0.05195	14669	0.006612	0.150	.....
KIF11	10	0.17183	0.29870	0.08991	0.56344	0.07093	14669	0.006618	0.150	.....
ABT1	6	0.42358	0.57542	0.00560	0.34765	0.10390	14669	0.006618	0.150	.....
SLC1A7	1	0.01399	0.65934	0.62637	0.05994	0.27173	14669	0.006621	0.150	.....
RGS14	5	0.25375	0.17882	0.99001	0.49451	0.02298	14669	0.006622	0.150	.....
ACLY	17	0.52448	0.69331	0.96404	0.00140	0.24176	14669	0.006626	0.150	.....
DNAJC9	10	0.13986	0.76424	0.47153	NA	0.02398	11265	0.006627	0.150	...?.
REPS1	6	0.30470	0.11988	0.50250	NA	0.04296	11265	0.006637	0.150	...?.
NEURL1-AS1	10	0.21179	0.98901	0.50649	0.11189	0.05894	14669	0.006643	0.150	.....
F2RL2	5	0.55145	0.12188	0.02398	NA	0.09091	11265	0.006651	0.150	...?.
LOC105372288	19	0.84216	0.53247	0.22777	0.11888	0.04995	14669	0.006652	0.150	.....
NUB1	7	0.61239	0.38561	0.82817	0.23377	0.02098	14669	0.006653	0.150	.....
EIF2B1	12	0.86114	0.00600	0.27173	0.33067	0.09990	14669	0.006661	0.150	.....
ZNF317	19	0.20280	0.18581	0.19281	0.02198	0.45055	14669	0.006663	0.150	.....
LOC100129316	9	0.15485	0.30569	0.07493	0.28771	0.14985	14669	0.006666	0.150	.....
GRINA	8	0.77922	0.55045	0.46753	0.03097	0.08891	14669	0.006667	0.150	.....
DBX2	12	0.42258	0.21479	0.44755	NA	0.02797	11265	0.006672	0.150	...?.
C4orf17	4	0.75125	0.75924	0.53946	0.11888	0.02697	14669	0.006695	0.150	.....
TLR10	4	0.54745	0.41558	0.52647	0.49950	0.01299	14669	0.0067	0.150	.....
SWT1	1	0.04795	0.29770	0.87413	0.15285	0.13686	14669	0.006702	0.150	.....
GK5	3	0.33067	0.29071	0.40360	0.41558	0.03696	14669	0.006708	0.150	.....
P4HA2-AS1	5	0.33666	0.09990	0.06294	0.81219	0.05395	14669	0.00671	0.150	.....
ATP8B1	18	0.73427	0.12288	0.15185	NA	0.03896	11265	0.006722	0.150	...?.
BDH1	3	0.14585	0.06194	0.54446	0.06194	0.32368	14669	0.006733	0.150	.....
RINT1	7	0.01798	0.17582	0.12787	0.88811	0.10789	14669	0.006741	0.150	.....
SYNJ2-IT1	6	0.16184	NA	0.41858	0.07093	0.13087	13691	0.006749	0.150	.?...
LOC100506178	7	0.40859	0.11888	0.02198	NA	0.11588	11265	0.006751	0.150	...?.

NFE2L3	7	0.26074	0.22677	0.05095	0.45455	0.09990	14669	0.006751	0.150	.....
ENPEP	4	0.20480	0.01798	0.18182	0.01399	0.79021	14669	0.006767	0.150	.....
DEFB122	20	0.10390	0.49650	0.70130	0.41658	0.03696	14669	0.006774	0.150	.....
LINC01611	6	0.70430	0.07393	0.01798	0.83117	0.04895	14669	0.00678	0.150	.....
LOC102723344	15	0.06394	0.36963	0.61538	NA	0.05095	11265	0.006784	0.150	...?.
EML2	19	0.49451	0.45255	0.57542	0.04396	0.10589	14669	0.006787	0.150	.....
HYPK	15	0.20779	0.76623	NA	0.81818	0.00560	13818	0.006788	0.150	..?..
COG8	16	0.10789	0.04096	0.57942	0.57343	0.08492	14669	0.006796	0.150	.....
CLPP	19	0.68232	NA	0.12388	0.06593	0.09590	13691	0.006804	0.150	.?...
FAM179B	14	0.81518	0.26274	0.12787	0.07892	0.12587	14669	0.006805	0.150	.....
LOC105375545	7	0.06094	0.70629	0.84615	0.42557	0.03097	14669	0.006806	0.150	.....
CTD-2151A2.1	5	0.08591	0.15684	0.08292	0.37962	0.19181	14669	0.006807	0.150	.....
SLC22A18	11	0.21578	0.92208	0.74925	0.07293	0.06693	14669	0.006814	0.150	.....
C1orf194	1	0.14286	0.68232	0.56144	0.26174	0.04795	14669	0.006843	0.150	.....
ARHGEF26-AS1	3	0.18581	0.78322	0.31469	0.70829	0.01499	14669	0.00686	0.150	.....
SOCS2	12	0.88511	0.03397	0.40559	NA	0.03297	11265	0.006868	0.150	...?.
KCNJ14	19	0.14985	NA	0.87013	0.00310	0.34466	13691	0.006874	0.150	.?...
RFWD2	1	0.65235	0.97203	0.30470	0.01499	0.12488	14669	0.006878	0.150	.....
NDFIP2	13	0.76024	0.62338	0.39660	0.28472	0.01720	14669	0.00689	0.150	.....
DGKA	12	0.03596	NA	0.19381	0.07592	0.28571	13691	0.006892	0.150	.?...
C17orf67	17	0.06893	0.85514	0.27772	0.31668	0.06893	14669	0.006895	0.150	.....
DENND5A	11	0.64535	0.33167	0.56743	NA	0.01299	11265	0.006896	0.150	...?.
ITK	5	0.65734	0.45155	0.51149	0.27073	0.02298	14669	0.006898	0.150	.....
NEB	2	0.28771	0.75824	0.98601	0.03397	0.08691	14669	0.006901	0.150	.....
MTX2	2	0.09091	0.12288	0.11588	0.31868	0.21479	14669	0.006914	0.150	.....
ZNRF3	22	0.35165	0.19580	0.41958	0.18182	0.09391	14669	0.006938	0.150	.....
DAPP1	4	0.15485	0.33866	0.02997	0.17483	0.25974	14669	0.006942	0.150	.....
ARF6	14	0.56044	0.02098	0.15984	0.31269	0.13786	14669	0.006956	0.150	.....
OR5D18	11	0.18082	0.71728	0.13786	0.03796	0.26074	14669	0.006961	0.150	.....
PHB2	12	0.22977	0.40859	0.44256	0.04795	0.18781	14669	0.006963	0.150	.....
NPY5R	4	0.70330	0.42757	0.00520	0.02997	0.37463	14669	0.006969	0.150	.....
P4HA1	10	0.52647	0.53147	0.38861	NA	0.01499	11265	0.006972	0.150	...?.
C9orf92	9	0.57742	0.16084	0.27473	0.27572	0.06294	14669	0.006972	0.150	.....
BAAT	9	0.09491	0.30470	0.54446	0.28671	0.08991	14669	0.006978	0.150	.....
RAPGEF6	5	0.05195	0.09391	0.01299	0.83716	0.19281	14669	0.006978	0.150	.....
LINC00311	16	0.07892	NA	0.98801	0.75325	0.00999	13691	0.006979	0.150	.?...
PON1	7	0.13087	0.77023	0.99401	NA	0.01399	11265	0.006979	0.150	...?.
RHPN1	8	0.10889	0.58941	0.02198	0.17183	0.25075	14669	0.006984	0.150	.....
OR2G3	1	0.73427	0.18382	0.30669	0.33067	0.03796	14669	0.006987	0.150	.....
ZBTB40	1	0.05994	0.09091	0.01399	0.19980	0.52947	14669	0.006998	0.150	.....
PRNT	20	0.92707	0.04196	0.01898	0.60739	0.07193	14669	0.007014	0.150	.....
ASAP3	1	0.15285	0.00540	0.50150	0.30869	NA	6657	0.007022	0.150	....?
DCLK3	3	0.00560	0.57043	0.24675	0.71429	0.09590	14669	0.007027	0.150	.....
SERINC4	15	0.46054	0.91508	0.39461	0.36763	0.01499	14669	0.007052	0.151	.....
NIM1K	5	0.16484	0.75924	0.90010	0.05195	0.09990	14669	0.007052	0.151	.....
SKIV2L2	5	0.10889	0.62937	0.94406	NA	0.01998	11265	0.007066	0.151	...?.
LOC105370457	14	0.85614	0.59740	0.94705	NA	0.00360	11265	0.00707	0.151	...?.
IQCG	3	0.30070	0.93506	0.14685	0.00340	0.42557	14669	0.007086	0.151	.....
LINC00174	7	0.83816	0.21578	0.76424	NA	0.00999	11265	0.007095	0.151	...?.
SMCO1	3	0.12587	0.89011	0.71029	0.49850	0.01698	14669	0.007098	0.151	.....
PEX2	8	0.11389	0.61838	0.34366	0.17882	0.10589	14669	0.007105	0.151	.....
AHI1	6	0.35964	0.55245	0.99800	0.20779	0.02597	14669	0.007107	0.151	.....
NTS	12	0.84615	0.92208	0.13886	0.78122	0.00420	14669	0.007109	0.151	.....
GTF3C3	2	0.52947	0.29071	0.18382	0.10290	0.13087	14669	0.007124	0.151	.....
FRA10AC1	10	0.53147	0.04296	0.42657	NA	0.04995	11265	0.00713	0.151	...?.
GSTK1	7	NA	NA	0.25075	0.12388	0.05095	12267	0.007134	0.151	??...
LOC100996583	1	0.12987	0.12188	0.77822	0.12188	0.17383	14669	0.007156	0.151	.....
ANAPC1	2	0.35165	0.96104	0.32468	0.86114	0.00520	14669	0.007156	0.151	.....
ZCCHC14	16	0.30270	0.95704	0.25674	NA	0.01798	11265	0.007159	0.151	...?.
ZBTB8OS	1	0.01998	0.60240	0.28971	0.95904	0.03596	14669	0.007162	0.151	.....
VASH2	1	0.45355	0.13287	0.42358	0.58841	0.02897	14669	0.007167	0.151	.....
LOC105376306	9	0.05095	0.02298	0.94206	NA	0.12188	11265	0.007177	0.151	...?.
EP400NL	12	0.81119	0.08292	0.88511	NA	0.01499	11265	0.007205	0.151	...?.
BMP3	4	0.52348	0.05694	0.05295	0.00370	0.85814	14669	0.007212	0.151	.....
EYA4	6	NA	0.81518	0.38362	NA	0.00900	9841	0.007214	0.151	?..?.
MYH2	17	0.78621	0.78422	0.47652	0.04795	0.05694	14669	0.007223	0.151	.....
PAUPAR	11	0.27872	0.06793	0.83916	NA	0.04296	11265	0.007235	0.151	...?.
JAML	11	0.16883	0.16384	0.15584	NA	0.09790	11265	0.007246	0.151	...?.
XIRP1	3	0.13986	0.23477	0.03896	0.32767	0.19880	14669	0.007248	0.151	.....

MTMR14	3	0.72927	0.49950	0.59341	0.04895	0.07293	14669	0.007261	0.151	.....
ACOT2	14	0.06993	0.24575	0.77223	NA	0.05495	11265	0.007262	0.151	...?.
RGS13	1	0.67033	0.22078	0.02498	0.01399	0.48851	14669	0.007263	0.151	.....
CDC37	19	0.00160	0.43856	0.25974	0.29471	0.32667	14669	0.007266	0.151	.....
KCNN2	5	0.71828	0.51249	0.11688	NA	0.02198	11265	0.007272	0.151	...?.
ZNF622	5	0.09491	0.00860	0.74625	0.37263	0.18581	14669	0.007274	0.151	.....
LOC149950	20	0.52647	0.36464	0.39760	0.23377	0.04595	14669	0.00728	0.151	.....
PEF1	1	0.29570	NA	0.00019	0.10989	0.41858	13691	0.007283	0.151	.?...
HIST2H2BF	1	0.03297	NA	0.82218	0.03097	0.25874	13691	0.007286	0.151	.?...
KMT2C	7	0.77323	0.11089	0.29570	0.54046	0.02697	14669	0.0073	0.151	.....
S100A10	1	0.49351	0.93706	0.25275	0.43556	0.01499	14669	0.007303	0.151	.....
PLAUR	19	0.79720	0.62837	0.00450	0.76124	0.02597	14669	0.007336	0.151	.....
STK11	20	NA	0.62637	0.64635	0.70230	0.00400	13245	0.007342	0.151	?....
ZYG11B	1	0.20679	0.61638	0.14985	0.87213	0.01898	14669	0.007344	0.151	.....
PDZD3	11	0.10390	0.40859	0.82517	0.42857	0.03896	14669	0.007352	0.151	.....
MUC4	3	0.91708	0.14186	0.58242	0.00550	0.27972	14669	0.007354	0.151	.....
LOC101927418	14	0.88212	0.03097	0.08991	0.17782	0.16783	14669	0.007359	0.151	.....
BAIAP2-AS1	17	0.41758	0.36164	0.05395	NA	0.05894	11265	0.007374	0.151	...?.
FAM160B1	10	0.10190	0.71728	0.44655	0.64236	0.02398	14669	0.007393	0.151	.....
TRAPPC4	11	0.18382	0.08691	0.13387	0.90110	0.05794	14669	0.007398	0.151	.....
SDCBP	8	0.76424	0.62038	0.73327	0.08691	0.03596	14669	0.007404	0.151	.....
LOC100505817	18	0.29570	0.24575	0.16583	0.64436	0.04296	14669	0.007416	0.151	.....
TFAP2A	6	0.85215	0.71928	0.77522	0.14985	0.01698	14669	0.007417	0.151	.....
S100B	21	0.22877	0.07792	0.01998	0.70729	0.13487	14669	0.00744	0.151	.....
EMB	5	0.34266	0.33167	0.98501	0.02797	0.14885	14669	0.007442	0.151	.....
NWD1	19	0.09990	0.63536	0.66733	0.10190	0.11988	14669	0.007451	0.151	.....
OR4X1	11	0.39061	0.72128	0.99500	0.10490	0.03796	14669	0.007451	0.151	.....
LINC00473	6	0.15085	0.55844	0.80420	0.01399	0.25974	14669	0.007469	0.151	.....
TTC19	17	0.77123	0.19081	0.27173	0.08891	0.11588	14669	0.007482	0.151	.....
ZNF774	15	0.54845	0.17083	0.64735	NA	0.02198	11265	0.007484	0.151	...?.
DDN	12	0.92208	NA	0.24875	0.01998	0.11888	13691	0.007534	0.151	.?...
ABHD12B	14	0.24276	0.65035	0.47552	0.07193	0.11888	14669	0.007534	0.151	.....
TMC3	15	0.07992	0.15185	0.42158	0.10490	0.29071	14669	0.007558	0.151	.....
DYRK3	1	0.80020	0.57243	0.19181	0.71129	0.00820	14669	0.007559	0.151	.....
RRP7BP	22	0.02298	0.51249	0.15784	0.35465	0.17283	14669	0.007561	0.151	.....
TRIM9	14	0.43157	0.46054	0.00330	0.03297	0.49850	14669	0.007567	0.151	.....
OTP	5	0.46653	0.26873	0.09590	0.01499	0.42857	14669	0.007575	0.151	.....
URAHP	16	0.57842	0.16384	0.89510	0.21079	0.04196	14669	0.007576	0.151	.....
DTWD1	15	0.33766	0.35564	0.03796	0.00200	0.84515	14669	0.007584	0.151	.....
ZNF649	19	0.72428	0.44755	0.14885	0.03497	0.17582	14669	0.007585	0.151	.....
RABL6	9	0.26074	0.91409	0.13287	0.04196	0.19880	14669	0.007592	0.151	.....
PER3	1	0.55345	0.01199	0.22777	0.00390	0.83417	14669	0.007609	0.151	.....
LYPD6B	2	0.10689	0.87812	0.37562	0.08492	0.14286	14669	0.007627	0.151	.....
ZSWIM5	1	0.66533	0.88911	0.47752	0.67932	0.00400	14669	0.007628	0.151	.....
KLHL17	1	0.25375	0.23876	0.50649	NA	0.03996	11265	0.007634	0.151	...?.
PPP1R13B	14	0.04296	0.66933	0.88112	0.87712	0.01399	14669	0.007637	0.151	.....
SCN9A	2	0.83816	0.05694	0.48651	0.20180	0.07193	14669	0.007638	0.151	.....
LRRN4CL	11	0.55844	0.46254	0.16184	0.23377	0.06094	14669	0.007642	0.151	.....
CRNKL1	20	0.05495	0.28272	0.69630	0.28072	0.11089	14669	0.007648	0.151	.....
SLC27A6	5	0.35065	0.46054	0.13287	0.05594	0.22178	14669	0.007651	0.151	.....
AGPAT3	21	0.06993	0.24975	0.57243	NA	0.06993	11265	0.007651	0.151	...?.
SGPP1	14	0.39760	0.55744	0.82917	NA	0.01140	11265	0.007659	0.151	...?.
ZNF470	19	0.25874	0.29770	0.42058	0.04496	0.23077	14669	0.007669	0.151	.....
CEP170B	14	0.76324	0.42058	0.57243	0.21778	0.02697	14669	0.007672	0.151	.....
FMO6P	1	0.01199	0.09491	0.30070	0.32468	0.32468	14669	0.007684	0.151	.....
LOC100506548	5	0.48851	0.63037	0.33966	0.49151	0.01698	14669	0.007685	0.151	.....
FAM131B	7	0.00380	0.15085	0.11189	0.13387	0.69730	14669	0.007694	0.151	.....
LINC01593	2	0.36863	0.23576	0.51948	0.02797	0.24176	14669	0.007694	0.151	.....
SNRPF	12	0.90110	0.09690	0.10789	0.03397	0.28571	14669	0.007698	0.151	.....
KCNK5	6	0.14885	0.46853	0.21778	NA	0.05794	11265	0.007706	0.151	...?.
SCG2	20	NA	0.23576	0.76823	0.27073	0.02797	13245	0.007711	0.151	?....
ZNF229	19	0.72927	0.11489	0.37163	0.15285	0.09191	14669	0.007714	0.151	.....
LRP10	14	0.95305	0.07093	0.10090	0.33566	0.07393	14669	0.007715	0.151	.....
KRTAP5-4	11	0.34366	0.05395	0.44955	NA	0.06593	11265	0.00774	0.151	...?.
LOC101928767	14	0.16384	0.22677	0.38761	NA	0.06194	11265	0.007747	0.151	...?.
IQGAP3	1	0.26374	0.35165	0.36464	0.14985	0.11588	14669	0.00775	0.151	.....
LINC01070	13	0.16084	0.26374	0.45554	0.65435	0.03696	14669	0.007753	0.151	.....
SAA2-SAA4	11	0.14785	0.53347	0.76823	0.01998	0.24575	14669	0.007784	0.151	.....
C6orf52	6	0.49750	0.61439	0.93407	0.02597	0.09690	14669	0.007794	0.151	.....

VDR	12	0.36563	0.26673	0.15684	0.38761	0.07093	14669	0.007803	0.151	.....
OR2F1	7	0.09091	0.54246	0.39461	0.33566	0.07692	14669	0.007812	0.151	.....
LOC101928068	1	0.28272	NA	0.08092	0.91708	0.01698	13691	0.007824	0.151	.?...
NEAT1	11	0.63836	0.97902	0.64036	0.08292	0.03497	14669	0.007825	0.151	.....
SH3BP2	4	0.19580	0.25874	0.58142	0.07393	0.18881	14669	0.007826	0.151	.....
ADGRL3	4	0.09890	0.67233	0.83516	NA	0.02498	11265	0.007834	0.151	...?.
HSD11B1L	19	0.67932	0.34466	0.36464	0.46953	0.01998	14669	0.007835	0.151	.....
MROH9	1	0.01499	0.99401	0.90909	0.27373	0.06893	14669	0.00784	0.151	.....
ATP11B	3	0.34166	0.64735	0.66833	0.17582	0.04396	14669	0.007849	0.151	.....
OR51B2	11	0.08392	0.20280	0.63137	0.19880	0.15285	14669	0.00785	0.151	.....
CLRN2	4	0.41459	0.71129	0.47552	0.21678	0.03696	14669	0.007852	0.151	.....
CBX3	7	0.70430	0.12987	0.03497	0.63536	0.05594	14669	0.007853	0.151	.....
CIC	19	0.03696	NA	0.92108	0.18382	0.08991	13691	0.007871	0.151	.?...
CPA1	7	0.51548	0.11489	0.51149	0.15984	0.10090	14669	0.007875	0.151	.....
GJB2	13	0.59041	NA	0.00230	0.00510	0.66933	13691	0.007888	0.151	.?...
FIG4	6	0.31069	0.29670	0.38262	0.14685	0.11588	14669	0.007897	0.151	.....
TSPO2	6	0.56743	0.28971	0.51548	NA	0.01998	11265	0.007902	0.151	...?.
PHLDA2	11	0.27672	NA	0.11289	NA	0.04196	10287	0.007915	0.151	.?.?.
LOC100996579	2	0.08791	NA	0.00630	0.31269	0.22877	13691	0.007918	0.151	.?...
OOSP1	11	0.42258	0.42657	0.00550	NA	0.10390	11265	0.007956	0.151	...?.
PLCB4	20	0.03996	0.38062	0.56743	NA	0.07393	11265	0.007979	0.151	...?.
LOC100996437	7	0.87712	0.10490	0.65035	NA	0.01798	11265	0.007985	0.151	...?.
SREBF1	17	0.27972	0.54446	0.32268	0.04995	0.18681	14669	0.008015	0.151	.....
PLK4	4	0.10290	0.03397	0.45255	0.47752	0.14685	14669	0.008027	0.151	.....
SLC43A3	11	0.93706	0.12787	0.02697	0.78222	0.03397	14669	0.008031	0.151	.....
LOC102724874	8	0.74725	0.95305	0.83716	0.57842	0.00270	14669	0.008051	0.151	.....
CIDEA	18	0.52847	0.47952	0.00530	0.78721	0.04695	14669	0.008077	0.151	.....
TPTE2	13	0.78222	0.96404	0.77822	0.28671	0.00770	14669	0.008089	0.151	.....
SLC16A9	10	0.03896	0.21778	0.40060	0.23876	0.21479	14669	0.008091	0.151	.....
HBB	11	0.14585	0.02797	0.58042	0.31668	0.17083	14669	0.008092	0.151	.....
OLFML2B	1	0.36264	0.30569	0.27473	0.12288	0.13886	14669	0.008092	0.151	.....
PCGF3	4	0.24875	0.46553	0.43856	0.16583	0.09191	14669	0.008109	0.151	.....
FMN2	1	NA	0.77423	0.10490	0.03796	0.15485	13245	0.008109	0.151	?....
RALYL	8	0.14186	0.40859	0.82118	NA	0.03097	11265	0.008112	0.151	...?.
OPA1	3	0.04595	0.98202	0.90310	0.09590	0.10789	14669	0.008115	0.151	.....
BPIFB4	20	0.58142	0.38162	0.31269	0.41558	0.02897	14669	0.008121	0.151	.....
TIMM50	19	0.31668	0.18182	0.16484	0.03497	0.38661	14669	0.008122	0.151	.....
BRF1	14	0.10689	0.61738	0.67832	0.86913	0.01299	14669	0.008133	0.151	.....
GSR	8	0.45554	0.91409	0.22478	0.03097	0.15385	14669	0.008146	0.151	.....
CD300LD	17	0.22777	0.30569	0.68831	0.71628	0.01898	14669	0.008152	0.151	.....
CST6	11	0.23576	0.41558	0.12488	NA	0.06394	11265	0.008154	0.151	...?.
LOC100507639	4	0.94705	0.41359	0.76424	0.51349	0.00610	14669	0.008156	0.151	.....
SPOPL	2	0.31768	0.79421	0.74625	0.09091	0.06294	14669	0.00816	0.151	.....
TSSC1	2	0.28272	0.17582	0.11389	0.35165	0.12587	14669	0.008172	0.151	.....
AARS2	6	0.92108	0.16983	0.21778	0.27473	0.05195	14669	0.008176	0.151	.....
LOC100507195	12	0.38561	0.37562	0.75425	0.41858	0.02298	14669	0.008179	0.151	.....
E2F6	2	0.59141	0.73626	0.11888	0.12388	0.08492	14669	0.008185	0.151	.....
EIF4E	4	0.55445	0.63636	0.45754	0.00880	0.22478	14669	0.008185	0.151	.....
SEPSECS	4	0.52947	0.42158	0.53347	0.14286	0.06094	14669	0.008189	0.151	.....
ABRA	8	0.96803	0.31668	0.24775	0.07692	0.09291	14669	0.008211	0.151	.....
SERAC1	6	0.18581	0.39960	0.52947	0.08292	0.16484	14669	0.008217	0.151	.....
ZNF808	19	0.64136	0.28372	0.15784	0.01698	0.32268	14669	0.00822	0.151	.....
CCDC107	9	0.12887	NA	0.39161	NA	0.03796	10287	0.008221	0.151	.?.?.
MIR4500HG	13	0.11588	0.10989	0.90709	NA	0.06094	11265	0.008229	0.151	...?.
DZIP1L	3	0.28971	0.60539	0.11788	0.00350	0.59241	14669	0.008237	0.151	.....
FAM175A	4	0.23277	0.31668	0.08791	0.20080	0.18282	14669	0.008239	0.151	.....
GEMIN4	17	0.42657	0.59441	0.07293	0.74525	0.02398	14669	0.008241	0.151	.....
NFAM1	22	0.57942	0.47253	0.07692	0.00580	0.47453	14669	0.008248	0.151	.....
NFE2L1	17	0.03696	0.19281	0.37862	0.06793	0.44555	14669	0.008255	0.151	.....
KCNA2	1	0.13886	0.06294	0.77223	0.38561	0.10090	14669	0.008257	0.151	.....
CCL23	17	0.44655	0.60639	0.23277	0.15485	0.07892	14669	0.008257	0.151	.....
AQR	15	0.24076	0.58242	0.60440	0.61139	0.01698	14669	0.008262	0.151	.....
LINC00346	13	0.28172	0.91608	0.82817	0.33766	0.01698	14669	0.008269	0.151	.....
CAP2	6	0.92308	0.48152	0.24376	0.46753	0.01499	14669	0.008279	0.151	.....
ARHGEF26	3	0.17582	0.86713	0.20480	0.62537	0.02697	14669	0.008284	0.151	.....
ZNF34	8	0.48851	0.21878	0.75824	NA	0.02098	11265	0.008291	0.151	...?.
LINC01011	6	0.77922	0.01950	0.06094	NA	0.11289	11265	0.008291	0.151	...?.
FAM157C	16	0.41159	0.21878	NA	0.02398	NA	5806	0.008292	0.151	..?.?
LINC00593	15	0.33666	0.40160	0.23377	NA	0.04096	11265	0.008297	0.151	...?.

LARGE-AS1	22	0.38262	0.02797	0.66434	0.08492	0.24176	14669	0.008314	0.151	.....
DPPA2	3	0.76224	0.93606	0.36164	0.02797	0.09191	14669	0.008316	0.151	.....
SMIM23	5	0.09491	0.76224	0.15485	0.70929	0.03996	14669	0.00832	0.151	.....
MMRN1	4	0.42458	0.32667	0.39860	0.18182	0.08092	14669	0.008328	0.151	.....
ACTR10	14	0.50350	0.53447	0.93307	0.22777	0.02398	14669	0.008328	0.151	.....
OSTN	3	0.24675	0.47453	0.29970	0.42857	0.04795	14669	0.008331	0.151	.....
ZNF385D	3	NA	0.73926	NA	NA	0.00740	8990	0.008334	0.151	?.??.
CLCNKB	1	0.32767	0.03497	0.25375	0.97103	0.04296	14669	0.008345	0.151	.....
GALK1	17	0.29371	0.28971	0.03796	0.51449	0.09790	14669	0.008349	0.151	.....
XPO5	6	0.87512	0.32068	0.56743	0.16084	0.03996	14669	0.008371	0.151	.....
CNEP1R1	16	0.63437	0.34965	0.10789	NA	0.03796	11265	0.008371	0.151	...?.
GGT5	22	0.07992	0.60639	0.42058	0.05794	0.24975	14669	0.008373	0.151	.....
KRBA2	17	0.58741	0.37463	0.91808	0.52048	0.00999	14669	0.008376	0.151	.....
ZNF701	19	0.29670	0.95904	0.68531	0.00600	0.23576	14669	0.008391	0.151	.....
DSEL	18	0.22877	0.14785	0.65634	0.41858	0.05994	14669	0.008394	0.151	.....
THRB-AS1	3	0.22378	0.22677	0.03796	0.64635	0.09790	14669	0.008408	0.151	.....
ARHGAP32	11	0.23277	0.49850	0.18382	NA	0.05095	11265	0.008411	0.151	...?.
PPM1B	2	0.29970	0.55944	0.14885	0.76324	0.02398	14669	0.008413	0.151	.....
RHEBL1	12	0.46753	NA	0.02997	0.15984	0.12987	13691	0.008415	0.151	.?...
AZGP1P1	7	0.11389	0.92607	0.24875	0.12388	0.13686	14669	0.008415	0.151	.....
EFEMP1	2	0.46853	0.26673	0.28871	0.80619	0.01798	14669	0.008416	0.151	.....
RBM39	20	0.51748	0.80220	0.44156	0.44655	0.01399	14669	0.008422	0.151	.....
SAXO2	15	0.30470	0.29371	0.18781	0.72727	0.03497	14669	0.008423	0.151	.....
CCNT2	2	0.01898	0.35964	0.67333	0.28571	0.15485	14669	0.008423	0.151	.....
C3orf84	3	0.54545	0.38062	0.64835	0.09391	0.07792	14669	0.008429	0.151	.....
MCOLN2	1	0.00799	0.76723	0.05894	0.34765	0.26474	14669	0.008429	0.151	.....
AEBP2	12	0.57842	0.82817	0.21479	NA	0.01598	11265	0.00843	0.151	...?.
CLP1	11	0.48651	0.10490	0.84316	0.08991	0.12488	14669	0.008432	0.151	.....
TTYH3	7	0.52348	0.83616	0.14585	NA	0.02098	11265	0.008433	0.151	...?.
UNG	12	0.21778	0.43556	0.02098	0.32068	0.16983	14669	0.008433	0.151	.....
XKR3	22	0.57443	0.80719	0.16284	0.21978	0.04695	14669	0.008451	0.151	.....
PAFAH1B3	19	0.04595	NA	0.86014	0.24975	0.07293	13691	0.008455	0.151	.?...
RAI14	5	0.94505	0.60140	0.22977	0.23377	0.02897	14669	0.008459	0.151	.....
LINC01473	2	0.85015	0.60839	0.38561	0.01399	0.15584	14669	0.008467	0.151	.....
XPA	9	0.19281	0.13986	0.98501	0.12787	0.13387	14669	0.008469	0.151	.....
SLC4A1	17	0.15285	0.65135	0.19281	0.85415	0.02398	14669	0.00847	0.151	.....
TARDBP	1	0.46054	0.38961	0.45055	0.23377	0.05395	14669	0.008495	0.152	.....
ZNF639	3	0.01199	0.94505	0.74126	0.02498	0.36364	14669	0.008502	0.152	.....
LINC00202-1	10	0.04396	0.13586	0.20180	NA	0.18282	11265	0.00854	0.152	...?.
C1orf228	1	0.07992	0.63337	0.59241	0.26573	0.07992	14669	0.008588	0.153	.....
FAM129B	9	0.10689	0.67333	0.38362	NA	0.04496	11265	0.008591	0.153	...?.
C14orf80	14	0.78621	NA	0.09790	0.25175	0.04196	13691	0.008601	0.153	.?...
HMHB1	5	0.12887	0.54046	0.00510	0.86214	0.09391	14669	0.008603	0.153	.....
ARL6	3	0.67732	0.50150	0.06693	0.18082	0.09391	14669	0.00861	0.153	.....
SLC25A45	11	0.45355	0.46553	0.18282	0.54745	0.03097	14669	0.008612	0.153	.....
CHAC1	15	0.19780	NA	0.09491	0.17283	0.14486	13691	0.008621	0.153	.?...
PCM1	8	0.04396	0.94406	0.17582	NA	0.07193	11265	0.008623	0.153	...?.
S100A13	1	0.11389	0.94905	0.97602	0.64236	0.01070	14669	0.008631	0.153	.....
LOC646471	1	0.97502	NA	0.01698	NA	0.02897	10287	0.008651	0.153	.?.?.
LINC01206	3	0.71029	0.36264	0.35564	0.19281	0.05395	14669	0.008666	0.153	.....
CEMIP	15	0.44256	0.03796	0.40859	NA	0.07293	11265	0.008691	0.153	...?.
BPI	20	0.26174	0.59840	0.16284	0.57742	0.03796	14669	0.008693	0.153	.....
SBNO1	12	0.11788	0.48651	0.80619	0.19780	0.08392	14669	0.008697	0.153	.....
KMO	1	0.12488	0.04496	0.19281	0.10190	0.47652	14669	0.0087	0.153	.....
LOC650226	7	0.07592	0.42557	0.03796	0.06094	0.53247	14669	0.008725	0.153	.....
NYAP1	7	0.46354	0.69131	0.06294	0.10689	0.14885	14669	0.008731	0.153	.....
PAK1IP1	6	0.98102	0.59940	0.70330	0.04695	0.05295	14669	0.008738	0.153	.....
TMEM40	3	0.19980	0.48851	0.46653	0.10789	0.13886	14669	0.008742	0.153	.....
MYH16	7	0.49550	0.20480	0.15385	0.32168	0.08791	14669	0.008746	0.153	.....
CDK19	6	0.75125	0.11289	0.02498	0.29670	0.14785	14669	0.008786	0.153	.....
ALOX12P2	17	0.26873	0.25475	0.44855	NA	0.04595	11265	0.008787	0.153	...?.
SOCS4	14	0.49051	0.16683	0.88511	0.30370	0.03996	14669	0.008789	0.153	.....
ITGB6	2	0.16783	0.32567	0.77522	0.57043	0.03097	14669	0.008791	0.153	.....
LOC494141	11	0.52847	0.65335	0.99800	0.00720	0.17083	14669	0.0088	0.153	.....
DEFB118	20	0.03796	NA	0.80420	0.87512	0.01798	13691	0.008808	0.153	.?...
NRBF2	10	0.19880	0.47752	0.19780	NA	0.05794	11265	0.008813	0.153	...?.
EFCAB2	1	0.44156	0.22677	0.69530	0.97902	0.00799	14669	0.008816	0.153	.....
FOXA3	19	0.41159	0.30070	0.50450	0.90809	0.01099	14669	0.008823	0.153	.....
VNN3	6	0.03197	0.63037	0.11189	0.17782	0.28272	14669	0.008833	0.153	.....

SOGA3	6	0.24376	0.30969	0.43157	0.37962	0.06194	14669	0.008851	0.153	.....
ZNF354C	5	0.14486	0.77223	0.34166	0.12587	0.12887	14669	0.008854	0.153	.....
OR1E2	17	0.00015	0.62038	0.41658	0.20879	0.49251	14669	0.008859	0.153	.....
LUZP2	11	NA	0.36464	0.05495	0.07193	NA	5233	0.008863	0.153	?...?
MARK4	19	0.10889	0.09391	0.86314	0.35564	0.10490	14669	0.008872	0.153	.....
PCDH15	10	0.03197	0.19580	0.33966	NA	NA	3253	0.008878	0.153	...??
SYT14	1	0.83017	0.81618	0.14885	0.21279	0.03696	14669	0.008883	0.153	.....
AIF1L	9	0.88412	0.00170	0.33067	0.47852	0.10889	14669	0.00889	0.153	.....
ASL	7	0.39960	0.27273	0.76424	0.02298	0.22178	14669	0.008891	0.153	.....
OR6K3	1	0.44655	0.66134	0.74825	0.03596	0.11089	14669	0.008894	0.153	.....
USP53	4	0.11389	0.82617	0.19481	0.12488	0.16983	14669	0.008914	0.154	.....
MCM3AP	21	0.67632	0.92208	0.01698	0.03097	0.25275	14669	0.008927	0.154	.....
CTTN	11	0.97403	0.25475	0.28372	0.04496	0.14186	14669	0.008952	0.154	.....
OR6V1	7	0.53147	0.03097	0.89810	0.97602	0.01399	14669	0.008958	0.154	.....
SPOCD1	1	0.56444	0.95504	0.12687	0.06194	0.11888	14669	0.00896	0.154	.....
E2F2	1	0.16484	0.01299	0.62737	0.74426	0.08691	14669	0.008964	0.154	.....
LINC00458	13	0.48152	0.24575	0.88511	0.00300	0.39461	14669	0.008986	0.154	.....
ZNF80	3	0.70529	0.04995	0.06793	0.03397	0.47552	14669	0.008988	0.154	.....
GLRA1	5	0.91209	0.16284	0.76424	0.27972	0.02797	14669	0.009004	0.154	.....
QDPR	4	0.24176	0.60040	0.09191	0.03297	0.35864	14669	0.009012	0.154	.....
APOL6	22	0.91409	0.56643	0.11289	0.28571	0.03796	14669	0.009015	0.154	.....
BTBD18	11	0.26573	0.65834	0.39960	0.10589	0.11389	14669	0.009024	0.154	.....
RPS6KA2-IT1	6	0.48851	0.26573	0.87113	0.69431	0.01099	14669	0.009028	0.154	.....
DUSP13	10	0.23576	0.40959	0.86913	0.22278	0.05794	14669	0.009037	0.154	.....
LINC01378	4	0.06094	0.75724	0.01399	0.12987	0.39760	14669	0.009044	0.154	.....
NEK3	13	0.03696	0.72627	0.72428	0.00031	0.94905	14669	0.009053	0.154	.....
PPP2R2C	4	0.33866	0.08791	0.04496	0.05794	0.52048	14669	0.009054	0.154	.....
IGLL3P	20	NA	NA	0.18282	0.12687	0.07193	12267	0.009071	0.154	??...
ASTN1	1	0.83117	0.77722	0.23177	0.23077	0.02997	14669	0.009084	0.154	.....
LOC102724589	10	0.07892	0.99500	0.74525	0.15385	0.07992	14669	0.009096	0.154	.....
FAM110D	1	0.93307	0.83317	0.33067	0.00096	0.34166	14669	0.009101	0.154	.....
ERCC1	19	0.10689	0.70729	0.60040	0.00550	0.46054	14669	0.009101	0.154	.....
LINC00639	14	0.36364	0.22977	0.20280	NA	0.06094	11265	0.009126	0.154	...?.
LRRC73	6	0.60539	0.81618	0.41658	0.48851	0.01199	14669	0.009129	0.154	.....
CD3EAP	19	0.45654	0.91009	0.46553	0.00100	0.42857	14669	0.009132	0.154	.....
ARHGEF18	19	0.76723	0.59441	0.80519	0.18581	0.02298	14669	0.009136	0.154	.....
OR4E2	14	0.24276	0.72627	0.86314	0.01798	0.18881	14669	0.00914	0.154	.....
RBPJ	4	0.17782	0.09391	0.10589	0.11988	0.40559	14669	0.009169	0.154	.....
MNX1	7	0.01099	0.95005	0.85015	0.25375	0.10390	14669	0.009169	0.154	.....
EVC2	4	0.90909	0.77323	0.45654	0.01598	0.11788	14669	0.009172	0.154	.....
TMEFF2	2	0.10190	0.26174	0.84116	0.44655	0.05994	14669	0.009196	0.154	.....
HNRNPA2B1	7	0.61938	0.14585	0.02398	0.87612	0.04895	14669	0.009206	0.154	.....
C11orf91	11	0.43956	0.24076	0.14486	0.91309	0.02498	14669	0.009208	0.154	.....
THBD	20	0.43956	0.27872	0.09590	0.00780	0.58741	14669	0.009208	0.154	.....
VIM	10	0.00790	0.26573	0.54046	0.03497	0.65934	14669	0.009213	0.154	.....
DIDO1	20	0.50849	0.30769	0.05195	0.72328	0.04296	14669	0.009214	0.154	.....
SORCS3	10	0.77722	0.85514	0.80819	0.00120	NA	6657	0.009222	0.154	....?
ITGB7	12	0.62138	0.64236	0.09590	0.15385	0.09291	14669	0.009225	0.154	.....
C12orf57	12	0.20380	0.31868	0.34066	0.36663	0.08192	14669	0.009227	0.154	.....
CEBPB-AS1	20	0.53846	0.00899	0.78821	0.55045	0.06094	14669	0.009229	0.154	.....
F5	1	0.11888	0.33167	0.06593	0.11988	0.36164	14669	0.009245	0.154	.....
MORC2-AS1	22	0.74825	0.34466	0.78222	0.03197	0.11688	14669	0.009252	0.154	.....
FAM213B	1	0.37463	NA	0.72927	0.08192	0.07493	13691	0.009256	0.154	.?...
LOC101927580	16	0.33467	0.24675	0.61439	0.08492	0.15485	14669	0.009259	0.154	.....
SAP130	2	0.71029	0.28272	0.18082	0.77423	0.01798	14669	0.009263	0.154	.....
PDE5A	4	0.02897	0.37562	0.39161	0.09590	0.35465	14669	0.009276	0.154	.....
CLHC1	2	0.27273	0.01099	0.57942	0.04496	0.49650	14669	0.009314	0.154	.....
ANKRD26P3	13	0.17782	0.45754	0.04396	0.04995	0.44955	14669	0.00932	0.154	.....
FAM53C	5	0.66234	0.46953	0.56943	0.00680	0.25774	14669	0.009322	0.154	.....
DIRC2	3	0.86613	0.53846	0.39660	0.46953	0.01299	14669	0.009327	0.154	.....
LOC102724264	10	NA	0.32567	0.37962	0.64136	0.01598	13245	0.009329	0.154	?....
TRIM28	19	0.92807	NA	0.58442	0.00560	0.16883	13691	0.009337	0.154	.?...
ACP7	19	0.97203	0.17483	0.17183	0.11189	0.12188	14669	0.009337	0.154	.....
NRROS	3	0.56444	NA	0.50150	0.30969	0.02298	13691	0.009339	0.154	.?...
ZNF32-AS3	10	0.04396	0.06993	0.94605	0.41059	0.13986	14669	0.00934	0.154	.....
HRAS	11	0.00142	0.11788	0.04296	0.72527	0.46154	14669	0.009354	0.154	.....
TRIM67	1	0.64036	0.10789	0.61838	0.26374	0.06194	14669	0.009359	0.154	.....
FBXO21	12	0.10989	0.78621	0.10789	0.74925	0.04396	14669	0.009373	0.154	.....
SPEM1	17	0.17782	0.39461	0.90310	0.00160	0.58042	14669	0.009383	0.154	.....

LOC101926913	2	0.02997	0.12388	0.43956	0.38761	0.21279	14669	0.009412	0.155	.....
EEA1	12	0.06593	0.32867	0.10190	0.36763	0.20180	14669	0.009419	0.155	.....
ZNF641	12	0.09491	0.26773	0.82717	0.09091	0.21179	14669	0.009423	0.155	.....
CEBPZ	2	0.00910	0.46054	0.37762	0.12488	0.38561	14669	0.009427	0.155	.....
GLO1	6	0.29570	0.99600	0.06494	0.10989	0.15385	14669	0.00944	0.155	.....
EMX2	10	0.01598	0.28372	0.24076	0.94705	0.08991	14669	0.00945	0.155	.....
MRPS18C	4	0.15385	NA	0.08492	0.17582	0.17782	13691	0.009452	0.155	.?...
HNRNPUL2	11	0.66434	0.46354	0.16284	0.39660	0.03896	14669	0.009465	0.155	.....
H2AFZ	4	0.04795	0.12787	0.04595	0.47053	0.30569	14669	0.009468	0.155	.....
ARMC8	3	0.08691	0.41658	0.81718	0.28971	0.08192	14669	0.009472	0.155	.....
TMEM126A	11	0.83916	0.18881	0.91009	NA	0.01260	11265	0.009474	0.155	...?.
FER	5	0.14685	0.00510	0.18382	0.25175	0.46054	14669	0.00952	0.155	.....
GBAS	7	0.82917	0.33467	0.35664	NA	0.01898	11265	0.009522	0.155	...?.
SLC5A12	11	0.12887	0.04895	0.28771	0.29071	NA	6657	0.009522	0.155	....?
CPNE3	8	0.41259	0.13786	0.30070	0.37962	0.08292	14669	0.00953	0.155	.....
SMU1	9	0.18482	0.31668	0.18781	0.40460	0.10390	14669	0.009534	0.155	.....
LINC01298	8	0.00210	0.40260	0.50549	0.13087	0.47353	14669	0.009535	0.155	.....
QTRT2	3	0.87912	0.03197	0.72827	0.15784	0.09690	14669	0.009539	0.155	.....
C8orf86	8	0.30270	0.52947	0.90709	0.58342	0.01399	14669	0.00954	0.155	.....
LINC01578	15	0.04895	0.06593	0.73227	0.41958	0.16284	14669	0.009572	0.155	.....
RRBP1	20	0.89810	0.87313	0.69630	0.39261	0.00670	14669	0.009574	0.155	.....
HSD17B2	16	0.85514	0.20779	0.63836	0.03996	0.13487	14669	0.009601	0.155	.....
NCSTN	1	0.34565	0.04995	0.35764	0.02797	0.49051	14669	0.009603	0.155	.....
CAPN12	19	0.65335	0.00190	0.33267	0.26374	0.23576	14669	0.009605	0.155	.....
F2RL3	19	0.16583	NA	0.10190	0.50450	0.06693	13691	0.009624	0.155	.?...
AKT3	1	0.42158	0.36963	0.09091	0.02298	0.41359	14669	0.00963	0.155	.....
LHX5	12	0.71928	0.54945	0.30669	0.41159	0.02298	14669	0.009633	0.155	.....
RGS8	1	0.16683	0.13686	0.14186	0.77423	0.08292	14669	0.009644	0.155	.....
TMC2	20	0.12587	0.09391	0.81918	0.14685	0.21079	14669	0.009649	0.155	.....
TRPM4	19	0.06494	0.07193	0.00160	NA	0.52647	11265	0.009652	0.155	...?.
CYFIP2	5	0.58442	0.19181	0.22178	0.01898	0.37463	14669	0.009655	0.155	.....
ERVV-2	19	0.09890	0.56843	0.16683	0.71628	0.05495	14669	0.009656	0.155	.....
RBBP4	1	0.07293	0.47053	0.15684	0.84416	0.05694	14669	0.009662	0.155	.....
SHISA6	17	0.39660	0.38861	0.42757	0.58342	0.02597	14669	0.009666	0.155	.....
ACADS	12	0.86414	0.11189	0.18981	0.43357	0.05295	14669	0.009677	0.155	.....
GKN2	2	0.84016	0.44955	0.83017	0.15085	0.03197	14669	0.009685	0.155	.....
KCNE3	11	0.64735	0.93506	0.12488	NA	0.01898	11265	0.009686	0.155	...?.
TSSC4	20	NA	NA	0.15185	0.10290	0.09491	12267	0.009701	0.155	??...
LOC100288637	15	0.08492	0.37163	0.29770	0.20879	0.19281	14669	0.009703	0.155	.....
DECR2	16	0.07193	0.12188	0.52248	0.13686	0.30669	14669	0.009704	0.155	.....
FUT4	11	0.19580	0.01299	0.54945	NA	0.14486	11265	0.009711	0.155	...?.
GPR31	6	0.79421	NA	0.05195	0.04196	0.18981	13691	0.009713	0.155	.?...
UBXN10-AS1	1	0.38462	NA	0.19880	0.06294	0.16683	13691	0.009722	0.155	.?...
INPP4B	4	0.04296	0.10390	0.48851	NA	NA	3253	0.009732	0.155	...??
KANK1	9	0.66234	0.38062	0.02398	NA	0.06593	11265	0.009741	0.155	...?.
GABARAPL3	15	0.76523	0.26873	0.61139	NA	0.01698	11265	0.009743	0.155	...?.
OR52E2	11	0.37862	0.90310	0.84915	0.12587	0.04296	14669	0.009744	0.155	.....
EMILIN2	18	0.57443	0.34366	0.68332	0.97502	0.00550	14669	0.00975	0.155	.....
CCL15-CCL14	17	0.16983	0.46553	0.60440	0.09690	0.15784	14669	0.009755	0.155	.....
ORC1	1	0.00280	NA	0.23077	0.05594	0.63037	13691	0.009768	0.155	.?...
ZNF273	7	0.51449	0.35564	0.66833	NA	0.01998	11265	0.009769	0.155	...?.
CEP68	2	0.22478	0.79820	0.03397	0.66434	0.05495	14669	0.00981	0.155	.....
OR5H15	3	0.31269	0.23876	0.01798	0.10290	0.41059	14669	0.009811	0.155	.....
NRSN1	6	0.05195	0.15784	0.83317	NA	0.09291	11265	0.00982	0.155	...?.
TPTE2P5	13	0.78921	0.84915	0.81718	NA	0.00500	11265	0.009824	0.155	...?.
LINC01273	20	0.89510	0.00599	0.79421	0.13387	0.15684	14669	0.009825	0.155	.....
ACTL7A	9	0.37962	0.08492	0.58242	0.40559	0.07193	14669	0.009833	0.155	.....
PAQR7	1	0.91808	NA	0.19780	0.31568	0.02398	13691	0.009837	0.155	.?...
GPC5-AS2	13	0.31169	0.46753	0.15385	0.06394	NA	6657	0.009851	0.155	....?
SGSM3	22	0.52747	0.29570	0.89411	0.04096	0.13786	14669	0.009876	0.155	.....
LNP1	3	0.47453	0.91109	0.55445	0.07093	0.07493	14669	0.009876	0.155	.....
FAM204A	10	0.67433	0.08092	0.74825	0.08591	0.13886	14669	0.00988	0.155	.....
SNAPIN	1	0.37063	0.27672	0.90310	0.90110	0.00899	14669	0.00989	0.155	.....
C15orf59	15	0.07992	0.37163	0.27373	0.01070	0.67532	14669	0.009909	0.155	.....
CD164	6	0.34765	0.01299	0.98901	0.18182	0.17682	14669	0.009918	0.155	.....
CACNA1A	19	0.07792	0.38861	0.40260	0.77622	0.04695	14669	0.009918	0.155	.....
PGPEP1L	15	0.89211	0.24875	0.66833	NA	0.01399	11265	0.009925	0.155	...?.
NKAIN4	20	0.12088	0.10190	0.66234	0.00230	0.89411	14669	0.009946	0.155	.....
DOHH	19	0.58342	0.10490	0.34865	0.13586	0.15784	14669	0.009951	0.155	.....

KIZ	20	0.30869	0.71029	0.41459	0.01998	0.25175	14669	0.009953	0.155	.....
PRSS48	4	0.85215	0.53746	0.06893	0.04496	0.19580	14669	0.009958	0.155	.....
TMEM245	9	0.75824	0.71229	0.49650	0.04196	0.09291	14669	0.009981	0.155	.....
CYP2B6	19	0.89910	0.77622	0.55844	0.09491	0.03996	14669	0.009982	0.155	.....
TMEM147-AS1	19	0.33167	0.84715	0.20180	0.03996	0.20979	14669	0.009993	0.155	.....
LOC102725254	19	0.35365	0.51748	0.56344	0.29371	0.04595	14669	0.009993	0.155	.....
NAPA	19	0.48352	NA	0.28571	0.14286	0.07692	13691	0.009999	0.155	.?...
PRG2	11	0.52647	0.12388	0.05594	0.60839	0.08492	14669	0.01002	0.155	.....
AEBP1	7	0.98501	0.24575	0.01898	0.36164	0.08691	14669	0.01002	0.155	.....
TRMT10A	4	0.69930	0.56543	0.65634	0.04695	0.09291	14669	0.01003	0.155	.....
TLL2	10	0.98402	0.68931	0.94605	0.00230	0.18482	14669	0.01004	0.155	.....
NSRP1	17	0.84416	0.00210	0.72527	0.13986	0.21179	14669	0.01005	0.155	.....
KRT6B	12	0.98002	0.62537	0.09091	0.15584	0.06793	14669	0.01006	0.155	.....
SEMA6A	5	0.36763	0.73526	0.14186	NA	0.03796	11265	0.01006	0.155	...?.
CDC42EP5	19	0.92108	0.81618	0.52148	0.00290	0.22677	14669	0.01008	0.155	.....
CACNA1H	16	0.07193	0.30270	0.09890	0.29071	NA	6657	0.01008	0.155	....?
PDE6B	4	0.11888	0.14585	0.65235	0.01099	0.60939	14669	0.01009	0.155	.....
CD52	1	0.07393	0.24975	0.29870	0.18581	0.26074	14669	0.01009	0.155	.....
LILRA4	19	0.15784	0.59441	0.26474	0.11688	0.18881	14669	0.01009	0.155	.....
RPL22L1	3	0.23776	0.26074	0.37163	0.44456	0.07193	14669	0.01012	0.155	.....
PPP1R13L	19	0.35664	0.44156	0.33566	0.01020	0.40460	14669	0.01013	0.155	.....
FBXO5	6	0.08591	0.75125	0.70529	0.23776	0.07992	14669	0.01014	0.155	.....
DOK6	18	0.37762	0.01160	0.50350	NA	0.11489	11265	0.01014	0.155	...?.
RPS5	19	0.63237	0.16284	0.31968	0.16084	0.12088	14669	0.01014	0.155	.....
AAMP	20	NA	0.90310	0.12388	0.13487	0.07393	13245	0.01014	0.155	?....
SLC25A4	4	0.05495	0.71928	0.57143	0.18482	0.13487	14669	0.01014	0.155	.....
UBE2H	7	0.61339	0.41159	0.91209	NA	0.01199	11265	0.01015	0.155	...?.
LOC400706	19	0.01099	0.38362	0.76623	0.10290	0.34665	14669	0.01017	0.155	.....
CARD19	9	0.07393	0.93606	0.84016	0.85714	0.01199	14669	0.01018	0.155	.....
LOC101927021	7	0.55045	0.30569	0.05794	NA	0.06893	11265	0.01019	0.155	...?.
IFT140	16	0.08292	0.80719	0.92008	NA	0.02797	11265	0.01019	0.155	...?.
KIAA1456	8	0.59141	0.99500	0.94306	NA	0.00520	11265	0.0102	0.155	...?.
ATP10D	4	0.65934	0.46753	0.22977	0.13886	0.09291	14669	0.0102	0.155	.....
KLC1	14	0.07393	0.14486	0.33267	0.44156	0.16284	14669	0.01021	0.155	.....
GACAT3	2	0.02597	0.51548	0.18881	0.81419	0.08392	14669	0.01021	0.155	.....
RIIAD1	1	0.72927	0.13886	0.44356	0.16384	0.09790	14669	0.01022	0.155	.....
CDKN2B-AS1	9	0.40060	0.66034	0.12987	NA	0.04096	11265	0.01022	0.155	...?.
FXYD6-FXYD2	11	0.61139	0.07393	0.06194	0.24176	0.20579	14669	0.01022	0.155	.....
SSH2	17	0.78022	0.31668	0.65634	0.03696	0.13287	14669	0.01022	0.155	.....
GPBAR1	20	NA	0.95105	0.25574	0.01399	0.19081	13245	0.01023	0.155	?....
EMID1	22	0.03197	0.17383	0.10789	0.22777	0.43357	14669	0.01028	0.156	.....
MTFR1L	1	0.94306	0.55145	0.30769	0.10090	0.06693	14669	0.01029	0.156	.....
TSHB	1	0.87113	0.09291	0.79321	0.02398	0.20779	14669	0.01029	0.156	.....
RRP9	3	0.45654	0.73327	0.75425	0.02298	0.14585	14669	0.0103	0.156	.....
LOC101927809	18	0.52148	0.79321	0.94905	0.07692	0.05295	14669	0.01031	0.156	.....
METTL22	16	0.06194	0.66933	0.60440	0.07992	0.22078	14669	0.01031	0.156	.....
TRIM64C	11	0.36164	0.11888	0.89111	0.32867	0.06294	14669	0.01032	0.156	.....
ADGB	6	0.91309	0.61139	0.53047	0.23876	0.02298	14669	0.01034	0.156	.....
CHD4	12	0.46054	0.09690	0.04995	NA	0.13087	11265	0.01034	0.156	...?.
HOXA11	7	0.10789	0.16583	0.85614	0.56543	0.06094	14669	0.01034	0.156	.....
ARL6IP5	3	0.80320	0.18082	0.22178	0.11988	0.13786	14669	0.01037	0.156	.....
GIT1	17	0.73127	0.34266	0.76224	0.19780	0.03996	14669	0.01037	0.156	.....
SLCO2B1	11	0.43257	0.13686	0.09391	0.87512	0.04895	14669	0.01038	0.156	.....
IRF2BP1	19	0.04396	NA	0.43157	0.26274	0.12987	13691	0.01038	0.156	.?...
BRF2	20	NA	0.53546	0.55844	NA	0.01499	9841	0.01039	0.156	?..?.
GTF2A1	14	0.76224	0.30769	0.12488	NA	0.03996	11265	0.0104	0.156	...?.
OR8A1	11	0.17982	0.01798	0.22278	NA	0.21179	11265	0.0104	0.156	...?.
SART3	12	0.74925	0.33367	0.71528	0.07293	0.08791	14669	0.0104	0.156	.....
TADA2B	4	0.73027	0.36464	0.38661	0.00410	0.39461	14669	0.01041	0.156	.....
PRKAA1	5	0.49451	0.26274	0.57742	0.13287	0.10390	14669	0.01041	0.156	.....
RBM44	20	NA	0.38262	0.20280	0.10689	0.12787	13245	0.01042	0.156	?....
CATSPER3	5	0.55045	0.06993	0.15884	0.08991	0.30969	14669	0.01043	0.156	.....
FBL	19	0.20979	0.20579	0.60639	0.13686	0.17483	14669	0.01044	0.156	.....
CLDN22	4	NA	0.23876	NA	0.02298	NA	4382	0.01045	0.156	?.?.?
LINC01085	4	0.01499	0.67832	0.25574	0.05495	0.50150	14669	0.01046	0.156	.....
LOC101927914	7	0.80220	0.99401	0.00280	0.02098	0.38062	14669	0.01047	0.156	.....
CDK5R1	17	0.24575	NA	0.43856	0.16583	0.08891	13691	0.01047	0.156	.?...
MYBBP1A	17	0.41758	NA	0.47353	0.29371	0.03696	13691	0.01048	0.156	.?...
LCN15	9	0.64535	0.56044	0.46653	0.11688	0.07093	14669	0.01049	0.156	.....

C7orf61	7	0.51049	0.57642	0.05794	0.25874	0.10190	14669	0.0105	0.156	.....
NSFP1	17	0.68631	0.63037	0.02797	0.08492	0.19880	14669	0.0105	0.156	.....
NT5C	17	0.53347	NA	0.89610	0.02198	0.12188	13691	0.0105	0.156	.?...
ATP2C2	16	0.27273	0.64535	0.27872	0.11988	0.14086	14669	0.0105	0.156	.....
IRAIN	15	0.49351	NA	0.43856	0.02997	0.16484	13691	0.0105	0.156	.?...
CA1	8	0.54346	0.33367	0.59041	0.78222	0.01199	14669	0.01051	0.156	.....
LOC101927640	6	0.02797	0.86214	0.10290	0.10490	0.37562	14669	0.01052	0.156	.....
ZNF217	20	0.29970	0.03696	0.50549	0.85614	0.04995	14669	0.01052	0.156	.....
SVIL	10	0.55644	0.06294	0.08891	NA	0.11389	11265	0.01053	0.156	...?.
SLC22A23	6	0.80619	0.23976	0.44256	NA	0.02330	11265	0.01053	0.156	...?.
KLHL11	17	0.32068	0.81319	0.98102	0.01099	0.19580	14669	0.01055	0.156	.....
SLC3A1	2	0.16583	0.18282	0.40559	0.42757	0.10490	14669	0.01057	0.156	.....
ACTL6B	7	0.21079	0.20180	0.49351	0.46553	0.07393	14669	0.01057	0.156	.....
SLAMF8	1	0.15684	NA	0.73427	0.01998	0.26374	13691	0.01058	0.156	.?...
ZNF493	19	0.25674	0.38362	0.25475	0.13087	0.18681	14669	0.01059	0.156	.....
NPAS2	2	0.00970	0.89111	0.66034	0.10789	NA	6657	0.0106	0.156	....?
CRYBB3	22	0.14785	0.56843	0.25275	0.57642	0.05594	14669	0.01061	0.156	.....
RPS28	19	0.79520	0.23477	0.60639	0.08591	0.09990	14669	0.01062	0.156	.....
MTTP	4	0.71129	0.34266	0.58641	0.07992	0.09890	14669	0.01063	0.156	.....
ELP2	18	0.24076	0.73826	0.51648	0.22677	0.06494	14669	0.01064	0.156	.....
NIFK	2	0.96703	0.09491	0.37163	0.06194	0.17782	14669	0.01064	0.156	.....
FAM160A1	4	0.54146	0.31968	0.28072	0.10290	0.15085	14669	0.01064	0.156	.....
PPARA	22	0.75724	0.51548	0.23976	0.01698	0.24675	14669	0.01067	0.156	.....
HPYR1	8	0.38661	NA	0.33167	NA	0.02697	10287	0.01067	0.156	.?.?.
LOC100129027	21	0.02298	0.10889	0.25075	0.29271	0.38162	14669	0.01069	0.156	.....
LINC01489	12	0.36763	0.29271	0.47053	NA	0.04096	11265	0.0107	0.156	...?.
LOC101928414	15	0.07592	0.00330	0.05395	0.53846	0.51648	14669	0.01072	0.157	.....
NOVA1	14	0.00035	0.20879	0.39660	0.50350	0.42757	14669	0.01073	0.157	.....
TEX29	13	0.37463	0.08691	0.97303	0.33966	0.06494	14669	0.01075	0.157	.....
CFAP70	10	0.18881	0.75425	0.44056	NA	0.03497	11265	0.01078	0.157	...?.
DDHD1	14	0.14785	0.37762	0.65734	NA	0.04995	11265	0.01078	0.157	...?.
ZCCHC4	4	0.27972	0.74925	0.05195	0.54346	0.06394	14669	0.01079	0.157	.....
RPL8	8	0.51149	0.80719	0.23576	NA	0.02298	11265	0.01081	0.157	...?.
HCG22	6	0.17682	0.22078	0.08791	NA	0.14186	11265	0.01081	0.157	...?.
MICA	6	0.20679	0.69231	0.16983	NA	0.05694	11265	0.01083	0.157	...?.
MAP3K5	6	0.45654	0.98901	0.34765	0.10190	0.07892	14669	0.01083	0.157	.....
CDC5L	6	0.51948	0.05894	0.22478	0.11788	0.27073	14669	0.01083	0.157	.....
EXPH5	11	0.00280	0.06593	0.16184	NA	0.50749	11265	0.01084	0.157	...?.
LOC100270804	20	0.39960	0.06993	0.16983	0.67632	0.08192	14669	0.01084	0.157	.....
CCDC64	12	0.58541	0.40460	0.00999	NA	0.09690	11265	0.01085	0.157	...?.
NBPF3	1	0.01998	0.00560	0.05794	0.89810	0.42557	14669	0.01085	0.157	.....
TTC23L	5	0.32168	0.67333	0.29471	0.13586	0.11688	14669	0.01086	0.157	.....
NIFK-AS1	2	0.98601	0.09790	0.36464	0.05495	0.18881	14669	0.01087	0.157	.....
CCNG1	5	0.51349	0.42857	0.96903	0.13087	0.05794	14669	0.01088	0.157	.....
CRYBB2	22	0.82517	0.32268	0.71329	0.61838	0.01040	14669	0.01089	0.157	.....
SH2D3A	19	0.39361	0.47952	0.06294	NA	0.07093	11265	0.0109	0.157	...?.
LOC284581	1	0.17283	0.16184	0.11588	0.02797	0.66234	14669	0.0109	0.157	.....
RTKN	2	0.60539	0.05495	0.76723	0.03596	0.27473	14669	0.01092	0.157	.....
GS1-124K5.4	7	0.79421	0.30370	0.72228	NA	0.01499	11265	0.01092	0.157	...?.
NOL4L	20	0.20679	0.12288	0.83516	0.26973	0.11289	14669	0.01092	0.157	.....
IL31RA	5	0.42258	0.15784	0.29670	NA	0.06494	11265	0.01094	0.157	...?.
C1orf50	1	0.35964	0.92308	0.41259	0.03297	0.17183	14669	0.01094	0.157	.....
KCNK15-AS1	20	0.71628	0.93606	0.23077	0.19381	0.04296	14669	0.01094	0.157	.....
LOC101928053	11	0.25674	0.03297	0.36563	NA	0.13387	11265	0.01095	0.157	...?.
GAPT	5	0.58242	0.15984	0.85614	0.01598	0.27572	14669	0.01096	0.157	.....
SENP3	17	0.25774	0.11189	0.94206	0.25275	0.10090	14669	0.01096	0.157	.....
ARFGEF3	6	0.42957	0.93506	0.29870	0.19481	0.05794	14669	0.01098	0.157	.....
AIM2	1	0.92108	0.19081	0.02298	0.90110	0.03397	14669	0.01098	0.157	.....
EQTN	9	0.28172	0.33467	0.74925	NA	0.03397	11265	0.01099	0.157	...?.
LINC01248	2	0.02198	0.38262	0.53946	0.09690	0.37463	14669	0.011	0.157	.....
DNAJC10	2	0.55045	0.92807	0.07293	0.15485	0.09990	14669	0.011	0.157	.....
OCEL1	19	0.91209	0.31369	0.56344	0.31469	0.02797	14669	0.01101	0.157	.....
GNB2	7	0.53247	0.36763	0.62637	0.81319	0.01080	14669	0.01102	0.157	.....
ANTXRLP1	20	NA	0.63237	0.06294	NA	0.04096	9841	0.01102	0.157	?..?.
SLC5A4	22	0.25974	0.19081	0.64935	0.48452	0.05694	14669	0.01102	0.157	.....
NTNG1	1	NA	0.59740	0.01499	0.24376	0.12587	13245	0.01104	0.157	?....
OR6C70	12	0.19980	0.42358	0.93307	0.11489	0.11788	14669	0.01104	0.157	.....
CCL2	17	0.36963	0.43057	0.41459	0.32767	0.05994	14669	0.01104	0.157	.....
FKTN	9	0.38861	0.33666	0.70030	0.08292	0.13786	14669	0.01104	0.157	.....

PRPF4	9	0.29271	0.06593	0.09191	0.07493	0.52048	14669	0.01104	0.157	.....
PPDPF	20	0.02997	NA	0.69331	0.95504	0.02498	13691	0.01104	0.157	.?...
CNOT1	16	0.05794	0.93107	0.47952	0.85315	0.02398	14669	0.01105	0.157	.....
NOL10	2	0.02498	0.21778	0.01898	0.74426	0.27772	14669	0.01105	0.157	.....
COPG1	3	0.14985	0.45155	0.61339	0.08392	0.20180	14669	0.01106	0.157	.....
CASP4	11	0.69530	0.91209	0.85215	0.04496	0.06194	14669	0.01106	0.157	.....
LEXM	1	0.88911	0.05395	0.70030	0.22378	0.07393	14669	0.01107	0.157	.....
COL27A1	9	0.41159	0.45554	0.86214	0.05594	0.12687	14669	0.01111	0.157	.....
B3GNT2	2	0.29071	0.27772	0.55644	0.01998	0.36663	14669	0.01111	0.157	.....
PILRB	7	0.48452	0.74426	0.02597	0.08492	0.23876	14669	0.01112	0.157	.....
NUDCD1	8	0.07792	0.78222	0.24675	NA	0.07293	11265	0.01112	0.157	...?.
SNX11	17	0.04396	0.31868	0.48551	0.05195	0.45754	14669	0.01114	0.157	.....
POMC	2	0.94905	0.00380	0.21379	0.03696	0.52847	14669	0.01117	0.157	.....
HLA-DQB1	6	0.12188	0.39461	0.11089	NA	0.12388	11265	0.01119	0.157	...?.
RSL1D1	16	0.15185	0.70230	0.31269	0.11389	0.18182	14669	0.01119	0.157	.....
CUTA	6	0.23976	0.75025	0.39960	0.00770	0.41558	14669	0.01119	0.157	.....
HOXB13	17	0.05594	0.81518	0.71329	0.43756	0.05495	14669	0.01119	0.157	.....
TMEM101	17	0.60440	0.79321	0.49451	0.08791	0.07393	14669	0.0112	0.157	.....
ANKRD35	1	0.50250	0.64735	0.13786	0.03796	0.24875	14669	0.0112	0.157	.....
DMRTC2	19	0.43556	NA	0.26973	0.00440	0.44156	13691	0.01123	0.157	.?...
RPS6	9	0.09291	0.49650	0.31768	0.11089	0.26274	14669	0.01123	0.157	.....
DDR2	1	0.68032	0.27572	0.78921	0.09690	0.08791	14669	0.01124	0.157	.....
SUCNR1	3	0.91309	0.82218	0.65534	0.11489	0.03297	14669	0.01124	0.157	.....
NWD2	4	0.87213	0.72328	0.45155	0.05794	0.07892	14669	0.01125	0.157	.....
TRA2B	3	0.93207	0.79820	0.42358	0.49351	0.00940	14669	0.01126	0.157	.....
EDEM3	1	0.00999	0.34466	0.27772	0.24975	0.36364	14669	0.01128	0.157	.....
OR5AP2	11	0.66733	0.46853	0.15584	0.27872	0.06793	14669	0.01128	0.157	.....
EXOC5	14	0.26873	NA	0.75924	0.12488	0.08092	13691	0.01129	0.157	.?...
IDO1	8	0.14985	0.99600	0.27872	0.93007	0.01598	14669	0.01129	0.157	.....
CHTOP	1	0.45355	0.42857	0.95804	0.55445	0.01499	14669	0.0113	0.157	.....
CELSR1	22	0.88711	0.26573	0.14885	0.01010	0.40559	14669	0.0113	0.157	.....
FTH1	11	0.78222	0.49351	0.64136	0.27772	0.02697	14669	0.01132	0.157	.....
GORAB	1	0.59141	0.61039	0.21778	0.39361	0.03896	14669	0.01133	0.157	.....
RPS6KL1	14	0.09091	0.14785	0.78921	0.19980	0.19880	14669	0.01134	0.157	.....
TYMSOS	18	0.56044	0.04496	0.79121	0.18981	0.12388	14669	0.01135	0.157	.....
LOC101927062	14	0.68032	0.42857	0.08292	0.00720	0.51049	14669	0.01136	0.157	.....
AQP3	9	0.05495	0.45754	0.47353	0.24176	0.17383	14669	0.01136	0.157	.....
REG3G	2	0.15684	0.18082	0.10090	0.72727	0.10989	14669	0.01136	0.157	.....
GYPB	4	0.12887	0.14186	0.05195	0.05195	0.71029	14669	0.01136	0.157	.....
44261	20	NA	NA	NA	NA	NA	13691	0.01138	0.157	.?...
STBD1	4	0.03996	0.36863	0.65135	0.29371	0.15784	14669	0.01139	0.157	.....
TRADD	16	0.56244	0.84915	0.16484	0.01299	0.29770	14669	0.01139	0.157	.....
RXFP3	5	0.05994	NA	0.67333	0.03097	0.33167	13691	0.0114	0.157	.?...
OBSCN	1	0.04595	0.16384	0.28971	0.56543	0.16883	14669	0.0114	0.157	.....
VOPP1	7	0.07293	0.56344	0.62138	NA	0.05994	11265	0.0114	0.157	...?.
EMG1	12	0.22078	0.45055	0.21479	0.02897	0.41459	14669	0.0114	0.157	.....
GRM6	5	0.09690	0.68432	0.00810	0.64236	0.15684	14669	0.01141	0.157	.....
LOC101928107	21	0.21778	0.85814	0.48252	0.03197	0.22478	14669	0.01142	0.157	.....
LSR	19	0.14186	0.28971	0.19281	0.21978	0.22677	14669	0.01143	0.157	.....
SLC22A11	11	0.19980	0.36963	0.37063	0.20180	0.14885	14669	0.01145	0.157	.....
LOC105369911	12	0.30370	0.10889	0.44755	NA	0.07892	11265	0.01145	0.157	...?.
SCARB2	4	0.07592	0.75325	0.72827	0.00950	0.44755	14669	0.01145	0.157	.....
DERA	12	0.02597	0.66234	0.68531	NA	0.07592	11265	0.01146	0.157	...?.
ANKRD30BP3	10	0.13087	0.28671	0.87113	NA	0.05295	11265	0.01148	0.157	...?.
ILDR2	1	0.01099	0.54545	0.59540	0.01598	0.69431	14669	0.01149	0.157	.....
HSD17B12	11	0.84915	0.48751	0.34466	NA	0.01798	11265	0.0115	0.157	...?.
LOC101928424	11	0.77922	0.09690	0.22677	0.05594	0.27972	14669	0.01151	0.157	.....
STAG3L5P-PVRIG	7	0.48452	0.74326	0.03197	0.09391	0.22278	14669	0.01151	0.157	.....
SOX12	20	0.03796	0.38262	0.65634	0.68831	0.06693	14669	0.01152	0.157	.....
UMAD1	7	NA	0.41359	NA	0.08991	0.07992	12394	0.01152	0.157	?.?..
PPIL6	6	0.47053	0.01090	0.99800	0.22278	0.15285	14669	0.01152	0.157	.....
ACY1	3	0.13487	0.88611	0.99201	0.04895	0.14685	14669	0.01154	0.157	.....
ST3GAL3	1	0.23576	0.99500	0.29271	0.47652	0.03497	14669	0.01154	0.157	.....
THCAT155	12	0.16084	0.34066	0.17283	NA	0.10390	11265	0.01155	0.157	...?.
KLHL42	12	0.44655	0.23077	0.52448	0.17483	0.11289	14669	0.01156	0.157	.....
LOC285804	6	0.39860	0.78322	0.25774	0.19481	0.07992	14669	0.01156	0.157	.....
PITPNM3	17	0.43357	0.20080	0.49750	0.35265	0.06993	14669	0.01157	0.157	.....
HAVCR2	5	0.35664	0.16983	0.73826	0.48751	0.04695	14669	0.01157	0.157	.....
TSPAN10	17	0.03896	0.54845	0.24276	0.15784	0.30070	14669	0.01158	0.157	.....

KRT75	12	0.85614	0.33367	0.11588	0.38162	0.05495	14669	0.01158	0.157	.....
FLOT2	17	0.27872	0.63137	0.75125	0.16983	0.07293	14669	0.01159	0.157	.....
CLDN11	3	0.08591	0.25574	0.08092	0.27473	0.31069	14669	0.01159	0.157	.....
DGCR2	22	0.43257	0.94805	0.10589	0.65335	0.02498	14669	0.01161	0.157	.....
RFK	9	0.26374	0.06294	0.13487	NA	NA	3253	0.01161	0.157	...??
C17orf105	17	0.33966	0.56843	0.19780	0.65035	0.03596	14669	0.01162	0.157	.....
SKIL	3	0.25874	0.65934	0.14985	0.20679	0.13586	14669	0.01164	0.157	.....
LINC01500	14	0.45554	0.70529	0.83317	0.05095	0.10390	14669	0.01164	0.157	.....
MMP28	17	0.17782	0.83317	0.47153	0.21479	0.08392	14669	0.01165	0.157	.....
SEZ6L2	16	0.28072	0.17183	0.89311	0.52647	0.04296	14669	0.01166	0.157	.....
C1QTNF9	13	0.06893	0.20480	0.63437	0.21379	0.21578	14669	0.01166	0.157	.....
GGH	8	0.91508	0.90509	0.52547	NA	0.00700	11265	0.01167	0.157	...?.
LOC101928697	13	0.08392	0.08092	0.52947	0.14286	0.36064	14669	0.01167	0.157	.....
HSF1	8	0.78821	0.74026	0.52348	0.38561	0.01598	14669	0.01169	0.157	.....
LYSMD4	15	0.26673	0.16683	0.44256	0.19381	0.17283	14669	0.01169	0.157	.....
UNC5CL	6	0.33766	0.34665	0.31369	NA	0.05395	11265	0.01172	0.158	...?.
WDR33	2	0.53147	0.36064	0.57842	0.58541	0.02198	14669	0.01172	0.158	.....
SERHL2	22	0.08092	0.50350	0.03197	0.82118	0.11089	14669	0.01173	0.158	.....
SPTAN1	9	0.79920	0.53447	0.40460	0.26474	0.03696	14669	0.01175	0.158	.....
ENDOU	12	0.91009	0.78422	0.77323	0.04296	0.06394	14669	0.01175	0.158	.....
ITIH5	10	0.70130	0.32468	0.15185	NA	0.04396	11265	0.01176	0.158	...?.
TCAF2	20	NA	NA	0.13087	NA	0.03097	8863	0.01176	0.158	??.?.
KCNMB3	3	0.40060	0.41159	0.63137	0.41159	0.03796	14669	0.01178	0.158	.....
ATF7	12	0.28871	0.02398	0.83017	NA	0.09291	11265	0.01179	0.158	...?.
LOC100506393	12	0.57143	0.48751	0.01499	NA	0.08691	11265	0.0118	0.158	...?.
TGM5	15	0.51249	0.27073	0.24675	0.47652	0.05694	14669	0.01182	0.158	.....
RNF169	11	0.11389	0.23077	0.77423	NA	0.07093	11265	0.01183	0.158	...?.
C16orf95	16	0.58042	0.03796	0.62438	0.84116	0.03097	14669	0.01184	0.158	.....
CCT6B	17	0.83516	0.14186	0.43357	0.05295	0.19780	14669	0.01184	0.158	.....
SLC25A29	14	0.04096	0.00460	0.36863	0.56344	0.37662	14669	0.01184	0.158	.....
MMP11	22	0.03197	0.24076	0.54745	0.21978	0.27373	14669	0.01187	0.158	.....
UCN2	3	0.69231	0.42657	0.80320	0.23776	0.03497	14669	0.01188	0.158	.....
ETS1	11	0.24575	0.60440	0.75824	NA	0.02697	11265	0.01189	0.158	...?.
ADCY7	16	0.94006	0.47852	0.03996	NA	0.04196	11265	0.01189	0.158	...?.
GPR33	14	0.86114	0.22478	0.06494	0.61638	0.04795	14669	0.01189	0.158	.....
LOC101928650	1	0.49451	0.95105	0.38362	0.35864	0.02697	14669	0.01189	0.158	.....
TIPIN	15	0.33666	0.17582	0.50949	0.02098	0.42058	14669	0.0119	0.158	.....
DNASE1L3	3	0.01998	0.88112	0.23776	0.42857	0.14186	14669	0.01191	0.158	.....
LINC00462	13	0.24176	NA	0.11389	0.04196	0.34066	13691	0.01191	0.158	.?...
TUBA8	22	0.02398	0.05095	0.62238	0.45255	0.26374	14669	0.01194	0.158	.....
SYNPO2	4	0.14685	0.35365	0.65934	0.00760	0.56044	14669	0.01194	0.158	.....
FAM221A	7	0.14785	0.69930	0.14885	0.03097	0.43556	14669	0.01194	0.158	.....
ODF4	17	0.56743	0.59840	0.58042	0.03696	0.15085	14669	0.01194	0.158	.....
KIF21B	1	0.01499	0.25774	0.63037	0.57842	0.14386	14669	0.01194	0.158	.....
RPL3L	16	NA	0.70929	0.72927	NA	0.01099	9841	0.01194	0.158	?..?.
BCL6	3	0.41159	0.85514	0.12987	0.46753	0.04296	14669	0.01195	0.158	.....
SURF6	9	0.03696	0.02298	0.70130	0.28571	0.36464	14669	0.01195	0.158	.....
NOC2L	1	0.12188	0.45455	0.60040	NA	0.05794	11265	0.01196	0.158	...?.
CALML5	10	0.34865	0.07393	0.16184	0.19880	0.27672	14669	0.01196	0.158	.....
NAA20	20	0.17782	0.59540	0.71129	0.06693	0.18282	14669	0.01197	0.158	.....
TSHZ1	18	0.07992	0.31568	0.16983	NA	0.14885	11265	0.01197	0.158	...?.
IL15RA	10	0.19181	0.86813	0.17483	0.06593	0.24975	14669	0.01198	0.158	.....
ADAM29	4	0.13187	0.84016	0.83716	0.03497	0.21079	14669	0.01201	0.158	.....
C12orf65	12	0.09491	0.15584	0.49351	NA	0.11988	11265	0.01201	0.158	...?.
FAM47E-STBD1	4	0.03497	0.38561	0.50949	0.29670	0.18981	14669	0.01202	0.158	.....
RIPK2	8	0.36064	0.59041	0.02198	0.83616	0.05195	14669	0.01202	0.158	.....
KRT8P41	11	0.23576	0.50649	0.65634	0.81219	0.01798	14669	0.01203	0.158	.....
PRH1-PRR4	12	0.43756	0.00110	0.13986	0.53846	0.29471	14669	0.01204	0.158	.....
NMRK1	9	0.61938	0.01698	0.36164	0.80519	0.05794	14669	0.01204	0.158	.....
ACTL9	19	0.43257	0.79920	0.57742	0.01798	0.20979	14669	0.01204	0.158	.....
CACNG2	22	0.20779	0.32468	0.78022	0.10989	0.16484	14669	0.01205	0.158	.....
PCAT4	4	0.22478	0.18182	0.69431	0.01598	0.47153	14669	0.01206	0.158	.....
LINC01229	16	0.09590	0.05594	0.74525	0.54545	0.12687	14669	0.01207	0.158	.....
MGMT	10	0.38761	0.30569	0.68432	NA	0.03397	11265	0.01211	0.158	...?.
CBX7	22	0.56543	0.33167	0.05295	0.10390	0.26174	14669	0.01212	0.158	.....
UTP23	8	0.09091	0.72328	0.12188	0.41858	0.12687	14669	0.01212	0.158	.....
TXNDC11	16	0.48452	0.24276	0.44855	0.08791	0.18282	14669	0.01212	0.158	.....
ERICH6	3	0.03497	0.40360	0.94106	0.19181	0.18082	14669	0.01213	0.158	.....
TLR1	4	0.56244	0.40959	0.50250	0.69431	0.01698	14669	0.01213	0.158	.....

TBC1D17	19	0.17083	0.17083	0.55544	0.07093	0.33267	14669	0.01213	0.158	.....
AGA	4	0.07293	0.00630	0.77822	0.39161	0.30170	14669	0.01214	0.158	.....
FAM168B	2	0.54146	0.49950	0.31968	0.17982	0.08891	14669	0.01215	0.158	.....
DDX1	2	0.74126	0.87512	0.02597	0.72228	0.02498	14669	0.01215	0.158	.....
TMEM59L	19	0.07892	0.36364	0.14885	0.21778	0.28871	14669	0.01216	0.158	.....
FGF11	17	0.90709	0.06893	0.65934	0.24076	0.07193	14669	0.01217	0.158	.....
GPSM2	1	0.90110	0.21678	0.07792	0.80220	0.02997	14669	0.01218	0.158	.....
PTGER1	19	0.70330	0.49451	0.33766	0.08791	0.11688	14669	0.01218	0.158	.....
CCDC150	2	0.53646	0.18781	0.43656	0.03696	0.29071	14669	0.01221	0.158	.....
RPS13	11	0.25774	0.91608	0.02697	0.47053	0.09191	14669	0.01222	0.158	.....
ZNF706	8	0.03097	NA	0.17183	0.04695	0.53147	13691	0.01222	0.158	.?...
S100PBP	1	0.03397	0.21179	0.67532	0.11489	0.37063	14669	0.01224	0.158	.....
ZC3HAV1	7	0.40160	0.06593	0.10390	0.10689	0.41658	14669	0.01225	0.158	.....
ZNF890P	7	0.45954	0.99101	0.81818	NA	0.00990	11265	0.01225	0.158	...?.
APH1A	1	0.24176	NA	0.13586	0.02098	0.42957	13691	0.01226	0.158	.?...
ZYX	7	0.05694	0.61339	0.43656	0.68132	0.05894	14669	0.01226	0.158	.....
TUBA1A	12	0.39760	NA	0.40959	0.10589	0.11089	13691	0.01227	0.158	.?...
ANKRD28	3	0.22777	0.03097	0.64436	0.07093	0.42458	14669	0.01228	0.158	.....
ELOVL2-AS1	6	0.11888	0.69131	0.05195	0.54046	0.11888	14669	0.01228	0.158	.....
LINC00167	11	0.05395	NA	0.65135	0.22977	0.12587	13691	0.01228	0.158	.?...
NID1	1	0.71928	0.94106	0.24575	0.04096	0.13387	14669	0.01231	0.158	.....
GBP7	1	0.69231	0.08691	0.81718	0.20080	0.08691	14669	0.01233	0.158	.....
DISP3	1	0.26673	0.77323	0.95405	0.16284	0.05894	14669	0.01234	0.158	.....
LOC105377671	4	0.24575	0.22977	0.40959	0.62438	0.05894	14669	0.01235	0.158	.....
COX17	3	0.12088	0.65335	0.10689	0.46853	0.11489	14669	0.01235	0.158	.....
KCNK16	6	0.23676	0.85814	0.42458	NA	0.03197	11265	0.01236	0.158	...?.
PAQR6	1	0.15085	0.37263	0.71429	0.01998	0.40559	14669	0.01237	0.158	.....
ENTPD6	20	0.14685	0.54945	0.01598	0.24476	0.30769	14669	0.01238	0.159	.....
TNS3	7	0.42957	0.37862	0.07592	NA	0.08092	11265	0.01242	0.159	...?.
EPC1	10	0.41758	0.00340	0.39760	NA	0.18581	11265	0.01243	0.159	...?.
DSCR4	21	0.42757	0.02597	0.77223	NA	0.07892	11265	0.01244	0.159	...?.
SCP2	1	0.33666	0.32567	0.21778	0.81119	0.03796	14669	0.01246	0.159	.....
C11orf74	11	0.26474	0.04895	0.21578	0.12887	0.40659	14669	0.01246	0.159	.....
GLDN	15	0.18082	0.74126	0.19481	0.30370	0.10789	14669	0.01248	0.159	.....
ROPN1L	5	0.84016	0.58941	0.03796	0.32867	0.07293	14669	0.01249	0.159	.....
CAPN15	16	0.05594	NA	0.92008	0.04695	0.25774	13691	0.01249	0.159	.?...
BSCL2	11	0.68631	0.39660	0.23776	0.43057	0.04396	14669	0.0125	0.159	.....
SBF2-AS1	11	0.52248	0.05095	0.52847	0.12388	0.22278	14669	0.01251	0.159	.....
NECTIN3	3	0.53946	0.36464	0.21778	0.27173	0.09191	14669	0.01252	0.159	.....
MASP2	1	0.28172	0.02597	0.52847	0.58442	0.11289	14669	0.01252	0.159	.....
SLC37A3	7	0.10490	0.38462	0.50949	0.09491	0.27672	14669	0.01253	0.159	.....
PSG3	19	0.33267	NA	0.48951	0.04595	0.18382	13691	0.01254	0.159	.?...
MDH1B	2	0.08991	0.79520	0.09291	0.01898	0.62038	14669	0.01255	0.159	.....
LINC00861	8	0.76324	0.03896	0.38362	0.54046	0.06494	14669	0.01257	0.159	.....
HNRNPUL2-BSCL	11	0.98801	0.32967	0.22577	0.42058	0.03497	14669	0.01261	0.160	.....
ZNF415	19	0.09291	0.28871	0.31069	0.11688	0.34565	14669	0.01263	0.160	.....
FZD10	12	0.19680	0.41858	0.04496	NA	0.13986	11265	0.01265	0.160	...?.
SLC47A2	17	0.61139	0.18382	0.99301	0.25075	0.05195	14669	0.01266	0.160	.....
NPBWR1	8	0.39061	NA	0.18881	NA	0.04196	10287	0.01269	0.160	.?.?.
NUP205	7	0.96404	0.14785	0.07592	0.05495	0.30869	14669	0.0127	0.160	.....
RBM46	4	0.70729	0.17283	0.23077	0.63137	0.04196	14669	0.01271	0.160	.....
SRGAP1	12	0.44955	0.53846	0.03097	NA	0.08591	11265	0.01272	0.160	...?.
USP8	15	0.33167	0.92408	0.73826	0.06294	0.10789	14669	0.01272	0.160	.....
CCDC144NL	17	0.22378	0.63836	0.37662	0.82018	0.02398	14669	0.01273	0.160	.....
SUSD1	9	0.40460	0.38861	0.48152	0.04396	0.23976	14669	0.01274	0.160	.....
EDDM3A	14	0.12687	0.90010	0.05195	0.93407	0.04595	14669	0.01274	0.160	.....
GAPDHS	19	0.73826	0.65834	0.48252	0.35365	0.02498	14669	0.01274	0.160	.....
LOC339298	18	0.18681	0.04396	0.34665	NA	0.16583	11265	0.01277	0.161	...?.
ERCC5	13	0.06494	0.96304	0.15185	0.31169	0.14785	14669	0.01281	0.161	.....
NPSR1-AS1	7	0.03796	0.04496	0.97403	NA	NA	3253	0.01281	0.161	...??
KLK2	19	0.00560	0.33367	0.90809	0.07592	0.50450	14669	0.01282	0.161	.....
CLUHP3	16	0.21079	0.85115	0.92707	0.14186	0.07393	14669	0.01282	0.161	.....
ZNF295-AS1	21	0.44156	0.86214	0.18382	NA	0.03297	11265	0.01283	0.161	...?.
PTGIR	19	0.50549	0.47453	0.04296	0.65834	0.05894	14669	0.01283	0.161	.....
SYCP3	12	0.72228	0.08192	0.52448	0.38262	0.06693	14669	0.01285	0.161	.....
POMK	8	0.42657	0.26573	0.71728	0.03297	0.25674	14669	0.01286	0.161	.....
FAM21C	10	0.67632	0.18781	0.35964	NA	0.04396	11265	0.01287	0.161	...?.
COL1A1	17	0.22278	0.61638	0.10490	0.07493	0.32368	14669	0.01288	0.161	.....
WDR1	4	0.67233	0.97502	0.48352	0.42957	0.01499	14669	0.01288	0.161	.....

KLHL6	3	0.57143	0.45954	0.09890	0.44456	0.06693	14669	0.01288	0.161	.....
TOM1L2	17	0.14585	0.59640	0.13387	0.10589	0.30569	14669	0.01289	0.161	.....
ZC3H4	19	0.02697	0.15385	0.14785	0.31469	0.41958	14669	0.01289	0.161	.....
CCDC71L	7	0.21079	NA	0.78921	0.30669	0.04895	13691	0.01291	0.161	.?...
SIMC1	5	0.70829	0.48751	0.47453	0.09890	0.09790	14669	0.01291	0.161	.....
TNFSF10	3	0.42458	0.54146	0.26074	0.75225	0.02498	14669	0.01291	0.161	.....
TFG	3	0.10290	0.66134	0.17682	0.41858	0.11988	14669	0.01293	0.161	.....
PWRN4	15	0.10190	0.43057	0.19281	0.29271	0.19780	14669	0.01294	0.161	.....
EIF4G1	3	0.07193	0.44256	0.14386	0.44056	0.17483	14669	0.01294	0.161	.....
SPDYE4	17	0.21179	NA	0.81119	NA	0.02697	10287	0.01294	0.161	.?.?.
LINC00643	14	0.46653	0.02098	0.61838	0.22877	0.19281	14669	0.01295	0.161	.....
AFTPH	2	0.75724	0.82118	0.27473	0.15385	0.06194	14669	0.01298	0.161	.....
LOC151121	2	0.13986	0.98601	0.42358	0.20779	0.09890	14669	0.01301	0.161	.....
MME	3	0.17283	0.29071	0.64436	0.66833	0.04695	14669	0.01301	0.161	.....
CASP8	2	0.58442	0.75724	0.05295	0.01698	0.40360	14669	0.01301	0.161	.....
ARF5	7	0.00080	0.02897	0.83516	NA	0.48352	11265	0.01301	0.161	...?.
SEMA6D	15	0.87912	0.81419	0.09690	NA	0.02398	11265	0.01301	0.161	...?.
FBRS	16	0.40859	NA	0.39960	0.02797	0.23377	13691	0.01302	0.161	.?...
ZBTB20-AS3	3	0.69431	0.96404	0.12587	0.02697	0.21578	14669	0.01303	0.161	.....
ERN1	17	0.09391	0.63636	0.17582	0.05894	0.41459	14669	0.01304	0.161	.....
LINC01411	5	0.17882	0.27572	0.58242	0.28172	0.12587	14669	0.01305	0.161	.....
PPP3CB	10	0.52048	0.27473	0.71129	NA	0.02997	11265	0.01305	0.161	...?.
BZW2	7	0.48751	0.11688	0.33267	NA	0.07393	11265	0.01306	0.161	...?.
SLC44A3	1	0.61538	0.00410	0.13886	0.23077	0.37862	14669	0.01306	0.161	.....
PRKACG	9	0.05994	0.59241	0.61638	NA	0.07193	11265	0.01307	0.161	...?.
BUD13	11	0.88112	0.48651	0.17283	0.13986	0.09990	14669	0.01307	0.161	.....
PSPC1	13	0.01399	0.66034	0.85714	0.93207	0.03996	14669	0.01307	0.161	.....
PQLC2	1	0.44655	0.75225	0.22378	0.13986	0.11688	14669	0.01308	0.161	.....
PPP3R1	2	0.32767	0.58442	0.45554	0.71728	0.02298	14669	0.01311	0.161	.....
SLC8B1	12	0.31169	0.02198	0.27173	0.03796	0.67532	14669	0.01311	0.161	.....
RSU1P2	10	0.42857	0.50350	0.72028	NA	0.02398	11265	0.01311	0.161	...?.
HCG9	6	0.76723	0.38362	0.21578	NA	0.03497	11265	0.01312	0.161	...?.
NUP210P1	3	0.71728	0.29471	0.00270	0.91908	0.07293	14669	0.01313	0.161	.....
ADRB1	10	0.30569	0.14685	0.38961	0.33367	0.13187	14669	0.01313	0.161	.....
LGI3	20	NA	0.49750	0.12088	0.21379	0.10190	13245	0.01314	0.161	?....
HEBP2	6	0.05694	NA	0.18681	0.08092	0.38362	13691	0.01314	0.161	.?...
CHRNB3	8	0.36863	0.83117	0.84815	0.03497	0.14286	14669	0.01314	0.161	.....
ARHGAP39	8	0.15185	NA	0.16384	NA	0.07992	10287	0.01317	0.161	.?.?.
WDR26	1	0.06593	0.48052	0.72028	0.09990	0.25275	14669	0.01317	0.161	.....
PPME1	11	0.12887	0.76424	0.48951	NA	0.04895	11265	0.01318	0.161	...?.
LOC100128531	22	0.55045	0.27173	0.12388	0.42957	0.08891	14669	0.0132	0.161	.....
ARHGEF10L	1	0.69830	0.45854	0.66034	0.51149	0.01830	14669	0.01321	0.161	.....
PLD3	19	0.39161	0.28871	0.47652	0.07393	0.22078	14669	0.01321	0.161	.....
TNFRSF10A	8	0.88412	0.97203	0.80120	NA	0.00540	11265	0.01322	0.161	...?.
RBBP5	1	0.56044	0.25674	0.20579	0.56044	0.05495	14669	0.01322	0.161	.....
LOC100506100	9	NA	NA	0.38961	0.04396	0.14086	12267	0.01322	0.161	??...
LOC101927587	1	0.45954	0.72428	0.19081	0.55744	0.03497	14669	0.01322	0.161	.....
SRSF3	6	0.62637	0.18482	0.28971	0.03696	0.32767	14669	0.01323	0.161	.....
TRIM11	1	0.02897	0.88611	0.53447	0.24575	0.15485	14669	0.01323	0.161	.....
ATP5C1	10	0.08891	0.30569	0.15684	0.03097	0.68232	14669	0.01324	0.161	.....
CALCA	11	0.42957	0.39061	0.34066	0.83616	0.02298	14669	0.01324	0.161	.....
PEX14	1	0.92907	0.01898	0.11089	0.19481	0.25874	14669	0.01326	0.161	.....
LINC00662	19	0.07692	0.99301	0.76523	0.66933	0.02597	14669	0.01326	0.161	.....
PPFIBP2	11	0.71528	0.47752	0.30270	0.25674	0.06094	14669	0.01327	0.161	.....
PALM2-AKAP2	9	0.54146	0.82018	0.19580	0.00560	0.43157	14669	0.01327	0.161	.....
DEF8	16	0.30669	0.73227	0.27073	0.04196	0.26474	14669	0.01328	0.161	.....
SLC22A31	16	0.12388	0.54146	0.45654	0.40360	0.08891	14669	0.01329	0.161	.....
GSG1	12	0.27772	0.55944	0.48851	0.24276	0.08891	14669	0.0133	0.161	.....
TPST1	7	0.89610	0.64535	0.94505	NA	0.00699	11265	0.0133	0.161	...?.
ATRN	20	0.63936	0.14086	0.29770	0.39660	0.08092	14669	0.01331	0.161	.....
LOC101927770	20	0.39560	0.35465	0.40859	0.91209	0.01998	14669	0.01331	0.161	.....
KLHDC8B	3	0.25075	0.69131	0.62737	0.31369	0.05594	14669	0.01331	0.161	.....
PNLDC1	6	0.22877	0.64635	0.26973	0.31768	0.09491	14669	0.01333	0.161	.....
NME9	3	0.39960	0.40460	0.76024	0.02697	0.24575	14669	0.01334	0.161	.....
PNPLA2	11	0.50549	0.43157	0.78921	0.03097	0.19381	14669	0.01334	0.161	.....
DHX29	5	0.18182	0.90709	0.94705	NA	0.02098	11265	0.01336	0.161	...?.
SLC52A1	17	0.76024	0.44056	0.35864	0.64036	0.01898	14669	0.01336	0.161	.....
LPIN3	20	0.20280	0.17083	0.98801	0.04895	0.29670	14669	0.01337	0.161	.....
TRMT61A	14	0.11888	0.02498	0.54945	0.66134	0.15385	14669	0.01337	0.161	.....

METTL8	2	0.25774	0.34166	0.28272	0.01200	0.58242	14669	0.01338	0.161	.....
EXOSC9	4	0.22478	0.27273	0.91109	0.01399	0.41658	14669	0.01341	0.161	.....
HMGA2	12	0.29271	0.63636	0.41459	0.95305	0.01499	14669	0.01341	0.161	.....
PODN	1	0.00750	0.59441	0.85415	0.37363	0.16384	14669	0.01342	0.161	.....
MINK1	17	0.26873	0.27173	0.64835	0.50450	0.05594	14669	0.01342	0.161	.....
LINC01432	20	0.85415	0.54845	0.34665	0.17483	0.06194	14669	0.01343	0.161	.....
SIK1	21	0.87113	NA	0.51648	0.00490	0.26374	13691	0.01344	0.161	.?...
GH1	17	0.97103	0.42158	0.00999	0.32867	0.11588	14669	0.01344	0.161	.....
ARPC5	1	0.50949	0.60939	0.69930	0.11489	0.08292	14669	0.01346	0.161	.....
LOC100129534	1	0.78521	0.69730	0.60839	0.07892	0.07493	14669	0.01346	0.161	.....
TUBA1B	12	0.37463	NA	0.66234	0.11489	0.09191	13691	0.01349	0.161	.?...
LEF1	4	0.49750	0.90310	0.09790	0.06993	0.19580	14669	0.01349	0.161	.....
MRFAP1	4	0.00260	NA	0.82318	0.08092	0.45355	13691	0.01349	0.161	.?...
C4orf36	4	0.62537	0.17483	0.82418	0.57642	0.02597	14669	0.01349	0.161	.....
HLF	17	0.12787	0.09291	0.29271	0.52248	0.17582	14669	0.0135	0.161	.....
ILF2	1	0.65235	0.31568	0.99800	0.39061	0.02498	14669	0.0135	0.161	.....
PRKCQ	10	0.96803	0.98202	0.29371	NA	0.01040	11265	0.0135	0.161	...?.
GHSR	3	0.14086	0.44056	0.38661	0.55045	0.07493	14669	0.0135	0.161	.....
LOC101929488	8	0.03596	0.62038	0.04695	NA	0.22078	11265	0.0135	0.161	...?.
LOC101928775	9	0.53047	0.89910	0.75924	0.00140	0.39461	14669	0.01351	0.161	.....
DNASE2B	1	0.12987	0.25674	0.58641	0.15085	0.23277	14669	0.01352	0.161	.....
DCSTAMP	8	0.42857	0.34466	0.65934	0.63736	0.02597	14669	0.01353	0.161	.....
TTK	6	0.73826	0.32268	0.97902	NA	0.01499	11265	0.01355	0.161	...?.
ADH1C	4	0.47652	0.27572	0.99401	0.70330	0.01598	14669	0.01355	0.161	.....
TNFAIP3	6	0.39560	0.80619	0.28172	0.02697	0.26973	14669	0.01357	0.161	.....
CRYZL1	21	0.81918	0.08492	0.78621	0.82617	0.01598	14669	0.0136	0.161	.....
SENP5	3	0.23277	0.26973	0.41558	0.27473	0.14086	14669	0.01361	0.161	.....
TMPRSS11D	4	0.03297	0.01160	0.51449	0.43257	0.40460	14669	0.01362	0.161	.....
CCDC13	3	0.75624	0.12787	0.31968	0.05894	0.26274	14669	0.01362	0.161	.....
LOC105374546	4	0.50050	0.42657	0.82218	0.16284	0.07293	14669	0.01363	0.161	.....
RPL31	2	0.10889	0.32967	0.11389	0.27173	0.27572	14669	0.01363	0.161	.....
FOXO3B	17	0.05694	0.12787	0.13287	0.33866	0.37363	14669	0.01364	0.161	.....
VEGFC	4	0.53447	0.19980	0.92907	0.47453	0.03297	14669	0.01364	0.161	.....
SIX3-AS1	2	0.68731	NA	0.07992	0.34965	0.06094	13691	0.01365	0.161	.?...
LRRIQ4	3	0.07792	0.92507	0.63337	0.28372	0.08791	14669	0.01366	0.161	.....
C2CD3	11	0.92408	0.64136	0.73926	NA	0.00899	11265	0.01367	0.161	...?.
ST6GALNAC1	17	0.47053	0.56543	0.03297	0.14486	0.23576	14669	0.01368	0.161	.....
ESYT2	7	0.52348	0.70629	0.90509	NA	0.01299	11265	0.01368	0.161	...?.
EMX2OS	10	0.01698	0.30370	0.35365	0.95504	0.10290	14669	0.01369	0.161	.....
ACTR2	2	NA	0.25974	0.83017	0.03097	0.19381	13245	0.0137	0.161	?....
OLR1	12	0.14585	0.19381	0.65834	0.09690	0.29770	14669	0.01372	0.161	.....
MDM1	12	0.39061	0.59441	0.41359	0.56444	0.03197	14669	0.01372	0.161	.....
SPIRE1	18	0.27373	0.38162	0.84515	0.85814	0.01698	14669	0.01373	0.161	.....
GPR63	6	0.80719	0.02398	0.24476	0.38661	0.13387	14669	0.01374	0.161	.....
XDH	2	0.86314	0.05395	0.26873	0.99900	0.02597	14669	0.01374	0.161	.....
LOC101929608	20	0.80719	0.21678	0.32468	0.29770	0.07193	14669	0.01375	0.161	.....
CPQ	8	0.46254	0.78122	0.00800	NA	0.09890	11265	0.01375	0.161	...?.
OPA3	19	0.12887	0.99500	0.23477	0.02498	0.39960	14669	0.01376	0.161	.....
DDX55	12	0.85315	0.03097	0.17083	0.10390	0.31968	14669	0.01376	0.161	.....
TIGIT	3	0.35265	0.58342	0.92408	0.87013	0.00899	14669	0.01376	0.161	.....
APOC3	11	0.81019	0.41658	0.07493	0.74725	0.03097	14669	0.01377	0.161	.....
LGALS16	19	0.43257	0.18282	0.49550	0.18881	0.14486	14669	0.01377	0.161	.....
UGCG	9	0.14985	0.96803	0.59540	0.06993	0.17483	14669	0.01379	0.161	.....
ZSCAN30	18	0.24476	0.09191	0.13586	0.57243	0.16184	14669	0.01379	0.161	.....
DOCK3	3	0.54346	0.98002	0.75624	0.63037	0.00799	14669	0.01379	0.161	.....
MC1R	16	0.13686	0.01350	0.44356	0.93007	0.12488	14669	0.0138	0.161	.....
GOLT1B	12	NA	0.05195	0.45455	NA	0.07093	9841	0.01381	0.161	?..?.
ZNF708	19	0.10290	0.62937	0.33267	0.07692	0.31469	14669	0.01385	0.161	.....
VN1R10P	6	0.14186	0.10390	0.27473	0.04995	0.61139	14669	0.01387	0.161	.....
PAQR5	15	0.06494	0.62138	0.19680	0.36663	0.17083	14669	0.01387	0.161	.....
PCDHB9	5	0.68032	0.58641	0.21479	0.60839	0.02597	14669	0.01389	0.161	.....
CAPN2	1	0.22977	0.60140	0.35564	0.03297	0.34266	14669	0.01389	0.161	.....
CCDC153	11	0.82617	0.66434	0.77522	0.03097	0.11289	14669	0.01389	0.161	.....
RHOH	4	0.81718	0.71429	0.82917	0.16184	0.03397	14669	0.01391	0.161	.....
VAMP5	2	0.25774	0.13287	0.04296	0.08691	0.58242	14669	0.01392	0.161	.....
SDCCAG8	1	0.11189	0.67033	0.19381	0.04895	0.42258	14669	0.01392	0.161	.....
LOC284080	17	0.53347	NA	0.42458	0.07093	0.12987	13691	0.01393	0.161	.?...
LINC01556	6	0.39660	0.78122	0.51648	0.11389	0.10290	14669	0.01393	0.161	.....
IFI27L1	14	0.80320	0.29970	0.59241	NA	0.02298	11265	0.01394	0.161	...?.

LOC101929579	3	0.99301	0.47752	0.85514	0.00560	0.22677	14669	0.01397	0.161	.....
FEV	20	NA	NA	0.64336	0.26074	0.03097	12267	0.01397	0.161	??...
PDX1	13	0.11988	0.50150	0.54046	0.68232	0.04895	14669	0.01397	0.161	.....
SYNGR1	22	0.27273	0.00899	0.16983	0.46853	0.30270	14669	0.01398	0.161	.....
DBP	19	0.38661	0.56943	0.69830	0.07093	0.14486	14669	0.01399	0.161	.....
SLC31A2	9	0.95205	0.71329	0.02098	0.21878	0.10589	14669	0.01401	0.161	.....
C2orf81	2	0.44456	0.02997	0.75325	0.07293	0.33067	14669	0.01401	0.161	.....
FAM21A	10	NA	NA	0.31668	NA	0.02398	8863	0.01402	0.161	??.?.
PRR5L	11	0.49850	0.27473	0.81319	0.10490	0.12787	14669	0.01404	0.161	.....
TMEM108	3	NA	0.38162	0.96803	0.14486	0.05894	13245	0.01405	0.161	?....
TRAPPC6B	14	0.68631	0.22677	0.84915	NA	0.02398	11265	0.01405	0.161	...?.
SPATA42	1	0.97103	0.18981	0.17283	0.44156	0.05694	14669	0.01405	0.161	.....
GPR78	4	0.93906	0.12288	0.47453	0.57942	0.03097	14669	0.01405	0.161	.....
LOC102724156	9	0.39960	0.57243	0.25674	0.18182	0.12587	14669	0.01406	0.161	.....
COX7A2	6	0.22478	0.02797	0.21678	NA	0.21878	11265	0.01406	0.161	...?.
SCGB3A2	5	0.63936	0.37962	0.00680	0.89810	0.06394	14669	0.01406	0.161	.....
PROKR2	20	0.69830	0.05395	0.09590	0.21578	0.25574	14669	0.01406	0.161	.....
FBXO16	8	0.12388	0.79021	0.21179	NA	0.07792	11265	0.01407	0.161	...?.
OR6B1	7	0.16583	0.35664	0.83516	0.39560	0.07293	14669	0.01411	0.161	.....
HOXB2	17	0.24875	NA	0.20480	0.00600	0.60440	13691	0.01411	0.161	.?...
RNF6	13	0.24376	0.44955	0.53946	0.17083	0.14086	14669	0.01412	0.161	.....
MEIS2	15	0.68032	0.22278	0.46354	0.10989	0.14985	14669	0.01412	0.161	.....
MRGPRX4	11	0.77423	0.04196	0.02897	NA	0.17183	11265	0.01413	0.161	...?.
ZNF32	10	0.03097	0.06294	0.93107	0.39061	0.24076	14669	0.01414	0.161	.....
PRRT1	6	0.58841	0.63736	0.29271	0.36663	0.04595	14669	0.01414	0.161	.....
LOC148709	1	0.43057	0.00240	0.39361	0.61039	0.18781	14669	0.01414	0.161	.....
IFIT5	10	0.50450	0.14186	0.27972	0.84316	0.04096	14669	0.01415	0.161	.....
RBP3	10	0.11788	0.18282	0.35065	NA	0.13886	11265	0.01416	0.161	...?.
CHTF18	20	NA	0.42158	0.29570	0.00860	0.41259	13245	0.01418	0.161	?....
TREM1	6	0.35365	0.46054	0.76523	0.00250	0.54745	14669	0.01419	0.161	.....
TMEM134	11	0.35664	0.37962	0.78821	0.15884	0.10589	14669	0.01419	0.161	.....
LOC101927166	17	0.04196	0.69530	0.11489	0.97303	0.07393	14669	0.01422	0.162	.....
KIF26A	14	0.40659	0.37363	0.79720	0.04096	0.21479	14669	0.01422	0.162	.....
PI4K2B	4	0.56044	0.92507	0.53646	0.02498	0.16883	14669	0.01423	0.162	.....
ZPBP2	17	0.99600	0.69930	0.45255	0.38262	0.01798	14669	0.01423	0.162	.....
IL1R1	2	NA	0.30969	0.10589	0.17682	0.16484	13245	0.01425	0.162	?....
FGD5P1	3	0.13287	0.26773	0.69131	0.00300	0.81718	14669	0.01426	0.162	.....
MRVI1	11	0.06394	0.87113	0.73526	0.17682	0.13686	14669	0.01426	0.162	.....
SIX6	14	0.59141	0.70629	0.25974	0.30869	0.05395	14669	0.01426	0.162	.....
PHGR1	15	0.03197	0.21978	0.25075	0.12987	0.53447	14669	0.01427	0.162	.....
TULP4	6	0.57742	0.28472	0.31868	0.07193	0.22677	14669	0.01428	0.162	.....
CCL5	17	0.13287	0.24176	0.28472	0.66933	0.09990	14669	0.01429	0.162	.....
LOC441242	7	0.87313	0.14785	0.95604	0.22178	0.05395	14669	0.0143	0.162	.....
KCNJ12	17	0.65435	NA	0.38462	0.36963	0.03197	13691	0.01432	0.162	.?...
DEFB116	20	0.09990	NA	0.47552	0.40659	0.08292	13691	0.01433	0.162	.?...
EPB41L4A-AS1	5	0.18781	0.53447	0.90210	0.00510	0.49950	14669	0.01434	0.162	.....
NUBPL	14	0.01898	0.46553	0.60340	0.95105	0.06294	14669	0.01435	0.162	.....
LINC00543	13	0.16583	0.05495	0.25774	0.12088	0.49750	14669	0.01435	0.162	.....
OPRK1	8	0.45954	0.58442	0.78122	NA	0.02098	11265	0.0144	0.162	...?.
TMCO2	1	0.53646	0.43856	0.22178	0.65035	0.03696	14669	0.0144	0.162	.....
CLEC4A	12	0.25974	NA	0.25574	NA	0.05395	10287	0.0144	0.162	.?.?.
SPINK9	5	0.14685	0.52248	0.38561	0.06993	0.31169	14669	0.01441	0.162	.....
HLA-G	6	0.25075	0.49750	0.51748	NA	0.04895	11265	0.01442	0.162	...?.
LOC105376430	10	0.03696	0.57443	0.25175	0.31668	0.22677	14669	0.01442	0.162	.....
YWHAQ	2	0.27672	0.11289	0.04895	0.59341	0.19081	14669	0.01442	0.162	.....
LINC01344	1	0.78422	0.97702	0.23876	0.04396	0.13686	14669	0.01443	0.162	.....
SFT2D3	2	0.28671	0.17183	0.69830	0.66833	0.04795	14669	0.01444	0.162	.....
ZNF879	5	0.93806	0.05495	0.23776	0.03397	0.40160	14669	0.01446	0.162	.....
TET3	2	0.57542	0.10490	0.94605	0.08991	0.16783	14669	0.01446	0.162	.....
FAM109B	22	0.05794	0.23876	0.11888	0.91808	0.12787	14669	0.01447	0.162	.....
GSS	20	0.07293	0.56044	0.15185	0.19381	0.30070	14669	0.01448	0.162	.....
KRT76	12	0.58641	0.37762	0.40060	0.41059	0.04995	14669	0.01449	0.162	.....
FOXN1	17	0.98501	0.41958	0.40859	0.01798	0.22777	14669	0.01451	0.162	.....
CR1	1	0.47353	0.19181	0.58541	0.29271	0.09191	14669	0.01452	0.162	.....
F2	11	0.04496	0.11089	0.63437	NA	NA	3253	0.01453	0.162	...??
MEIKIN	5	0.02298	0.55345	0.01898	0.89610	0.19680	14669	0.01455	0.162	.....
OR56A4	11	0.60739	0.42557	0.45255	NA	0.03097	11265	0.01455	0.162	...?.
TMEM119	12	0.45155	0.24476	0.03497	0.37263	0.18881	14669	0.01455	0.162	.....
TMEM171	5	0.79321	0.25075	0.16084	0.03297	0.34366	14669	0.01456	0.162	.....

LOC101926944	1	0.32567	0.33467	0.92707	0.61638	0.02797	14669	0.01457	0.162	.....
LINC01543	18	0.12787	0.69231	0.22977	0.04695	0.40160	14669	0.01458	0.162	.....
VPS9D1-AS1	16	0.77123	0.93806	0.20380	0.24176	0.04795	14669	0.01459	0.162	.....
LINC01289	8	0.16983	0.55445	0.03097	0.16184	0.36563	14669	0.01459	0.162	.....
LCMT2	15	0.40659	NA	0.61339	0.27073	0.04995	13691	0.01459	0.162	.?...
JPH4	20	NA	NA	0.84316	0.21978	0.03097	12267	0.0146	0.162	??...
UGDH-AS1	4	0.57343	0.17582	0.68432	0.50250	0.04296	14669	0.01461	0.162	.....
C1orf131	1	0.52647	0.65135	0.64136	0.95704	0.00710	14669	0.01461	0.162	.....
DOCK9-AS2	13	0.62637	0.88212	0.50150	0.02697	0.16583	14669	0.01463	0.162	.....
PTTG1	5	0.87812	0.00200	0.65035	0.98102	0.04595	14669	0.01463	0.162	.....
ZNF792	19	0.68332	0.18482	0.49351	0.12587	0.14785	14669	0.01464	0.162	.....
DNAJC16	1	0.69031	0.63636	0.38861	0.02997	0.20280	14669	0.01466	0.162	.....
TMEM265	20	NA	NA	0.04096	0.08791	NA	4255	0.01467	0.162	??..?
ZNF827	4	0.17083	0.22478	0.51449	0.28871	0.16284	14669	0.0147	0.162	.....
DCAF6	1	0.03397	0.17283	0.07892	0.19980	0.60140	14669	0.01471	0.162	.....
IRAK3	12	0.76224	0.05395	0.59640	0.48052	0.06194	14669	0.01473	0.162	.....
KIF2A	5	0.31269	0.38561	0.86114	0.33167	0.05994	14669	0.01474	0.162	.....
SPRYD7	13	0.99800	0.08292	0.09790	0.05694	0.36164	14669	0.01475	0.162	.....
MVP	16	0.38661	0.11489	0.57143	0.15185	0.20579	14669	0.01475	0.162	.....
SCHLAP1	2	0.29371	0.66833	0.44655	0.03397	NA	6657	0.01476	0.162	....?
PARP10	8	0.92507	0.80619	0.77323	0.09091	0.04795	14669	0.01476	0.162	.....
PITPNC1	17	0.25974	0.71628	0.51548	0.47952	0.04296	14669	0.01476	0.162	.....
JRK	8	0.02797	0.29970	0.14186	0.33367	0.36763	14669	0.01478	0.162	.....
LOC100507537	3	0.05495	0.13886	0.54645	0.03297	0.70030	14669	0.0148	0.162	.....
ZSWIM3	20	0.60539	0.76324	0.04895	0.04396	0.30769	14669	0.0148	0.162	.....
PKIG	20	0.33067	0.67932	0.47952	0.01399	0.36264	14669	0.01482	0.162	.....
REXO1	19	0.46454	0.01399	0.06993	0.83417	0.16084	14669	0.01484	0.162	.....
TTLL7	1	0.87812	0.39461	0.36364	0.00310	0.47752	14669	0.01485	0.162	.....
RPL10A	6	0.58242	0.04096	0.28571	0.92907	0.05195	14669	0.01487	0.162	.....
LINC00239	14	0.04496	0.27872	0.10589	0.75524	0.17982	14669	0.01488	0.162	.....
PRDM7	16	0.69431	0.22977	0.88511	0.05195	0.16284	14669	0.01489	0.162	.....
PNN	14	0.76723	0.03397	0.91508	NA	0.04695	11265	0.0149	0.162	...?.
KRIT1	7	0.03896	0.98801	0.11189	0.86913	0.07293	14669	0.0149	0.162	.....
MPZL1	1	0.19481	0.83616	0.31568	0.00480	0.61039	14669	0.01493	0.162	.....
LINC00521	14	0.43357	0.71628	0.84615	NA	0.01798	11265	0.01493	0.162	...?.
CCDC191	3	0.91608	0.20579	0.91009	0.09291	0.09391	14669	0.01493	0.162	.....
RFC1	4	0.97602	0.36064	0.29271	0.26274	0.05894	14669	0.01493	0.162	.....
ISCU	12	0.66234	0.60140	0.66533	0.04396	0.14086	14669	0.01494	0.162	.....
TMEM254-AS1	10	0.86014	0.47952	0.07193	NA	0.04795	11265	0.01495	0.162	...?.
PBXIP1	1	0.31469	0.35864	0.38961	0.23377	0.13586	14669	0.01496	0.162	.....
ZNF439	19	0.60240	NA	0.03097	0.41159	0.08492	13691	0.01496	0.162	.?...
LOC101054525	11	0.48352	0.25674	0.00690	0.56444	0.17882	14669	0.01497	0.162	.....
FAAP20	1	0.10190	0.63536	0.18881	0.02897	0.55844	14669	0.01497	0.162	.....
GGT7	20	0.07692	0.92507	0.13586	0.08392	0.36763	14669	0.01497	0.162	.....
ZNF689	16	NA	NA	0.04496	0.08591	NA	4255	0.01498	0.162	??..?
MRPL34	19	0.86913	0.70430	0.03996	0.60839	0.03696	14669	0.01499	0.162	.....
PIGG	4	0.73027	0.39361	0.91209	0.04396	0.13387	14669	0.01499	0.162	.....
SAE1	19	0.20080	0.15884	0.54745	0.57842	0.08891	14669	0.01501	0.162	.....
SNURF	15	0.32567	NA	0.07592	0.16683	0.19880	13691	0.01502	0.162	.?...
SOX10	22	0.03097	0.01499	0.13786	0.23077	0.79021	14669	0.01503	0.162	.....
GAS5	1	0.35465	0.38861	0.20180	0.16184	0.20380	14669	0.01504	0.162	.....
C3orf18	3	0.27772	0.82418	0.97502	0.63237	0.01499	14669	0.01504	0.162	.....
SKP1P2	12	0.17582	0.65734	0.18681	0.28971	0.14885	14669	0.01505	0.162	.....
RBFADN	18	0.04496	0.35864	0.34066	0.49950	0.16983	14669	0.01505	0.162	.....
MKX	10	0.68432	0.22378	0.93007	NA	0.02398	11265	0.01506	0.162	...?.
UQCRFS1	19	0.87912	0.21678	0.90110	0.16384	0.06494	14669	0.01506	0.162	.....
TLX1	10	0.68931	0.85115	0.03996	0.19381	0.12388	14669	0.01506	0.162	.....
MED22	9	0.07892	0.20080	0.42358	0.28372	0.25375	14669	0.01508	0.162	.....
PHRF1	11	0.56743	0.44356	0.25874	0.09491	0.18581	14669	0.01508	0.162	.....
RTTN	18	0.79321	0.93007	0.34565	NA	0.01499	11265	0.0151	0.162	...?.
BNC2	9	0.65235	0.26174	0.22078	0.34066	0.09191	14669	0.01511	0.162	.....
SLC22A18AS	11	0.04595	0.96903	0.53846	NA	0.06793	11265	0.01512	0.162	...?.
EXT2	11	0.17283	0.54246	0.64635	NA	0.05195	11265	0.01513	0.162	...?.
CKS2	9	0.33467	0.37762	0.54545	0.72428	0.03197	14669	0.01514	0.162	.....
ANKRD55	5	0.05694	0.14785	0.81419	NA	0.13487	11265	0.01515	0.162	...?.
LINC01061	4	0.17483	0.63636	0.07892	0.26074	0.21379	14669	0.01516	0.162	.....
PCGF1	2	0.22877	NA	0.36863	0.02897	0.34166	13691	0.01516	0.162	.?...
FDPS	1	0.16084	0.19980	0.52747	0.08492	0.35864	14669	0.01516	0.162	.....
OTUB2	14	0.12687	0.53646	0.34466	NA	0.08691	11265	0.01517	0.162	...?.

RHBDF1	16	0.09291	0.18581	0.38561	NA	0.15385	11265	0.01517	0.162	...?.
ACOT8	20	0.37562	0.70430	0.11289	0.21678	0.14486	14669	0.01517	0.162	.....
UBXN8	8	0.58442	0.91808	0.34366	0.10390	0.09890	14669	0.01518	0.162	.....
LOC101927143	1	0.33566	0.12687	0.80819	0.31868	0.10589	14669	0.01518	0.162	.....
UBC	12	0.02897	NA	0.48651	NA	0.10889	10287	0.0152	0.162	.?.?.
TTI1	20	0.72028	0.26673	0.75824	0.19780	0.07093	14669	0.0152	0.162	.....
HAMP	19	0.11489	0.61938	0.28272	0.16583	0.23277	14669	0.0152	0.162	.....
ZFP57	6	0.02198	0.78122	0.94605	NA	0.07293	11265	0.01521	0.162	...?.
SMYD5	2	0.01798	0.89810	0.20280	0.47053	0.17483	14669	0.01521	0.162	.....
LOC100508631	4	0.56643	0.79520	0.37762	0.11788	0.09990	14669	0.01521	0.162	.....
SHD	20	NA	0.20579	0.60440	0.08192	0.16883	13245	0.01521	0.162	?....
LINC00319	21	0.02697	0.87912	0.04895	0.22278	0.38861	14669	0.01522	0.162	.....
ZNF492	19	0.73526	0.21079	0.37562	0.00799	0.49251	14669	0.01523	0.162	.....
SEC24D	4	0.97403	0.56743	0.58242	0.21079	0.03796	14669	0.01523	0.162	.....
ACTA1	1	0.41059	0.54346	0.59441	0.63536	0.02498	14669	0.01524	0.162	.....
C12orf29	12	0.10090	NA	0.14785	0.44855	0.12987	13691	0.01525	0.162	.?...
LINC01548	21	0.39161	0.21079	0.18082	0.20180	0.22378	14669	0.01525	0.162	.....
LILRP2	19	0.15684	0.84815	0.36963	0.30270	0.09790	14669	0.01525	0.162	.....
USP22	17	0.54645	0.79520	0.69331	0.13387	0.06693	14669	0.01526	0.162	.....
LINC01435	10	0.94705	0.76024	0.71628	0.30869	0.01798	14669	0.01527	0.162	.....
ZNF174	16	0.99700	0.24575	0.59740	0.27173	0.04695	14669	0.01527	0.162	.....
MPDZ	9	0.73227	0.13586	0.73626	0.05295	0.21279	14669	0.01527	0.162	.....
TSPAN12	7	0.55544	0.24076	0.22078	NA	0.06893	11265	0.01528	0.162	...?.
TTBK1	6	0.75125	0.75924	0.25674	0.07592	0.12987	14669	0.01529	0.162	.....
MIOS	7	0.03896	0.08891	0.78222	0.22577	0.34865	14669	0.0153	0.162	.....
VPS41	7	0.57542	0.10689	0.17183	0.17782	0.24675	14669	0.01531	0.162	.....
CFAP97	4	0.39660	0.87712	0.21279	0.27073	0.08092	14669	0.01532	0.162	.....
SGSH	17	0.92907	0.04595	0.32468	0.12288	0.22278	14669	0.01533	0.162	.....
RNF135	17	0.54845	NA	0.28072	0.25275	0.06893	13691	0.01534	0.162	.?...
CRMP1	4	0.61538	0.44056	0.25974	0.30070	0.07892	14669	0.01534	0.162	.....
FYN	6	0.41558	0.35065	0.77822	0.01698	0.33566	14669	0.01535	0.162	.....
SLC11A2	12	0.00130	0.85215	0.76723	0.47453	0.19481	14669	0.01535	0.162	.....
COPRS	17	0.23576	0.32168	0.86014	0.40959	0.06593	14669	0.01535	0.162	.....
ADSL	22	0.50949	0.55644	0.91908	0.10689	0.08691	14669	0.01536	0.162	.....
LINC00202-2	10	0.03896	0.16084	0.69530	NA	0.16583	11265	0.01537	0.162	...?.
MINA	3	0.88112	0.12388	0.04995	0.56543	0.09590	14669	0.01537	0.162	.....
LHX5-AS1	12	0.61638	0.56643	0.29870	0.56843	0.03097	14669	0.01537	0.162	.....
LINC01210	3	0.62038	0.43357	0.83916	0.04895	0.14785	14669	0.01537	0.162	.....
TMEM254	10	0.89111	0.57043	0.08392	NA	0.03996	11265	0.01538	0.162	...?.
ZNF783	7	0.57542	0.28771	0.32567	0.11289	0.18781	14669	0.0154	0.162	.....
PI4KB	1	0.57642	0.23576	0.86114	0.11489	0.12288	14669	0.01543	0.162	.....
EDN3	20	0.66833	NA	0.11189	0.18482	0.10889	13691	0.01543	0.162	.?...
MORC2	22	0.94106	0.24975	0.34066	0.51449	0.03497	14669	0.01543	0.162	.....
CA5A	16	0.18382	0.35165	0.66034	0.01299	0.52947	14669	0.01544	0.162	.....
LOC105378349	10	0.87113	0.67532	0.16583	0.98402	0.00940	14669	0.01545	0.162	.....
SMURF1	7	0.05495	0.64436	0.12388	0.16284	0.37063	14669	0.01545	0.162	.....
SCEL	13	0.38262	0.60040	0.50649	0.04196	0.23676	14669	0.01546	0.162	.....
PCCB	3	0.35365	0.90609	0.82318	NA	0.01798	11265	0.01547	0.162	...?.
TREML5P	6	0.05295	0.46753	0.05395	0.29471	0.37063	14669	0.01547	0.162	.....
NPTN	15	0.82517	0.76224	0.95704	0.00590	0.20579	14669	0.01547	0.162	.....
EXO1	1	0.34765	0.90509	0.32468	0.16284	0.10789	14669	0.01547	0.162	.....
FBN2	5	0.19980	0.10290	0.80819	0.18881	0.21878	14669	0.01548	0.162	.....
TTC8	14	0.24476	0.14785	0.89710	NA	0.06693	11265	0.01549	0.162	...?.
DOLPP1	9	0.29770	NA	0.11588	0.04895	0.35265	13691	0.01549	0.162	.?...
LINC00612	12	0.73127	0.00570	0.77023	0.08292	0.34765	14669	0.0155	0.162	.....
TEX35	1	0.28472	0.03896	0.76124	0.04695	0.47852	14669	0.0155	0.162	.....
TEKT2	1	0.30370	0.04196	0.21578	0.13886	0.44555	14669	0.0155	0.162	.....
DRC3	17	0.25574	0.39760	0.30769	0.02498	0.48052	14669	0.01554	0.162	.....
PRLHR	10	0.60739	0.83816	0.94206	0.34466	0.01898	14669	0.01555	0.162	.....
NEUROD1	2	0.62038	0.09890	0.21179	0.56543	0.08791	14669	0.01555	0.162	.....
C1orf145	1	0.28971	0.52947	0.73127	0.18981	0.10090	14669	0.01556	0.162	.....
SAFB2	19	0.23177	0.49151	0.42757	0.61638	0.05195	14669	0.01556	0.162	.....
FLOT1	6	0.08192	0.34066	0.06793	0.19181	0.44655	14669	0.01556	0.162	.....
PDE4C	19	0.27073	0.56743	0.63736	0.45554	0.04895	14669	0.01556	0.162	.....
RASGRP1	15	0.65634	0.35964	0.20979	0.24775	0.10889	14669	0.01557	0.162	.....
ZSWIM1	20	0.40759	0.27273	0.33666	0.28771	0.12488	14669	0.01557	0.162	.....
TTC9C	11	0.72128	0.45355	0.37862	0.37562	0.04595	14669	0.01558	0.162	.....
G6PC3	17	0.32368	0.42957	0.40260	0.12987	0.18681	14669	0.01559	0.162	.....
TRAPPC12	2	0.19580	0.00600	0.70030	0.39560	0.27672	14669	0.01559	0.162	.....

NOTCH4	6	0.97203	0.33467	0.32967	0.11089	0.11788	14669	0.01559	0.162	.....
PADI6	1	0.22078	0.20080	0.86913	0.01698	0.48452	14669	0.0156	0.162	.....
LSM11	5	0.58242	0.65435	0.17383	0.77922	0.02398	14669	0.01561	0.162	.....
SHOX2	3	0.48951	0.16683	0.34665	0.34965	0.11389	14669	0.01561	0.162	.....
DCLRE1B	1	0.11489	0.94306	0.13387	0.87013	0.04695	14669	0.01562	0.162	.....
CNP	17	0.85514	0.69530	0.50350	0.00170	0.42757	14669	0.01564	0.162	.....
CYP2E1	10	0.29570	0.55744	0.70130	0.02797	NA	6657	0.01564	0.162	....?
WRN	8	0.20380	0.03397	0.65235	0.58541	0.13586	14669	0.01565	0.162	.....
KRTAP12-3	21	0.75624	0.45355	0.11788	0.17682	0.13686	14669	0.01565	0.162	.....
INO80	15	0.30070	0.13986	0.24376	0.10889	0.37163	14669	0.01565	0.162	.....
ENO3	17	0.77023	0.87912	0.42557	0.23776	0.03896	14669	0.01566	0.162	.....
FHIT	3	0.00470	0.40060	0.86813	NA	NA	3253	0.01568	0.162	...??
GPX7	1	0.00150	0.15185	0.11189	0.35365	0.75824	14669	0.01568	0.162	.....
PMEL	12	0.05994	NA	0.64635	0.17982	0.17982	13691	0.01569	0.162	.?...
SVOP	12	0.17882	0.18082	0.86913	0.67033	0.05694	14669	0.01569	0.162	.....
FKBP11	12	0.17582	0.12188	0.66134	0.73427	0.07293	14669	0.01571	0.162	.....
ZNF280B	22	0.13487	NA	0.26074	NA	0.08192	10287	0.01573	0.162	.?.?.
ZNF57	19	0.73227	0.05594	0.76224	0.08591	0.21578	14669	0.01573	0.162	.....
MSH4	1	0.88312	0.47353	0.13586	0.27473	0.07892	14669	0.01575	0.162	.....
MUC21	6	0.11089	0.18282	0.89610	0.07193	0.36064	14669	0.01575	0.162	.....
SRR	17	0.74026	0.88412	0.85415	0.06194	0.07193	14669	0.01576	0.162	.....
MELK	9	0.10689	0.25375	0.61039	0.09091	0.35764	14669	0.01578	0.162	.....
FAM109A	12	0.08791	NA	0.99600	0.10989	0.16384	13691	0.01579	0.162	.?...
ACAT2	6	0.27173	0.96703	0.26274	0.17083	0.12587	14669	0.01579	0.162	.....
SCAF1	19	0.75025	NA	0.81419	0.06094	0.08492	13691	0.0158	0.162	.?...
HS3ST1	4	0.52547	0.07193	0.14186	0.07792	0.45854	14669	0.01581	0.162	.....
OR1N1	9	0.55245	0.42957	0.30070	0.10689	0.17682	14669	0.01581	0.162	.....
SH2B2	7	0.12787	0.46553	0.25375	0.86913	0.05894	14669	0.01583	0.162	.....
FAM117B	2	0.99900	0.22478	0.02597	0.26573	0.16484	14669	0.01583	0.162	.....
MASP1	3	0.05994	0.08192	0.10290	0.05594	0.93107	14669	0.01583	0.162	.....
SON	21	0.37063	0.19281	0.37463	0.40360	0.10989	14669	0.01583	0.162	.....
PRR19	19	0.06693	0.88012	0.58142	0.18182	0.16484	14669	0.01585	0.162	.....
KCNRG	13	0.91508	0.12987	0.14585	0.19580	0.16983	14669	0.01585	0.162	.....
FAM222A-AS1	12	0.39760	NA	0.06194	0.13387	0.22577	13691	0.01585	0.162	.?...
SMCHD1	18	0.84515	0.17283	0.85814	0.58442	0.02198	14669	0.01585	0.162	.....
STRADA	17	0.95604	0.78521	0.14785	0.11289	0.10090	14669	0.01586	0.162	.....
LPCAT3	12	0.30569	0.44855	0.12887	0.03896	0.46753	14669	0.01586	0.162	.....
IRG1	13	0.02597	0.70529	0.08392	0.37562	0.29271	14669	0.01586	0.162	.....
TRIM14	9	0.12388	0.54246	0.96903	0.07592	0.21778	14669	0.01587	0.162	.....
FASTKD2	2	0.03197	0.88412	0.35764	0.03596	0.52148	14669	0.01588	0.162	.....
IRF2	4	0.68731	0.63736	0.84915	0.00840	0.25075	14669	0.01589	0.162	.....
SUPT5H	19	0.16783	0.38561	0.10889	0.16883	0.33766	14669	0.0159	0.162	.....
MTIF2	2	0.16384	0.03996	0.44256	0.49451	0.20679	14669	0.0159	0.162	.....
TBX4	17	0.95005	0.50749	0.63337	0.33966	0.02597	14669	0.01591	0.162	.....
OR1M1	19	0.91109	NA	0.46953	0.17183	0.04795	13691	0.01593	0.162	.?...
OR5L2	11	0.16084	0.18681	0.31868	0.49251	0.15085	14669	0.01594	0.162	.....
UBAC2-AS1	13	0.03097	0.88911	0.18581	0.06094	0.51548	14669	0.01594	0.162	.....
P4HA3	11	0.59041	0.64935	0.51049	NA	0.02398	11265	0.01595	0.162	...?.
MIR5695	6	0.70529	0.03297	0.20280	0.97303	0.05495	14669	0.01595	0.162	.....
SIGLEC9	19	0.16783	0.18382	0.07093	0.02298	0.88511	14669	0.01595	0.162	.....
ADHFE1	8	0.04995	0.22577	0.76324	0.03796	0.57542	14669	0.01596	0.162	.....
ZNF778	16	0.01698	0.25774	0.30969	0.18681	0.50450	14669	0.01596	0.162	.....
TTC33	5	0.47053	0.19481	0.71828	0.37363	0.07093	14669	0.01596	0.162	.....
LOC284344	19	0.17982	NA	0.64835	0.38861	0.06094	13691	0.01597	0.162	.?...
ROPN1L-AS1	5	NA	NA	NA	0.01598	NA	3404	0.01598	0.162	???.?
CSRP3	11	0.72028	0.77323	0.10989	0.39061	0.05395	14669	0.016	0.162	.....
RILPL1	12	0.82318	0.03796	0.01998	0.13287	0.48252	14669	0.016	0.162	.....
FGL1	8	0.05794	0.72827	0.42458	NA	0.09391	11265	0.01601	0.162	...?.
DARS	2	0.01998	0.01998	0.86214	0.63536	0.27173	14669	0.01603	0.162	.....
DNMT3B	20	0.04496	0.15185	0.16883	0.75425	0.20779	14669	0.01604	0.162	.....
TGFB1	19	0.18382	0.63736	0.52547	0.07193	0.24875	14669	0.01604	0.162	.....
LINC00934	12	0.84815	0.51948	0.14585	NA	0.03796	11265	0.01605	0.162	...?.
LINC01425	21	0.57542	0.89710	0.63636	0.00330	0.36464	14669	0.01607	0.162	.....
C10orf90	10	0.11788	0.32168	0.18482	0.19381	0.33067	14669	0.01607	0.162	.....
CACNA1C-AS2	12	0.08292	0.14985	0.13087	0.01898	0.99900	14669	0.01609	0.162	.....
PIGX	3	0.30969	0.62537	0.06893	0.63636	0.07892	14669	0.01609	0.162	.....
SCHIP1	3	0.73726	0.30470	0.04695	NA	0.08791	11265	0.01609	0.162	...?.
ZNF682	19	0.01898	0.22278	0.87113	0.11389	0.44855	14669	0.01611	0.162	.....
UBN2	7	0.21878	0.79221	0.30370	0.24675	0.11988	14669	0.01612	0.162	.....

CENPL	1	0.44056	0.38661	0.60240	0.03097	0.29271	14669	0.01612	0.162	.....
POU6F2	7	0.23976	0.04695	NA	0.61838	0.09990	13818	0.01613	0.162	..?..
CD209	19	0.06394	NA	0.31868	0.67233	0.07792	13691	0.01615	0.162	.?...
PDK2	17	0.00072	0.48152	0.22677	0.18781	0.74126	14669	0.01617	0.163	.....
C9orf62	9	0.29171	0.72128	0.82917	NA	0.02697	11265	0.01619	0.163	...?.
ZBTB4	17	0.76324	0.00400	0.57443	0.12188	0.35265	14669	0.0162	0.163	.....
S100A14	1	0.19580	0.80420	0.78921	0.23676	0.07792	14669	0.01621	0.163	.....
DIRC3-AS1	20	NA	0.73127	NA	0.01099	NA	4382	0.01623	0.163	?.?.?
FAM95C	9	0.43856	NA	0.27872	NA	0.04096	10287	0.01624	0.163	.?.?.
MVK	12	0.20180	0.82817	0.40959	0.00480	0.59540	14669	0.01625	0.163	.....
LAMP3	3	0.33966	0.62537	0.15784	0.40160	0.09491	14669	0.0163	0.163	.....
PVRIG2P	7	0.53047	0.81119	0.03596	0.11289	0.23576	14669	0.0163	0.163	.....
C19orf66	19	0.38062	NA	0.46154	0.39660	0.04795	13691	0.01631	0.163	.?...
FOXO6	1	0.14286	0.35065	0.32667	0.04995	0.47852	14669	0.01631	0.163	.....
LOC100129307	13	0.38462	0.72627	0.50250	0.51748	0.03397	14669	0.01632	0.163	.....
ZSCAN20	1	0.88312	0.76024	0.90210	0.10290	0.04795	14669	0.01632	0.163	.....
NGF	1	0.42058	0.63337	0.00570	0.10589	0.43057	14669	0.01633	0.163	.....
PSIP1	9	0.09291	0.59740	0.28172	0.02597	0.57942	14669	0.01633	0.163	.....
CHST13	3	0.77522	0.24875	0.82817	0.68132	0.01698	14669	0.01635	0.163	.....
GDF11	12	0.77622	0.12488	0.40959	0.06793	0.25674	14669	0.01635	0.163	.....
DIMT1	5	0.19381	0.97902	0.61738	0.43856	0.04296	14669	0.01635	0.163	.....
ZNF891	12	0.50150	0.19381	0.07592	NA	0.12587	11265	0.01637	0.163	...?.
NCKIPSD	3	0.47952	0.06693	0.96004	0.09790	0.22478	14669	0.0164	0.163	.....
DAAM2	6	0.28472	0.05594	0.71528	NA	0.10789	11265	0.01641	0.163	...?.
LINC00544	13	0.48751	0.80320	0.05195	NA	0.06693	11265	0.01641	0.163	...?.
TUB	11	0.02098	0.61339	0.16384	0.61638	0.18781	14669	0.01641	0.163	.....
APCDD1L	20	0.16084	0.00930	0.12388	0.09790	0.86813	14669	0.01644	0.163	.....
RPL23AP64	11	0.49950	0.80020	0.16783	0.66933	0.03197	14669	0.01645	0.163	.....
C5AR1	19	0.63836	NA	0.26973	0.11089	0.12388	13691	0.01645	0.163	.?...
KLK8	19	0.93407	0.81818	0.47453	0.02398	0.15385	14669	0.01645	0.163	.....
PROCR	20	0.04695	0.91708	0.10589	0.36064	0.21179	14669	0.01646	0.163	.....
DOC2GP	11	1.00000	0.05095	0.07393	0.39760	0.15285	14669	0.01647	0.163	0....
NME8	7	0.20180	0.07692	0.98601	0.65634	0.07293	14669	0.01647	0.163	.....
PISD	22	0.22378	0.77223	0.93806	0.53147	0.02797	14669	0.01648	0.163	.....
LINC00479	21	0.30370	0.18282	0.32168	0.61638	0.08991	14669	0.01649	0.163	.....
MSTO1	1	NA	0.27273	NA	0.03297	NA	4382	0.0165	0.163	?.?.?
FXYD3	19	0.13387	0.11988	0.77822	0.11888	0.33666	14669	0.0165	0.163	.....
PIGT	20	0.01499	0.60539	0.77223	0.33866	0.18681	14669	0.0165	0.163	.....
MPZ	1	0.21578	0.84216	0.29670	0.00490	0.63237	14669	0.01651	0.163	.....
LINC01492	9	0.17383	0.59241	0.10290	0.64535	0.09890	14669	0.01657	0.164	.....
ASGR1	17	0.59940	0.15085	0.15584	NA	0.09790	11265	0.01658	0.164	...?.
TRIM34	11	0.36164	0.00390	0.01898	0.43956	0.56144	14669	0.01658	0.164	.....
LOC105375429	7	0.19880	0.51149	0.17582	0.65035	0.08292	14669	0.01658	0.164	.....
TMEM102	17	0.09990	0.16084	0.74226	0.01798	0.68332	14669	0.01661	0.164	.....
OR1S1	11	0.16783	0.22278	0.03397	NA	0.25375	11265	0.01662	0.164	...?.
LIF	22	0.14186	0.53447	0.21479	0.46054	0.12887	14669	0.01664	0.164	.....
FOSB	19	0.01598	0.22977	0.67732	0.73427	0.14785	14669	0.01666	0.164	.....
MIR5694	10	0.27872	0.04396	0.26274	NA	NA	3253	0.01666	0.164	...??
CCDC71	3	0.27772	0.81918	0.85514	0.21778	0.06593	14669	0.01667	0.164	.....
BCHE	3	0.72328	0.42857	0.60240	0.16583	0.08192	14669	0.01669	0.164	.....
ZNF816	19	0.38362	0.39660	0.76623	0.19880	0.10090	14669	0.0167	0.164	.....
LINC01184	5	0.13387	0.90909	0.31469	0.12188	0.21878	14669	0.01672	0.164	.....
SURF2	9	0.28372	0.22378	0.22178	0.41359	0.15285	14669	0.01672	0.164	.....
LINC00363	13	0.01099	0.85914	0.46154	0.21578	NA	6657	0.01673	0.164	....?
ALKBH6	19	0.96703	NA	0.12288	0.02597	0.24775	13691	0.01674	0.164	.?...
TGFBR1	9	0.39760	0.92108	0.33267	0.00699	0.44056	14669	0.01677	0.164	.....
VPS9D1	16	0.29670	0.99800	0.27572	0.66434	0.03097	14669	0.01677	0.164	.....
EED	11	0.21978	0.09191	0.98302	NA	0.08492	11265	0.01677	0.164	...?.
TMEM30C	3	0.41359	0.89610	0.90609	0.15884	0.05894	14669	0.01678	0.164	.....
ZBTB45	19	0.89610	NA	0.54346	0.13387	0.05794	13691	0.01678	0.164	.?...
PDX1-AS1	13	0.13786	0.45255	0.54645	0.70430	0.05694	14669	0.01679	0.164	.....
IER2	19	0.18482	0.36963	0.95405	0.06194	0.25974	14669	0.01681	0.165	.....
C14orf177	14	0.39361	0.12288	0.40759	0.12887	0.28172	14669	0.01682	0.165	.....
LRRC14B	5	0.83816	0.20679	0.27772	0.35564	0.07992	14669	0.01684	0.165	.....
WWP1	8	0.40959	0.44855	NA	0.03796	0.24076	13818	0.01687	0.165	..?..
SMC6	2	0.86613	0.17682	0.74825	0.00780	0.39461	14669	0.01688	0.165	.....
RREB1	6	0.49650	0.89111	0.16783	0.08492	0.18482	14669	0.01688	0.165	.....
P3H1	1	0.43656	0.99800	0.52547	0.16284	0.07393	14669	0.01689	0.165	.....
ZAR1	4	0.78621	0.18082	0.01390	0.64336	0.12188	14669	0.0169	0.165	.....

SRSF4	1	0.12987	0.70130	0.49151	0.31868	0.11289	14669	0.01691	0.165	.....
SDR39U1	14	0.32567	NA	0.22977	0.01998	0.42358	13691	0.01691	0.165	.?...
RGPD1	2	0.71129	NA	0.61838	0.13087	0.06893	13691	0.01693	0.165	.?...
OR5W2	11	0.87013	0.77223	0.06793	0.52747	0.03996	14669	0.01695	0.165	.....
STMN1	1	0.77822	0.93706	0.41159	0.55544	0.01499	14669	0.01695	0.165	.....
UTS2R	17	NA	0.01698	NA	NA	NA	978	0.01698	0.165	?.???
MT1DP	16	0.32567	0.90609	0.63237	0.05994	0.16384	14669	0.01698	0.165	.....
WDR49	3	0.87013	0.88112	0.04795	0.02697	0.30669	14669	0.01699	0.165	.....
ARRB1	11	0.05694	0.41858	0.91409	0.08991	0.31568	14669	0.01701	0.165	.....
XAB2	19	0.75624	0.81019	0.11389	0.21479	0.09091	14669	0.01701	0.165	.....
ANKRD62	18	0.00046	0.03397	0.66833	0.60140	0.59540	14669	0.01702	0.165	.....
ADAM3A	20	NA	0.01399	0.35964	NA	0.13686	9841	0.01702	0.165	?..?.
RAB11A	15	0.08492	0.23077	0.80719	0.41159	0.14186	14669	0.01703	0.165	.....
DSCR3	21	0.07792	0.25674	0.18881	0.68032	0.16384	14669	0.01703	0.165	.....
BEST1	11	0.65734	0.27073	0.35564	0.30769	0.09091	14669	0.01704	0.165	.....
GJA3	13	0.43856	0.54046	0.20779	0.43556	0.07692	14669	0.01705	0.165	.....
LOC101927415	12	0.84715	0.06494	0.08891	0.20579	0.26174	14669	0.01707	0.165	.....
RNF34	12	0.30170	0.31968	0.40060	0.01898	0.52148	14669	0.01708	0.165	.....
PYY	17	0.37862	0.38861	0.88012	0.31668	0.06394	14669	0.01709	0.165	.....
CTNNAL1	9	0.97103	0.45455	0.19481	0.14286	0.11588	14669	0.0171	0.165	.....
COL4A2	13	0.23676	0.04695	0.19680	NA	0.22478	11265	0.0171	0.165	...?.
ZNF124	1	0.25574	0.85315	0.02198	0.10989	0.37163	14669	0.01712	0.165	.....
ODF2	9	0.80819	0.81119	0.11489	0.43257	0.04396	14669	0.01713	0.165	.....
ZNF888	19	0.61139	NA	0.41059	0.78721	0.01399	13691	0.01714	0.165	.?...
RASGEF1A	10	0.91508	0.45554	0.55145	NA	0.01998	11265	0.01714	0.165	...?.
KIAA1522	1	0.08691	0.18482	0.35864	0.18581	0.37962	14669	0.01715	0.165	.....
SPINK13	5	0.38561	0.24775	0.93506	0.75624	0.02597	14669	0.01715	0.165	.....
C1orf52	1	0.69231	0.85714	0.72028	0.19580	0.04196	14669	0.0172	0.166	.....
MMAA	4	0.28971	0.09590	0.46154	0.15584	0.30270	14669	0.0172	0.166	.....
G3BP1	5	0.91508	0.59740	0.61838	0.20480	0.04496	14669	0.01721	0.166	.....
LILRA6	20	NA	NA	0.15684	NA	0.04096	8863	0.01722	0.166	??.?.
LONP2	16	0.47353	0.91908	0.51049	0.20180	0.06593	14669	0.01722	0.166	.....
C7orf49	7	0.03497	0.19580	0.48751	NA	0.20879	11265	0.01723	0.166	...?.
TSLP	5	0.13886	0.86613	0.73327	0.51548	0.04496	14669	0.01723	0.166	.....
RASSF7	11	0.08691	0.53347	0.00430	0.49251	0.36563	14669	0.01726	0.166	.....
HCFC2	12	0.14785	0.06094	0.12787	0.87013	0.17582	14669	0.01728	0.166	.....
CREG1	1	0.34565	0.77722	0.56344	0.58841	0.02897	14669	0.01728	0.166	.....
DNAH6	2	NA	0.22677	0.94006	0.03696	0.20979	13245	0.01729	0.166	?....
TNFRSF8	1	0.53546	0.72128	0.75524	0.35664	0.03297	14669	0.0173	0.166	.....
LOC100289473	20	0.27872	NA	0.10090	0.79620	0.05095	13691	0.0173	0.166	.?...
LRIG1	3	0.33067	0.28771	0.21179	0.29371	0.17782	14669	0.01731	0.166	.....
EFCAB6	22	0.39960	0.47253	0.88711	0.38761	0.04496	14669	0.01731	0.166	.....
PLPP1	5	0.08192	0.60140	0.95704	NA	0.05894	11265	0.01731	0.166	...?.
DCUN1D1	3	0.72527	0.96204	0.36563	0.30070	0.03696	14669	0.01731	0.166	.....
ZNF546	19	0.02997	0.54346	0.13686	0.18382	0.45854	14669	0.01734	0.166	.....
PUS7	7	0.00999	0.62238	0.29870	0.84515	0.13487	14669	0.01735	0.166	.....
CSGALNACT2	10	0.52448	0.19780	0.38362	NA	0.06893	11265	0.01735	0.166	...?.
SERPINB8	18	0.79620	0.48851	0.16983	0.11489	0.16384	14669	0.01739	0.166	.....
NFE2	12	0.54346	NA	0.47453	0.50450	0.02897	13691	0.01742	0.166	.?...
KIF4B	5	0.37662	0.00710	0.40060	0.28372	0.35165	14669	0.01742	0.166	.....
GEMIN2	14	0.86513	0.11089	0.92008	NA	0.03097	11265	0.01745	0.166	...?.
ANP32B	9	0.16084	NA	0.52448	0.63137	0.04595	13691	0.01746	0.166	.?...
ANKRD6	6	0.74525	0.09291	0.82917	NA	0.04296	11265	0.01746	0.166	...?.
NCALD	8	0.01798	0.22078	0.64236	0.07992	0.61838	14669	0.01746	0.166	.....
RXFP1	4	0.83017	0.24675	0.97702	0.04795	0.15485	14669	0.01746	0.166	.....
SLC25A15	13	0.49451	0.83417	0.45255	NA	0.02697	11265	0.01748	0.166	...?.
SLC25A32	8	0.49351	0.58142	0.85914	0.07093	0.13387	14669	0.01749	0.166	.....
SGMS2	4	0.68332	0.94905	0.79021	0.09091	0.06693	14669	0.0175	0.166	.....
STAC2	17	0.31369	NA	0.96603	0.00340	0.44855	13691	0.01751	0.166	.?...
PPP1R12B	1	0.36364	0.05894	0.35265	0.66833	0.11189	14669	0.01751	0.166	.....
C20orf203	20	0.82617	0.67932	0.04695	0.21778	0.12687	14669	0.01752	0.166	.....
A2M-AS1	12	0.78022	0.01399	0.59940	0.28472	0.17083	14669	0.01752	0.166	.....
ACOT6	14	0.44555	0.10589	0.99301	NA	0.05395	11265	0.01753	0.166	...?.
FAM174A	5	0.71129	0.27073	0.73227	0.29171	0.06094	14669	0.01755	0.166	.....
RGS1	1	0.37562	NA	0.12388	0.00940	0.59241	13691	0.0176	0.167	.?...
SPACA3	17	0.10789	0.26973	0.64935	0.75824	0.07193	14669	0.01761	0.167	.....
TLX1NB	10	0.65934	0.83716	0.02098	0.27572	0.13586	14669	0.01761	0.167	.....
LOC105370177	13	0.03297	0.80220	0.12587	NA	0.18382	11265	0.01762	0.167	...?.
INSR	19	0.40959	0.22577	0.37962	NA	0.07892	11265	0.01763	0.167	...?.

LINC01566	16	NA	NA	0.15385	0.56543	0.03596	12267	0.01763	0.167	??...
DSCAS	18	0.56044	0.57443	0.18282	0.13586	0.17283	14669	0.01764	0.167	.....
ZWINT	10	0.47153	0.97303	0.23177	0.01998	0.32268	14669	0.01765	0.167	.....
TTC17	11	0.02498	0.04496	0.62238	NA	0.31369	11265	0.01765	0.167	...?.
LSM12	17	0.54845	0.69031	0.41658	0.14286	0.10989	14669	0.01765	0.167	.....
OR52J3	11	0.47652	0.98402	0.72927	0.32168	0.03197	14669	0.01765	0.167	.....
CLSPN	1	0.93407	0.80519	0.08292	0.05794	0.19780	14669	0.01768	0.167	.....
DAG1	3	NA	0.16583	0.77522	0.20180	0.10090	13245	0.01769	0.167	?....
MRPL35	2	0.08791	0.40959	0.51948	0.41259	0.14486	14669	0.01769	0.167	.....
CCDC188	22	0.23876	0.64535	0.56244	0.79920	0.02797	14669	0.01769	0.167	.....
LINC00690	3	0.47652	0.22478	0.11688	0.81219	0.06893	14669	0.01771	0.167	.....
SCD5	4	0.92907	0.45654	0.74725	0.15584	0.05994	14669	0.01771	0.167	.....
CPA5	7	0.10490	0.99700	0.83217	0.12488	0.14585	14669	0.01772	0.167	.....
SRCAP	16	0.51848	NA	0.31668	0.07592	0.17882	13691	0.01773	0.167	.?...
CAT	11	0.76623	0.05694	0.38761	NA	0.08192	11265	0.01773	0.167	...?.
PNMA1	14	0.09990	0.39261	0.24476	NA	0.14985	11265	0.01774	0.167	...?.
GTF2H3	12	0.76024	0.01299	0.24775	0.43257	0.18581	14669	0.01775	0.167	.....
LOC101929698	20	0.34765	0.05295	0.80020	0.91908	0.04695	14669	0.01776	0.167	.....
RAB4A	1	0.10689	0.14486	0.52647	0.13487	0.40260	14669	0.01778	0.167	.....
LPP	3	0.68731	0.00370	NA	0.53746	0.12687	13818	0.01779	0.167	..?..
CYP2U1	4	0.50849	0.23776	0.75225	0.03297	0.30669	14669	0.01781	0.167	.....
CST7	20	0.07193	0.37363	0.17982	0.36464	0.26773	14669	0.01782	0.167	.....
PPFIA4	1	0.44755	0.98302	0.44156	0.02697	0.23576	14669	0.01782	0.167	.....
SCEL-AS1	13	0.67832	0.81219	0.41159	0.01698	0.26074	14669	0.01782	0.167	.....
FAM98B	15	0.55345	0.56843	0.23277	0.04396	0.29171	14669	0.01784	0.167	.....
KLHL10	17	0.30669	0.90010	0.98501	0.04695	0.15285	14669	0.01784	0.167	.....
LOC100129518	6	0.41459	0.90010	0.35065	0.08292	0.16683	14669	0.01786	0.167	.....
GNAS-AS1	20	0.11788	0.27073	0.43956	0.26074	0.23976	14669	0.01787	0.167	.....
ABCC1	16	0.83217	0.66633	0.79620	NA	0.01299	11265	0.01787	0.167	...?.
LRRC37A8P	17	0.21878	0.57542	0.33766	0.63337	0.06194	14669	0.01788	0.167	.....
MMP16	8	0.11489	0.70430	0.32667	NA	0.09091	11265	0.0179	0.167	...?.
HIPK1	1	0.44356	0.98302	0.43756	0.63337	0.02098	14669	0.01793	0.167	.....
HLA-DOA	6	0.66533	0.97602	0.29670	NA	0.02298	11265	0.01793	0.167	...?.
THOC1	18	0.56943	0.26873	0.42757	0.39461	0.07792	14669	0.01794	0.167	.....
GCKR	2	0.04795	0.36763	0.63137	0.41558	0.17183	14669	0.01796	0.167	.....
VPS26B	11	0.30170	0.78621	0.48152	0.09590	0.17483	14669	0.01797	0.167	.....
NKAIN3	8	0.05295	0.16983	NA	NA	NA	2402	0.01798	0.167	..???
IKZF3	17	0.93906	0.38062	0.82617	0.05794	0.11988	14669	0.01799	0.167	.....
EXTL1	1	0.91109	0.04496	0.15984	0.32967	0.17383	14669	0.01799	0.167	.....
EIF5A	17	0.31968	0.11788	0.34965	NA	0.12488	11265	0.01799	0.167	...?.
LOC283440	12	0.89910	NA	0.39960	0.06094	0.12288	13691	0.018	0.167	.?...
SLC7A5	16	0.54446	0.18282	0.95005	0.42857	0.05195	14669	0.018	0.167	.....
CMYA5	5	0.51349	0.08292	0.32567	0.15485	0.28372	14669	0.018	0.167	.....
LINC01098	4	0.09091	0.54545	NA	0.80819	0.04296	13818	0.01801	0.167	..?..
ZNF781	19	0.75924	0.02198	0.46454	0.25874	0.19481	14669	0.01801	0.167	.....
AP3B1	5	0.02198	0.56843	0.71928	NA	0.12787	11265	0.01801	0.167	...?.
CCPG1	15	0.58841	0.90809	0.13087	NA	0.04096	11265	0.01801	0.167	...?.
CKAP2	13	0.02697	0.13087	0.56943	0.11588	0.59640	14669	0.01803	0.167	.....
EPHA3	3	0.93107	0.57143	0.31369	0.34665	0.04296	14669	0.01803	0.167	.....
ABCC10	6	0.86813	0.10390	0.27473	0.17882	0.18382	14669	0.01805	0.167	.....
VNN1	6	0.09191	0.96903	0.93806	NA	0.03996	11265	0.01806	0.167	...?.
OR51Q1	11	0.30070	0.54945	0.68232	0.71828	0.02897	14669	0.01811	0.168	.....
ARHGEF1	19	0.75724	NA	0.83716	0.22977	0.03497	13691	0.01813	0.168	.?...
ZNF326	1	0.13686	0.67732	0.44955	0.21479	0.17083	14669	0.01814	0.168	.....
ATG16L2	11	0.23077	0.09790	0.74326	0.72927	0.07493	14669	0.01815	0.168	.....
HAAO	2	0.14086	0.21578	0.69431	0.07692	0.38861	14669	0.01816	0.168	.....
KPNA4	3	0.00510	0.90310	0.44555	0.56643	0.17682	14669	0.01817	0.168	.....
TFB2M	1	0.19580	0.10390	0.09690	0.27373	0.40759	14669	0.01817	0.168	.....
CAND2	3	0.43157	0.57742	0.63836	0.21379	0.08791	14669	0.01817	0.168	.....
TCEB3B	18	0.35764	0.29570	0.42557	NA	0.07393	11265	0.01821	0.168	...?.
C17orf74	17	0.19780	0.84615	0.67632	0.55644	0.03896	14669	0.01822	0.168	.....
LOC100506869	12	0.66833	0.80120	0.89411	0.77622	0.00699	14669	0.01823	0.168	.....
LOC101927045	14	0.59540	0.70230	0.55744	0.01299	0.30470	14669	0.01824	0.168	.....
G3BP2	4	0.72627	0.64436	0.31968	0.24675	0.07093	14669	0.01825	0.168	.....
HMCES	3	0.04895	0.88911	0.82917	0.02797	0.41658	14669	0.01825	0.168	.....
LOC100507250	12	0.19081	0.28272	0.09491	0.25375	0.32767	14669	0.01826	0.168	.....
NUMB	14	0.81718	0.06893	0.85315	NA	0.04496	11265	0.01828	0.168	...?.
MCM8-AS1	20	0.56044	0.43357	0.13786	0.23477	0.15684	14669	0.01829	0.168	.....
PLCE1	10	0.67133	0.08392	0.05994	NA	0.16084	11265	0.01829	0.168	...?.

ST3GAL5-AS1	2	0.13387	0.38162	0.26474	0.13087	0.36464	14669	0.01831	0.168	.....
LGALS13	19	0.03197	0.44555	0.98102	0.01898	0.62837	14669	0.01834	0.168	.....
SNTB2	16	0.07992	0.82418	0.21479	NA	0.11489	11265	0.01834	0.168	...?.
IDH3B	20	0.23976	0.09091	0.47852	0.25475	0.25774	14669	0.01835	0.168	.....
GRIP1	12	0.02797	0.69730	0.40360	NA	0.14386	11265	0.01837	0.168	...?.
CHMP7	8	0.03996	0.81219	0.20380	NA	0.15185	11265	0.01837	0.168	...?.
CALCRL	2	NA	0.04296	0.21179	0.05794	0.49550	13245	0.01838	0.168	?....
LOC100287072	17	0.47552	NA	0.40659	0.14086	0.12088	13691	0.01838	0.168	.?...
ANKRD36B	2	0.88611	0.92507	0.88012	0.75924	0.00440	14669	0.01839	0.168	.....
TCN2	22	0.02398	0.39960	0.71229	0.16484	0.35864	14669	0.01841	0.168	.....
C17orf64	17	0.00490	NA	0.38661	0.13187	0.52847	13691	0.01843	0.168	.?...
CTSV	9	0.78921	NA	0.12288	0.96004	0.01499	13691	0.01843	0.168	.?...
LOC105369739	12	0.18382	0.62737	0.85514	NA	0.04496	11265	0.01844	0.168	...?.
FAM26D	6	0.13886	0.86713	0.34765	NA	0.07093	11265	0.01844	0.168	...?.
DLG5-AS1	10	0.87912	0.10789	0.17682	0.09990	0.29371	14669	0.01845	0.168	.....
CLIC3	9	NA	0.62537	0.90909	0.00170	0.43756	13245	0.01846	0.168	?....
ANKRD34B	5	0.63736	0.16583	0.55944	0.84216	0.02897	14669	0.01851	0.169	.....
UBE2M	19	0.82318	NA	0.31768	0.11489	0.10290	13691	0.01852	0.169	.?...
CRYBG3	3	0.34166	0.38462	0.03297	0.72727	0.11888	14669	0.01853	0.169	.....
MRPS22	3	0.94705	0.21179	0.20080	0.01199	0.50450	14669	0.01853	0.169	.....
PSD4	2	0.03796	0.62038	0.48252	0.08591	0.42957	14669	0.01854	0.169	.....
AP3B2	15	0.19680	0.53546	0.06693	0.95704	0.07293	14669	0.01854	0.169	.....
SKP2	5	0.13986	0.00999	0.24276	0.70629	0.29970	14669	0.01855	0.169	.....
SYNDIG1L	14	0.10290	0.62138	0.31469	0.88511	0.05694	14669	0.01855	0.169	.....
WDR7	18	0.32468	0.01240	NA	NA	NA	2402	0.01858	0.169	..???
ATOH8	2	0.67433	0.41558	0.46054	0.19381	0.10190	14669	0.01859	0.169	.....
IL33	9	0.22078	0.53347	0.72727	0.10190	0.20380	14669	0.0186	0.169	.....
ZNF606	19	0.44755	0.47253	0.29770	0.06194	0.29071	14669	0.01862	0.169	.....
ZNF418	19	0.62338	0.05794	0.69431	0.03097	0.43556	14669	0.01863	0.169	.....
OR5H1	3	0.33167	0.11089	0.02298	0.15984	0.57542	14669	0.01863	0.169	.....
TANGO6	16	0.86414	0.14086	0.05295	NA	0.11588	11265	0.01864	0.169	...?.
DDX11-AS1	12	0.30170	0.47752	0.57343	0.58541	0.04995	14669	0.01865	0.169	.....
ASB17	1	0.83017	0.52248	0.08791	0.30569	0.09990	14669	0.01865	0.169	.....
TMX2-CTNND1	11	0.06993	0.81718	0.65135	0.20779	0.17083	14669	0.01866	0.169	.....
CACNG1	17	0.11489	0.13786	0.68531	0.27972	0.24476	14669	0.01866	0.169	.....
MYH14	19	0.56943	0.38462	0.82617	0.06194	0.17682	14669	0.01867	0.169	.....
LOC100128593	9	0.26773	0.43556	0.70529	0.33866	0.08991	14669	0.01867	0.169	.....
CHL1	3	0.10190	0.85914	0.35564	0.24975	0.16783	14669	0.01869	0.169	.....
PDK1	2	0.87313	0.32767	0.16284	0.42657	0.07193	14669	0.01869	0.169	.....
ADAMTS17	15	0.78022	0.22478	0.56643	0.02997	NA	6657	0.0187	0.169	....?
MTRF1	13	0.68531	0.86813	0.70829	0.05195	0.11788	14669	0.01872	0.169	.....
MED9	17	0.12887	0.44855	0.27073	0.77223	0.08392	14669	0.01873	0.169	.....
PSEN1	14	0.01170	0.55045	0.37562	NA	0.21978	11265	0.01874	0.169	...?.
DEFB119	20	0.01598	NA	0.17083	0.91209	0.12388	13691	0.01874	0.169	.?...
TOMM34	20	0.03097	0.39361	0.48252	0.03397	0.71728	14669	0.01874	0.169	.....
LNX1-AS2	4	0.13786	0.37762	0.35365	0.48352	0.13686	14669	0.01875	0.169	.....
KCNA6	12	0.24476	0.82318	0.06693	NA	0.10490	11265	0.01875	0.169	...?.
POTEKP	2	0.78222	0.56543	0.09590	0.12488	0.18681	14669	0.01875	0.169	.....
NUP54	4	0.02398	0.20380	0.53646	0.09690	0.61638	14669	0.01876	0.169	.....
SPATA31E1	9	0.00300	0.60839	0.51049	0.48551	0.27972	14669	0.01876	0.169	.....
ASH1L	1	0.04096	0.21079	0.94605	0.02997	0.65734	14669	0.01879	0.169	.....
DLG1	3	0.04695	0.31968	0.38661	0.23576	0.34765	14669	0.01879	0.169	.....
LINC01499	11	0.14286	0.36663	0.64136	NA	0.08891	11265	0.01879	0.169	...?.
TMBIM1	20	NA	0.70030	0.18681	0.18382	0.11189	13245	0.0188	0.169	?....
LINC01512	6	0.64436	0.04895	0.67133	0.08192	0.30569	14669	0.0188	0.169	.....
FANCA	16	0.64735	0.68531	NA	0.55544	0.01798	13818	0.01883	0.169	..?..
RAP1GAP	1	0.40859	0.32967	0.03497	0.33267	0.24176	14669	0.01883	0.169	.....
LINC00603	20	NA	0.24675	NA	0.04096	NA	4382	0.01884	0.169	?.?.?
BEST4	1	0.17283	0.39660	0.85415	0.77922	0.03896	14669	0.01884	0.169	.....
STAG3L5P	7	0.52248	0.82617	0.04595	0.11688	0.24675	14669	0.01887	0.169	.....
PNMAL1	19	0.32867	0.96703	0.26374	0.09890	0.18981	14669	0.01887	0.169	.....
CCDC82	11	0.57642	0.47453	0.23277	0.32767	0.09590	14669	0.01887	0.169	.....
MAT2A	2	0.06893	0.61439	0.02498	0.15485	0.56444	14669	0.01888	0.169	.....
KMT5A	12	0.40260	0.43556	0.80619	0.17383	0.11289	14669	0.01888	0.169	.....
KPTN	19	0.37862	NA	0.48951	0.06094	0.21079	13691	0.01888	0.169	.?...
IRF7	11	0.78621	0.93506	0.50250	0.03996	0.13986	14669	0.01889	0.169	.....
SPATC1	20	NA	0.72527	0.26474	0.43556	0.04296	13245	0.01889	0.169	?....
GPA33	1	0.10390	0.37962	0.09890	0.03596	0.78022	14669	0.0189	0.169	.....
ZCWPW1	7	0.46553	0.89510	0.02498	0.10290	0.30370	14669	0.01892	0.169	.....

LOC101929563	9	0.23077	0.21778	0.25974	NA	0.13886	11265	0.01892	0.169	...?.
XPC	3	0.97902	0.56543	0.37762	0.00350	0.43956	14669	0.01893	0.169	.....
MYHAS	17	0.76823	0.37163	0.56543	0.31469	0.05794	14669	0.01894	0.169	.....
PRRX1	1	0.70729	0.53347	0.40959	0.04296	0.22577	14669	0.01894	0.169	.....
SLC4A4	4	0.13087	0.38462	0.08891	NA	NA	3253	0.01896	0.169	...??
REEP2	5	0.76124	0.28671	0.54146	0.24476	0.08591	14669	0.01896	0.169	.....
RASIP1	19	0.75624	0.00340	0.07393	0.11588	0.67932	14669	0.01896	0.169	.....
NOP56	20	0.68531	0.66234	0.61339	0.22977	0.05694	14669	0.01897	0.169	.....
THEM4	1	0.15984	0.97702	0.24476	0.38961	0.10290	14669	0.01901	0.169	.....
TCAM1P	17	0.86014	0.36763	0.32767	0.24775	0.08691	14669	0.01901	0.169	.....
LOC101928708	16	0.25774	0.09690	0.19980	0.60440	0.17383	14669	0.01904	0.169	.....
SPATA9	5	0.21678	0.81419	0.22977	0.72827	0.05095	14669	0.01904	0.169	.....
ALS2CL	3	0.14186	0.39860	0.49051	0.08691	0.35864	14669	0.01904	0.169	.....
RASGRP3	2	0.68132	0.33866	0.04296	0.77522	0.06893	14669	0.01906	0.169	.....
LOC101927278	10	0.14785	0.83317	0.70230	0.06494	0.24575	14669	0.01906	0.169	.....
FNDC5	1	0.03796	0.16184	0.44555	0.04895	0.78222	14669	0.01906	0.169	.....
RAPGEF3	12	0.88511	0.47153	0.60240	0.09990	0.10589	14669	0.01908	0.169	.....
DYNC1H1	14	0.29570	0.61838	0.39161	0.48851	0.06793	14669	0.0191	0.169	.....
ATP6V0B	1	0.29870	0.87512	0.05095	0.23676	0.20380	14669	0.0191	0.169	.....
LAPTM4B	8	0.41858	0.17083	0.04895	0.11089	0.50250	14669	0.01911	0.169	.....
SIDT1	3	0.59940	0.62937	0.35564	0.05894	0.21179	14669	0.01911	0.169	.....
TUBB6	18	0.97502	0.18781	0.15385	0.45055	0.08192	14669	0.01916	0.169	.....
HEBP1	12	0.09990	0.87512	0.24376	0.73327	0.06893	14669	0.01916	0.169	.....
IQGAP2	5	0.11588	0.02797	0.51449	NA	0.25674	11265	0.01917	0.169	...?.
GPR6	6	0.79021	0.03796	0.25674	0.54945	0.11588	14669	0.01917	0.169	.....
LYZ	12	0.48052	0.11888	0.34565	0.41159	0.13786	14669	0.01919	0.169	.....
ADGRG7	3	0.07692	0.90110	0.04895	0.23576	0.33467	14669	0.0192	0.169	.....
LOC102031319	10	0.29071	0.06993	0.45455	NA	0.14585	11265	0.0192	0.169	...?.
CCL13	17	0.18681	0.95704	0.98501	0.47453	0.03397	14669	0.01921	0.169	.....
ORAOV1	11	0.14985	0.18981	0.45854	0.13986	0.36863	14669	0.01921	0.169	.....
SLC27A2	15	0.40859	0.14985	0.01299	NA	0.26474	11265	0.01921	0.169	...?.
CCDC183	9	0.27173	0.64036	0.11688	0.22078	0.21778	14669	0.01921	0.169	.....
FBXO32	8	0.12088	0.15285	0.36264	NA	0.18482	11265	0.01922	0.169	...?.
DIP2A	21	0.59740	0.04196	0.05395	0.72028	0.16983	14669	0.01923	0.169	.....
LINC01446	7	0.81918	0.04995	0.21978	NA	0.11089	11265	0.01925	0.169	...?.
EPB41L4A-AS2	5	0.88611	0.10689	0.29570	0.07992	0.28871	14669	0.01926	0.169	.....
AKR7L	1	0.24476	0.43856	0.74126	0.14086	0.18082	14669	0.01927	0.169	.....
ARID3A	19	0.25075	0.20280	0.27173	0.73127	0.09790	14669	0.01928	0.169	.....
CNFN	19	0.11489	NA	0.78521	0.15385	0.16883	13691	0.01928	0.169	.?...
RAPSN	11	0.97602	0.08791	0.76324	0.75724	0.02398	14669	0.01929	0.169	.....
LOC100506679	22	0.78022	0.15584	0.02797	0.02997	0.67333	14669	0.01929	0.169	.....
SIGLEC11	19	0.18182	0.10989	0.21179	0.48551	0.23676	14669	0.01929	0.169	.....
TMEM232	5	0.17682	0.84216	0.65035	0.03896	NA	6657	0.01931	0.169	....?
EIF5A2	3	0.16284	0.70130	0.28072	0.38961	0.12587	14669	0.01932	0.169	.....
ORMDL3	17	0.58142	0.53447	0.80220	0.46454	0.03097	14669	0.01932	0.169	.....
UBE2B	5	0.25075	0.31768	0.24276	NA	0.11888	11265	0.01933	0.169	...?.
KCNH4	17	0.46254	0.45455	0.51648	0.01698	0.40859	14669	0.01934	0.169	.....
FAM57A	17	0.16583	1.00000	0.07692	0.74625	0.07293	14669	0.01934	0.169	.0...
UBA3	3	0.80719	0.35465	0.31469	0.36464	0.06893	14669	0.01938	0.169	.....
PCSK6	15	0.79820	0.27073	0.17882	0.10390	0.24076	14669	0.01938	0.169	.....
RFX5	1	0.58042	0.74925	0.54845	0.05894	0.16384	14669	0.01938	0.169	.....
LRRC74B	22	0.04995	0.16783	0.83217	0.72827	0.11888	14669	0.01939	0.169	.....
ZNF681	19	0.12687	0.48851	0.89610	0.72428	0.04496	14669	0.0194	0.169	.....
TPM4	19	0.49850	0.01199	0.13487	0.24176	0.45355	14669	0.01941	0.169	.....
RPL18	19	0.54745	NA	0.13886	0.47353	0.06394	13691	0.01941	0.169	.?...
SAFB	19	0.32767	0.41259	0.49550	0.65634	0.05095	14669	0.01941	0.169	.....
SH2D4A	8	0.46853	0.43956	0.59840	NA	0.04296	11265	0.01943	0.169	...?.
LOC105377763	5	0.83916	0.94605	0.09790	0.46154	0.04196	14669	0.01943	0.169	.....
LYPD6	2	0.28571	0.90809	NA	0.07792	0.15285	13818	0.01944	0.169	..?..
CCL1	17	0.16484	0.44755	0.72627	0.10290	0.25674	14669	0.01944	0.169	.....
TMPRSS11A	4	0.01698	0.23177	0.05994	0.79221	0.34066	14669	0.01945	0.169	.....
HEATR9	17	0.22078	0.46653	0.23477	0.66633	0.08492	14669	0.01946	0.169	.....
PTPN6	12	0.80719	0.50549	0.23077	0.01499	0.39560	14669	0.01947	0.169	.....
TRIM59	3	0.01998	0.69530	0.33267	0.73926	0.13087	14669	0.01948	0.169	.....
SYT9	11	NA	0.90310	0.04595	NA	0.05794	9841	0.01948	0.169	?..?.
TET1	10	0.50649	0.10789	0.26673	0.63237	0.09990	14669	0.01949	0.169	.....
ZNF138	7	0.02997	0.34066	0.29471	NA	0.23776	11265	0.01949	0.169	...?.
CHURC1	14	0.61439	0.86913	0.92408	0.07293	0.09091	14669	0.0195	0.169	.....
STAU2-AS1	8	0.44356	0.57343	0.77722	NA	0.03097	11265	0.01951	0.169	...?.

ACTN1	14	0.72028	0.78222	0.58941	NA	0.01898	11265	0.01952	0.169	...?.
ELOVL2	6	0.18681	0.38262	0.03896	0.81019	0.14186	14669	0.01953	0.169	.....
GYS2	12	0.18282	0.45654	0.82218	NA	0.06094	11265	0.01954	0.169	...?.
HOOK1	1	0.19980	0.10889	0.32468	0.80020	0.11588	14669	0.01957	0.169	.....
ARMC10	7	0.97203	0.43956	0.50450	0.00500	0.40859	14669	0.01958	0.169	.....
GPR107	9	0.74126	0.37363	0.90509	0.32967	0.04196	14669	0.01959	0.169	.....
POP7	7	0.28172	0.07393	0.52847	0.32667	0.21678	14669	0.0196	0.169	.....
SLC2A11	22	0.79620	0.68132	0.07892	0.15784	0.15784	14669	0.0196	0.169	.....
ARHGAP28	18	0.37163	0.28272	0.99800	NA	0.04396	11265	0.01961	0.169	...?.
EHD4	15	0.03297	0.00910	0.68631	0.65834	0.35365	14669	0.01961	0.169	.....
DMBX1	1	0.80420	0.76823	0.34665	0.04895	0.17383	14669	0.01961	0.169	.....
CCDC122	13	0.10390	0.11788	0.20280	NA	0.26274	11265	0.01962	0.169	...?.
POM121L9P	22	0.90609	0.40060	0.69331	0.34166	0.03896	14669	0.01967	0.170	.....
UROD	1	0.54246	0.22478	0.46354	0.65335	0.05195	14669	0.01967	0.170	.....
MICU2	13	0.01299	0.72228	0.05195	0.85614	0.21778	14669	0.0197	0.170	.....
POLR3E	16	0.52647	0.64635	0.73726	0.04296	0.19381	14669	0.0197	0.170	.....
LOC388813	21	0.01299	0.90909	0.34466	0.16583	0.39161	14669	0.01971	0.170	.....
PPARGC1A	4	0.38062	0.33666	0.17383	0.61139	0.09391	14669	0.01972	0.170	.....
CAB39	20	NA	0.83117	0.07093	0.05994	0.26773	13245	0.01973	0.170	?....
TNIK	3	0.10090	0.58042	0.41259	0.00390	0.90310	14669	0.01973	0.170	.....
GPRC5D	12	0.53846	0.30270	0.34565	0.21878	0.15385	14669	0.01974	0.170	.....
WSB2	12	0.50949	0.05794	0.57443	NA	0.10090	11265	0.01975	0.170	...?.
CCDC117	22	0.73526	0.50849	0.84715	0.02797	0.19880	14669	0.01976	0.170	.....
ZBTB16	11	NA	0.34066	0.58142	0.03696	0.26074	13245	0.01976	0.170	?....
RPP14	3	0.08891	NA	0.70030	0.02498	0.45754	13691	0.01977	0.170	.?...
OR51E2	11	0.40559	0.63936	0.55744	0.95305	0.01598	14669	0.01979	0.170	.....
DEFB121	20	0.05295	0.51548	0.56543	0.64935	0.10390	14669	0.01979	0.170	.....
FMO4	1	0.41958	0.71129	0.79520	0.01898	0.28472	14669	0.01979	0.170	.....
LRRC27	10	0.90310	0.62038	0.26973	0.22677	0.07493	14669	0.0198	0.170	.....
VAMP8	2	0.02597	0.26174	0.08691	0.57343	0.36963	14669	0.0198	0.170	.....
EFCAB10	7	0.02198	0.34565	0.19081	0.91309	0.17383	14669	0.01981	0.170	.....
NCBP1	9	0.21778	0.11389	0.85315	0.18382	0.24875	14669	0.01984	0.170	.....
CABP2	11	0.34865	0.87213	0.04795	0.17682	0.23976	14669	0.01984	0.170	.....
LARP1B	4	0.05794	0.10290	0.24975	0.68332	0.25375	14669	0.01985	0.170	.....
IL20RB	3	0.59341	0.84116	0.25974	0.25375	0.08092	14669	0.01985	0.170	.....
C10orf12	10	0.40959	0.46553	0.08891	0.46553	0.12887	14669	0.01986	0.170	.....
SSTR2	17	0.11788	0.83117	0.81618	NA	0.05095	11265	0.01987	0.170	...?.
TEX21P	14	0.65035	0.27273	0.12388	NA	0.09191	11265	0.01988	0.170	...?.
POLR2F	22	0.01499	0.02897	0.08392	0.31369	0.88711	14669	0.01989	0.170	.....
NMBR	6	0.50949	0.16883	0.97403	0.02198	0.37463	14669	0.0199	0.170	.....
ANXA7	10	0.48851	0.38761	0.77922	NA	0.03796	11265	0.01991	0.170	...?.
SLC47A1	17	0.08392	0.17782	0.84715	0.90010	0.07093	14669	0.01991	0.170	.....
MYC	8	0.86513	NA	0.80320	0.22378	0.03596	13691	0.01992	0.170	.?...
DDX11	12	0.20879	0.42657	0.74426	0.60240	0.05794	14669	0.01992	0.170	.....
INHBA-AS1	7	0.53746	0.78322	0.18981	0.12388	0.17283	14669	0.01993	0.170	.....
SH3GLB2	9	0.09491	NA	0.76224	0.17083	0.17782	13691	0.01993	0.170	.?...
HSDL1	16	0.02697	0.19880	0.70729	0.30370	0.32967	14669	0.01995	0.170	.....
LINC00865	10	0.54246	0.19381	0.98901	0.19081	0.11389	14669	0.01996	0.170	.....
SIRT6	19	0.52248	0.72128	0.99600	0.18881	0.05794	14669	0.01997	0.170	.....
NCAPD3	11	0.87912	0.87413	0.63736	0.01199	0.21578	14669	0.01997	0.170	.....
MYLK2	20	0.26673	0.56843	0.97902	0.28472	0.07592	14669	0.01998	0.170	.....
LOC101927948	2	0.29371	0.61638	0.27572	0.22378	0.16683	14669	0.01998	0.170	.....
LONRF1	8	0.30270	0.73726	0.04995	NA	0.11688	11265	0.01999	0.170	...?.
DPRX	19	0.42957	NA	0.15485	NA	0.06593	10287	0.02002	0.170	.?.?.
JUP	17	0.10589	0.24376	0.13087	0.10090	0.61938	14669	0.02002	0.170	.....
NXPH3	17	0.96703	0.06394	0.14885	0.45255	0.12787	14669	0.02002	0.170	.....
GPR161	1	0.02697	0.50150	0.09790	0.42458	0.35764	14669	0.02003	0.170	.....
HOXB5	17	0.10090	0.70330	0.18282	0.23576	0.26474	14669	0.02003	0.170	.....
TMEM246-AS1	9	0.90909	0.33966	0.30669	0.78921	0.02398	14669	0.02004	0.170	.....
MCOLN1	19	0.71928	0.19281	0.87612	0.13487	0.12987	14669	0.02005	0.170	.....
SMR3B	4	0.11389	0.29870	0.48052	0.66833	0.10789	14669	0.02005	0.170	.....
CCDC113	16	0.16184	0.41758	0.13487	0.69530	0.12587	14669	0.02006	0.170	.....
INHBA	7	0.57942	0.79421	0.15684	0.12388	0.17483	14669	0.02007	0.170	.....
SERHL	22	0.08891	NA	0.02597	0.81518	0.14885	13691	0.02009	0.170	.?...
PTN	7	0.64835	0.65035	0.01698	0.01230	0.70030	14669	0.0201	0.170	.....
PIGL	17	0.99201	0.45255	0.08092	0.43956	0.07093	14669	0.0201	0.170	.....
LOC101927164	1	0.18382	0.89710	0.52148	0.09690	0.21179	14669	0.0201	0.170	.....
PLEKHD1	14	0.11389	0.02697	0.25475	NA	0.34466	11265	0.02013	0.170	...?.
LOC284578	1	0.31968	0.05794	0.30370	0.48452	0.20380	14669	0.02016	0.170	.....

SNRPA1	15	0.61039	0.77323	0.33566	0.10390	0.14585	14669	0.02017	0.170	.....
MMAB	12	0.18981	0.64935	0.37562	0.00880	0.66334	14669	0.02018	0.170	.....
DUOXA1	15	0.53746	0.02398	0.35864	0.55345	0.15884	14669	0.02018	0.170	.....
TRIML2	4	0.81918	0.37463	0.29271	0.53247	0.04695	14669	0.02018	0.170	.....
MPL	1	0.04695	0.42458	0.87313	0.30470	0.19181	14669	0.02019	0.170	.....
TEKT4	2	0.41958	0.53746	0.86513	0.10290	0.14186	14669	0.02019	0.170	.....
LOC101927438	2	0.27672	0.15385	0.20679	0.01798	0.81618	14669	0.02019	0.170	.....
YARS	1	0.04595	0.54246	0.35065	0.08192	0.51548	14669	0.0202	0.170	.....
PLPP2	19	NA	0.77622	0.80519	0.19880	0.04895	13245	0.0202	0.170	?....
FAN1	15	0.08891	0.02797	0.80619	0.18482	0.47552	14669	0.0202	0.170	.....
DCTN1	2	0.08991	0.03996	0.63237	0.14486	0.54645	14669	0.02023	0.170	.....
COL6A1	21	0.06294	0.55245	0.38062	0.35564	0.20679	14669	0.02024	0.170	.....
HNRNPL	19	0.17982	0.21179	0.53047	0.01798	0.69431	14669	0.02025	0.170	.....
POU6F1	12	0.15684	0.16484	0.90809	0.21978	0.22178	14669	0.02025	0.170	.....
LOC105376398	10	0.56144	0.61938	0.00810	0.26873	0.25475	14669	0.02026	0.170	.....
ITPR3	6	NA	0.54146	0.75425	NA	0.02398	9841	0.02026	0.170	?..?.
BLOC1S3	19	0.95105	NA	0.23277	0.04196	0.19580	13691	0.02028	0.170	.?...
ASCL5	1	0.55944	NA	0.89111	0.00230	0.44955	13691	0.02029	0.170	.?...
ELSPBP1	19	0.10190	0.47552	0.42857	0.49550	0.13387	14669	0.0203	0.170	.....
KLK5	19	0.00480	0.53846	0.70929	0.69930	0.17283	14669	0.0203	0.170	.....
USP47	11	0.95704	0.19081	0.46553	NA	0.04196	11265	0.02031	0.170	...?.
PRORSD1P	2	0.32068	0.70030	0.10490	0.32168	0.15784	14669	0.02031	0.170	.....
C20orf85	20	0.02897	0.25375	0.28172	0.39760	0.35864	14669	0.02032	0.170	.....
MIR3188	19	0.88711	NA	0.05594	0.67333	0.03796	13691	0.02034	0.170	.?...
EIF2B2	14	0.15684	0.09191	0.80120	0.87512	0.07692	14669	0.02035	0.170	.....
TMEM52B	12	0.79021	0.49351	0.74925	0.01330	0.28472	14669	0.02035	0.170	.....
UNC119B	12	0.94306	0.42557	0.19281	0.30270	0.08292	14669	0.02036	0.170	.....
NT5E	6	0.47253	0.22178	0.78821	0.02198	0.40759	14669	0.02037	0.170	.....
SOS1	2	0.77522	0.30769	0.64436	0.10490	0.14585	14669	0.02037	0.170	.....
SF3B4	1	0.24875	NA	0.42358	0.03097	0.37962	13691	0.02037	0.170	.?...
HIPK4	19	0.06793	0.34765	0.74426	0.04595	0.52647	14669	0.02038	0.170	.....
LOC389247	4	0.39760	NA	0.16883	0.42058	0.08891	13691	0.0204	0.170	.?...
SLC1A1	9	0.19381	0.16284	0.13087	NA	0.22178	11265	0.0204	0.170	...?.
FNIP1	5	0.03397	0.43956	0.01998	0.93007	0.24675	14669	0.0204	0.170	.....
PDP1	8	0.41958	0.60639	0.73926	NA	0.03397	11265	0.02042	0.170	...?.
UMODL1	21	0.00640	0.61339	0.26474	NA	0.28971	11265	0.02046	0.170	...?.
MED24	17	0.19381	0.67932	0.23377	0.12787	0.28771	14669	0.02047	0.170	.....
RANBP1	22	0.42358	0.21379	0.88012	0.38761	0.07992	14669	0.0205	0.170	.....
PARP3	3	0.42458	0.48452	0.25574	0.05395	0.35365	14669	0.02051	0.170	.....
PTBP3	9	0.43956	0.93307	0.54346	0.00100	0.66234	14669	0.02052	0.170	.....
GBA2	9	0.01798	0.69131	0.17782	0.65534	0.20480	14669	0.02054	0.171	.....
CST8	20	0.69830	0.28671	0.35564	0.15185	0.17283	14669	0.02055	0.171	.....
HIST1H2BK	6	0.63237	0.64935	0.09391	0.17283	0.18082	14669	0.02058	0.171	.....
SEPT7P2	7	0.19780	0.00899	0.39161	0.60939	0.28671	14669	0.02058	0.171	.....
SETD6	16	0.03996	0.92008	0.20679	0.91608	0.07992	14669	0.02062	0.171	.....
IRF3	19	0.74725	NA	0.87612	0.21678	0.04196	13691	0.02064	0.171	.?...
LOC100130673	7	0.20679	0.67632	0.32468	NA	0.08092	11265	0.02064	0.171	...?.
LOC101927391	7	0.07193	0.40859	0.46354	NA	0.14486	11265	0.02065	0.171	...?.
LOC729307	4	0.11489	0.46154	0.44456	0.84416	0.06693	14669	0.02066	0.171	.....
EME2	16	0.22278	0.25574	0.89411	0.44156	0.09391	14669	0.02066	0.171	.....
PCYOX1	2	0.21978	0.89411	0.97502	0.01598	0.32368	14669	0.02066	0.171	.....
GRAMD1A	19	0.66733	0.01399	0.01499	0.30969	0.52448	14669	0.02066	0.171	.....
CRISPLD1	8	0.23177	0.30769	0.06893	0.19181	0.40859	14669	0.02069	0.171	.....
LOC100506022	1	0.66633	NA	0.27173	0.01698	0.35365	13691	0.02073	0.171	.?...
HEMK1	3	0.28172	0.95005	0.94905	0.70629	0.01698	14669	0.02074	0.171	.....
EN1	2	0.80519	0.19481	0.57143	0.06294	0.23976	14669	0.02075	0.171	.....
GAS6-AS1	13	0.37562	0.06693	0.56943	NA	0.12288	11265	0.02076	0.171	...?.
ZC3HAV1L	7	0.11089	0.03696	0.05195	0.22677	0.75924	14669	0.02076	0.171	.....
SCGB1D4	11	0.50549	0.38661	0.73626	0.86713	0.01998	14669	0.02076	0.171	.....
LINC01120	2	0.36464	0.27173	0.00950	0.83217	0.16783	14669	0.02076	0.171	.....
NXPE3	3	0.46254	0.43856	0.09091	0.56444	0.10490	14669	0.02077	0.171	.....
LOC101927550	7	0.02198	NA	0.07692	0.08791	0.71329	13691	0.02079	0.171	.?...
TCHP	12	0.27173	0.59441	0.22478	0.30370	0.15784	14669	0.0208	0.171	.....
OGDH	7	0.01199	0.79720	0.17383	NA	0.25075	11265	0.02081	0.171	...?.
MCCC1	3	0.57642	0.42557	0.25574	0.54945	0.06394	14669	0.02083	0.171	.....
CUL1	7	0.90909	0.48951	0.90909	0.41958	0.02298	14669	0.02084	0.171	.....
TP73-AS1	1	0.66434	0.19580	0.48252	0.03297	0.38262	14669	0.02084	0.171	.....
PLBD2	12	0.19580	0.67433	0.25574	0.93906	0.04396	14669	0.02084	0.171	.....
PIK3C2A	11	0.44555	0.86913	0.10090	0.47852	0.07992	14669	0.02084	0.171	.....

C21orf58	21	0.76424	0.10989	0.16983	0.64136	0.09091	14669	0.02085	0.171	.....
MED8	1	0.05495	NA	0.98801	0.27273	0.13586	13691	0.02086	0.171	.?...
USP42	7	0.13387	0.03297	0.33866	0.34066	0.41459	14669	0.02088	0.171	.....
PTPRO	12	0.64336	0.56843	0.53946	NA	0.03197	11265	0.02088	0.171	...?.
SGOL1	3	0.42757	0.29770	0.37862	0.54446	0.08092	14669	0.02089	0.171	.....
TMEM106B	7	0.09590	0.08591	0.12188	0.18981	0.64236	14669	0.02091	0.171	.....
PRKAA2	1	0.72727	0.56044	0.15285	0.00160	0.80120	14669	0.02091	0.171	.....
LOC101927189	6	NA	0.01499	0.43656	NA	NA	1829	0.02091	0.171	?..??
CDKN1B	12	0.12488	0.64336	0.11089	0.05994	0.56843	14669	0.02092	0.171	.....
ATP6V1C1	8	0.83816	0.24675	0.59341	0.50350	0.04296	14669	0.02092	0.171	.....
TRIM68	11	0.03996	0.42857	0.20380	0.77622	0.16683	14669	0.02094	0.171	.....
GSTA7P	6	0.17483	0.97403	0.24975	0.20280	0.18182	14669	0.02096	0.171	.....
MRPL9	1	0.25874	0.89910	0.77922	0.02997	0.27273	14669	0.02098	0.171	.....
OR7E5P	11	0.74126	0.73427	0.03197	0.51249	0.08192	14669	0.02098	0.171	.....
ZNF544	19	0.09191	0.31768	0.47153	0.33167	0.23177	14669	0.021	0.172	.....
ANKRD46	8	0.03197	0.83417	0.35964	0.21079	0.30270	14669	0.02103	0.172	.....
KLHL3	5	0.30869	0.08991	0.39461	NA	0.15085	11265	0.02105	0.172	...?.
SOCS2-AS1	12	0.86713	0.13487	0.46354	NA	0.05694	11265	0.02105	0.172	...?.
TUBB3	16	0.88611	0.24775	0.06793	0.60240	0.08492	14669	0.02106	0.172	.....
DRC1	2	0.19780	0.09690	0.39560	0.10789	0.50050	14669	0.02106	0.172	.....
TES	7	0.19980	0.50849	0.68631	NA	0.06693	11265	0.02107	0.172	...?.
RBFA	18	0.01898	0.71528	0.25275	NA	0.21279	11265	0.02107	0.172	...?.
SLC35F5	2	0.12388	0.24376	0.86014	0.76424	0.06993	14669	0.02107	0.172	.....
CHORDC1	11	0.96903	0.11189	0.48452	0.13287	0.17982	14669	0.0211	0.172	.....
ATP5G2	12	0.32967	0.28971	0.52947	0.07093	0.33866	14669	0.02113	0.172	.....
ENHO	9	0.70030	0.66733	0.14486	0.68132	0.03896	14669	0.02113	0.172	.....
MDH1	2	0.11588	0.79421	0.13487	0.00999	0.84216	14669	0.02114	0.172	.....
TUBA1C	12	0.74525	0.72827	0.10889	0.09790	0.20779	14669	0.02115	0.172	.....
PHTF2	7	0.30470	0.35764	0.13886	0.74426	0.09491	14669	0.02116	0.172	.....
CD8B	2	0.53646	0.79920	0.23377	0.28571	0.09191	14669	0.02117	0.172	.....
C1orf54	1	0.54545	NA	0.02997	0.10490	0.32068	13691	0.0212	0.172	.?...
HTT	4	0.72428	0.89710	0.60739	0.05594	0.13087	14669	0.02121	0.172	.....
SOD2	6	0.25874	0.53746	0.87113	0.12488	0.16983	14669	0.02122	0.172	.....
UBAC2	13	0.18082	0.65135	0.29471	0.10490	0.31868	14669	0.02122	0.172	.....
KBTBD11	8	0.32867	0.90609	0.24975	NA	0.05694	11265	0.02123	0.172	...?.
PPFIA3	19	0.30070	0.15684	0.02098	0.64336	0.25774	14669	0.02125	0.172	.....
ZNF540	19	0.67932	0.01998	0.61239	0.27772	0.20879	14669	0.02126	0.172	.....
LOC102577426	5	0.63736	NA	0.63636	0.20879	0.06593	13691	0.02126	0.172	.?...
BOP1	8	0.96903	0.82118	0.80719	0.20779	0.03397	14669	0.02127	0.172	.....
MSH6	2	0.09690	0.41059	0.78521	0.95704	0.04496	14669	0.02127	0.172	.....
SHC4	15	0.99600	0.01200	0.78122	0.04296	0.40859	14669	0.02128	0.172	.....
PSTPIP2	18	0.97303	0.20579	0.64436	0.04695	0.22478	14669	0.0213	0.172	.....
PLBD1-AS1	12	0.86214	0.84515	0.33766	0.08492	0.12388	14669	0.0213	0.172	.....
CLDN3	7	0.89510	NA	0.29471	0.13586	0.10090	13691	0.02131	0.172	.?...
CES1P1	16	0.06394	NA	0.15884	0.15984	0.39361	13691	0.02132	0.172	.?...
LOC338963	15	0.11189	0.78222	0.06094	0.68432	0.14086	14669	0.02132	0.172	.....
ATXN7L3	17	0.18981	0.38561	0.69530	0.51948	0.08691	14669	0.02132	0.172	.....
USP32P1	17	0.17982	NA	0.36264	0.11189	0.26673	13691	0.02134	0.172	.?...
ACOT7	1	0.30170	0.26573	0.80320	0.21778	0.15984	14669	0.02135	0.172	.....
CSNK2B	6	0.08192	0.22677	0.88511	NA	0.12687	11265	0.02135	0.172	...?.
ERV3-1	7	0.83317	0.11189	0.97303	0.25874	0.08492	14669	0.02136	0.172	.....
LINC00626	1	0.86513	0.04496	0.89111	0.18482	0.15185	14669	0.02136	0.172	.....
CDH18	5	0.21578	0.16284	0.16084	NA	NA	3253	0.02137	0.172	...??
WDR17	4	0.37263	0.76723	0.78022	0.27972	0.06593	14669	0.02138	0.172	.....
CD163L1	12	0.25375	0.26573	0.65035	0.04895	0.41658	14669	0.02139	0.172	.....
ZNF628	19	0.28372	0.88412	0.66733	0.04296	0.24975	14669	0.0214	0.172	.....
C3orf80	3	0.46853	0.18182	0.21578	0.09291	0.39960	14669	0.02141	0.172	.....
WDR19	4	0.82018	0.57742	0.34865	0.44056	0.04396	14669	0.02141	0.172	.....
ZFYVE28	4	0.94705	0.67532	0.57343	0.15684	0.06693	14669	0.02142	0.172	.....
LOC143666	11	0.33666	0.81119	0.16583	0.03097	0.44356	14669	0.02143	0.172	.....
SCAND2P	15	0.18082	0.25175	0.04695	NA	0.26474	11265	0.02144	0.172	...?.
SGCD	5	0.25275	0.03497	0.02797	0.89910	0.27373	14669	0.02144	0.172	.....
NELFA	4	0.28571	0.81319	0.03197	0.40559	0.18082	14669	0.02145	0.172	.....
LOC100131303	16	0.43656	0.29870	0.14985	0.33267	0.18681	14669	0.02146	0.172	.....
NPTX2	7	0.12388	0.90809	0.80619	0.73327	0.03397	14669	0.02147	0.172	.....
RNF150	4	NA	0.95405	0.83017	0.60939	0.01130	13245	0.02148	0.172	?....
TNFAIP8L3	15	0.06494	0.86214	0.53147	0.95804	0.04196	14669	0.0215	0.172	.....
TEX36-AS1	10	0.22777	NA	0.94006	0.84615	0.01898	13691	0.0215	0.172	.?...
GPR137B	1	0.46653	0.49451	0.07592	0.57043	0.10589	14669	0.0215	0.172	.....

IMPA1	8	0.01399	0.35265	0.98601	0.28671	0.29371	14669	0.0215	0.172	.....
PCCA	13	0.00060	0.29371	0.56244	0.97802	0.27572	14669	0.02153	0.172	.....
MFSD7	4	0.03596	NA	0.79920	0.07093	0.38961	13691	0.02154	0.172	.?...
B4GALT4-AS1	3	0.34565	0.04695	0.40360	0.03397	0.71628	14669	0.02156	0.172	.....
LOC105376554	11	0.09690	0.77822	0.65634	NA	0.07493	11265	0.02159	0.172	...?.
KCTD7	7	0.94406	0.32368	0.64036	NA	0.02797	11265	0.02159	0.172	...?.
EVC	4	0.96304	0.87113	0.45455	0.09690	0.08791	14669	0.0216	0.172	.....
AGO4	1	0.99201	0.75325	0.04096	0.06693	0.25674	14669	0.0216	0.172	.....
ADH1B	4	0.40559	0.71129	0.34965	0.66833	0.04096	14669	0.02162	0.172	.....
GGCX	2	0.06394	0.51149	0.00700	0.37063	0.52547	14669	0.02163	0.173	.....
UPP2	2	0.97702	0.07692	0.04496	0.32168	0.23876	14669	0.02164	0.173	.....
RNF183	9	0.45055	0.09590	0.05994	0.10290	0.59540	14669	0.02166	0.173	.....
IMPAD1	8	0.00999	0.26573	0.21778	NA	0.39760	11265	0.02168	0.173	...?.
LINC00570	2	0.62038	NA	0.79520	0.37762	0.03297	13691	0.02169	0.173	.?...
MYCBPAP	17	0.95205	0.04795	0.95704	0.20080	0.12388	14669	0.02169	0.173	.....
OR5H14	3	0.23377	0.04995	0.03696	0.43856	0.46254	14669	0.02169	0.173	.....
LOC101926940	5	0.57343	0.42058	0.08092	0.36064	0.15485	14669	0.0217	0.173	.....
IQGAP1	15	0.48551	0.21778	0.61439	0.59141	0.06194	14669	0.02173	0.173	.....
RERGL	12	0.07293	0.48352	0.10989	0.01598	0.99001	14669	0.02176	0.173	.....
HTRA2	2	0.01798	NA	0.35864	0.02697	0.79421	13691	0.02177	0.173	.?...
DNM2	19	0.22677	0.32667	0.68931	0.07792	0.33067	14669	0.02177	0.173	.....
LOC100130264	20	0.17283	0.72627	0.54545	0.03397	0.40759	14669	0.02178	0.173	.....
CAMK2D	4	0.77822	0.36963	0.04496	0.12288	NA	6657	0.02178	0.173	....?
NDUFA4L2	12	NA	0.43457	NA	0.02897	NA	4382	0.02179	0.173	?.?.?
C2orf66	2	0.78521	0.14086	0.14685	0.30869	0.18082	14669	0.02179	0.173	.....
FBXO41	2	0.02198	0.41459	0.17383	0.61638	0.27872	14669	0.0218	0.173	.....
UGT1A6	20	NA	0.03097	0.28871	0.09091	0.45455	13245	0.0218	0.173	?....
DHX15	4	0.99001	0.03197	0.21778	0.13187	0.33467	14669	0.02183	0.173	.....
TNRC18P1	4	0.40160	NA	0.25175	0.21878	0.14186	13691	0.02184	0.173	.?...
GLP1R	6	0.06693	0.13387	0.45754	NA	0.23776	11265	0.02185	0.173	...?.
SH2D2A	1	0.33267	0.10589	0.40959	0.01898	0.74026	14669	0.02185	0.173	.....
NDUFS6	5	0.33367	0.06993	0.53546	0.55245	0.14086	14669	0.02185	0.173	.....
AKNAD1	1	0.06494	0.24575	0.21578	0.32567	0.38162	14669	0.02185	0.173	.....
ADAM5	8	0.98102	0.22078	0.09690	NA	0.08192	11265	0.02187	0.173	...?.
LPP-AS2	3	0.80320	0.08991	0.30270	0.33766	0.14885	14669	0.02187	0.173	.....
MS4A14	11	0.25075	0.22378	0.80719	0.00600	0.74725	14669	0.02187	0.173	.....
LOC102724532	17	0.23876	0.71329	0.36563	0.05894	0.34166	14669	0.02188	0.173	.....
NKG7	20	NA	NA	0.78022	0.11089	0.08791	12267	0.02188	0.173	??...
KLRB1	12	0.60240	0.97802	0.03796	NA	0.06993	11265	0.0219	0.173	...?.
FGFBP2	4	0.63137	0.68731	0.28771	0.37962	0.06593	14669	0.02192	0.173	.....
KCNT2	1	NA	0.51748	0.12987	0.08492	0.26973	13245	0.02193	0.173	?....
TFDP2	3	0.04296	0.36164	0.53846	0.08192	0.55245	14669	0.02194	0.173	.....
MPHOSPH9	12	0.13886	0.09690	0.50050	NA	0.19580	11265	0.02194	0.173	...?.
C22orf23	22	0.00799	0.13287	0.05594	0.21978	0.97303	14669	0.02196	0.173	.....
SUMO1P1	20	0.49051	0.98801	0.45255	0.59441	0.02697	14669	0.02197	0.173	.....
MTA1	20	NA	NA	0.48252	0.59341	0.02398	12267	0.02198	0.173	??...
MOG	6	0.12188	0.92108	0.39361	NA	0.07992	11265	0.02199	0.173	...?.
WDR12	2	0.43357	0.67033	0.10689	0.13387	0.26374	14669	0.022	0.173	.....
DDX39B	6	0.10490	0.04695	0.51848	0.17083	0.53646	14669	0.022	0.173	.....
AANAT	17	0.57343	NA	0.52747	0.00600	0.44755	13691	0.02201	0.173	.?...
NTN5	19	0.04695	0.22577	0.85415	0.10090	0.47852	14669	0.02202	0.173	.....
FBXW10	17	0.11988	0.36164	0.33167	0.32268	0.24476	14669	0.02202	0.173	.....
PIP	7	0.69630	0.25674	0.84515	0.82118	0.01998	14669	0.02203	0.173	.....
LCTL	15	0.28971	0.93806	0.77722	0.01499	0.34366	14669	0.02203	0.173	.....
ISCA2	14	0.62537	NA	0.63237	0.16883	0.08292	13691	0.02206	0.173	.?...
GLYATL2	11	0.73926	0.35764	0.71728	0.44056	0.04396	14669	0.02209	0.173	.....
IPO11	5	0.60639	0.71728	0.55844	0.47552	0.03596	14669	0.0221	0.173	.....
NOC4L	12	0.57143	0.11189	0.75724	NA	0.06893	11265	0.02211	0.173	...?.
DIRAS3	1	0.11089	NA	0.48951	0.58042	0.08292	13691	0.02211	0.173	.?...
STMND1	6	0.02098	0.27872	0.76523	0.14785	0.48152	14669	0.02213	0.173	.....
SRP68	17	0.31069	0.85614	0.94805	0.04496	0.20180	14669	0.02216	0.173	.....
PDE8B	5	0.70230	0.62637	0.98302	0.09091	0.09790	14669	0.02217	0.173	.....
OR8J1	11	0.97802	0.42258	0.34466	0.84316	0.01798	14669	0.02219	0.174	.....
WDR63	1	0.03596	0.24176	0.45554	0.14885	0.53746	14669	0.02222	0.174	.....
C2CD4C	20	NA	0.37063	0.54246	0.38262	0.06294	13245	0.02222	0.174	?....
LRRC49	15	0.08591	0.06194	0.29770	0.13487	0.68631	14669	0.02222	0.174	.....
DENND4C	9	0.11988	0.96404	0.59640	0.05594	0.31568	14669	0.02225	0.174	.....
LINC01141	1	0.31169	0.80719	0.38162	0.33666	0.09491	14669	0.02225	0.174	.....
FANCE	6	0.96803	0.05694	0.29371	0.28771	0.16883	14669	0.02225	0.174	.....

CCDC88A	2	0.73726	0.42458	0.21978	0.09391	0.23776	14669	0.02227	0.174	.....
B4GALNT2	17	0.27572	0.90410	0.08791	0.85914	0.05495	14669	0.02227	0.174	.....
SLC34A2	4	0.69031	0.26873	0.22478	0.40060	0.11089	14669	0.0223	0.174	.....
FSIP1	15	0.52647	0.36364	0.02897	0.90410	0.08591	14669	0.0223	0.174	.....
NR1I3	1	0.61239	0.92008	0.19081	0.17982	0.12288	14669	0.0223	0.174	.....
SLC22A1	6	0.10589	0.06693	0.98601	0.65834	0.13986	14669	0.0223	0.174	.....
TICAM1	19	0.59141	0.10989	0.97902	0.11788	0.20080	14669	0.02231	0.174	.....
TRIM15	6	0.03896	0.43556	0.76923	0.76124	0.09790	14669	0.02232	0.174	.....
AKAP11	13	0.02797	0.78921	0.08192	NA	0.26174	11265	0.02233	0.174	...?.
RBM24	6	0.18082	0.04695	0.40759	0.32368	0.35764	14669	0.02233	0.174	.....
KRT222	17	0.29870	0.03896	0.54246	0.27373	0.30769	14669	0.02233	0.174	.....
NAA30	14	0.39361	NA	0.06593	0.97303	0.04496	13691	0.02235	0.174	.?...
LOC101928371	2	0.80819	0.15784	0.85914	0.34266	0.06893	14669	0.02235	0.174	.....
ADGRL2	1	0.38162	0.41658	NA	0.12288	0.17982	13818	0.02237	0.174	..?..
DCTD	4	0.54446	0.10789	0.10190	0.26174	NA	6657	0.02238	0.174	....?
CUBN	10	NA	0.60939	0.51548	0.40659	0.04496	13245	0.02238	0.174	?....
UBAP2L	1	0.14086	0.09291	0.70330	0.14386	0.42058	14669	0.02239	0.174	.....
ZKSCAN1	7	0.35664	0.89510	0.73526	0.83516	0.01598	14669	0.0224	0.174	.....
CTDSP2	12	0.06494	0.63836	0.29371	0.05694	0.57243	14669	0.0224	0.174	.....
FKBP10	17	0.62837	0.78821	0.99401	0.02697	0.17782	14669	0.02241	0.174	.....
GP1BA	17	0.87413	0.78521	0.74525	0.24575	0.03896	14669	0.02243	0.174	.....
HOXD4	2	0.05295	0.25874	0.97702	0.51748	0.14785	14669	0.02244	0.174	.....
APOLD1	12	0.87213	0.71229	0.12388	0.02597	0.34765	14669	0.02245	0.174	.....
LOC100996325	5	0.12987	0.31968	0.62238	0.09990	0.38462	14669	0.02245	0.174	.....
FABP1	2	0.23077	0.65235	0.11888	0.37163	0.18182	14669	0.02246	0.174	.....
SYCP2L	6	NA	0.66533	0.05495	0.12787	0.24376	13245	0.02246	0.174	?....
ABHD4	14	0.25275	0.10889	0.50350	0.02697	0.69930	14669	0.02246	0.174	.....
MTERF1	7	0.10090	0.19580	0.02997	0.22777	0.65235	14669	0.0225	0.174	.....
HLA-DMB	6	NA	0.51648	0.20779	0.13287	0.18781	13245	0.0225	0.174	?....
RAG2	11	0.39261	0.01199	0.36663	0.08392	0.66833	14669	0.0225	0.174	.....
CDK2	12	0.20480	NA	0.52348	0.22378	0.14785	13691	0.02251	0.174	.?...
KRT3	12	0.67832	0.62238	0.17582	0.87612	0.02797	14669	0.02251	0.174	.....
MSMO1	4	0.74825	0.75425	0.24775	0.08891	0.17483	14669	0.02252	0.174	.....
WFS1	4	0.79121	0.69930	0.09590	0.07093	0.26274	14669	0.02255	0.174	.....
NDUFAF4P1	15	0.94406	0.61838	0.03896	0.10190	0.25275	14669	0.02255	0.174	.....
HMGCS1	5	0.47552	0.87512	0.87313	0.37263	0.03596	14669	0.02256	0.174	.....
LYRM9	17	0.07992	0.94106	0.09890	0.21778	0.32667	14669	0.02256	0.174	.....
LOC642852	21	0.01030	0.93307	0.28671	NA	0.21279	11265	0.02258	0.174	...?.
TCP1	6	0.22278	0.03596	0.13886	NA	0.32967	11265	0.02259	0.174	...?.
MOB3B	9	0.16583	0.39061	0.21279	0.30869	0.25774	14669	0.02259	0.174	.....
LOC101927666	17	0.13686	0.04795	0.47952	0.13287	0.57742	14669	0.0226	0.174	.....
NCK1-AS1	3	0.37363	0.51748	0.13586	0.94406	0.05095	14669	0.02261	0.174	.....
NCK1	3	0.34565	0.88911	0.15385	0.98002	0.03097	14669	0.02262	0.174	.....
RRP1B	21	0.54745	0.06893	0.23377	NA	0.15385	11265	0.02262	0.174	...?.
LOC101928978	4	0.03497	0.65435	0.22677	0.76124	0.14785	14669	0.02263	0.174	.....
GBE1	3	0.16783	0.02797	0.26873	0.57243	NA	6657	0.02265	0.174	....?
FBXL3	13	0.83017	0.55345	0.00370	0.03497	0.64136	14669	0.02266	0.174	.....
LOC101926966	2	0.61938	0.07992	0.37063	0.05994	0.45654	14669	0.02266	0.174	.....
PGAP1	2	0.69031	0.24376	0.03497	0.20280	0.32168	14669	0.02267	0.174	.....
PPP3CC	8	0.01499	0.38561	0.32168	0.36863	0.39760	14669	0.02267	0.174	.....
LOC100506122	4	0.70430	0.39960	0.48651	0.43057	0.05894	14669	0.02268	0.174	.....
PERP	6	0.80519	0.65435	0.46054	0.39161	0.04296	14669	0.02268	0.174	.....
TFR2	7	0.13986	0.55744	0.58442	0.48851	0.10390	14669	0.02268	0.174	.....
GPR88	1	0.74126	0.03497	0.79021	0.37862	0.12188	14669	0.02268	0.174	.....
ZNF563	19	0.85215	0.06593	0.28172	0.27672	0.19281	14669	0.02269	0.174	.....
AKNA	9	0.15385	0.63137	0.79321	0.57942	0.06194	14669	0.02273	0.174	.....
LINC00376	13	0.21079	0.85415	0.45055	0.05495	0.32068	14669	0.02275	0.174	.....
GTF3C5	9	0.23576	0.00600	0.95504	0.20280	0.43956	14669	0.02277	0.174	.....
KDSR	18	0.19281	0.88112	0.37962	NA	0.06993	11265	0.02278	0.174	...?.
TMED2	12	0.78222	0.05495	0.09990	0.25375	0.31069	14669	0.02279	0.174	.....
APOL4	22	0.09890	0.43257	0.07493	0.23277	0.45155	14669	0.02279	0.174	.....
PSME4	2	0.53946	0.42258	0.81119	0.35964	0.06194	14669	0.02281	0.174	.....
PSMD14	2	0.16583	0.47453	0.16284	0.62138	0.14186	14669	0.02284	0.174	.....
CFHR1	1	0.53746	NA	0.09091	0.91808	0.03596	13691	0.02284	0.174	.?...
FZD10-AS1	12	0.19081	0.87512	0.04795	NA	0.14785	11265	0.02288	0.174	...?.
LINC01207	4	0.36464	0.38961	0.47353	NA	0.07493	11265	0.02292	0.174	...?.
HOXB-AS1	17	0.02498	0.42857	0.44156	0.11588	0.56144	14669	0.02292	0.174	.....
IGFL1	19	0.17483	0.64635	0.10490	0.84016	0.09191	14669	0.02293	0.174	.....
LOC101929595	4	0.32168	0.63237	0.86713	0.03297	0.29471	14669	0.02295	0.174	.....

LYRM4	6	0.32368	0.63437	0.47453	NA	0.05994	11265	0.02296	0.174	...?.
OR7E156P	13	0.00140	0.56344	0.57842	0.04196	0.98302	14669	0.02297	0.174	.....
CRY1	12	0.32767	0.93007	0.65135	0.25574	0.08092	14669	0.02297	0.174	.....
LINC01608	8	0.20180	0.36064	0.20080	0.19980	0.33367	14669	0.02297	0.174	.....
MRPS14	1	0.01399	0.05794	0.33267	0.17582	0.85714	14669	0.02298	0.174	.....
LOC102723895	11	0.09291	0.59041	0.46354	0.09291	0.39760	14669	0.02299	0.174	.....
MYH1	17	0.19980	0.28472	0.05794	0.68531	0.20080	14669	0.02302	0.174	.....
PCLO	7	0.28272	0.74825	0.45854	NA	0.05894	11265	0.02303	0.174	...?.
LOC100505853	18	0.16683	0.31568	0.56144	NA	0.11588	11265	0.02304	0.174	...?.
ZKSCAN2	16	0.04496	0.87612	0.06294	0.47053	0.27173	14669	0.02304	0.174	.....
C5orf58	5	0.46454	0.26873	0.18382	0.01798	0.68531	14669	0.02304	0.174	.....
IL1A	2	0.62238	0.44755	0.25874	0.11289	0.22977	14669	0.02304	0.174	.....
SFRP1	8	0.00999	0.76424	0.07093	0.06593	0.92308	14669	0.02307	0.174	.....
CCSAP	1	0.17083	0.23676	0.22178	0.29970	0.31269	14669	0.02307	0.174	.....
H1FX	3	0.06394	0.89211	0.50150	0.02697	0.55844	14669	0.02308	0.174	.....
ATPIF1	1	0.84615	NA	0.33467	0.00700	0.41459	13691	0.02309	0.174	.?...
MFSD14C	9	0.32767	0.49950	0.62537	0.06593	0.28571	14669	0.0231	0.174	.....
DKFZp686K1684	11	0.27672	0.20280	0.97702	NA	0.07493	11265	0.0231	0.174	...?.
APOA1-AS	11	0.96703	0.69431	0.05495	0.39560	0.08192	14669	0.02311	0.174	.....
NCKAP1	2	0.16184	0.89011	0.18981	0.17982	0.25674	14669	0.02311	0.174	.....
HINT1	5	0.09690	0.49750	0.48951	0.45654	0.15385	14669	0.02312	0.174	.....
OC90	8	0.80619	0.10789	0.31169	0.22677	0.19680	14669	0.02312	0.174	.....
BBS7	4	0.23277	0.63836	0.91708	0.01199	0.46254	14669	0.02312	0.174	.....
SLC25A24	1	0.82817	0.97702	0.17782	0.34665	0.05495	14669	0.02312	0.174	.....
SIRT5	6	0.51449	0.44456	0.07992	0.54745	0.11688	14669	0.02315	0.174	.....
THBS1	15	0.46653	0.52547	0.02398	0.99700	0.07093	14669	0.02315	0.174	.....
SHANK1	19	0.03497	0.34166	0.25175	0.61838	0.24875	14669	0.02315	0.174	.....
ADAM19	5	0.30070	0.78821	0.01898	0.14885	0.40260	14669	0.02316	0.174	.....
LOC102724467	16	0.14486	0.52448	0.81918	NA	0.07493	11265	0.02317	0.174	...?.
EPHA1	7	0.63836	0.48252	0.40559	NA	0.04795	11265	0.02317	0.174	...?.
C20orf194	20	0.35365	0.55544	0.37962	0.07093	0.31169	14669	0.02318	0.174	.....
GTF2IP12	4	0.10390	0.66434	0.33966	0.07792	0.43856	14669	0.02318	0.174	.....
DUS1L	17	0.76324	0.24975	0.46853	0.20080	0.14086	14669	0.0232	0.174	.....
LOC101928797	9	0.14785	0.24176	0.01998	0.65435	NA	6657	0.0232	0.174	....?
LRRN2	1	0.18082	0.23976	0.15185	0.12288	0.53247	14669	0.0232	0.174	.....
WDR91	7	0.29970	0.01399	0.41359	NA	0.25874	11265	0.02321	0.174	...?.
CCDC36	3	0.42857	0.70030	0.72028	0.16683	0.11289	14669	0.02321	0.174	.....
CCDC141	2	0.35165	0.62837	0.49650	0.04296	0.33267	14669	0.02321	0.174	.....
PDCD10	3	0.64236	0.45455	0.25175	0.01698	0.49351	14669	0.02322	0.174	.....
APOA1	11	0.90010	0.58641	0.05495	0.48851	0.07992	14669	0.02322	0.174	.....
TUFT1	1	0.57942	0.04196	0.76324	0.15085	0.27473	14669	0.02323	0.174	.....
LINC00320	21	0.68831	0.60240	0.37463	0.60939	0.03596	14669	0.02324	0.174	.....
LINC00936	12	0.31568	0.01998	0.83916	0.59540	0.15485	14669	0.02324	0.174	.....
GALNT13	2	NA	0.96703	0.02997	0.08791	NA	5233	0.02324	0.174	?...?
LOC101927934	5	0.39760	0.11289	0.58641	NA	0.10889	11265	0.02324	0.174	...?.
PIP5K1B	9	0.45754	0.41459	0.27872	NA	0.08192	11265	0.02325	0.174	...?.
LGALS4	19	0.29471	0.25674	0.75724	0.18382	0.20579	14669	0.02326	0.174	.....
LOC339862	3	0.87612	0.83217	0.83217	0.22777	0.03796	14669	0.02327	0.174	.....
LOC157273	8	0.22378	0.00142	0.08092	NA	0.62138	11265	0.02328	0.174	...?.
JMY	5	0.09391	0.75225	0.93706	0.03696	0.38561	14669	0.02329	0.174	.....
LOC646268	1	0.60240	0.83317	0.73926	0.48452	0.02697	14669	0.02331	0.174	.....
RTN2	19	0.05395	0.52847	0.13087	0.69730	0.19680	14669	0.02331	0.174	.....
KANK2	19	0.95704	0.02198	0.79620	0.39560	0.10789	14669	0.02334	0.174	.....
CHRNE	17	0.58142	0.57143	0.63237	0.16484	0.11489	14669	0.02336	0.174	.....
ZNF497	19	0.97602	0.95904	0.40160	0.09191	0.09590	14669	0.02338	0.174	.....
GAS6	13	0.50649	0.03097	0.15485	NA	0.23377	11265	0.02339	0.174	...?.
NEGR1	1	0.25574	0.75225	0.81918	NA	0.04296	11265	0.02339	0.174	...?.
ATXN8OS	13	0.33167	0.53347	0.98302	NA	0.03996	11265	0.0234	0.174	...?.
MLH3	14	0.38262	0.04995	0.97103	0.92807	0.05195	14669	0.0234	0.174	.....
DHRS4-AS1	14	0.02098	NA	0.18182	0.20480	0.46653	13691	0.02341	0.174	.?...
TXLNB	6	0.00023	0.95704	0.63437	NA	0.33666	11265	0.02342	0.174	...?.
EPN3	17	0.08392	0.68831	0.66134	0.19680	0.22478	14669	0.02342	0.174	.....
STX6	1	0.98601	0.24176	0.75724	0.00140	0.64036	14669	0.02343	0.174	.....
CCDC47	17	0.91808	0.75125	0.09990	0.27872	0.09491	14669	0.02344	0.174	.....
LYPLA2P2	19	0.13187	0.06494	0.53946	0.21978	0.43157	14669	0.02344	0.174	.....
FAM225B	9	0.11089	0.36863	0.81918	0.05495	0.45055	14669	0.02345	0.174	.....
SLC8A1-AS1	2	0.07493	0.51049	0.24875	0.06693	0.60639	14669	0.02345	0.174	.....
C5orf28	5	0.67732	0.27572	0.18182	0.09890	0.31768	14669	0.02345	0.174	.....
GID4	17	0.39660	0.47153	0.29471	0.01998	0.54945	14669	0.02347	0.174	.....

FBXW7	4	0.11688	0.47253	0.26773	0.05195	0.59940	14669	0.02349	0.174	.....
LRIT2	10	0.99700	0.78022	0.93107	0.00710	0.23676	14669	0.02349	0.174	.....
C4orf32	4	0.50250	0.69930	0.22378	0.22577	0.14386	14669	0.02349	0.174	.....
RAB11FIP1	8	0.59540	0.32368	0.54346	NA	0.05495	11265	0.0235	0.174	...?.
SDCCAG3	9	0.73027	0.04695	0.30869	NA	0.12987	11265	0.02351	0.174	...?.
C21orf2	21	0.32068	0.28971	0.08891	0.10689	0.50250	14669	0.02353	0.174	.....
MEX3D	19	0.61938	NA	0.53546	0.63736	0.02498	13691	0.02353	0.174	.?...
PPP1R11	6	0.83716	0.64735	0.10889	0.22877	0.13087	14669	0.02354	0.174	.....
LRRC4B	19	0.10190	0.01798	0.89011	0.34466	0.37562	14669	0.02358	0.175	.....
ACSL6	5	0.03896	0.22877	0.02498	0.61139	0.47752	14669	0.02359	0.175	.....
CYFIP1	15	0.18182	0.33866	0.20380	0.58142	0.16384	14669	0.02359	0.175	.....
ABHD18	4	0.12388	0.10689	0.55045	0.53746	0.20979	14669	0.02361	0.175	.....
SCRN2	17	0.09391	0.59540	0.91708	0.06194	0.35764	14669	0.02362	0.175	.....
RASAL2-AS1	1	0.02298	0.15185	0.60839	0.18182	0.57143	14669	0.02362	0.175	.....
LOC100130691	2	0.30270	0.15784	0.74426	0.01099	0.71129	14669	0.02364	0.175	.....
TFPI	2	0.92707	0.01698	0.53147	0.04396	0.50150	14669	0.02365	0.175	.....
KRTCAP3	2	0.14985	0.29471	0.41359	0.12687	0.40859	14669	0.02368	0.175	.....
GALNT12	9	0.16484	0.76324	0.16084	0.08392	0.43157	14669	0.02369	0.175	.....
CLEC7A	12	0.54146	0.40959	0.66034	0.09890	0.19481	14669	0.02369	0.175	.....
RC3H1	1	0.08791	0.26773	0.61638	0.01499	0.83816	14669	0.02374	0.175	.....
DDX39A	19	0.34066	0.83117	0.07093	0.07892	0.39560	14669	0.02375	0.175	.....
LINC01583	15	0.90310	0.11788	0.63037	0.28172	0.10889	14669	0.02376	0.175	.....
CCDC112	5	0.63736	0.86014	0.77423	NA	0.01998	11265	0.02379	0.175	...?.
WNT2	7	0.08591	0.41758	0.97602	0.01798	0.61738	14669	0.02379	0.175	.....
LINC01246	2	0.39660	0.23576	0.75325	0.17882	0.19081	14669	0.0238	0.175	.....
ZNF571-AS1	19	0.73926	0.01199	0.67732	0.23576	0.25774	14669	0.02381	0.175	.....
KLHL20	1	0.35265	0.35664	0.60340	0.07493	0.31069	14669	0.02383	0.175	.....
MIER2	19	0.18881	0.40460	0.92108	0.08292	0.29371	14669	0.02383	0.175	.....
CSPG4	15	0.86414	0.04096	0.75924	NA	0.07293	11265	0.02384	0.175	...?.
EPHA1-AS1	7	0.52747	0.43556	0.38861	NA	0.06294	11265	0.02384	0.175	...?.
KIFC1	6	0.71928	0.53846	0.42058	0.15385	0.13387	14669	0.02384	0.175	.....
HAUS1	18	0.21778	0.29171	0.88811	NA	0.08092	11265	0.02385	0.175	...?.
CCDC127	5	0.90709	0.31968	0.12388	NA	0.07493	11265	0.02385	0.175	...?.
CCDC102A	16	0.29770	0.34066	0.41059	0.05594	0.44056	14669	0.02385	0.175	.....
RBM17	10	0.63437	0.27872	0.15185	0.01400	0.68232	14669	0.02387	0.175	.....
UBXN4	2	0.00999	0.33966	0.86713	0.91209	0.13087	14669	0.02387	0.175	.....
PILRA	7	0.38561	0.92208	0.03696	0.12587	0.32767	14669	0.0239	0.175	.....
LINC01310	22	0.85215	0.18581	0.08392	0.45854	0.14186	14669	0.0239	0.175	.....
PTPRC	1	0.24376	0.08092	0.20579	0.24276	0.43457	14669	0.0239	0.175	.....
IDH2	15	0.62837	0.73826	0.30669	NA	0.04296	11265	0.02391	0.175	...?.
C8orf4	8	0.20180	0.71628	0.56743	0.15085	0.20480	14669	0.02392	0.175	.....
MECR	1	0.14286	0.66833	0.68032	0.33866	0.12587	14669	0.02392	0.175	.....
FADS3	11	0.15485	0.97303	0.57343	0.47353	0.07293	14669	0.02393	0.175	.....
FAM227B	15	0.22677	0.23377	0.18382	0.10889	0.51449	14669	0.02393	0.175	.....
ITGB4	17	0.56144	0.30170	0.36064	0.08192	0.30869	14669	0.02394	0.175	.....
SIGLEC17P	19	0.05594	NA	0.55744	0.08891	0.39760	13691	0.02394	0.175	.?...
RIMS4	20	0.20180	0.48452	0.26174	0.31469	0.21079	14669	0.02396	0.175	.....
PLEKHM1	17	0.47652	0.30669	0.99101	0.74725	0.02897	14669	0.02397	0.175	.....
KIF5C	2	0.58841	0.22378	0.17982	0.43057	0.14685	14669	0.02397	0.175	.....
ASH1L-AS1	20	NA	NA	NA	0.02398	NA	3404	0.02398	0.175	???.?
TPD52L3	9	0.84615	0.84915	0.42557	0.14486	0.08991	14669	0.024	0.175	.....
CYSTM1	5	0.13986	0.04995	0.59041	0.50250	0.25674	14669	0.02401	0.175	.....
TRIM31-AS1	6	NA	0.33167	0.96903	0.42857	0.04196	13245	0.02402	0.175	?....
B3GNTL1	17	0.93706	0.08991	0.02897	NA	0.18082	11265	0.02403	0.175	...?.
CFAP206	6	0.01798	0.38861	0.05894	NA	0.44655	11265	0.02405	0.175	...?.
TMEM246	9	0.46953	0.17582	0.58641	0.23477	0.18581	14669	0.02407	0.176	.....
MUC5B	11	0.84615	0.67333	0.51449	0.88711	0.01180	14669	0.02409	0.176	.....
LPAR3	1	0.47852	0.65435	0.66134	0.16983	0.11888	14669	0.02409	0.176	.....
TAF15	17	0.21479	0.39860	0.25175	0.65834	0.11588	14669	0.0241	0.176	.....
LOC101928303	1	0.08392	0.13686	0.43057	0.15185	0.54046	14669	0.02412	0.176	.....
FAM91A1	8	0.69630	0.79421	0.24076	0.08192	0.20080	14669	0.02413	0.176	.....
SCGB1A1	11	0.06194	0.23576	0.90110	0.93606	0.07892	14669	0.02414	0.176	.....
LACTBL1	1	0.41958	0.13586	0.17882	0.04695	0.64835	14669	0.02417	0.176	.....
REL	2	0.47752	0.82517	0.55644	0.24076	0.08591	14669	0.02418	0.176	.....
DCAF13	8	0.40659	0.63237	0.82018	0.21479	0.09890	14669	0.0242	0.176	.....
ARHGEF12	11	0.23377	0.76723	0.97702	0.74925	0.02597	14669	0.0242	0.176	.....
ZNF727	7	0.34865	0.71329	0.06294	NA	0.12288	11265	0.02421	0.176	...?.
KCTD19	16	0.39760	0.13287	0.76623	0.36164	0.13986	14669	0.02421	0.176	.....
TAS2R3	7	0.12587	0.45954	0.64136	0.47353	0.12687	14669	0.02423	0.176	.....

FSCN3	7	0.02098	0.12088	0.48551	NA	0.35265	11265	0.02424	0.176	...?.
HID1-AS1	17	0.36563	NA	0.22977	0.19580	0.18382	13691	0.02426	0.176	.?...
LOC101927431	2	0.09491	0.43257	0.64935	0.02498	0.64535	14669	0.02429	0.176	.....
CFAP52	17	0.06294	0.00110	0.60440	NA	0.56344	11265	0.02429	0.176	...?.
PLA2G1B	12	0.05495	0.70829	0.00260	NA	0.45355	11265	0.02429	0.176	...?.
LINC01118	2	0.04396	0.91608	0.38761	0.46054	0.16184	14669	0.0243	0.176	.....
MUC17	7	0.56244	0.80320	0.71828	0.05095	0.18482	14669	0.02432	0.176	.....
MANSC1	12	0.07093	0.12687	0.92408	0.27772	0.31069	14669	0.02433	0.176	.....
CDHR5	11	0.85315	0.58841	0.39461	0.07493	0.17982	14669	0.02435	0.176	.....
AZGP1	7	0.30969	0.97502	0.42058	0.18082	0.14086	14669	0.02435	0.176	.....
USP30	12	0.66434	0.64835	0.54246	0.38162	0.05295	14669	0.02436	0.176	.....
UBXN11	1	0.07093	0.20779	0.60140	0.24476	0.35964	14669	0.02438	0.176	.....
TRIM22	11	0.51948	0.20480	0.01898	0.06494	0.71628	14669	0.0244	0.176	.....
C5orf66-AS2	5	0.05894	0.17383	0.36064	0.75624	0.20280	14669	0.02441	0.176	.....
ATP6V1G2-DDX3	6	0.21179	0.24276	0.74126	0.79321	0.06693	14669	0.02442	0.176	.....
GEN1	2	0.74326	0.22478	0.90410	0.07493	0.19980	14669	0.02445	0.176	.....
APLF	2	0.11788	0.11489	0.90110	0.55145	0.15984	14669	0.02445	0.176	.....
LOC101928306	4	0.03397	0.22677	0.68831	0.58442	0.21479	14669	0.02447	0.177	.....
RGS5	1	0.37163	0.05594	0.27473	0.56444	0.20480	14669	0.02447	0.177	.....
PRDM11	11	0.06893	0.74126	0.52847	0.09491	0.38561	14669	0.02448	0.177	.....
GXYLT2	3	0.90110	0.39860	0.35764	0.01698	0.40060	14669	0.02449	0.177	.....
CEP97	3	0.87512	0.30470	0.10989	0.70729	0.06394	14669	0.02451	0.177	.....
SLC25A31	4	0.07293	0.23876	0.58841	0.64236	0.16284	14669	0.02455	0.177	.....
KBTBD11-OT1	8	0.04096	0.89710	0.10789	NA	0.21678	11265	0.02455	0.177	...?.
VWA3A	16	0.21479	NA	0.32667	0.12188	0.27672	13691	0.02457	0.177	.?...
XCR1	3	0.94805	0.45554	0.08991	0.28971	0.13087	14669	0.02458	0.177	.....
SLC2A5	1	0.08591	0.02997	0.89011	0.24076	0.45355	14669	0.02459	0.177	.....
TNR	1	NA	0.95904	0.16783	0.13886	0.14486	13245	0.02459	0.177	?....
API5	11	0.03896	0.06993	0.67033	NA	0.30669	11265	0.02463	0.177	...?.
ZFPL1	11	0.55145	0.10689	0.11089	0.55944	0.18482	14669	0.02464	0.177	.....
CCDC148-AS1	2	0.89510	0.06893	0.62737	0.13487	0.22378	14669	0.02464	0.177	.....
SLC5A5	19	0.21978	0.19980	0.55245	0.88511	0.07393	14669	0.02465	0.177	.....
RIMKLA	1	0.02797	0.29770	0.77622	0.76823	0.14286	14669	0.02465	0.177	.....
QSER1	11	0.41359	0.31568	0.66933	0.04895	0.35664	14669	0.02468	0.177	.....
PLEKHG6	12	0.47053	NA	0.73926	NA	0.03297	10287	0.02469	0.177	.?.?.
MED15P9	2	0.90210	NA	0.04296	0.11089	0.23576	13691	0.02469	0.177	.?...
SURF4	9	0.22178	0.16683	0.22777	0.52947	0.21578	14669	0.02471	0.177	.....
FCRL3	1	0.09790	0.59241	0.20879	0.49750	0.19281	14669	0.02471	0.177	.....
PDPR	16	0.21778	NA	0.05594	NA	0.16084	10287	0.02471	0.177	.?.?.
TAS2R16	7	0.34366	0.44156	0.46354	0.40959	0.11089	14669	0.02472	0.177	.....
SBF2	11	NA	0.04296	0.26174	NA	NA	1829	0.02473	0.177	?..??
HSPBAP1	3	0.61538	0.96304	0.26374	0.16384	0.12388	14669	0.02473	0.177	.....
RBM48	7	0.54645	0.89610	0.91608	0.12388	0.08891	14669	0.02474	0.177	.....
RPS15A	16	0.73926	0.29471	0.83916	0.00240	0.62637	14669	0.02475	0.177	.....
LOC101927795	2	0.62438	0.44156	0.19181	0.47353	0.09491	14669	0.02476	0.177	.....
ARHGAP11B	15	0.17383	NA	0.23976	0.31469	0.18581	13691	0.02476	0.177	.?...
PWAR1	15	0.51249	NA	0.69431	0.11089	0.13586	13691	0.02477	0.177	.?...
RPARP-AS1	10	0.69131	0.13986	0.32967	0.13187	0.29171	14669	0.02478	0.177	.....
KIF23	15	0.46953	0.46354	0.10090	0.36963	0.17483	14669	0.02479	0.177	.....
PPP6R1	19	0.49650	0.57043	0.09590	0.43357	0.13387	14669	0.0248	0.177	.....
TEKT1	17	0.40060	0.78921	0.34166	NA	0.05694	11265	0.0248	0.177	...?.
ALOX15	17	0.31668	0.75824	0.95305	0.11788	0.14186	14669	0.0248	0.177	.....
LOC101927434	1	0.17183	0.31169	0.24076	0.42358	0.22877	14669	0.0248	0.177	.....
PLBD1	12	0.24076	0.21279	0.37762	NA	0.14885	11265	0.02482	0.177	...?.
CCKAR	4	0.65534	0.64036	0.33966	0.05395	0.26374	14669	0.02483	0.177	.....
HEATR5A	14	0.06793	0.21479	0.36464	0.15984	0.53447	14669	0.02486	0.177	.....
SELE	1	0.49051	0.14086	0.54446	0.32867	0.16284	14669	0.0249	0.177	.....
PREPL	2	0.28272	0.27572	0.85714	0.24176	0.16783	14669	0.02492	0.177	.....
RAPH1	2	0.91009	0.44555	0.13886	0.23576	0.14286	14669	0.02492	0.177	.....
SULT1C2P1	2	0.01399	0.33966	0.67433	0.03796	0.86114	14669	0.02493	0.177	.....
SEC13	3	0.59141	0.64535	0.07193	0.19680	0.22278	14669	0.02494	0.177	.....
ANKRD33	12	0.23676	0.65235	0.53347	0.04096	0.39960	14669	0.02496	0.177	.....
DBNDD2	20	0.00270	0.67632	0.15984	0.53746	0.44555	14669	0.02497	0.177	.....
CYTH2	19	0.31269	0.63736	0.95904	0.07493	0.20579	14669	0.02499	0.178	.....
CSF2	5	0.13686	0.01299	0.05095	0.77822	0.47253	14669	0.02499	0.178	.....
ZC3H15	2	0.42358	0.17083	0.52248	0.02897	0.55844	14669	0.025	0.178	.....
LOC100129917	4	0.41658	0.41758	0.91908	0.33067	0.08192	14669	0.02501	0.178	.....
MARVELD3	16	0.30170	0.56144	0.16683	0.17582	0.28871	14669	0.02503	0.178	.....
BCAS4	20	0.75924	0.45654	0.62737	0.06094	0.20679	14669	0.02507	0.178	.....

IMMT	2	0.04695	0.25375	0.59041	0.48851	0.24276	14669	0.02507	0.178	.....
DERL2	17	0.90709	0.53946	0.06194	0.32567	0.12887	14669	0.02508	0.178	.....
FMO3	1	0.01099	0.72028	0.35964	0.58641	0.23776	14669	0.02509	0.178	.....
TMPO-AS1	12	0.37363	0.21578	0.07592	0.71828	0.15884	14669	0.02513	0.178	.....
PBX3	9	0.93706	0.83816	0.26074	NA	0.02897	11265	0.02515	0.178	...?.
APOBEC1	12	0.28472	0.60140	0.17682	0.17183	0.28671	14669	0.02515	0.178	.....
SASH1	6	0.49950	0.96603	0.79421	0.25075	0.05794	14669	0.02517	0.178	.....
LOC100130298	8	0.58442	0.84915	0.16284	0.03097	0.38362	14669	0.02517	0.178	.....
POLR1C	6	0.26573	0.33267	0.15984	0.28272	0.28771	14669	0.02518	0.178	.....
SMIM2	13	0.30070	0.22278	0.23576	0.64535	0.13886	14669	0.0252	0.178	.....
RND3	2	0.12188	0.57343	0.31369	0.69830	0.10989	14669	0.02521	0.178	.....
MEP1B	18	0.66633	0.50150	0.62537	0.02797	0.30769	14669	0.02522	0.178	.....
PKNOX1	21	0.06294	0.22577	0.91508	0.16983	0.37263	14669	0.02522	0.178	.....
MED18	1	0.41259	0.06793	0.20180	0.28472	0.35165	14669	0.02524	0.178	.....
C2orf70	2	0.11888	0.36264	0.04895	0.66633	0.25874	14669	0.02525	0.178	.....
TBC1D29	17	0.86913	0.31269	0.83916	0.00280	0.55445	14669	0.02525	0.178	.....
HLA-DOB	6	0.09690	0.95305	0.02797	NA	0.22378	11265	0.02525	0.178	...?.
SAMD11	1	0.60040	0.60839	0.76923	NA	0.03097	11265	0.02526	0.178	...?.
KIAA1715	2	0.57243	0.84615	0.13686	0.20979	0.15584	14669	0.02527	0.178	.....
TSPAN2	1	0.28172	0.07393	0.53946	0.08192	0.53846	14669	0.02527	0.178	.....
TTPA	8	0.66733	0.84915	0.42458	NA	0.03197	11265	0.02528	0.178	...?.
OR51I1	11	0.13387	0.79620	0.46254	0.45754	0.11389	14669	0.02529	0.178	.....
C2orf47	2	0.23277	0.35165	0.00899	0.23976	0.56144	14669	0.02529	0.178	.....
TMEM130	7	0.03796	0.94006	0.18482	0.24975	0.34565	14669	0.0253	0.178	.....
SFXN1	5	0.15185	0.90909	0.14785	0.22777	0.26074	14669	0.02531	0.178	.....
ATXN2	12	0.03297	0.44655	0.86414	0.50050	0.17183	14669	0.02532	0.178	.....
KLF6	10	0.20380	0.36763	0.26773	NA	0.14985	11265	0.02533	0.178	...?.
FAM131A	3	0.27373	0.85514	0.69630	0.21179	0.11888	14669	0.02534	0.178	.....
LINC00700	10	0.85514	0.32368	0.61239	0.02597	NA	6657	0.02535	0.178	....?
GOLGA6L17P	15	0.07293	0.27972	0.08192	0.72128	0.27473	14669	0.02536	0.178	.....
MEOX2-AS1	7	0.34565	0.96304	0.01499	0.51648	0.16284	14669	0.02537	0.178	.....
ZNF76	6	0.06893	0.08492	0.21578	0.72827	0.30470	14669	0.02538	0.178	.....
PPFIBP1	12	0.05594	0.08192	0.01698	0.52647	0.67433	14669	0.02539	0.178	.....
PPP1R12A	12	0.35764	0.46054	0.21778	NA	0.10889	11265	0.0254	0.178	...?.
CST9L	20	0.55544	0.02298	0.42857	0.16883	0.40859	14669	0.02541	0.178	.....
HIST1H2AH	6	0.79021	0.49850	0.39461	NA	0.04296	11265	0.02541	0.178	...?.
GSTA5	6	0.27473	0.15584	0.01898	0.54446	0.36364	14669	0.02541	0.178	.....
KCNJ3	2	0.06593	0.71728	0.53546	0.51049	0.13586	14669	0.02542	0.178	.....
BLOC1S1	12	0.02997	0.61439	0.17483	0.21279	0.49750	14669	0.02543	0.178	.....
COL6A4P1	3	0.03097	0.53247	0.26174	0.42657	0.31169	14669	0.02546	0.178	.....
SCUBE3	6	0.09091	0.06194	0.18182	0.72228	0.32168	14669	0.02549	0.178	.....
ABCC9	12	0.68232	0.98601	0.25275	NA	0.03596	11265	0.0255	0.178	...?.
ZNF486	19	0.43856	0.35265	0.70629	0.68831	0.04695	14669	0.02551	0.178	.....
LOC105370333	13	0.00210	NA	0.64735	0.97602	0.14585	13691	0.02551	0.178	.?...
OR2A14	7	0.24975	NA	0.00899	0.93107	0.12687	13691	0.02552	0.178	.?...
ALDH3B2	11	0.60240	0.05495	0.10789	0.66933	0.18382	14669	0.02554	0.178	.....
CFAP45	1	0.20579	0.12188	0.13487	0.89710	0.16883	14669	0.02555	0.178	.....
OR52E6	11	0.79221	0.91908	0.09191	0.81219	0.02997	14669	0.02556	0.178	.....
HDAC7	12	0.89111	0.42358	0.04995	0.12288	0.29870	14669	0.02557	0.178	.....
CELA3A	1	0.96903	0.32068	0.38661	0.24476	0.10589	14669	0.02559	0.178	.....
PLPPR5	1	0.10689	0.50949	0.70230	NA	0.10689	11265	0.02559	0.178	...?.
OR51B5	11	0.53546	0.29570	0.66933	NA	0.05994	11265	0.02559	0.178	...?.
RAP2A	13	0.42657	0.85714	0.05994	NA	0.10190	11265	0.0256	0.178	...?.
SNRPA	19	0.95105	0.27273	0.48252	0.05594	0.25574	14669	0.0256	0.178	.....
TUSC5	17	0.94605	0.69131	0.41758	0.34166	0.04895	14669	0.02561	0.178	.....
ZFAND4	10	0.59640	0.80220	0.08192	NA	0.07692	11265	0.02562	0.178	...?.
LOC374443	12	0.89710	0.95105	0.26174	0.06094	0.17483	14669	0.02562	0.178	.....
LOC105369340	11	0.28072	NA	0.64036	0.05794	0.28272	13691	0.02564	0.178	.?...
ADAM2	8	0.01598	0.27572	0.03596	0.24875	0.85115	14669	0.02564	0.178	.....
KLHL8	4	0.61638	0.95904	0.97003	0.02198	0.19880	14669	0.02564	0.178	.....
FSD2	15	0.05694	0.30869	0.74226	0.73626	0.12488	14669	0.02564	0.178	.....
BAP1	3	0.61538	0.94605	0.23676	0.16583	0.13487	14669	0.02565	0.178	.....
TAS2R42	12	0.16484	0.06494	0.21778	0.62637	0.28472	14669	0.02566	0.178	.....
NDOR1	20	NA	NA	0.25774	NA	0.04795	8863	0.02567	0.178	??.?.
NUP188	9	0.21479	0.93506	0.44655	0.11189	0.23676	14669	0.02568	0.178	.....
SLC39A6	18	0.29471	0.97902	0.58242	0.86014	0.02298	14669	0.02568	0.178	.....
HEATR3	16	0.34565	0.50549	0.27572	NA	0.09690	11265	0.02568	0.178	...?.
B3GAT3	20	NA	0.40859	0.03297	0.20879	NA	5233	0.02569	0.178	?...?
INTS8	8	0.14386	0.70230	0.75425	NA	0.07293	11265	0.0257	0.178	...?.

METTL17	14	0.21978	0.72428	0.28971	0.05794	0.42557	14669	0.0257	0.178	.....
LINC00243	6	0.82418	0.44356	0.37163	0.72028	0.03297	14669	0.0257	0.178	.....
ABCA7	19	0.16583	0.29371	0.99201	0.03197	0.50549	14669	0.02571	0.178	.....
ZNF720	16	0.13287	0.97203	0.92208	0.32268	0.08991	14669	0.02571	0.178	.....
ZNF98	19	0.10390	0.13686	0.83816	0.40959	0.22677	14669	0.02571	0.178	.....
CLDN1	3	0.05095	0.56244	0.83417	0.61239	0.11089	14669	0.02572	0.178	.....
APLNR	11	0.97303	0.00280	0.83317	0.14685	0.34466	14669	0.02573	0.178	.....
MAGEF1	3	0.17982	NA	0.70629	NA	0.06593	10287	0.02573	0.178	.?.?.
PPT2-EGFL8	6	0.94206	0.22278	0.01180	0.19181	0.37363	14669	0.02573	0.178	.....
TTC41P	12	0.07393	0.14885	0.65135	0.11189	0.56444	14669	0.02574	0.178	.....
KIAA1671	22	0.08791	0.52847	0.95005	0.17982	0.23676	14669	0.02574	0.178	.....
ODC1	2	0.53946	0.33866	0.46953	0.20879	0.17083	14669	0.02574	0.178	.....
CUEDC1	17	0.76024	0.90909	0.79520	0.61139	0.01499	14669	0.02576	0.178	.....
USP32	17	0.00799	NA	0.16683	0.33067	0.47652	13691	0.02577	0.178	.?...
EDNRA	4	0.25674	0.96104	0.93706	0.84216	0.01798	14669	0.02578	0.178	.....
FAM234B	12	0.11688	0.23576	0.63836	0.60839	0.14585	14669	0.02578	0.178	.....
PAPD4	5	0.06094	0.45455	0.61039	0.28272	0.27173	14669	0.02578	0.178	.....
POT1-AS1	7	0.14885	0.03297	0.13586	NA	0.42158	11265	0.0258	0.178	...?.
RPSAP58	19	0.56344	0.55744	0.49750	0.81918	0.02797	14669	0.0258	0.178	.....
KCNJ6	21	0.10390	0.47752	0.28372	NA	0.17383	11265	0.02581	0.178	...?.
HACD4	9	0.48651	0.18482	0.84416	0.02797	0.44555	14669	0.02581	0.178	.....
LINC00692	3	0.61039	0.78222	0.10190	0.85714	0.03996	14669	0.02582	0.178	.....
SNRPB2	20	0.28472	0.34965	0.48951	0.18881	0.24775	14669	0.02585	0.178	.....
ASH2L	8	0.13586	0.84216	0.45854	0.01998	0.57642	14669	0.02586	0.178	.....
MIR548AN	13	0.01698	0.70629	0.28072	0.03596	0.87413	14669	0.02586	0.178	.....
CSF3	17	0.62138	0.58042	0.49950	0.08492	0.20380	14669	0.02586	0.178	.....
ANO2	12	0.87812	0.50849	0.26973	NA	0.04695	11265	0.02587	0.178	...?.
SMPD2	6	0.47053	0.01099	0.93207	0.37363	0.22478	14669	0.0259	0.178	.....
DHH	12	0.85714	0.10390	0.17982	0.39560	0.16783	14669	0.02591	0.178	.....
CKB	14	0.05495	0.32268	0.75924	0.96204	0.08392	14669	0.02592	0.178	.....
GAB4	22	0.06494	0.81718	0.78222	0.22977	0.19980	14669	0.02592	0.178	.....
SLC6A20	3	0.81319	0.24076	0.25774	0.00400	0.79520	14669	0.02592	0.178	.....
SLC2A2	3	0.34965	0.17483	0.43856	0.60440	0.11988	14669	0.02593	0.178	.....
KRT42P	17	0.55345	0.15185	0.31369	0.31469	0.20080	14669	0.02594	0.178	.....
CLRN1	3	0.52048	0.48352	0.31868	0.19081	0.18681	14669	0.02594	0.178	.....
NDUFAF6	8	0.52348	0.42058	0.21978	0.92907	0.04496	14669	0.02595	0.178	.....
LOC101929006	6	0.42557	0.98302	0.45654	NA	0.03996	11265	0.02598	0.178	...?.
PDIA6	2	0.02597	0.09590	0.27972	0.15684	0.82817	14669	0.02599	0.178	.....
PRADC1	2	0.01499	0.90909	0.54246	0.23277	0.33167	14669	0.02599	0.178	.....
C18orf21	18	0.73027	0.38561	0.39361	0.17682	0.15984	14669	0.02599	0.178	.....
CLYBL	13	0.85015	0.26174	0.42058	0.01110	0.53347	14669	0.026	0.178	.....
DNPH1	6	0.29670	0.20779	0.37862	0.17782	0.33367	14669	0.02601	0.178	.....
OR9G9	11	0.24476	0.75425	0.04496	0.21978	0.33766	14669	0.02602	0.178	.....
ABCG8	2	0.77522	0.41059	0.56643	0.54645	0.04296	14669	0.02602	0.178	.....
GALNT15	3	0.43457	0.25974	0.45455	0.08591	0.35465	14669	0.02602	0.178	.....
GZF1	20	0.51848	0.36364	0.02498	0.18282	0.41059	14669	0.02604	0.178	.....
SREK1	5	0.34366	0.18082	0.12488	0.23576	0.38362	14669	0.02606	0.178	.....
MEF2D	1	0.11289	0.25175	0.22278	0.15984	0.52947	14669	0.02606	0.178	.....
SERPINA1	14	0.98002	0.59141	0.01530	NA	0.09890	11265	0.02606	0.178	...?.
TRAPPC6A	19	0.79221	0.53546	0.46054	0.12388	0.14985	14669	0.02606	0.178	.....
MKRN7P	20	0.14386	NA	0.60240	0.57443	0.07892	13691	0.02606	0.178	.?...
SIL1	5	0.85814	0.20979	0.05095	0.32667	0.22178	14669	0.02607	0.178	.....
SNX21	20	0.50450	0.87213	0.07692	0.24076	0.18282	14669	0.02607	0.178	.....
PUS7L	12	0.30170	0.25874	0.52148	0.36064	0.16883	14669	0.02608	0.178	.....
ZNF671	19	0.48452	0.08092	0.69031	0.24875	0.22078	14669	0.02608	0.178	.....
LOC101928977	1	0.35265	0.54246	0.07093	0.08991	0.47652	14669	0.02609	0.178	.....
LOC101927168	18	0.51848	0.07992	0.93706	NA	0.08392	11265	0.02609	0.178	...?.
NOC2LP2	2	0.49451	0.11588	0.03397	0.40659	0.34665	14669	0.02609	0.178	.....
C7orf34	7	0.30470	0.07692	0.35165	0.15984	0.45954	14669	0.02611	0.178	.....
NR2F1-AS1	5	0.22877	0.26274	0.37263	0.22178	0.30569	14669	0.02612	0.178	.....
DUSP27	1	0.08791	0.13986	0.41259	0.48052	0.29471	14669	0.02612	0.178	.....
AOX2P	2	0.78422	0.51848	0.29171	0.16783	0.15385	14669	0.02613	0.178	.....
LINC01264	10	0.49550	0.39161	0.07792	NA	0.13986	11265	0.02613	0.178	...?.
SPTLC2	14	0.06893	0.18981	0.55345	NA	0.22278	11265	0.02614	0.178	...?.
LOC103312105	11	0.29071	0.62138	0.20180	0.16683	0.28272	14669	0.02615	0.178	.....
PPP1R3A	7	0.13686	0.92008	0.31568	0.10889	0.32967	14669	0.02616	0.178	.....
N6AMT1	21	0.95005	0.34466	0.50849	NA	0.03896	11265	0.02617	0.178	...?.
TMEM86A	11	0.29071	0.94206	0.95305	0.52947	0.03497	14669	0.02618	0.178	.....
SIDT2	11	0.15684	0.95904	0.07193	0.16883	0.36264	14669	0.02619	0.178	.....

NOD2	16	0.07093	0.09790	0.95804	NA	0.20080	11265	0.02621	0.178	...?.
PSMB5	14	0.56943	NA	0.42258	0.14486	0.14585	13691	0.02622	0.178	.?...
PKDREJ	22	0.35764	NA	0.16983	0.00990	0.69730	13691	0.02624	0.178	.?...
CLEC18A	16	0.04496	0.46254	0.16084	NA	0.28172	11265	0.02624	0.178	...?.
SLC38A7	16	0.08591	0.55844	0.36464	0.37562	0.22777	14669	0.02624	0.178	.....
TYR	11	0.84515	0.62138	0.74426	NA	0.02298	11265	0.02627	0.178	...?.
CTDSPL2	15	0.26274	0.76224	0.05495	0.89510	0.08591	14669	0.02628	0.178	.....
MTDH	8	0.25774	0.33367	0.52448	NA	0.10689	11265	0.02629	0.178	...?.
CHRAC1	8	0.05794	0.74825	0.03397	0.93107	0.17782	14669	0.02629	0.178	.....
OR5I1	11	0.80819	0.53546	0.71728	0.38661	0.04496	14669	0.0263	0.178	.....
SPDYE3	7	0.39660	0.94605	0.05195	0.30070	0.18781	14669	0.02631	0.178	.....
TOP2B	3	0.27473	0.18581	0.13686	0.59740	0.20979	14669	0.02632	0.178	.....
DLGAP5	14	0.94306	0.63736	0.52048	0.03497	0.21978	14669	0.02632	0.178	.....
NAB2	12	0.01060	0.49351	0.58741	0.93806	0.14386	14669	0.02633	0.178	.....
SLC10A4	4	0.72128	0.28671	0.08591	0.82817	0.07493	14669	0.02638	0.178	.....
MIR202HG	10	0.06294	NA	0.96004	0.56044	0.08492	13691	0.02639	0.178	.?...
DZIP3	3	0.11389	0.55644	0.14785	0.81319	0.13187	14669	0.02639	0.178	.....
MDM2	12	0.70929	0.25475	0.48052	0.15684	0.19481	14669	0.0264	0.178	.....
ZNF75A	16	0.26074	0.25375	0.39061	0.09590	0.44356	14669	0.0264	0.178	.....
NUMBL	19	0.81518	0.72028	0.02697	0.27672	0.17682	14669	0.02641	0.178	.....
MFSD8	4	0.13686	0.07792	0.79421	0.25475	0.35065	14669	0.02643	0.178	.....
ZNF669	1	0.73826	0.91508	0.12288	0.26673	0.10989	14669	0.02644	0.178	.....
CHI3L2	1	0.29870	0.53646	0.12388	0.21479	0.29870	14669	0.02644	0.178	.....
MYLK3	16	0.00030	0.63437	0.13087	0.64735	0.60140	14669	0.02645	0.178	.....
CCL4	17	0.67033	NA	0.91908	0.35564	0.03696	13691	0.02648	0.178	.?...
CDIPT-AS1	16	0.02498	0.71528	0.70230	0.91309	0.08292	14669	0.0265	0.178	.....
ZNF212	7	0.52448	0.50749	0.24476	0.22877	0.18082	14669	0.02653	0.178	.....
ADIPOR1	1	0.52448	0.16384	0.56344	0.45155	0.11688	14669	0.02653	0.178	.....
IQCE	7	0.32867	0.50250	0.08292	0.08492	0.50450	14669	0.02653	0.178	.....
OR4A15	11	0.59141	NA	0.50250	0.05295	0.23277	13691	0.02654	0.178	.?...
MARK3	14	0.11988	0.17383	0.95804	0.22178	0.29071	14669	0.02657	0.178	.....
LOC389602	7	0.38961	NA	0.33666	0.02697	0.43457	13691	0.02658	0.178	.?...
SOS2	14	0.02797	0.18082	0.09291	0.18082	0.85215	14669	0.0266	0.178	.....
LOC441025	4	0.02398	0.99500	0.80120	0.09990	0.37263	14669	0.02661	0.178	.....
DPYSL3	5	0.03197	0.75524	0.17682	0.89610	0.14386	14669	0.02661	0.178	.....
CCDC86	11	0.65235	0.06394	0.58741	0.69930	0.08292	14669	0.02662	0.178	.....
ANKRD45	1	0.08292	0.13187	0.66234	0.07093	0.67233	14669	0.02662	0.178	.....
LINC00390	13	0.06893	0.26573	0.67033	0.53047	0.19481	14669	0.02663	0.178	.....
AGER	6	0.13287	0.37562	0.43556	0.77622	0.10589	14669	0.02664	0.178	.....
GBP1P1	1	0.58841	0.92208	0.12088	0.03896	0.37562	14669	0.02664	0.178	.....
LHFPL4	3	0.53147	0.28571	0.14985	0.11688	0.38362	14669	0.02664	0.178	.....
CFHR3	1	0.17582	0.44955	0.06793	0.53047	0.24575	14669	0.02664	0.178	.....
LINC01222	1	0.50350	0.15385	0.16184	0.11988	0.45355	14669	0.02664	0.178	.....
VPS4A	16	0.13087	0.45854	0.36464	0.57642	0.14885	14669	0.02666	0.178	.....
LOC101926905	5	0.43257	0.50549	0.89111	0.04595	0.27572	14669	0.02666	0.178	.....
NAMPT	7	0.25974	0.59740	0.07692	0.31369	0.26873	14669	0.02667	0.178	.....
H1FX-AS1	3	0.04695	0.78621	0.46054	0.04096	0.62238	14669	0.02674	0.179	.....
CYP17A1	10	0.99500	0.40859	0.19381	0.50949	0.06394	14669	0.02674	0.179	.....
GCNT4	5	0.00610	NA	0.78921	0.99201	0.09990	13691	0.02678	0.179	.?...
NPR1	1	0.51149	0.70829	0.65534	0.93107	0.01698	14669	0.0268	0.179	.....
OR2W5	1	0.61738	0.16484	0.63536	0.07193	0.33067	14669	0.02681	0.179	.....
OR4S2	20	NA	0.47652	0.16683	0.40759	0.10789	13245	0.02682	0.179	?....
CLINT1	5	0.01898	0.70030	0.58641	0.60440	0.17483	14669	0.02685	0.179	.....
KLHL41	2	0.52348	0.70330	0.01499	0.26973	0.27972	14669	0.02686	0.179	.....
ZNF512	2	0.30569	0.53147	0.22977	0.64735	0.09790	14669	0.02686	0.179	.....
ZFP62	5	0.15285	0.90609	0.61938	NA	0.06893	11265	0.02686	0.179	...?.
SEMA4F	2	0.14785	0.41758	0.29570	0.19281	0.36164	14669	0.02686	0.179	.....
ZNF354A	5	0.10789	0.20080	0.25175	0.98901	0.13686	14669	0.02688	0.179	.....
GPR162	12	0.11588	NA	0.95205	NA	0.06793	10287	0.02688	0.179	.?.?.
ALDH3A1	17	0.30869	0.79720	0.70829	0.10290	0.19680	14669	0.02688	0.179	.....
SIX3	2	0.90909	0.27772	0.26274	0.28072	0.13387	14669	0.02689	0.179	.....
CFH	1	0.31369	0.09890	0.55145	0.09990	0.46753	14669	0.0269	0.179	.....
PQLC3	2	0.42458	0.42657	0.48252	0.38561	0.11289	14669	0.02691	0.179	.....
LOC100128006	17	0.24775	0.53347	0.22278	0.00830	0.86414	14669	0.02693	0.179	.....
GSTA1	6	0.15085	0.33566	0.03297	0.72727	0.25874	14669	0.02693	0.179	.....
ERICH6-AS1	3	0.40260	0.34366	0.75824	0.11189	0.24076	14669	0.02695	0.179	.....
CCL17	16	0.21279	0.27572	0.58641	0.01399	0.75824	14669	0.02696	0.179	.....
NOTO	2	0.08591	0.79121	0.11688	0.52847	0.20979	14669	0.02699	0.179	.....
PMPCA	9	0.78222	0.04595	0.37363	NA	0.12887	11265	0.027	0.179	...?.

HCG26	6	0.80919	0.70829	0.77023	0.16384	0.07892	14669	0.02701	0.179	.....
SRRM3	7	0.91009	0.33666	0.19081	NA	0.06993	11265	0.02702	0.179	...?.
RSU1	10	0.46953	0.80819	0.98701	0.24076	0.06693	14669	0.02703	0.179	.....
SNX16	8	0.82118	0.12687	0.30370	0.97203	0.04496	14669	0.02703	0.179	.....
ERCC6L2	9	0.17782	0.01380	0.82218	NA	0.26274	11265	0.02704	0.179	...?.
SKAP1	17	0.21179	0.44156	0.29570	0.24875	0.26973	14669	0.02704	0.179	.....
RMDN2	2	NA	0.36663	0.08791	0.38861	0.15884	13245	0.02705	0.179	?....
WDHD1	14	0.42957	0.37363	0.21878	0.63836	0.10090	14669	0.02705	0.179	.....
ZNF214	11	0.00240	0.10390	0.69031	0.98002	0.31868	14669	0.02705	0.179	.....
FAM151B	5	0.70929	0.40659	0.00510	0.09391	0.58941	14669	0.02705	0.179	.....
C12orf77	12	0.20280	0.93706	0.72927	NA	0.05195	11265	0.02708	0.179	...?.
TSR1	17	0.38861	0.55644	0.95405	0.12987	0.15684	14669	0.02708	0.179	.....
MZT1	13	0.06194	0.75824	0.23377	NA	0.18082	11265	0.02709	0.179	...?.
CEP131	17	0.25674	0.52947	0.54645	0.06294	0.37662	14669	0.02709	0.179	.....
DACT1	14	0.57742	0.48751	0.85714	0.04595	0.24875	14669	0.0271	0.179	.....
LOC105747689	2	0.26374	0.67632	0.62438	0.18382	0.17782	14669	0.0271	0.179	.....
CD300E	17	0.17083	0.99600	0.84116	0.91209	0.02298	14669	0.02711	0.179	.....
PDGFC	4	0.11089	0.49351	0.97502	0.80020	0.05694	14669	0.02711	0.179	.....
LINC01509	9	0.27473	0.40559	0.74825	0.43556	0.10390	14669	0.02712	0.179	.....
SULF2	20	0.15185	0.52647	0.80819	0.04296	0.44356	14669	0.02712	0.179	.....
RRAGD	6	0.52048	0.03097	0.90210	NA	0.11988	11265	0.02713	0.179	...?.
H2AFX	11	0.31568	0.02198	0.52348	0.53946	0.24675	14669	0.02715	0.179	.....
DNAJC9-AS1	10	0.63237	0.73826	0.46753	NA	0.03896	11265	0.02715	0.179	...?.
LOC101927881	2	0.47453	0.39461	0.35465	0.02198	0.55145	14669	0.02716	0.179	.....
IL6	7	0.91409	0.00670	0.28272	NA	0.20180	11265	0.02717	0.179	...?.
LOC101927153	15	0.36064	0.18282	0.07093	0.77123	0.17383	14669	0.02718	0.179	.....
SSX2IP	1	0.16783	0.20380	0.14286	0.36064	0.37363	14669	0.0272	0.179	.....
MIS12	17	0.58242	0.68631	0.04496	0.67732	0.09091	14669	0.02721	0.179	.....
NOL4	18	0.29171	0.75924	0.41558	0.56843	0.07193	14669	0.02721	0.179	.....
AKT1	14	0.18881	0.24476	0.73227	0.31169	0.20879	14669	0.02721	0.179	.....
UBXN10	1	0.34066	NA	0.27972	0.25175	0.16583	13691	0.02722	0.179	.?...
SPATA22	17	0.01499	0.44256	0.87912	0.42358	0.26174	14669	0.02726	0.179	.....
LINC01598	20	0.09191	NA	0.91409	0.83616	0.04496	13691	0.02728	0.179	.?...
TMUB1	7	0.62537	0.19580	0.78721	0.23177	0.14386	14669	0.02729	0.179	.....
TRPM5	11	0.60739	0.49950	0.56344	0.14086	0.16583	14669	0.02731	0.179	.....
IL17RA	22	0.35265	0.94805	0.95904	0.06194	0.18082	14669	0.02731	0.179	.....
SYT4	18	0.00370	NA	0.09690	0.75225	0.36064	13691	0.02733	0.179	.?...
RAP2B	3	0.46853	0.04096	0.49251	0.05594	0.61938	14669	0.02733	0.179	.....
B4GALT7	5	0.46154	0.23676	0.25974	0.14186	0.34865	14669	0.02734	0.179	.....
FGFR4	5	0.19481	NA	0.27173	0.22777	0.23277	13691	0.02736	0.179	.?...
SPATA19	11	0.03796	0.57642	0.68232	NA	0.15784	11265	0.02738	0.179	...?.
HECTD3	1	0.43956	0.25475	0.43656	0.85514	0.05894	14669	0.02738	0.179	.....
KCNK17	6	0.43956	0.60539	0.66633	NA	0.04895	11265	0.02738	0.179	...?.
PVALB	22	0.23976	0.71429	0.33167	0.05894	0.41558	14669	0.02738	0.179	.....
ZSCAN5A	19	0.84116	0.89710	0.86114	0.12088	0.07393	14669	0.02739	0.179	.....
RPE	20	NA	0.62038	0.33067	0.01698	0.42957	13245	0.02741	0.179	?....
PANK4	1	0.46454	0.90010	0.65135	0.12787	0.13586	14669	0.02747	0.180	.....
HYAL3	3	0.61039	0.80120	0.52847	0.59940	0.03297	14669	0.02748	0.180	.....
SIRT2	19	0.12887	0.09690	0.17483	0.61538	0.32068	14669	0.02749	0.180	.....
TJP1	15	0.37862	0.12288	0.89610	0.46254	0.12088	14669	0.0275	0.180	.....
CYP2D7	22	0.26973	0.03596	0.57842	0.70529	0.17483	14669	0.02751	0.180	.....
SPTBN5	15	0.00770	0.03596	0.54046	0.42258	0.68631	14669	0.02752	0.180	.....
LRRC37B	17	0.24276	NA	0.52647	0.62837	0.06194	13691	0.02752	0.180	.?...
FNDC1	6	0.58841	0.15784	0.81119	0.09291	0.27672	14669	0.02752	0.180	.....
ZSCAN5B	19	0.45255	NA	0.61439	0.04795	0.26673	13691	0.02755	0.180	.?...
IL3	5	0.83516	0.96903	0.74725	0.06793	0.11289	14669	0.02756	0.180	.....
ENC1	5	0.57642	NA	0.37962	0.01099	0.48352	13691	0.02758	0.180	.?...
CYP11B1	8	0.74026	NA	0.23477	0.21379	0.12288	13691	0.02758	0.180	.?...
KLHL24	3	0.98302	0.57043	0.16883	0.51149	0.05794	14669	0.02765	0.180	.....
RAB3C	5	NA	0.09590	0.06593	NA	0.20779	9841	0.02765	0.180	?..?.
MGAT4A	2	0.36863	0.28272	0.18881	0.98002	0.07493	14669	0.02765	0.180	.....
CTHRC1	8	0.48452	0.77622	0.88511	0.12987	0.12088	14669	0.02767	0.180	.....
HSPA4L	4	0.12787	0.05195	0.34565	0.60739	0.30969	14669	0.02769	0.180	.....
PIGR	1	0.12587	0.38362	0.71928	0.10190	0.39760	14669	0.0277	0.180	.....
HLA-B	6	0.07493	0.94805	0.49351	0.67233	0.08791	14669	0.0277	0.180	.....
CDHR2	5	0.08292	0.90909	0.00800	0.15485	0.67732	14669	0.02771	0.180	.....
LOC645513	4	0.09990	0.85315	0.30769	0.06693	0.48851	14669	0.02776	0.180	.....
PHLDB2	3	0.39061	0.32967	0.55345	0.23177	0.19281	14669	0.02778	0.180	.....
E2F5	8	0.85514	0.31968	0.94805	0.73227	0.02098	14669	0.02779	0.180	.....

GDNF-AS1	5	0.70829	0.70929	0.10989	0.00440	0.75524	14669	0.02779	0.180	.....
INCA1	17	0.97103	0.41459	0.29870	0.57143	0.04895	14669	0.0278	0.180	.....
LINC01602	8	0.57942	0.33367	0.47752	NA	0.07093	11265	0.0278	0.180	...?.
FAM172A	5	0.46753	0.53546	0.54046	0.53047	0.06494	14669	0.0278	0.180	.....
CA9	9	0.00490	0.67532	0.97502	0.24176	0.38362	14669	0.0278	0.180	.....
ZNF555	19	0.47053	0.56444	0.02597	0.77023	0.11988	14669	0.0278	0.180	.....
LRRC37A2	17	0.38961	NA	0.06793	0.73926	0.08891	13691	0.02781	0.180	.?...
SIPA1L2	1	0.52248	0.24875	0.75524	0.42757	0.09191	14669	0.02781	0.180	.....
HTT-AS	4	0.50749	0.62438	0.39261	0.37163	0.09590	14669	0.02782	0.180	.....
IFNA17	9	0.99001	0.95105	0.13786	0.07792	0.18781	14669	0.02783	0.180	.....
LOC284825	21	0.08791	0.37163	NA	NA	0.13986	10414	0.02783	0.180	..??.
RAD52	12	0.35964	0.07692	0.94006	0.55644	0.11788	14669	0.02786	0.180	.....
PRDM5	4	NA	0.04895	0.69331	0.40559	0.14186	13245	0.02787	0.180	?....
SELV	19	0.44855	0.67732	0.38462	0.12288	0.22178	14669	0.02787	0.180	.....
CASC11	8	0.06593	NA	0.58741	0.33467	0.19580	13691	0.02788	0.180	.?...
PKP1	1	NA	0.18082	0.28272	0.02498	0.58342	13245	0.02788	0.180	?....
LGALS3	14	0.85015	0.45455	0.71229	0.05594	0.20480	14669	0.0279	0.180	.....
APMAP	20	0.07692	0.50150	0.16284	0.38961	0.32767	14669	0.0279	0.180	.....
CDH24	14	NA	0.42158	0.11988	0.06593	0.40060	13245	0.02791	0.180	?....
LOC101929380	5	0.36464	0.04995	0.46553	0.37363	0.27572	14669	0.02791	0.180	.....
FAM92B	16	0.16384	0.95205	0.12587	0.07493	0.47952	14669	0.02792	0.180	.....
SYS1	20	0.00130	0.82517	0.13786	0.56743	0.49351	14669	0.02795	0.180	.....
MERTK	2	0.58442	0.47153	0.46853	0.66034	0.04895	14669	0.02796	0.180	.....
UBE2T	1	0.39261	0.25175	0.25275	0.58442	0.13786	14669	0.02796	0.180	.....
GOLGA6L3	20	NA	NA	NA	0.02797	NA	3404	0.02797	0.180	???.?
RBP2	3	0.95305	0.28472	0.07892	0.01000	0.74625	14669	0.02797	0.180	.....
C11orf96	11	0.08691	0.76324	0.43856	NA	0.12687	11265	0.02797	0.180	...?.
LOC101929468	4	0.63636	0.50649	0.35864	0.81019	0.03696	14669	0.02799	0.180	.....
RPS8	1	0.63437	0.26074	0.62338	0.87912	0.03397	14669	0.028	0.180	.....
COX5A	15	0.48651	0.20080	0.27173	NA	0.13187	11265	0.02801	0.180	...?.
LEKR1	3	0.67433	0.12787	0.91908	0.46753	0.07892	14669	0.02802	0.180	.....
REPIN1	7	0.54645	0.14885	0.41259	0.17183	0.29071	14669	0.02802	0.180	.....
DYRK1B	19	0.28172	0.84316	0.39860	0.08691	0.28971	14669	0.02803	0.180	.....
ANKRD36	2	0.81818	0.77023	0.97702	0.71628	0.01150	14669	0.02804	0.180	.....
BRPF1	3	0.32368	0.34066	0.09391	0.27572	0.33367	14669	0.02805	0.180	.....
ISG20	15	0.47353	0.23676	0.50450	0.10789	0.32168	14669	0.02806	0.180	.....
AK1	9	0.13087	NA	0.02697	0.11588	0.62537	13691	0.02808	0.180	.?...
TTC32	2	0.36963	0.42058	0.09790	0.63337	0.14386	14669	0.02809	0.180	.....
ANGPT1	8	0.65634	0.59840	NA	0.58641	0.03097	13818	0.0281	0.180	..?..
NALCN-AS1	13	0.65235	0.10090	0.72827	0.33966	0.14086	14669	0.0281	0.180	.....
TRAPPC3L	6	0.15185	0.85415	0.24875	NA	0.11888	11265	0.0281	0.180	...?.
DCLK1	13	0.22777	0.13586	0.13387	NA	0.27772	11265	0.02812	0.180	...?.
COG6	13	0.69131	0.70729	0.10390	NA	0.07592	11265	0.02812	0.180	...?.
FAM166A	20	NA	NA	0.83616	NA	0.02498	8863	0.02813	0.180	??.?.
RPL3	22	0.45455	0.59940	0.17782	0.44855	0.12388	14669	0.02813	0.180	.....
HIPK3	11	0.84715	0.46054	0.81019	0.03497	0.23876	14669	0.02817	0.181	.....
GSDMB	17	0.55944	0.55844	0.83317	0.71429	0.02697	14669	0.02818	0.181	.....
LOC642943	9	0.02997	0.00880	0.28771	0.53946	0.69431	14669	0.02818	0.181	.....
DEFB114	6	0.75724	0.50849	0.12787	0.06394	0.36563	14669	0.02818	0.181	.....
FBLIM1	1	0.51349	0.21479	0.68831	0.97403	0.03497	14669	0.02819	0.181	.....
TMED4	7	0.23776	0.92607	0.09291	0.44655	0.16284	14669	0.02819	0.181	.....
SLC43A2	17	0.21079	0.13387	0.36464	0.24575	0.38661	14669	0.02821	0.181	.....
SCN4A	17	0.92807	0.05195	0.38861	0.21379	0.24575	14669	0.02821	0.181	.....
LOC100294362	17	0.40559	0.39461	0.14885	0.05395	0.55245	14669	0.02823	0.181	.....
FAM53A	4	0.06394	0.97902	0.48052	0.13287	NA	6657	0.02825	0.181	....?
NKX2-8	14	0.80120	0.63636	0.02498	NA	0.10989	11265	0.02829	0.181	...?.
TTN	2	0.31668	0.74426	0.32667	0.04895	0.40759	14669	0.02831	0.181	.....
CELA3B	1	0.87413	0.22577	0.65035	0.49151	0.05994	14669	0.02831	0.181	.....
SNAP25-AS1	20	0.27772	0.52148	0.19281	0.22478	0.28472	14669	0.02832	0.181	.....
CERCAM	9	0.97602	0.84116	0.37662	0.08791	0.13786	14669	0.02833	0.181	.....
LPAL2	6	0.13187	0.17483	0.37363	0.36164	0.33067	14669	0.02835	0.181	.....
VPS36	13	0.01698	0.15984	0.53147	0.25175	0.60440	14669	0.02835	0.181	.....
HSPA12B	20	0.56144	0.27473	0.58641	0.13487	0.23477	14669	0.02835	0.181	.....
LOC101928796	21	0.01598	0.21978	0.17083	0.43556	0.57043	14669	0.02836	0.181	.....
TRIM65	17	0.39860	0.05794	0.95504	0.16983	0.30070	14669	0.02836	0.181	.....
PCF11-AS1	11	0.88711	0.53546	0.25774	0.90310	0.02498	14669	0.02836	0.181	.....
FIBP	11	0.72527	0.35365	0.30569	NA	0.07293	11265	0.02837	0.181	...?.
ZNF738	19	0.37762	0.49750	0.42757	0.21678	0.19580	14669	0.02837	0.181	.....
CEACAM3	19	0.28172	NA	0.68631	0.00780	0.58541	13691	0.02839	0.181	.?...

PPP1R3B	8	0.15984	0.09790	0.64436	0.22378	0.39461	14669	0.0284	0.181	.....
LOC102725080	8	0.04296	0.95305	0.19181	0.08192	0.59441	14669	0.0284	0.181	.....
FLJ27354	1	0.11089	0.73826	0.71429	0.90310	0.04795	14669	0.02842	0.181	.....
LCN6	9	0.12987	0.65035	0.75025	0.29770	0.16583	14669	0.02843	0.181	.....
LINC01372	7	0.23277	0.29770	0.24276	NA	0.17682	11265	0.02844	0.181	...?.
RPL26L1	5	0.00430	0.64835	0.16883	NA	0.43157	11265	0.02845	0.181	...?.
ALOX12B	17	0.34266	0.91908	0.11489	0.41159	0.13886	14669	0.02845	0.181	.....
JAG2	14	0.20979	0.96004	0.85814	0.04895	0.28172	14669	0.02847	0.181	.....
ASRGL1	11	0.45954	0.05395	0.91109	0.69930	0.09091	14669	0.02849	0.181	.....
C14orf39	14	0.72527	0.76024	0.63836	0.23077	0.07592	14669	0.02849	0.181	.....
C12orf66	12	0.15185	0.19680	0.88611	NA	0.13487	11265	0.02851	0.181	...?.
LOC101929406	1	0.01199	0.71728	0.96503	0.62737	0.14785	14669	0.02851	0.181	.....
PPM1M	3	0.38462	0.16484	0.40460	0.11988	0.40859	14669	0.02851	0.181	.....
ZNF441	19	0.06394	0.28472	0.29071	0.26973	0.45654	14669	0.02853	0.181	.....
C12orf73	12	0.23676	0.17582	0.91608	0.09191	0.39061	14669	0.02853	0.181	.....
KDELC1	13	0.32667	0.41858	0.91808	0.01798	0.48951	14669	0.02854	0.181	.....
DMP1	4	0.75125	0.91708	0.89910	0.07692	0.10989	14669	0.02854	0.181	.....
RALGDS	9	0.07493	0.74625	0.09291	0.60739	0.22378	14669	0.02854	0.181	.....
HERC2	15	0.22078	0.67732	0.18082	0.28871	0.24076	14669	0.02857	0.181	.....
DDX42	17	0.65534	0.86913	0.06094	NA	0.08192	11265	0.02857	0.181	...?.
LOC105378663	1	0.26873	NA	0.33167	0.15884	0.24575	13691	0.02858	0.181	.?...
TSSC2	11	0.20080	0.20579	0.71828	0.02398	0.69530	14669	0.02859	0.181	.....
MRGPRE	11	0.11189	0.80420	0.74725	0.03297	0.47752	14669	0.02859	0.181	.....
ANKRD44	2	0.40759	0.57842	0.94705	0.17782	0.12887	14669	0.0286	0.181	.....
ARHGAP33	19	0.55445	0.31868	0.53147	0.01199	0.60140	14669	0.02861	0.181	.....
GJB4	1	0.39161	0.08492	0.21379	NA	0.21978	11265	0.02861	0.181	...?.
FIBIN	11	0.21878	0.39760	0.87612	0.08292	NA	6657	0.02862	0.181	....?
LOC105369879	12	0.43457	0.03796	0.82917	0.44456	0.18082	14669	0.02863	0.181	.....
ZNF564	19	0.77223	0.02098	0.14086	0.44056	0.29371	14669	0.02864	0.181	.....
MNDA	1	0.03996	0.60539	0.32567	0.16883	0.48551	14669	0.02864	0.181	.....
FBXO4	5	0.32468	0.86613	0.20979	NA	0.08691	11265	0.02865	0.181	...?.
F2R	5	0.91708	0.15584	0.09990	NA	0.12887	11265	0.02866	0.181	...?.
DERL3	22	0.21678	0.53546	0.22378	0.07692	0.50050	14669	0.02868	0.181	.....
DDB2	11	0.17582	0.94206	0.98601	0.04196	0.30170	14669	0.02868	0.181	.....
KIF25	6	0.74525	0.15185	0.12887	NA	0.14386	11265	0.02869	0.181	...?.
LINC01333	5	0.74925	0.95005	0.01499	0.15385	0.27972	14669	0.0287	0.181	.....
HPN-AS1	19	0.29770	0.52148	0.77822	0.16883	0.19081	14669	0.02871	0.181	.....
SUSD6	14	0.18182	0.31568	0.03097	NA	0.32867	11265	0.02873	0.181	...?.
ANGPTL6	20	NA	NA	0.85714	NA	0.02498	8863	0.02873	0.181	??.?.
NAE1	16	0.94206	0.98402	0.17183	0.06194	0.20779	14669	0.02873	0.181	.....
SBNO2	19	0.57443	0.18981	0.71928	0.89910	0.03896	14669	0.02874	0.181	.....
DUOX1	15	0.84216	0.05395	0.96204	0.96503	0.03097	14669	0.02876	0.181	.....
C4orf3	4	0.36264	0.94006	0.53646	0.32567	0.08791	14669	0.02877	0.181	.....
HELQ	4	0.13686	NA	0.11189	0.54645	0.18681	13691	0.02877	0.181	.?...
BEND5	1	0.94605	0.51648	0.49351	0.05994	0.20979	14669	0.02877	0.181	.....
ZFP30	19	0.89411	0.11189	0.52747	0.50549	0.08791	14669	0.02881	0.181	.....
CCR8	3	0.14585	0.42757	0.57742	0.42258	0.17283	14669	0.02882	0.181	.....
FAT4	4	0.36464	0.32068	0.21379	0.20380	0.31968	14669	0.02882	0.181	.....
CEP162	6	0.70629	0.14785	0.75325	0.04296	0.38362	14669	0.02883	0.181	.....
NOS1AP	1	NA	0.87812	0.45654	0.03397	0.25774	13245	0.02884	0.181	?....
INPP5J	22	0.25974	0.23277	0.92408	0.67732	0.07792	14669	0.02885	0.181	.....
LOC101927379	5	0.60240	0.96204	0.39061	NA	0.03796	11265	0.02886	0.181	...?.
PRC1	15	0.79121	0.68631	0.53746	0.12488	0.13287	14669	0.02887	0.181	.....
DNAH12	3	0.31868	0.42158	0.53347	0.66334	0.07692	14669	0.02888	0.181	.....
MGC27345	7	0.29171	0.79021	0.75524	NA	0.05095	11265	0.02888	0.181	...?.
CPED1	7	0.28372	0.27772	0.68232	NA	0.10390	11265	0.02889	0.181	...?.
MAP1B	5	0.20579	0.30370	0.65035	0.80719	0.07892	14669	0.02889	0.181	.....
SMARCD2	17	0.94406	0.84016	0.11788	0.35165	0.08192	14669	0.02889	0.181	.....
LINC01471	3	0.17383	0.38961	0.37063	0.08891	0.49550	14669	0.02891	0.181	.....
MGC16275	17	0.10889	0.85215	0.53147	NA	0.09990	11265	0.02893	0.181	...?.
MPZL2	11	0.32368	0.60140	0.09491	NA	0.14785	11265	0.02894	0.181	...?.
ST3GAL4	11	0.34765	0.66833	0.68531	0.05095	0.31469	14669	0.02895	0.181	.....
FOXD2-AS1	1	0.02198	0.03497	0.90609	0.36763	0.53047	14669	0.02895	0.181	.....
COQ10A	12	0.30270	NA	0.66034	0.34765	0.09590	13691	0.02896	0.181	.?...
ACTG1	17	0.15884	0.54346	0.54545	0.26973	0.21778	14669	0.02896	0.181	.....
FOXG1	14	0.02897	NA	NA	NA	NA	1424	0.02897	0.181	.????
NUP62	19	0.18382	0.33367	0.21379	0.01880	0.90509	14669	0.02903	0.181	.....
ANK3	10	0.14985	0.17083	0.34865	NA	NA	3253	0.02903	0.181	...??
CYP1B1-AS1	2	0.41958	0.26074	0.21079	0.49650	0.17083	14669	0.02904	0.181	.....

PHB	17	0.27772	0.25375	0.16084	0.26274	0.36264	14669	0.02904	0.181	.....
CDIPT	16	0.02398	0.88212	0.73327	0.72827	0.10689	14669	0.02906	0.181	.....
LINC01478	18	0.34266	0.78422	0.82418	0.07493	0.22078	14669	0.02906	0.181	.....
ABCC3	17	0.21179	0.25774	0.73726	0.02498	0.64535	14669	0.0291	0.181	.....
XBP1	22	0.76823	0.93506	0.67832	0.02498	0.22877	14669	0.0291	0.181	.....
ECE2	3	0.50849	0.00057	0.11788	0.60639	0.53247	14669	0.02912	0.181	.....
DNAI1	9	0.42358	0.86613	0.47453	0.56943	0.05095	14669	0.02913	0.181	.....
MCC	5	0.27073	0.10090	0.18781	NA	0.26274	11265	0.02915	0.182	...?.
TMPRSS11GP	4	0.06593	0.85115	0.08192	0.31868	0.37163	14669	0.02918	0.182	.....
SYS1-DBNDD2	20	0.00220	0.69730	0.15584	0.57143	0.48851	14669	0.0292	0.182	.....
PCDHB12	5	0.87413	0.34166	0.56543	0.54446	0.04895	14669	0.02922	0.182	.....
TAL2	9	0.19081	NA	0.51249	0.38462	0.12887	13691	0.02923	0.182	.?...
CSMD2	1	0.94406	0.94705	0.71628	0.29570	0.03596	14669	0.02924	0.182	.....
PUS10	2	0.17782	0.79421	0.85015	0.09890	0.24875	14669	0.02925	0.182	.....
RAB3D	19	0.35065	NA	0.08492	0.11389	0.38761	13691	0.02928	0.182	.?...
ZNRF2P2	7	0.03896	0.43057	0.56543	NA	0.21079	11265	0.02928	0.182	...?.
MUL1	1	0.74426	0.07692	0.49351	0.20879	0.24975	14669	0.0293	0.182	.....
SUMF2	7	0.52048	0.31768	0.91808	0.88112	0.02797	14669	0.02932	0.182	.....
CD300A	17	0.00740	0.33467	0.31868	0.20879	0.73526	14669	0.02932	0.182	.....
TSC22D4	7	0.52947	0.88112	0.06194	0.19381	0.23976	14669	0.02934	0.182	.....
GIGYF2	20	NA	0.50949	0.16384	0.36164	0.12787	13245	0.02935	0.182	?....
LOC101927342	1	0.86414	0.10789	0.42358	0.00250	0.93407	14669	0.02935	0.182	.....
DGKQ	4	0.31469	0.31968	0.03596	0.07093	0.75225	14669	0.02936	0.182	.....
MORN2	2	0.74026	0.32667	0.01199	0.14086	0.49251	14669	0.02938	0.182	.....
LINC00994	3	0.04795	0.25075	0.14086	0.30270	0.57542	14669	0.02941	0.182	.....
CLDN19	1	0.16883	0.91808	0.44855	0.22877	0.19381	14669	0.02942	0.182	.....
CRB1	1	0.83017	0.66034	0.91409	0.07692	0.12987	14669	0.02943	0.182	.....
CDK5R2	20	NA	NA	0.56543	0.50749	0.03796	12267	0.02943	0.182	??...
NARS	18	0.89510	0.93506	0.84116	NA	0.01499	11265	0.02944	0.182	...?.
ZNRF1	16	0.16683	0.09790	0.77822	NA	0.18382	11265	0.02944	0.182	...?.
FANCG	9	0.37363	0.82817	0.29371	0.18881	0.19481	14669	0.02945	0.182	.....
L2HGDH	14	0.22378	0.65435	0.10290	0.21179	0.35165	14669	0.02946	0.182	.....
PPP2CB	8	0.77722	0.36064	0.63337	0.08591	0.21778	14669	0.02947	0.182	.....
PRX	19	0.99600	0.01798	0.51648	0.12388	0.36663	14669	0.02947	0.182	.....
TMCO6	5	0.10290	0.26873	0.03796	0.92807	0.25574	14669	0.02948	0.182	.....
LINC01020	5	0.62837	0.20979	0.16583	NA	0.13686	11265	0.02948	0.182	...?.
KRT79	12	0.84815	0.25974	0.17283	0.21079	0.22577	14669	0.02948	0.182	.....
PSMB11	14	NA	NA	0.13187	0.03497	0.40759	12267	0.02948	0.182	??...
LY75-CD302	2	0.43656	0.98202	0.82817	0.36763	0.05095	14669	0.02948	0.182	.....
SLC10A6	4	0.88611	0.05994	0.66334	0.79820	0.05495	14669	0.02949	0.182	.....
CDCA4	14	0.34865	0.17283	0.03696	0.09291	0.76523	14669	0.02951	0.182	.....
SMC2-AS1	9	0.10789	0.50050	0.01798	0.50150	0.41059	14669	0.02951	0.182	.....
SERPINA9	14	0.80619	0.08991	0.19780	NA	0.14286	11265	0.02951	0.182	...?.
LOC101927964	10	0.24675	0.60440	0.66134	0.13886	0.24276	14669	0.02952	0.182	.....
ASNS	7	0.07293	0.53447	0.83716	0.94505	0.06294	14669	0.02954	0.182	.....
FOXO1	13	0.64935	0.45055	0.10090	NA	0.11189	11265	0.02959	0.182	...?.
ARL6IP6	2	0.89411	0.37962	0.95904	0.01698	0.31369	14669	0.02961	0.182	.....
SH3BP4	20	NA	0.14286	0.27273	0.38661	0.17283	13245	0.02961	0.182	?....
LOC105374972	6	0.28571	0.92807	0.35065	NA	0.07293	11265	0.02963	0.182	...?.
SSTR1	14	0.87612	0.19081	0.67433	NA	0.05495	11265	0.02963	0.182	...?.
MYCNOS	2	0.40559	0.51449	0.01499	0.44955	0.28072	14669	0.02964	0.182	.....
SYT3	19	0.03796	0.27872	0.31269	0.26773	0.52348	14669	0.02964	0.182	.....
THNSL1	10	0.22078	0.21578	0.28971	NA	0.19880	11265	0.02965	0.182	...?.
CAPZA1	1	0.76723	0.61339	0.86813	0.45255	0.03596	14669	0.02966	0.182	.....
CDC20B	5	0.19580	0.73127	0.14985	NA	0.14885	11265	0.02968	0.182	...?.
SLF2	10	0.84216	0.01598	0.74226	0.35664	0.19081	14669	0.02969	0.182	.....
44257	20	NA	NA	NA	NA	NA	14669	0.02969	0.182	.....
CORO1B	11	0.57043	0.10490	0.78621	0.29171	0.17383	14669	0.02972	0.182	.....
CYBA	16	0.15185	0.47952	0.25475	0.18781	0.38961	14669	0.02973	0.182	.....
NUP107	12	0.26074	0.83916	0.07792	0.30370	0.24975	14669	0.02974	0.182	.....
TRIM49B	11	0.52847	0.18781	0.66933	0.48152	0.10589	14669	0.02975	0.182	.....
ARMC12	6	0.73526	0.12687	0.46054	0.02498	0.57343	14669	0.02976	0.182	.....
MLEC	12	0.82817	0.61838	0.21479	0.56543	0.05794	14669	0.02976	0.182	.....
TRH	3	0.30170	0.24076	0.37463	0.62138	0.13786	14669	0.02978	0.182	.....
MYO5A	15	0.71129	0.12188	0.07892	0.06494	0.64036	14669	0.02983	0.183	.....
GRK1	13	0.35465	0.57542	0.09291	0.31169	0.25175	14669	0.02985	0.183	.....
THG1L	5	0.65035	0.18482	0.17682	0.85215	0.08492	14669	0.02986	0.183	.....
LOC101927572	19	0.79720	0.00500	0.71828	0.01898	0.83916	14669	0.02987	0.183	.....
CYP1B1	2	0.46254	0.29271	0.31069	0.47153	0.14386	14669	0.02987	0.183	.....

TRAK1	3	0.23177	0.21578	0.88312	0.92907	0.05794	14669	0.02987	0.183	.....
KDF1	1	0.66134	0.27073	0.89211	0.09790	0.21279	14669	0.02988	0.183	.....
TUG1	22	0.48452	0.24575	0.29171	0.74825	0.09191	14669	0.02988	0.183	.....
LINC01364	1	0.36064	0.10889	0.73826	0.59640	0.12388	14669	0.02989	0.183	.....
ATPAF2	17	0.38262	0.51449	0.34466	0.05594	0.43057	14669	0.02991	0.183	.....
TDO2	4	0.47852	0.58242	0.37662	0.42657	0.10090	14669	0.02992	0.183	.....
LINC01047	13	0.29570	0.28671	0.07393	0.80619	0.16683	14669	0.02992	0.183	.....
GPRC5A	12	0.19580	0.10989	0.97403	0.18482	NA	6657	0.02993	0.183	....?
AGGF1	5	0.44356	0.93307	0.25774	0.20979	0.16184	14669	0.02993	0.183	.....
TRPM6	9	0.14685	0.84715	0.62138	0.13087	0.26573	14669	0.02993	0.183	.....
LYST	1	0.59640	0.81019	0.33267	0.03896	0.33267	14669	0.02994	0.183	.....
RTN4RL1	17	0.12887	0.35165	0.20480	0.95604	0.12787	14669	0.02994	0.183	.....
MTR	1	0.04196	0.37762	0.16284	0.59341	0.33267	14669	0.02995	0.183	.....
PSG8	20	NA	NA	0.89011	0.01598	0.28472	12267	0.03	0.183	??...
CCNB1	5	0.08492	0.06793	0.20879	0.66733	0.37762	14669	0.03001	0.183	.....
CR1L	1	0.68432	0.14486	0.27473	0.13087	0.36064	14669	0.03004	0.183	.....
MAP3K8	10	0.57642	0.16384	0.53946	NA	0.09890	11265	0.03007	0.183	...?.
SLC46A3	13	0.84416	0.69331	0.62537	0.05994	0.18382	14669	0.03007	0.183	.....
DNASE1	16	0.38062	0.44655	0.38262	0.29071	0.18482	14669	0.03008	0.183	.....
TRIM3	11	0.63736	0.00630	0.02198	NA	0.50150	11265	0.03009	0.183	...?.
SAMD15	14	0.09590	0.50450	0.48252	0.09491	0.49550	14669	0.03012	0.183	.....
SS18	18	0.22877	0.81618	0.82617	0.68132	0.04496	14669	0.03019	0.184	.....
SMIM24	19	0.28472	NA	0.40360	0.87213	0.04496	13691	0.03021	0.184	.?...
OR5R1	11	0.64735	0.04096	0.29371	0.74226	0.14186	14669	0.03022	0.184	.....
DLGAP2	8	0.30969	0.42857	0.09291	NA	0.18781	11265	0.03023	0.184	...?.
SUPT16H	14	0.27872	0.39860	0.74426	0.54545	0.09291	14669	0.03023	0.184	.....
CR2	1	0.71129	0.39760	0.14785	0.62537	0.09091	14669	0.03024	0.184	.....
LCP2	5	0.12687	0.21179	0.61139	0.33367	0.29071	14669	0.03024	0.184	.....
CEACAM21	19	0.31269	0.15584	0.46853	0.08092	0.52448	14669	0.03025	0.184	.....
LOC645949	2	0.83816	0.47353	0.32368	0.41259	0.08192	14669	0.03025	0.184	.....
ZBTB49	4	0.69930	0.38661	0.13786	0.00660	0.83217	14669	0.03028	0.184	.....
LOC643711	12	0.46154	0.88511	0.53247	0.69231	0.03497	14669	0.0303	0.184	.....
RHOBTB1	10	0.04296	0.31868	0.74925	NA	0.20679	11265	0.03031	0.184	...?.
TAS2R1	5	0.60539	0.29071	0.34366	0.70330	0.07393	14669	0.03031	0.184	.....
OSBPL1A	18	0.31469	0.90509	0.04396	0.09491	0.47153	14669	0.03032	0.184	.....
PCDHB18P	5	0.76723	0.92707	0.75325	0.57742	0.02098	14669	0.03032	0.184	.....
NDST1	5	0.12787	0.51948	0.81019	0.46653	0.13087	14669	0.03033	0.184	.....
HTR3B	11	0.24775	0.13287	0.40659	0.78821	0.14086	14669	0.03033	0.184	.....
GATA5	20	0.38761	0.46354	0.24376	0.17582	0.29471	14669	0.03033	0.184	.....
ADGRE1	19	0.36663	0.01898	0.82018	NA	0.19580	11265	0.03034	0.184	...?.
CUX2	12	0.17283	0.63137	0.87912	0.17383	0.21079	14669	0.03037	0.184	.....
MBOAT1	6	0.91209	0.41459	0.64136	0.01040	0.43756	14669	0.03037	0.184	.....
DNAH1	3	0.27073	0.32867	0.65934	0.71828	0.08092	14669	0.03037	0.184	.....
C1orf43	1	0.08392	0.14785	0.96603	0.17982	0.41958	14669	0.03038	0.184	.....
LINC01125	2	0.98501	0.84715	0.67233	0.45255	0.02597	14669	0.03039	0.184	.....
ZGLP1	19	0.20480	0.23776	0.50549	0.21479	0.33866	14669	0.03039	0.184	.....
RAPGEF2	4	0.74126	0.37762	0.13586	0.10190	0.35265	14669	0.0304	0.184	.....
PPP1R14A	19	0.10889	0.84316	0.08891	0.79221	0.14486	14669	0.03041	0.184	.....
ZNF721	4	0.57343	0.45455	0.94605	0.04496	0.27073	14669	0.03041	0.184	.....
SMC2	9	0.32667	0.31868	0.18482	0.19580	0.37263	14669	0.03042	0.184	.....
BZW1	2	0.83017	NA	0.18482	0.58641	0.05095	13691	0.03045	0.184	.?...
DOPEY1	6	0.34865	0.46953	0.11389	0.53447	0.17383	14669	0.03047	0.184	.....
LINC00032	9	0.20280	0.55944	0.49650	NA	0.10889	11265	0.03048	0.184	...?.
DESI2	1	0.02697	0.93007	0.82817	0.06793	0.48152	14669	0.03049	0.184	.....
FNDC3A	13	0.25375	0.40460	0.34266	NA	0.13786	11265	0.0305	0.184	...?.
UGDH	4	0.47652	0.33966	0.87213	0.45754	0.07892	14669	0.0305	0.184	.....
FTSJ2	7	0.15884	0.19181	0.33067	NA	0.23077	11265	0.0305	0.184	...?.
MASTL	10	0.09690	0.10989	0.93307	NA	0.20080	11265	0.03052	0.184	...?.
OR4C15	11	0.63037	0.45854	0.27872	0.51249	0.09191	14669	0.03052	0.184	.....
RAB3GAP1	2	0.02697	0.79720	0.83516	0.47952	0.16883	14669	0.03053	0.184	.....
PHGDH	1	0.20579	0.53646	0.71928	0.55445	0.09391	14669	0.03053	0.184	.....
ZBTB9	6	0.54545	0.20480	0.54346	0.11489	0.31568	14669	0.03053	0.184	.....
PIK3R6	17	0.67932	0.46853	0.38262	NA	0.06394	11265	0.03055	0.184	...?.
CDKL2	4	0.64635	0.62737	0.23177	0.23876	0.15984	14669	0.03057	0.184	.....
NCOA1	2	0.99900	0.77123	0.48252	0.01450	0.31668	14669	0.0306	0.184	.....
TRIM13	13	0.85115	0.12587	0.05794	0.10889	0.51648	14669	0.03061	0.184	.....
ZNF730	19	0.94006	0.52547	0.35964	0.03097	0.33367	14669	0.03063	0.184	.....
PRPF40A	2	0.58641	0.40759	0.88212	0.01099	0.48352	14669	0.03063	0.184	.....
CDH2	18	0.23277	0.48951	0.35564	0.15584	NA	6657	0.03063	0.184	....?

NEBL	10	0.17682	0.51149	0.06494	0.38162	NA	6657	0.03063	0.184	....?
PAFAH2	1	0.93407	0.06294	0.02498	0.64735	0.22577	14669	0.03066	0.184	.....
RIC3	11	0.97902	0.55345	0.78721	0.07592	0.14386	14669	0.03067	0.184	.....
KC6	18	0.65035	0.18482	0.49950	0.27073	0.18282	14669	0.03067	0.184	.....
DLEC1	3	NA	0.18881	0.12987	0.59940	0.13786	13245	0.03067	0.184	?....
NPC2	14	0.95604	0.22478	0.18382	0.31269	0.16583	14669	0.03068	0.184	.....
IL18RAP	2	0.64336	0.35265	0.18082	0.06993	0.43157	14669	0.0307	0.184	.....
TMEM8B	9	0.14086	0.65435	0.48352	0.77123	0.08392	14669	0.03072	0.184	.....
OXR1	8	NA	0.24376	0.23277	0.15285	0.29371	13245	0.03073	0.184	?....
MYH10	17	0.34565	0.67932	0.03497	NA	0.18282	11265	0.03076	0.184	...?.
NDUFAF7	2	0.32268	0.34066	0.23976	0.18781	0.35165	14669	0.03076	0.184	.....
UNC13A	19	0.60040	0.12488	0.12987	0.08591	0.58042	14669	0.03077	0.184	.....
MAP2K6	17	0.05295	0.98901	0.06593	NA	0.25375	11265	0.03077	0.184	...?.
LOC100288748	8	0.54545	0.20380	0.78821	NA	0.07592	11265	0.03077	0.184	...?.
TSHZ3	19	0.15884	0.51548	0.16484	0.28571	0.34066	14669	0.03078	0.184	.....
TOPORS	9	0.32767	0.55544	0.87712	0.00690	0.62238	14669	0.03078	0.184	.....
KLHL28	14	0.91708	0.67532	0.07692	0.25874	0.15584	14669	0.03081	0.184	.....
LOC101928887	10	0.36563	0.30769	0.66533	0.49950	0.10889	14669	0.03081	0.184	.....
ZNF320	19	0.32368	0.59141	0.00160	0.60340	0.34166	14669	0.03083	0.184	.....
CARD8	19	0.44555	0.17582	0.13686	0.12288	0.52448	14669	0.03084	0.184	.....
EFR3B	2	0.65634	0.43956	0.87113	0.01320	0.42657	14669	0.03087	0.184	.....
ST7L	1	0.72627	0.74326	0.80420	0.33666	0.05095	14669	0.03087	0.184	.....
PARP12	7	0.63037	0.72028	0.03596	0.34466	0.19580	14669	0.03087	0.184	.....
FAM171B	2	0.88312	0.04895	0.84915	0.06993	0.35365	14669	0.03088	0.184	.....
BTBD3	20	0.45255	0.46354	0.45055	0.03896	0.44955	14669	0.03089	0.184	.....
NAGS	17	0.93506	NA	0.73327	0.17882	0.07193	13691	0.03091	0.184	.?...
FKBP9P1	7	0.20779	0.80420	0.94605	NA	0.05495	11265	0.03092	0.184	...?.
GTF2I	7	0.64136	0.84316	0.00360	NA	0.17882	11265	0.03094	0.184	...?.
ANKS1A	6	0.58342	0.00410	0.53147	0.88711	0.16484	14669	0.03095	0.184	.....
CEP85	1	0.06394	0.10789	0.77123	0.40160	0.34965	14669	0.03096	0.184	.....
HARS2	5	0.06094	0.22577	0.04096	0.92507	0.32767	14669	0.03096	0.184	.....
CYP24A1	20	0.51648	0.39361	0.38062	0.72428	0.06693	14669	0.03096	0.184	.....
HNRNPC	14	0.16284	0.33267	0.33267	0.61738	0.17682	14669	0.031	0.184	.....
ZNF638	2	0.00390	0.53646	0.59141	0.22977	0.59740	14669	0.03101	0.184	.....
EIF3H	8	0.08691	0.93207	0.27572	NA	0.14685	11265	0.03102	0.184	...?.
MGC34796	1	0.49750	0.07293	0.12887	0.25874	0.43856	14669	0.03103	0.184	.....
TRPC2	11	0.06294	0.09990	0.20579	0.13586	0.86114	14669	0.03103	0.184	.....
RNF180	5	0.08092	0.82917	0.53946	0.03497	0.59441	14669	0.03103	0.184	.....
CCDC67	11	0.84815	0.89810	0.62937	NA	0.02298	11265	0.03108	0.185	...?.
LOC100506272	4	0.84715	0.93606	0.11489	0.10090	0.22078	14669	0.03111	0.185	.....
HOOK3	8	0.56144	0.62038	0.49151	0.02897	0.39461	14669	0.03111	0.185	.....
KIAA1841	2	0.36663	0.60839	0.30569	0.35964	0.15385	14669	0.03112	0.185	.....
SLC35F6	2	0.29570	0.19980	0.21379	0.65235	0.18382	14669	0.03112	0.185	.....
BOD1L1	4	0.40759	0.57942	0.98302	0.13886	0.16084	14669	0.03112	0.185	.....
ZBTB37	1	0.45455	0.19980	0.28671	0.30969	0.25275	14669	0.03114	0.185	.....
MAVS	20	0.08791	0.98102	0.85015	0.09391	0.30070	14669	0.03115	0.185	.....
CAPN13	2	0.09890	0.49151	0.62737	0.10490	0.44456	14669	0.03117	0.185	.....
ANKRD52	12	0.35664	NA	0.40859	0.42657	0.10090	13691	0.03118	0.185	.?...
DND1	5	0.12687	0.01998	0.07892	0.82617	0.45754	14669	0.03121	0.185	.....
NOX3	6	0.46753	0.05894	0.91508	0.35964	0.18781	14669	0.03121	0.185	.....
PRNP	20	0.59940	NA	0.47153	0.05395	0.26973	13691	0.03122	0.185	.?...
ZNF837	19	0.80220	0.08891	0.75624	0.18182	0.21379	14669	0.03124	0.185	.....
TMEM98	17	0.07892	0.09191	0.73726	0.13686	0.61339	14669	0.03129	0.185	.....
HDAC3	5	0.98901	0.75225	0.45854	0.32268	0.05594	14669	0.03129	0.185	.....
PIK3C2B	1	0.30769	0.58242	0.31568	0.27772	0.20879	14669	0.0313	0.185	.....
ZNF571	19	0.95405	0.00750	0.69730	0.26174	0.27273	14669	0.0313	0.185	.....
C8orf48	8	0.25175	0.41658	0.01898	0.23676	0.54146	14669	0.03131	0.185	.....
ESR2	14	0.60539	0.06593	0.39560	NA	0.15684	11265	0.03132	0.185	...?.
C2orf54	20	NA	0.78022	0.00560	0.09091	0.50549	13245	0.03132	0.185	?....
C8orf22	8	0.35664	0.42058	0.93506	0.14685	0.20380	14669	0.03133	0.185	.....
C1orf64	1	0.88611	0.16983	0.37662	0.01798	0.58741	14669	0.03134	0.185	.....
TACO1	17	0.95604	0.18382	0.63137	0.11189	0.21778	14669	0.03135	0.185	.....
MSX1	4	0.10490	0.67632	0.50749	0.04595	0.57143	14669	0.03137	0.185	.....
ANG	14	0.50649	0.61538	0.34066	0.66034	0.06294	14669	0.03138	0.185	.....
COA6	1	0.24176	NA	0.04695	0.10290	0.54246	13691	0.03139	0.185	.?...
CWC22	2	0.20879	0.09690	0.18282	0.84815	0.21379	14669	0.03139	0.185	.....
LIMK2	22	0.42058	0.12288	0.97802	0.32468	0.16683	14669	0.03139	0.185	.....
LINC00312	3	0.53147	0.89411	0.11489	0.16883	0.23377	14669	0.03141	0.185	.....
EPB41L5	2	0.95504	NA	0.42557	0.11389	0.13387	13691	0.03141	0.185	.?...

EPDR1	7	0.78222	0.00370	0.88112	0.37662	0.24775	14669	0.03142	0.185	.....
LINC00683	18	0.06094	0.12687	0.60839	0.46354	0.34565	14669	0.03144	0.185	.....
ALDH6A1	14	0.17982	0.93307	0.42458	NA	0.08991	11265	0.03145	0.185	...?.
PPP2R5A	1	0.46653	0.76623	0.08991	0.75724	0.08092	14669	0.03148	0.185	.....
SMIM10L1	12	0.31169	0.24176	0.44256	0.63437	0.12987	14669	0.03148	0.185	.....
TBC1D24	16	NA	NA	0.13487	NA	0.07592	8863	0.03149	0.185	??.?.
ABCA4	1	0.02198	0.24875	NA	0.31968	0.41958	13818	0.0315	0.185	..?..
TFCP2	12	0.02098	0.38462	0.67532	0.44356	0.31868	14669	0.0315	0.185	.....
TRMT10C	3	0.88312	0.03996	0.19081	0.17383	0.41359	14669	0.0315	0.185	.....
PLXNA2	1	0.05395	0.11988	0.90010	0.02198	0.98402	14669	0.03151	0.185	.....
GPATCH3	1	0.58841	0.75924	0.62737	0.19081	0.11788	14669	0.03152	0.185	.....
LOC101929319	2	0.87413	0.36164	0.45854	0.08192	0.25075	14669	0.03153	0.185	.....
MGC57346-CRHI	17	0.17982	0.38761	0.95305	0.71029	0.07493	14669	0.03153	0.185	.....
CALY	10	0.29770	0.94905	0.21379	0.06793	0.39361	14669	0.03154	0.185	.....
TMEM82	1	0.47453	0.86913	0.67033	0.31968	0.07792	14669	0.03155	0.185	.....
MEFV	16	0.06494	0.41259	0.18781	0.16783	0.60539	14669	0.03155	0.185	.....
GPALPP1	13	0.19081	0.00999	0.53247	0.56643	0.37862	14669	0.03156	0.185	.....
ZSWIM7	17	0.78721	0.61139	0.19281	0.34066	0.11588	14669	0.03156	0.185	.....
C12orf60	12	0.21578	0.23377	0.62338	NA	0.14685	11265	0.0316	0.185	...?.
STAG3	7	0.12288	0.52847	0.31469	0.13786	0.45754	14669	0.0316	0.185	.....
KIDINS220	2	0.53447	0.02797	0.00290	0.50450	0.63636	14669	0.03161	0.185	.....
LOC101929634	14	0.19281	0.06893	0.03297	0.74625	0.41359	14669	0.03162	0.185	.....
IK	5	0.02498	0.54346	0.02997	0.99001	0.30070	14669	0.03163	0.185	.....
ING3	7	0.07692	0.38661	0.58741	NA	0.18581	11265	0.03164	0.185	...?.
ZNF573	19	0.93506	0.02897	0.79321	0.38761	0.14386	14669	0.03165	0.185	.....
HSPA12A	10	0.12787	0.65934	0.63137	0.44555	0.14486	14669	0.03166	0.185	.....
TOX3	16	0.70529	0.00680	0.47253	NA	0.22677	11265	0.03166	0.185	...?.
PGPEP1	19	0.54346	0.94206	0.38661	0.31269	0.09191	14669	0.03167	0.185	.....
NINJ2	12	0.07992	0.71429	0.14486	NA	0.22777	11265	0.03168	0.185	...?.
ALG8	11	0.14286	0.01698	0.97502	NA	0.27772	11265	0.03169	0.185	...?.
FAM120B	6	0.06593	0.40460	0.97303	0.08691	0.47552	14669	0.03169	0.185	.....
RIT2	18	0.05994	0.19381	0.10789	0.77423	NA	6657	0.03171	0.185	....?
LOC101927151	19	0.05195	0.96404	0.83616	0.96803	0.04795	14669	0.03171	0.185	.....
DPY19L4	8	0.08492	0.17383	0.10090	NA	0.41758	11265	0.03173	0.185	...?.
SNRPG	2	0.29471	0.44755	0.96404	0.04795	0.37063	14669	0.03173	0.185	.....
FAM105A	5	0.75624	0.28272	0.76424	0.03497	0.35365	14669	0.03174	0.185	.....
FGFBP1	4	0.39760	0.57343	0.52048	0.01020	0.63936	14669	0.03175	0.185	.....
ACPP	3	0.55045	0.13087	0.88911	0.72627	0.06394	14669	0.03175	0.185	.....
MXD3	5	0.23776	0.00310	0.99700	0.04595	0.94206	14669	0.03175	0.185	.....
HLA-DPB1	6	0.02198	0.62637	0.22577	NA	0.30969	11265	0.03175	0.185	...?.
KMT5B	11	0.57642	0.28571	0.15784	0.25175	0.27572	14669	0.03175	0.185	.....
IFIH1	2	0.87313	0.12288	0.31369	0.51948	0.12088	14669	0.03176	0.185	.....
OLFML2A	9	0.22877	0.73127	0.20579	0.63836	0.11888	14669	0.03177	0.185	.....
C1QA	1	0.15884	0.48651	0.14985	0.04695	0.76124	14669	0.03178	0.185	.....
MMP8	11	0.79720	0.01998	0.33566	0.88611	0.11489	14669	0.0318	0.185	.....
H3F3AP4	1	0.90609	0.23976	0.13686	0.04795	0.50250	14669	0.0318	0.185	.....
PABPC1P2	2	0.01399	0.58342	0.59540	0.39361	NA	6657	0.03181	0.185	....?
ZNF485	10	0.34266	0.39860	0.12288	NA	0.17782	11265	0.03183	0.185	...?.
B4GALT2	1	0.36963	0.32967	0.23477	0.28971	0.26773	14669	0.03183	0.185	.....
ST8SIA4	5	0.42957	0.41359	0.30569	0.56044	0.11588	14669	0.03184	0.185	.....
CD276	15	0.84615	0.01099	0.39560	0.01898	0.89810	14669	0.03184	0.185	.....
SLMO2-ATP5E	20	0.82418	0.45455	0.72028	0.05195	0.24376	14669	0.03185	0.185	.....
MEPCE	7	0.38262	0.69331	0.05095	0.25375	0.30869	14669	0.03186	0.185	.....
DDX24	14	0.47652	NA	0.06693	NA	0.12488	10287	0.03186	0.185	.?.?.
PSMD11	17	0.78322	0.00530	0.41958	0.43257	0.29870	14669	0.03186	0.185	.....
LENG8-AS1	19	0.80519	0.69431	0.55844	0.01798	0.35265	14669	0.03188	0.185	.....
CEACAM20	19	0.66733	0.31169	0.79520	0.08891	0.23976	14669	0.03188	0.185	.....
FLJ34503	6	0.60539	0.75724	0.02298	0.31968	0.23477	14669	0.03188	0.185	.....
LINC01330	3	0.05095	0.02298	0.75724	0.07193	0.99001	14669	0.03188	0.185	.....
RCAN2	6	0.15385	0.71429	0.00300	NA	0.39660	11265	0.03191	0.185	...?.
RD3	1	0.76324	0.30669	0.21978	0.10390	0.33766	14669	0.03193	0.185	.....
NOL3	16	0.70629	0.15784	0.34765	0.30969	0.19980	14669	0.03194	0.185	.....
LIMS1	2	0.15185	0.27473	0.31169	0.02198	0.92507	14669	0.03194	0.185	.....
ZNF382	19	0.34366	0.98402	0.46753	0.46853	0.07393	14669	0.03194	0.185	.....
DENND2A	7	0.34166	0.01130	0.83417	0.29371	0.38761	14669	0.03194	0.185	.....
RPS2	16	0.15385	0.17083	0.33367	NA	0.25075	11265	0.03196	0.185	...?.
HFE2	1	NA	NA	NA	0.03197	NA	3404	0.03197	0.185	???.?
C11orf49	11	0.27273	0.04695	0.86414	0.34665	0.28172	14669	0.03197	0.185	.....
LOC388942	2	0.68132	0.26673	0.36863	0.00899	0.73027	14669	0.03199	0.185	.....

UTS2	1	0.51249	0.02098	0.62737	0.02597	0.83417	14669	0.032	0.185	.....
ZMYM5	13	0.07493	0.29770	0.94306	0.95005	0.08492	14669	0.032	0.185	.....
SOWAHB	4	0.30969	0.09391	0.68631	0.83616	0.10490	14669	0.032	0.185	.....
SELPLG	12	0.12687	0.42258	0.43956	0.01998	0.83417	14669	0.032	0.185	.....
TTLL3	3	0.11588	0.32068	0.38761	0.67333	0.18182	14669	0.03201	0.185	.....
C6orf15	6	0.51848	0.77622	0.93606	NA	0.03197	11265	0.03202	0.185	...?.
TPM3	1	0.50150	0.23576	0.32068	0.36364	0.19780	14669	0.03202	0.185	.....
RAPGEF4-AS1	2	0.72128	0.36264	0.64935	0.09491	0.23177	14669	0.03203	0.185	.....
CPEB1	15	0.30669	0.82517	0.28472	NA	0.09191	11265	0.03204	0.185	...?.
ANP32E	1	0.40160	NA	0.00380	0.13387	0.59740	13691	0.03207	0.185	.?...
AXIN1	16	0.59241	0.48751	0.79121	0.10090	0.20180	14669	0.03208	0.185	.....
SPIRE2	16	0.88911	0.31169	0.43357	NA	0.06094	11265	0.03209	0.185	...?.
KLHL5	4	0.36464	0.62637	0.45355	0.43057	0.11189	14669	0.03209	0.185	.....
ZDHHC17	12	0.87113	0.11389	0.15485	0.50350	0.16783	14669	0.03212	0.185	.....
GLE1	9	0.84116	0.94605	0.11688	0.74126	0.03896	14669	0.03216	0.185	.....
IL1RN	2	0.71229	0.36863	0.41359	0.09291	0.28571	14669	0.03216	0.185	.....
TAS2R8	12	0.44655	NA	0.22278	0.17383	0.23177	13691	0.03216	0.185	.?...
PPL	16	0.57842	0.50150	0.86513	0.05295	0.26573	14669	0.03216	0.185	.....
CYP20A1	2	0.58042	0.72827	0.30370	0.16983	0.18881	14669	0.03218	0.185	.....
TXNL4B	16	0.93506	0.17183	0.47153	0.01399	0.58242	14669	0.0322	0.185	.....
SMAD5	5	0.17183	0.14386	0.07093	0.12088	0.83217	14669	0.0322	0.185	.....
TOPORS-AS1	9	NA	0.33367	NA	0.05594	NA	4382	0.03224	0.185	?.?.?
IFRD2	3	0.58342	0.62537	0.77622	0.29670	0.08492	14669	0.03225	0.185	.....
GLTP	12	0.24276	0.38262	0.50649	0.61039	0.12088	14669	0.03226	0.185	.....
TAF11	6	0.43856	0.02797	0.50549	0.77822	0.16084	14669	0.03226	0.185	.....
CLTA	9	0.23876	0.75225	0.87013	0.95205	0.02797	14669	0.03227	0.185	.....
ARL10	5	0.67532	0.20280	0.54346	0.17183	0.23576	14669	0.03234	0.186	.....
FAM184A	6	0.44356	0.59540	0.08891	0.98302	0.06893	14669	0.03235	0.186	.....
AGTR1	3	0.70729	0.23576	0.41359	0.41259	0.12787	14669	0.03236	0.186	.....
LLPH	12	0.23776	0.23876	0.39161	0.51449	0.19980	14669	0.03236	0.186	.....
GALK2	15	0.46154	0.48651	0.04595	0.39061	0.25774	14669	0.03237	0.186	.....
SYNE3	14	0.99800	0.53047	0.05694	NA	0.08991	11265	0.03242	0.186	...?.
TMEM215	9	0.16184	0.59640	0.28871	0.06294	0.57443	14669	0.03244	0.186	.....
44450	20	NA	NA	NA	NA	NA	14669	0.03244	0.186	.....
LOC349160	7	NA	0.89910	0.56543	0.23576	0.08092	13245	0.03246	0.186	?....
ACTN4	19	0.73127	0.04895	0.15684	0.34565	0.32468	14669	0.03247	0.186	.....
FAM21EP	10	0.54545	NA	0.91409	NA	0.03297	10287	0.03252	0.186	.?.?.
WTH3DI	2	0.69331	NA	0.44955	0.20280	0.12088	13691	0.03257	0.186	.?...
YPEL4	11	0.43956	0.60739	0.66334	0.26274	0.12987	14669	0.03258	0.186	.....
VAV2	9	0.02498	0.07393	0.44755	NA	0.47353	11265	0.03261	0.186	...?.
LOC146880	17	0.07393	NA	0.35165	0.25175	0.32268	13691	0.03263	0.186	.?...
HRH4	18	0.43556	0.87113	0.50849	0.55345	0.05694	14669	0.03264	0.186	.....
SLC44A4	6	0.44655	0.41858	0.51349	0.01360	0.64136	14669	0.03265	0.186	.....
TDG	12	0.84715	0.01698	0.51449	0.03397	0.70330	14669	0.03265	0.186	.....
KLK1	19	0.19780	0.80719	0.99900	0.10190	0.23177	14669	0.03266	0.186	.....
LRRC61	7	0.68032	0.37163	0.14685	0.09790	0.39361	14669	0.03266	0.186	.....
LINC00411	13	0.65634	0.82318	0.77023	0.20380	0.08891	14669	0.03267	0.186	.....
LINC00161	21	0.67732	0.83017	0.44555	NA	0.04196	11265	0.03267	0.186	...?.
KIR3DX1	19	0.00240	0.47353	0.79121	0.30470	0.54545	14669	0.03269	0.186	.....
CREB3L1	11	0.05994	0.27972	0.44755	0.39061	0.36164	14669	0.03269	0.186	.....
KRT80	12	0.12288	0.47453	0.38262	0.06593	0.61239	14669	0.03269	0.186	.....
OVOL2	20	0.98701	0.50649	0.85215	0.06693	0.16284	14669	0.03272	0.186	.....
RASSF1	3	0.15584	1.00000	0.78122	0.44356	0.08891	14669	0.03273	0.186	.0...
APOBEC3B-AS1	20	NA	0.24575	0.06294	0.51648	0.18981	13245	0.03273	0.186	?....
MEGF8	19	0.11688	0.63836	0.93407	0.11788	0.30869	14669	0.03273	0.186	.....
DGUOK-AS1	2	0.87612	0.69830	0.99401	0.14186	0.08392	14669	0.03273	0.186	.....
POLR3A	10	0.26873	0.36264	0.34665	0.67233	0.12787	14669	0.03274	0.186	.....
SHISA5	3	0.65435	0.47053	0.81718	0.31868	0.08492	14669	0.03275	0.186	.....
KIF13A	6	0.09391	0.06793	0.70130	0.30270	0.45455	14669	0.03277	0.186	.....
LINC01567	16	0.08591	NA	0.14885	NA	0.22078	10287	0.03278	0.186	.?.?.
FPR2	19	0.90909	NA	0.15884	NA	0.05594	10287	0.03279	0.186	.?.?.
LOC400958	2	0.74525	0.26474	0.24975	0.16583	0.27872	14669	0.0328	0.186	.....
DARS-AS1	2	0.02498	0.11788	0.84316	0.64136	0.30370	14669	0.03281	0.186	.....
LINC01589	22	0.66633	NA	0.04795	0.36464	0.16983	13691	0.03282	0.186	.?...
LOC285626	5	0.71129	0.07792	0.03996	0.63437	0.25574	14669	0.03282	0.186	.....
KCNG2	18	0.11289	0.77922	0.28472	NA	0.15684	11265	0.03282	0.186	...?.
ZNF254	19	0.15984	0.82717	0.52348	0.13686	0.29770	14669	0.03283	0.186	.....
KLF3-AS1	4	0.03197	0.82018	0.46953	0.31868	0.30869	14669	0.03286	0.186	.....
SH3RF2	5	0.64935	0.46553	0.18981	0.64436	0.08691	14669	0.03289	0.186	.....

MYH13	17	0.27972	0.15984	0.64436	0.43656	0.19980	14669	0.0329	0.186	.....
MGC45922	19	0.28571	0.40759	0.44755	0.35964	0.19281	14669	0.03291	0.186	.....
OR5AR1	11	0.10390	0.10689	0.02198	0.37962	0.75025	14669	0.03291	0.186	.....
TDRD9	14	0.48951	0.60440	0.22278	0.54446	0.10490	14669	0.03291	0.186	.....
OR2H2	6	0.79520	0.98302	0.22378	0.80519	0.02697	14669	0.03293	0.186	.....
TRABD2A	2	0.49750	0.10689	0.72428	0.23277	0.25275	14669	0.03295	0.186	.....
NTHL1	16	0.40260	0.17083	0.56543	0.00790	0.89211	14669	0.03295	0.186	.....
FREM3	4	0.73626	0.68232	0.77423	0.01000	0.40759	14669	0.03296	0.186	.....
WBP1	2	0.48951	0.07792	0.11289	0.08092	0.74625	14669	0.03298	0.186	.....
HCG23	6	0.15085	0.31069	0.00510	0.91309	NA	6657	0.03298	0.186	....?
PLEKHA8P1	12	0.21279	0.49850	0.22278	0.57443	0.17383	14669	0.03299	0.186	.....
LOC105375396	7	0.30170	0.63836	0.72128	0.86014	0.03996	14669	0.033	0.186	.....
MAP4K1	19	0.36164	0.20080	0.43357	0.44955	0.19181	14669	0.033	0.186	.....
KIAA1958	9	0.44855	0.91409	0.72827	0.00113	0.77023	14669	0.03301	0.186	.....
BMP1	8	0.04895	0.40060	0.39161	0.73327	0.20779	14669	0.03301	0.186	.....
MRPL10	17	0.02498	0.92907	0.84016	0.48951	0.16384	14669	0.03301	0.186	.....
PTPRS	19	0.62238	0.59441	0.30470	NA	0.07193	11265	0.03303	0.186	...?.
LOC440300	15	0.62038	0.09590	0.02398	NA	0.31668	11265	0.03304	0.186	...?.
RAB39A	11	0.96104	0.85514	0.01698	NA	0.09590	11265	0.03305	0.186	...?.
AATK	17	0.01399	0.88012	0.66533	0.23976	0.37463	14669	0.03306	0.186	.....
LINC01588	14	0.62737	0.26573	0.94206	0.08492	0.25275	14669	0.03306	0.186	.....
MTHFD2	2	0.27273	0.33766	0.08092	0.53247	0.26673	14669	0.03307	0.186	.....
LOR	1	0.07093	0.79221	0.07493	0.71429	0.22577	14669	0.03307	0.186	.....
TMEM50B	21	0.04396	0.18581	0.36563	0.82917	0.25574	14669	0.03308	0.186	.....
ZDBF2	2	0.44855	0.10589	0.78322	0.03297	0.60340	14669	0.03308	0.186	.....
LINC01087	2	0.88511	0.98501	0.03696	0.11489	0.26773	14669	0.0331	0.186	.....
CIB3	19	0.30170	0.25075	0.51249	0.35065	0.22078	14669	0.03312	0.186	.....
ATP8B3	19	0.78621	0.02398	0.75425	0.26474	0.24975	14669	0.03312	0.186	.....
STK17B	2	0.57443	0.45355	0.94805	0.28072	0.09690	14669	0.03312	0.186	.....
DENND6A	3	0.48651	0.31369	0.51848	0.92907	0.04895	14669	0.03313	0.186	.....
ALKBH2	12	0.80519	0.73626	0.67133	0.16683	0.10490	14669	0.03314	0.186	.....
KTN1-AS1	14	0.70529	0.65534	0.45055	0.03397	0.34765	14669	0.03314	0.186	.....
PIAS2	18	0.53946	0.48951	0.56643	NA	0.06593	11265	0.03316	0.186	...?.
NVL	1	0.65435	0.16683	0.10889	0.06893	0.61538	14669	0.03316	0.186	.....
LRRC74A	14	0.16084	0.34366	0.64635	NA	0.14585	11265	0.03316	0.186	...?.
URAD	13	0.28372	0.83017	0.70729	0.11988	0.22278	14669	0.03317	0.186	.....
STC1	8	0.49351	0.46054	0.99301	0.88911	0.02498	14669	0.03318	0.186	.....
TMEM45B	11	0.41459	0.61139	0.48252	NA	0.07592	11265	0.0332	0.187	...?.
LINC00836	10	0.32667	0.09491	0.66933	NA	0.16484	11265	0.03321	0.187	...?.
C3orf22	3	0.33766	0.80519	0.73826	0.81918	0.03297	14669	0.03325	0.187	.....
CPNE1	20	0.59141	0.56144	0.47453	0.36763	0.10290	14669	0.03327	0.187	.....
RARRES2	7	0.88112	0.16084	0.29171	0.10390	0.35564	14669	0.03328	0.187	.....
LRRC23	12	0.10290	NA	0.50649	0.14386	0.35465	13691	0.0333	0.187	.?...
PRSS46	3	0.52048	0.87512	0.53646	0.01698	0.42557	14669	0.0333	0.187	.....
TXNDC12	1	0.74026	0.36663	0.22178	0.47752	0.11788	14669	0.0333	0.187	.....
GCSAM	3	0.15984	0.40659	0.67133	0.38362	0.19680	14669	0.03333	0.187	.....
BCAS3	17	0.77922	0.16084	0.09191	0.01698	0.86813	14669	0.03334	0.187	.....
GBP1	1	0.72727	0.22478	0.10689	0.27872	0.28172	14669	0.03335	0.187	.....
GTPBP1	22	0.01798	0.44855	0.45255	0.19780	0.59441	14669	0.03336	0.187	.....
PHKG2	16	0.06494	NA	0.09790	0.25175	0.47353	13691	0.03336	0.187	.?...
PLEKHS1	10	0.42058	0.99301	0.58541	0.17982	0.13786	14669	0.03339	0.187	.....
BAG3	10	0.20579	0.87213	0.00410	0.41159	0.41359	14669	0.03343	0.187	.....
LINC01081	16	0.04196	0.96503	0.37363	0.18781	0.39161	14669	0.03343	0.187	.....
KLF3	4	0.01798	0.94106	0.47952	0.34266	0.31469	14669	0.03344	0.187	.....
ZNF713	7	0.29371	0.76723	0.45854	NA	0.08292	11265	0.03345	0.187	...?.
USP19	3	0.21878	0.42657	0.62238	0.30270	0.21179	14669	0.03347	0.187	.....
LOC101927762	10	0.87912	0.80020	0.62737	0.10789	0.12887	14669	0.03347	0.187	.....
AKR7A2	1	0.97103	0.10290	0.71329	0.05395	0.35964	14669	0.03347	0.187	.....
CST9	20	0.54346	0.02697	0.44655	0.17383	0.47552	14669	0.03348	0.187	.....
ERICH4	19	0.21179	NA	0.88312	0.10989	0.24476	13691	0.03348	0.187	.?...
LIG4	13	0.56743	0.68432	0.17283	0.93407	0.04595	14669	0.03348	0.187	.....
CDPF1	22	0.54845	0.16084	0.09690	0.13187	0.54146	14669	0.0335	0.187	.....
CDT1	16	0.19381	0.61339	0.79520	NA	0.08691	11265	0.03351	0.187	...?.
PVRL3-AS1	3	0.57842	NA	0.35265	0.51648	0.06893	13691	0.03352	0.187	.?...
PDCD5	19	0.76923	0.35564	0.77522	0.37363	0.07892	14669	0.03352	0.187	.....
LINC01005	7	0.35964	0.57842	0.20879	NA	0.12587	11265	0.03353	0.187	...?.
GPR160	3	0.27772	0.99800	0.37562	0.12787	0.25574	14669	0.03354	0.187	.....
TMEM147	19	0.59441	0.82817	0.24875	0.21379	0.16484	14669	0.03357	0.187	.....
LINC00314	21	0.36763	0.49650	0.31968	0.19081	0.27972	14669	0.03359	0.187	.....

ERI1	8	0.14186	0.46553	0.71229	0.08492	0.44855	14669	0.03359	0.187	.....
PKI55	20	NA	0.89610	0.10190	0.08991	0.29471	13245	0.0336	0.187	?....
INAFM1	19	0.90509	NA	0.01598	0.41059	0.15984	13691	0.03361	0.187	.?...
CENPW	6	0.02897	0.11988	0.35864	0.33666	0.62737	14669	0.03361	0.187	.....
LOC401052	3	0.03497	NA	0.48352	0.14585	0.47652	13691	0.03362	0.187	.?...
KPNA5	6	0.33766	0.05894	0.34665	NA	0.25075	11265	0.03362	0.187	...?.
LOC100507557	6	0.14585	0.72228	0.07792	0.33866	0.34765	14669	0.03363	0.187	.....
GOSR1	17	0.13187	0.95504	0.90210	0.10789	0.25774	14669	0.03363	0.187	.....
ITGA11	15	0.21778	0.60240	0.60440	0.09990	0.35165	14669	0.03365	0.187	.....
SCGB2A1	11	0.95305	0.06394	0.46953	0.67832	0.08991	14669	0.03367	0.187	.....
LOC101929574	10	0.28372	NA	0.00990	0.32667	0.39860	13691	0.0337	0.187	.?...
SUPT7L	2	0.54545	0.33566	0.53646	0.43157	0.11688	14669	0.03371	0.187	.....
GJD2	15	0.10589	NA	0.06993	0.06693	0.75724	13691	0.03373	0.187	.?...
SERPINI1	3	0.37662	0.94505	0.10889	0.12188	0.34466	14669	0.03375	0.187	.....
HLA-E	6	0.56743	0.24675	0.43157	0.99800	0.04895	14669	0.03375	0.187	.....
RGS16	1	0.05095	0.93606	0.67732	0.27073	0.24276	14669	0.03376	0.187	.....
SLC35G6	17	0.87213	0.01099	0.63337	0.11788	0.45854	14669	0.03377	0.187	.....
C1orf168	1	0.44056	0.42158	0.16783	0.71229	0.11489	14669	0.03377	0.187	.....
MBD2	18	0.30569	0.09091	0.17682	0.20180	0.56344	14669	0.03377	0.187	.....
CPT1C	19	0.54446	0.89111	0.46753	0.41958	0.07093	14669	0.03378	0.187	.....
GPATCH8	17	0.46753	0.33067	0.38961	0.27572	0.21678	14669	0.03379	0.187	.....
HYLS1	11	0.37463	0.87413	0.36963	0.16683	0.21179	14669	0.0338	0.187	.....
C9orf66	9	0.21878	0.10290	0.20979	0.38262	0.42058	14669	0.03382	0.187	.....
GRM1	6	0.03297	0.68531	0.00960	0.24476	0.81918	14669	0.03383	0.187	.....
ANKRD60	20	0.14286	0.31668	0.48751	0.41059	0.25375	14669	0.03383	0.187	.....
ZNF816-ZNF321F	19	0.34166	NA	0.63137	0.18082	0.17882	13691	0.03384	0.187	.?...
PDE6H	12	0.37762	0.77522	0.55644	NA	0.06394	11265	0.03385	0.187	...?.
MAFF	22	0.06094	0.60240	0.31269	0.14486	0.53546	14669	0.03385	0.187	.....
CNNM1	10	0.10889	0.46953	0.50050	0.80220	0.11888	14669	0.03386	0.187	.....
NCS1	9	0.31668	0.48152	0.70230	0.12687	0.27872	14669	0.03386	0.187	.....
POLR3C	1	0.63836	0.95804	0.65335	0.04895	0.22078	14669	0.03387	0.187	.....
TRERF1	6	0.87712	0.36963	0.08791	NA	0.11389	11265	0.03387	0.187	...?.
LOC101929551	2	0.82717	0.06893	0.23477	0.18581	0.37163	14669	0.03388	0.187	.....
SLC26A1	4	0.19481	0.64336	0.16284	0.36364	0.26474	14669	0.03388	0.187	.....
L1TD1	1	0.75225	0.37562	0.01499	0.63237	0.18581	14669	0.03389	0.187	.....
SYNC	1	0.10090	0.62338	0.24076	0.96803	0.10989	14669	0.0339	0.187	.....
SEMA4G	10	0.42158	0.38062	0.09491	0.60240	0.17982	14669	0.0339	0.187	.....
CLEC3B	3	0.81119	0.75025	0.69231	0.08392	0.16084	14669	0.03393	0.187	.....
ACVR1B	12	0.64835	0.66533	0.63237	0.12587	0.16983	14669	0.03394	0.187	.....
LINC00430	13	0.07493	0.27572	0.88911	0.06194	0.63437	14669	0.03395	0.187	.....
LIAS	4	0.38961	0.71429	0.69431	0.04296	0.35165	14669	0.03395	0.187	.....
ZMIZ1-AS1	10	0.34466	0.89910	0.14286	NA	0.11089	11265	0.03396	0.187	...?.
QTRT1	19	0.38861	0.18182	0.93107	0.24376	0.21079	14669	0.03396	0.187	.....
THUMPD3-AS1	3	0.94106	0.02498	0.57243	0.01299	0.80120	14669	0.03396	0.187	.....
CHRM4	11	NA	0.03197	0.66034	0.17083	0.34665	13245	0.03397	0.187	?....
FAM183CP	8	0.52248	0.54845	0.57942	0.12188	0.22977	14669	0.03397	0.187	.....
LINC00641	14	0.35864	NA	0.18082	0.10989	0.36464	13691	0.03398	0.187	.?...
DYNC1I2	2	0.34665	0.83217	0.17483	0.12687	0.33666	14669	0.03399	0.187	.....
KIAA1257	3	0.45754	0.72827	0.09790	0.37063	0.18981	14669	0.034	0.187	.....
EFNB2	13	0.95305	0.78921	0.17283	0.53746	0.05694	14669	0.03403	0.187	.....
ZFPM2	8	NA	0.41958	0.71728	0.04396	NA	5233	0.03403	0.187	?...?
CRHBP	5	0.92408	0.57842	0.30370	0.35564	0.08991	14669	0.03407	0.187	.....
ZNF345	19	0.56543	0.00940	0.30470	0.50150	0.34466	14669	0.03408	0.187	.....
PDZK1	1	0.94306	0.56444	0.26873	0.19281	0.15385	14669	0.03411	0.187	.....
AKAP10	17	0.44456	0.19880	0.60539	0.01199	0.77223	14669	0.03412	0.187	.....
CLSTN2	3	0.69331	0.01299	0.33267	NA	NA	3253	0.03413	0.187	...??
TUBB1	20	0.60340	0.55944	0.31868	0.03397	0.46154	14669	0.03414	0.187	.....
ZNF804A	2	0.47552	0.45854	0.21079	0.41958	0.16983	14669	0.03414	0.187	.....
SLK	10	0.00680	0.55744	0.50350	0.57143	0.35065	14669	0.03415	0.187	.....
LOC100506885	6	0.09790	0.33966	0.45455	0.17582	0.47852	14669	0.03416	0.187	.....
GID8	20	0.71928	0.14785	0.25674	0.97502	0.06893	14669	0.03416	0.187	.....
HNRNPA3P1	10	0.37662	0.80420	0.50649	0.11688	0.24276	14669	0.03417	0.187	.....
SFXN3	10	0.26374	0.32867	0.07093	0.50749	0.30170	14669	0.03418	0.187	.....
FBXL15	10	0.23377	0.09690	0.93806	0.81718	0.10789	14669	0.03418	0.187	.....
PRKAG2	7	0.18182	0.02897	0.45355	NA	0.34665	11265	0.03419	0.187	...?.
HES3	1	0.32468	NA	0.98402	NA	0.04795	10287	0.0342	0.187	.?.?.
NLRP14	11	0.00102	0.87113	0.86414	NA	0.31069	11265	0.0342	0.187	...?.
MOAP1	14	0.37163	0.14386	0.41858	NA	0.17383	11265	0.03421	0.187	...?.
CFHR5	1	0.67233	0.23277	0.24376	0.42158	0.17183	14669	0.03421	0.187	.....

KCTD1	18	0.77822	0.25375	0.68831	0.73427	0.04595	14669	0.03421	0.187	.....
TTC34	1	0.23676	0.77423	0.87812	0.24476	0.14486	14669	0.03422	0.187	.....
GSTO1	10	0.01998	0.77423	0.03397	0.35365	0.62537	14669	0.03423	0.187	.....
AMER3	20	NA	0.17083	0.35265	0.57642	0.11489	13245	0.03425	0.187	?....
MTMR10	15	0.03896	0.57043	0.57542	0.73826	0.16284	14669	0.03426	0.187	.....
CLEC19A	16	0.76424	0.32667	0.22677	0.37762	0.15385	14669	0.03428	0.187	.....
SOST	17	0.64535	0.56244	0.30470	0.06593	NA	6657	0.03428	0.187	....?
LOC105377682	5	0.27473	0.33966	0.65834	0.11988	0.36164	14669	0.03428	0.187	.....
BTNL3	5	0.92308	0.30170	0.00340	0.86314	0.16284	14669	0.03429	0.187	.....
SLC44A5	1	NA	0.38362	0.32867	NA	0.08492	9841	0.03431	0.187	?..?.
LOC101929106	3	0.13387	0.75524	0.68931	0.02997	0.56344	14669	0.03431	0.187	.....
POU4F1	13	0.05495	0.74525	0.77023	NA	0.13586	11265	0.03433	0.187	...?.
HARBI1	11	0.12687	0.30470	0.33966	NA	0.23177	11265	0.03434	0.187	...?.
COPS7B	20	NA	0.29770	0.22378	0.02897	0.59141	13245	0.03435	0.187	?....
GCM2	6	0.11489	0.86314	0.24376	0.23576	0.32468	14669	0.03439	0.187	.....
SEMA7A	15	0.33766	0.29870	0.07493	NA	0.24575	11265	0.03439	0.187	...?.
KRTAP9-4	17	0.22977	0.35165	0.23976	0.12088	0.52448	14669	0.0344	0.187	.....
CLDN23	8	0.51648	0.18781	0.91608	0.18082	0.22178	14669	0.03441	0.187	.....
PDZK1IP1	1	0.77023	0.68831	0.63836	0.50849	0.04396	14669	0.03442	0.187	.....
HMGCS2	1	0.20180	0.19780	0.31968	0.64436	0.21179	14669	0.03442	0.187	.....
TRIM16L	17	0.04595	0.94905	0.70729	0.43457	0.17183	14669	0.03443	0.187	.....
DEFB129	20	0.46953	NA	0.35365	0.06593	0.33766	13691	0.03444	0.187	.?...
PDYN	20	0.28971	0.66034	0.52847	0.86314	0.05195	14669	0.03447	0.187	.....
LOC101928766	17	0.08591	0.41958	0.97902	0.13686	0.38062	14669	0.03448	0.187	.....
LOC101927583	12	0.33367	0.61638	0.31668	0.31668	0.19381	14669	0.03448	0.187	.....
PBX4	19	0.75924	0.18182	0.26773	0.09091	0.42258	14669	0.03449	0.187	.....
FSTL4	5	0.84216	0.97103	0.04695	NA	0.07792	11265	0.03449	0.187	...?.
DDX56	7	0.44555	0.66633	0.12687	0.19580	0.29471	14669	0.03449	0.187	.....
PI3	20	0.61139	0.32168	0.88412	0.11289	0.22278	14669	0.03451	0.188	.....
SMAP2	1	0.08791	0.00540	0.67832	0.16184	0.90310	14669	0.03453	0.188	.....
NR5A2	1	0.14985	0.72428	0.23277	0.17582	0.39061	14669	0.03454	0.188	.....
ACTL8	1	0.87512	0.06993	0.68531	0.33966	0.15984	14669	0.03457	0.188	.....
RXRG	1	0.28771	0.51748	0.92408	0.24875	0.16084	14669	0.03458	0.188	.....
VCAM1	1	0.51249	0.77622	0.86913	0.39361	0.06094	14669	0.0346	0.188	.....
CENPO	2	NA	0.13487	0.67233	0.44256	0.11988	13245	0.03465	0.188	?....
LINC00552	13	0.25874	0.71928	0.18282	NA	0.14186	11265	0.03465	0.188	...?.
PHF14	7	0.65435	0.64835	0.75125	0.95005	0.01798	14669	0.03466	0.188	.....
POLR1A	2	0.11389	0.14086	0.75325	0.46354	0.27273	14669	0.03467	0.188	.....
RAD23A	19	0.84815	0.29970	0.55944	0.25574	0.13786	14669	0.03468	0.188	.....
FAM43A	3	0.63137	0.30270	0.91109	0.04196	0.35065	14669	0.03468	0.188	.....
RMI2	16	0.83417	0.79720	0.35165	0.14785	0.15185	14669	0.03468	0.188	.....
LINC01337	5	0.71229	0.35265	0.55844	0.93407	0.03297	14669	0.03471	0.188	.....
LAMA5	20	0.18182	0.25075	0.67532	0.07193	0.55944	14669	0.03471	0.188	.....
ADGRF3	2	0.29770	0.95405	0.13786	0.01280	0.77023	14669	0.03471	0.188	.....
GABRB1	4	0.46653	0.55544	0.88412	0.30370	0.10390	14669	0.03472	0.188	.....
C6orf223	6	0.24975	0.23976	0.36264	NA	0.18981	11265	0.03472	0.188	...?.
LOC101928132	11	0.59141	0.93506	0.18681	NA	0.06893	11265	0.03472	0.188	...?.
LILRB5	19	0.42857	NA	0.12987	0.44356	0.15185	13691	0.03475	0.188	.?...
ZBED3	5	0.89411	0.27473	0.74126	0.12488	0.18981	14669	0.03475	0.188	.....
HBP1	7	0.97602	0.41259	0.48452	0.49151	0.05994	14669	0.03477	0.188	.....
RNF2	1	0.24875	0.19580	0.88412	0.01660	0.75724	14669	0.03478	0.188	.....
CTLA4	2	0.07792	0.72328	0.68931	0.14885	0.35864	14669	0.03479	0.188	.....
COPZ2	17	0.02797	0.16184	0.73526	0.28272	0.52847	14669	0.0348	0.188	.....
APOA1BP	1	0.27972	0.79121	0.06693	0.76823	0.12887	14669	0.0348	0.188	.....
NIPSNAP1	22	0.00920	0.13387	0.49351	0.30969	0.72727	14669	0.03481	0.188	.....
CACNA2D2	3	0.13686	0.79720	0.95704	0.39960	0.11588	14669	0.03481	0.188	.....
POLR1E	9	0.66533	0.53946	0.28472	0.43257	0.10989	14669	0.03482	0.188	.....
REEP3	10	0.42258	0.90310	0.29371	0.74326	0.05495	14669	0.03482	0.188	.....
BCLAF1	6	0.07193	0.12687	0.24675	0.20480	0.71928	14669	0.03483	0.188	.....
DNAH2	17	0.48252	0.89111	0.22078	0.89211	0.04296	14669	0.03483	0.188	.....
SESTD1	2	0.23177	0.95904	0.59640	0.01998	0.51948	14669	0.03483	0.188	.....
GIT2	12	0.41758	0.63736	0.20779	0.44456	0.14985	14669	0.03484	0.188	.....
FBXW11	5	0.27373	0.17183	0.06693	0.15285	0.69830	14669	0.03484	0.188	.....
THYN1	11	0.84016	0.84416	0.90909	0.03397	0.19980	14669	0.03485	0.188	.....
BRCAT107	5	0.00580	0.29471	0.13387	NA	0.62837	11265	0.03487	0.188	...?.
PCDHB13	5	0.83916	0.22278	0.41459	0.54246	0.09291	14669	0.03488	0.188	.....
LINC00885	3	0.18981	0.21279	0.91508	0.09990	0.44755	14669	0.03488	0.188	.....
MPRIP	17	0.10290	0.44955	0.88611	0.08092	0.47253	14669	0.03489	0.188	.....
PRR14	16	NA	NA	0.33467	0.27572	0.11289	12267	0.0349	0.188	??...

LINC00504	4	0.13786	0.15385	0.33167	NA	0.28871	11265	0.03492	0.188	...?.
ZNF70	22	0.12288	NA	0.36763	0.60539	0.13986	13691	0.03493	0.188	.?...
FLJ46066	3	0.39361	NA	0.06094	0.05894	0.57842	13691	0.03493	0.188	.?...
OR10K2	1	0.58342	0.64835	0.15285	0.02298	0.60639	14669	0.03494	0.188	.....
TPMT	6	0.26773	0.49451	0.08591	0.39261	0.30270	14669	0.03495	0.188	.....
LOC102546294	5	0.14286	0.59840	0.38362	0.20480	0.34665	14669	0.03497	0.188	.....
LOC101927571	18	0.73427	0.86513	0.32168	0.66334	0.04096	14669	0.03498	0.188	.....
STAMBP	2	0.92408	0.38062	0.91908	0.01199	0.40360	14669	0.03502	0.188	.....
OXCT1	5	0.26074	0.68831	0.11588	NA	0.16983	11265	0.03504	0.188	...?.
CCNE2	8	0.18382	0.52248	0.51548	0.63936	0.12088	14669	0.03504	0.188	.....
LINC00337	1	0.69231	0.38262	0.39660	NA	0.07992	11265	0.03505	0.188	...?.
FST	5	NA	0.96803	0.03397	NA	0.10290	9841	0.03505	0.188	?..?.
LOC101927876	1	0.34665	0.28771	0.25574	0.80819	0.11988	14669	0.03505	0.188	.....
LOC101929541	1	0.45654	0.30470	0.24775	0.96603	0.07592	14669	0.03507	0.188	.....
CCAT1	8	0.49151	0.99101	0.02098	0.99301	0.07193	14669	0.03508	0.188	.....
NECAB2	16	0.18981	0.79520	0.25175	0.76623	0.10090	14669	0.0351	0.188	.....
SPACA6P-AS	19	0.23377	0.14985	0.65435	NA	0.17782	11265	0.03511	0.188	...?.
PALM2	9	0.27572	0.51149	0.21878	0.02498	0.76424	14669	0.03513	0.188	.....
PAIP1	5	0.44955	0.22078	0.17882	0.14885	0.46853	14669	0.03514	0.188	.....
METTL7A	12	0.01898	0.12887	0.27572	0.66234	0.48951	14669	0.03515	0.188	.....
SMAD1	4	0.17682	0.62937	0.43157	0.33866	0.21778	14669	0.03515	0.188	.....
DNAAF5	7	0.98202	0.96703	0.82118	0.71229	0.01199	14669	0.03515	0.188	.....
CPNE9	3	0.34266	0.01199	0.44755	0.22178	0.59740	14669	0.03518	0.188	.....
KRT5	12	0.89411	0.45155	0.05594	0.45355	0.15584	14669	0.03519	0.188	.....
PIGW	17	0.24875	0.37562	0.75524	0.34366	0.18182	14669	0.03519	0.188	.....
FLJ37201	10	0.79421	0.51249	0.22877	0.13586	0.25175	14669	0.03523	0.188	.....
LEMD1	1	0.16284	0.76024	0.95704	0.40160	0.11189	14669	0.03524	0.188	.....
TRAF6	11	0.35764	0.51449	0.44855	0.01898	0.64735	14669	0.03524	0.188	.....
PSMB6	17	0.87612	NA	0.40160	0.24176	0.09890	13691	0.03525	0.188	.?...
ZNF860	3	0.61239	0.81618	0.53646	0.14486	0.16084	14669	0.03525	0.188	.....
DEF6	6	0.07892	0.20679	0.21678	0.67233	0.32268	14669	0.03525	0.188	.....
PLA2G5	1	0.50649	0.58941	0.28671	0.46753	0.11888	14669	0.03525	0.188	.....
LINC01532	19	0.11389	0.38462	0.60739	0.82118	0.11788	14669	0.03526	0.188	.....
YEATS4	12	0.54645	0.63636	0.06494	0.45255	0.18082	14669	0.03526	0.188	.....
CYB5R1	1	0.21578	0.38062	0.81319	0.88112	0.06494	14669	0.03529	0.188	.....
CFAP61	20	0.77822	0.80919	0.50350	0.29570	0.08092	14669	0.0353	0.188	.....
C4orf45	4	0.48152	0.49750	0.84016	0.03297	0.39061	14669	0.03531	0.188	.....
FBXO38	5	0.39860	0.92308	0.94505	0.27772	0.08392	14669	0.03531	0.188	.....
ROMO1	20	0.64835	0.85714	0.85514	0.94006	0.01299	14669	0.03532	0.188	.....
LOC441666	10	0.91409	0.80819	0.64735	0.92408	0.01220	14669	0.03532	0.188	.....
LDLRAD3	11	0.00300	0.21179	0.15584	0.87013	0.55445	14669	0.03533	0.188	.....
AAGAB	15	0.49351	0.69630	0.23876	0.01399	0.66234	14669	0.03533	0.188	.....
PARN	16	0.48751	0.48252	0.36164	NA	0.09690	11265	0.03534	0.188	...?.
CYP4F3	19	0.14486	0.59540	0.91508	0.18881	0.24775	14669	0.03535	0.188	.....
GALNT16	14	0.34066	0.03796	0.14985	NA	0.36863	11265	0.03536	0.188	...?.
VPS72	1	0.47353	0.66933	0.32567	0.02597	0.53247	14669	0.03536	0.188	.....
MIR924HG	18	0.48452	0.80020	0.42058	0.05095	NA	6657	0.03537	0.188	....?
LAMTOR2	1	0.55944	0.07792	0.93506	0.30370	0.19780	14669	0.03539	0.188	.....
ONECUT3	19	0.85614	0.01698	0.47253	0.88012	0.10889	14669	0.03543	0.188	.....
CTSA	20	0.41858	0.98701	0.16084	0.14785	0.27173	14669	0.03543	0.188	.....
HHIPL2	1	0.62737	0.27473	0.69031	0.11788	0.26673	14669	0.03544	0.188	.....
AMPD1	1	0.62737	0.02597	0.45654	0.37063	0.29970	14669	0.03545	0.188	.....
WBP1L	10	0.98501	0.76324	0.00650	0.93606	0.07293	14669	0.03546	0.188	.....
DSPP	4	0.31768	0.81718	0.92607	0.24476	0.11988	14669	0.03546	0.188	.....
IGFBP7-AS1	4	0.97502	0.83017	0.82218	0.26174	0.05095	14669	0.03551	0.188	.....
ZFP42	4	0.37862	NA	0.88412	0.58042	0.04895	13691	0.03554	0.189	.?...
LOC101927849	4	0.44256	0.02498	0.12488	0.93706	0.23576	14669	0.03555	0.189	.....
ZNF529	19	0.56444	0.94106	0.25674	0.66933	0.05395	14669	0.03556	0.189	.....
LOC100133077	9	0.05295	NA	0.57043	0.25275	0.31668	13691	0.03556	0.189	.?...
LOC101929512	2	0.02498	0.55145	0.34166	0.52747	0.33267	14669	0.03559	0.189	.....
EHMT2	6	0.50250	0.23576	0.46753	0.14286	0.33966	14669	0.03559	0.189	.....
RSAD1	17	0.47053	0.17982	0.53347	0.40859	0.18382	14669	0.0356	0.189	.....
ATOX1	5	0.90310	0.47153	0.85714	0.35165	0.06094	14669	0.03566	0.189	.....
TCF15	20	0.87413	NA	0.26174	0.27572	0.10889	13691	0.03566	0.189	.?...
GCC1	7	0.03297	0.39361	0.66434	NA	NA	3253	0.03567	0.189	...??
ANO4	12	NA	0.35864	0.15884	0.32268	0.20180	13245	0.03567	0.189	?....
HR	8	0.40260	0.78821	0.47752	NA	0.06993	11265	0.03569	0.189	...?.
PCDHB7	5	0.52048	0.40759	0.07692	0.51349	0.20080	14669	0.0357	0.189	.....
SEPSECS-AS1	4	0.54146	0.75624	0.64436	0.14386	0.16983	14669	0.03571	0.189	.....

ZDHHC20	13	0.47353	0.18482	0.02398	0.07792	0.84815	14669	0.03571	0.189	.....
LHX8	1	0.18881	0.34066	0.87413	0.12987	0.35964	14669	0.03571	0.189	.....
COQ3	6	0.50350	0.12987	0.46254	0.74226	0.11489	14669	0.03572	0.189	.....
NIPAL3	1	0.36763	0.06494	0.48352	0.01598	0.98601	14669	0.03577	0.189	.....
FAM135A	6	NA	0.59640	0.55644	0.04296	0.31568	13245	0.03577	0.189	?....
HTATIP2	11	0.12587	0.12488	0.87712	0.56643	0.21678	14669	0.03578	0.189	.....
TMEM192	4	0.39461	0.56044	0.50050	0.04096	0.46753	14669	0.03579	0.189	.....
SENP7	3	0.83816	0.43756	0.02597	0.15185	0.41059	14669	0.03583	0.189	.....
ZMAT2	5	0.10290	0.19580	0.05295	0.82318	0.35564	14669	0.03585	0.189	.....
YTHDC2	5	0.51648	0.78222	0.09391	NA	0.11588	11265	0.03586	0.189	...?.
KIAA0513	16	0.04695	0.62837	0.73626	0.60839	0.16484	14669	0.03587	0.189	.....
SLC35E4	22	0.00999	0.94406	0.32567	0.56244	0.30270	14669	0.03588	0.189	.....
IRS1	20	NA	0.25075	0.81319	0.17483	0.18182	13245	0.03588	0.189	?....
RAB2B	14	0.40160	0.97003	0.67433	0.37962	0.07792	14669	0.03588	0.189	.....
VASP	19	0.09590	0.59441	0.35664	0.88911	0.11888	14669	0.03591	0.189	.....
TRIL	7	0.80919	0.46953	0.42657	NA	0.06094	11265	0.03592	0.189	...?.
ZBTB3	11	0.61039	0.76324	0.16683	0.74126	0.06394	14669	0.03593	0.189	.....
RSAD2	2	0.10689	0.74825	0.07692	0.36563	0.37862	14669	0.03593	0.189	.....
IPMK	10	0.01099	0.06394	0.33966	0.26673	0.93307	14669	0.03593	0.189	.....
ABHD14B	3	NA	0.73626	0.13387	0.01299	0.63736	13245	0.03595	0.189	?....
SLC29A3	10	0.40060	0.10490	0.50450	0.33966	0.28172	14669	0.03601	0.189	.....
CLCC1	1	0.14785	0.67133	0.22677	0.69930	0.15185	14669	0.03602	0.189	.....
MTHFSD	16	0.94905	0.31169	0.88611	0.49051	0.04995	14669	0.03603	0.189	.....
EVA1A	2	0.27772	0.56543	0.83417	0.10390	0.29371	14669	0.03604	0.189	.....
LOC729296	20	0.35465	NA	0.07493	0.29371	0.27772	13691	0.03604	0.189	.?...
RAD51D	17	0.66933	0.62138	0.33367	0.30969	0.13187	14669	0.03605	0.189	.....
AAK1	2	0.09391	0.81918	0.61838	0.35265	0.19980	14669	0.03606	0.189	.....
MRC2	17	0.01099	0.52847	0.02897	0.41858	0.76024	14669	0.03606	0.189	.....
FCHO1	19	0.02997	0.65534	0.33267	0.05095	0.85914	14669	0.03606	0.189	.....
TECRL	4	0.08791	0.52248	0.70230	0.68332	0.13287	14669	0.03607	0.189	.....
LOC101927686	6	0.22178	0.61538	0.53047	0.15385	0.31169	14669	0.03607	0.189	.....
SCNN1B	16	0.30170	0.54945	0.42857	0.08991	0.40759	14669	0.03608	0.189	.....
TBXAS1	7	0.79620	0.40659	0.75924	NA	0.04695	11265	0.03608	0.189	...?.
ADAMTS8	11	0.83217	0.55245	0.63736	0.35365	0.07493	14669	0.03609	0.189	.....
LOC101928924	20	NA	0.10190	0.71229	NA	0.10290	9841	0.03611	0.189	?..?.
NHP2	5	0.28571	0.66733	0.27672	0.84915	0.07892	14669	0.03611	0.189	.....
ERG	21	0.65734	0.26074	0.12088	NA	0.16184	11265	0.03616	0.189	...?.
WNT10B	12	0.24476	NA	0.23077	0.37962	0.19880	13691	0.03617	0.189	.?...
PPARD	6	0.06993	0.54446	0.10490	0.56044	0.34166	14669	0.03618	0.189	.....
MXI1	10	0.21479	0.59341	0.54945	0.39261	0.16983	14669	0.03618	0.189	.....
LRRC8A	9	0.41958	NA	0.07393	0.16484	0.36264	13691	0.03618	0.189	.?...
DHX16	6	0.12488	0.56743	0.23477	0.83117	0.14386	14669	0.03618	0.189	.....
GCC2	2	0.08891	0.15884	0.23177	0.21578	0.66833	14669	0.03619	0.189	.....
MYH15	3	0.25574	0.16883	0.61738	0.40460	0.24276	14669	0.03622	0.189	.....
MIR5096	1	0.09690	0.03696	0.63836	0.12388	0.80320	14669	0.03622	0.189	.....
GMIP	19	0.98402	0.01898	0.30370	0.09191	0.56743	14669	0.03624	0.189	.....
DDX51	12	0.91908	0.50549	0.97602	NA	0.02797	11265	0.03626	0.189	...?.
FRMD5	15	0.62138	0.28971	0.26174	0.78521	0.08691	14669	0.03626	0.189	.....
TRIM6-TRIM34	11	0.33067	0.13487	0.21678	0.43457	0.32268	14669	0.03626	0.189	.....
LOC343052	1	0.80819	0.42857	0.75325	0.12687	0.17483	14669	0.03627	0.189	.....
CACNB3	12	0.12488	0.79021	0.24975	0.42058	0.23277	14669	0.03627	0.189	.....
C6orf89	6	0.09191	0.41059	0.80819	NA	0.15984	11265	0.03628	0.189	...?.
LOC728392	17	0.86314	0.96503	0.02298	0.51648	0.11389	14669	0.03629	0.189	.....
TMEM62	15	0.56444	0.37962	0.40460	0.46454	0.12488	14669	0.03629	0.189	.....
LAMB4	7	0.21479	0.75225	0.18681	0.21479	0.33666	14669	0.0363	0.189	.....
TDP2	6	0.20080	0.60739	0.88112	0.37562	0.13686	14669	0.03631	0.189	.....
LINC01493	11	0.41159	0.77722	0.30669	0.06693	0.38961	14669	0.03631	0.189	.....
MRPS31	13	0.07792	0.75025	0.51548	NA	0.15684	11265	0.03632	0.189	...?.
LOC440910	2	0.02398	0.76124	0.19880	0.68132	0.27672	14669	0.03635	0.189	.....
NCAM2	21	0.50250	0.76224	0.17383	0.03497	0.53147	14669	0.03636	0.189	.....
RNF175	4	0.58442	0.02298	0.02098	0.57043	0.49351	14669	0.03636	0.189	.....
SLFN12L	17	0.05195	0.96304	0.14585	0.70929	0.20080	14669	0.03636	0.189	.....
CXCR4	2	0.02597	0.86014	0.37962	0.16983	0.51548	14669	0.03637	0.189	.....
RGPD4	2	0.45155	0.10689	0.03896	0.05195	0.99101	14669	0.03637	0.189	.....
OR4E1	14	0.35864	0.98002	0.53946	0.42158	0.08691	14669	0.03638	0.189	.....
MTAP	9	0.13287	0.43457	0.97902	0.30969	0.20979	14669	0.03639	0.189	.....
LCN2	9	0.09391	0.11788	0.64136	0.16184	0.60639	14669	0.03639	0.189	.....
TRMO	9	0.71229	0.26873	0.62637	0.08092	0.32068	14669	0.0364	0.189	.....
OXTR	3	0.07592	0.18482	0.22378	0.71928	0.32268	14669	0.0364	0.189	.....

SNCB	5	0.02398	0.99700	0.15684	0.46753	0.34565	14669	0.03641	0.189	.....
SLC35B3	6	0.42957	0.23976	0.97502	0.29570	0.16284	14669	0.03641	0.189	.....
PACS1	11	0.40659	0.70430	0.16983	NA	0.12088	11265	0.03641	0.189	...?.
EIF3G	19	0.53147	0.22278	0.41059	0.27473	0.24575	14669	0.03644	0.189	.....
LINC00971	3	0.89510	0.13686	0.03596	0.14186	0.53946	14669	0.03649	0.189	.....
NXPH4	12	0.42458	0.62737	0.43656	0.16184	0.24675	14669	0.03649	0.189	.....
TMPRSS2	21	0.06593	0.12088	0.68032	0.55245	0.32168	14669	0.0365	0.190	.....
DCTN1-AS1	2	0.13786	0.06394	0.79321	0.27273	0.44955	14669	0.03653	0.190	.....
LOC101929312	20	0.56543	0.43457	0.25175	0.56344	0.11788	14669	0.03654	0.190	.....
DBI	2	0.33666	0.41558	0.28072	0.55744	0.15984	14669	0.03655	0.190	.....
DNAAF1	16	0.09391	0.01299	0.86913	0.69231	0.34565	14669	0.03656	0.190	.....
NFKBIZ	3	0.95305	0.05794	0.08492	0.80120	0.15684	14669	0.03657	0.190	.....
MIR1273H	4	0.79321	0.36064	0.79421	0.35165	0.08791	14669	0.03658	0.190	.....
ATP5G3	2	0.52248	0.32567	0.47752	0.44655	0.13686	14669	0.0366	0.190	.....
NCKAP1L	12	0.24376	0.39760	0.75924	0.15584	0.30969	14669	0.03663	0.190	.....
COL8A1	3	0.99201	0.86713	0.76823	0.01499	0.27872	14669	0.03664	0.190	.....
FEM1B	15	0.90010	0.10090	0.72228	0.02198	0.55345	14669	0.03664	0.190	.....
C11orf71	11	0.97602	0.05894	0.42557	0.09990	0.41059	14669	0.03665	0.190	.....
IL17F	6	0.22478	0.10290	0.66334	NA	0.20979	11265	0.03666	0.190	...?.
ZNF878	19	0.28571	0.92208	0.49351	0.07892	0.32967	14669	0.03666	0.190	.....
GPR37	7	0.39560	0.59241	0.17582	NA	0.13586	11265	0.03666	0.190	...?.
LINC01320	2	0.59740	0.21678	0.65634	0.04296	0.47852	14669	0.03667	0.190	.....
LINC00222	6	0.05794	0.69431	0.03297	0.46154	0.47453	14669	0.0367	0.190	.....
MFSD2B	2	0.98102	0.03097	0.10290	0.32068	0.37662	14669	0.0367	0.190	.....
ELAVL1	19	0.08392	0.65934	0.43756	0.15385	0.44655	14669	0.03672	0.190	.....
LINC00628	1	0.31968	NA	0.20180	0.09890	0.41259	13691	0.03673	0.190	.?...
ABCB10	1	0.46154	0.57542	0.60440	0.16883	0.20979	14669	0.03675	0.190	.....
ZFAND2A	7	0.89610	0.74925	0.07193	NA	0.08492	11265	0.03678	0.190	...?.
ABCA2	9	0.37662	0.84316	0.47752	0.17782	0.20280	14669	0.0368	0.190	.....
ERO1B	1	0.61439	0.04795	0.11089	0.22378	0.54246	14669	0.03681	0.190	.....
ADAM32	8	0.32767	0.30270	0.41359	NA	0.15085	11265	0.03681	0.190	...?.
SVBP	1	0.84316	0.44256	0.42757	0.13387	0.21578	14669	0.03681	0.190	.....
ORC6	16	0.06194	NA	0.19181	0.39860	0.33367	13691	0.03681	0.190	.?...
SCUBE1	22	0.16484	0.43656	0.02597	0.09391	0.88112	14669	0.03685	0.190	.....
PCYT2	20	NA	0.55944	0.11688	0.13586	NA	5233	0.03685	0.190	?...?
TMEM123	11	0.95005	0.50350	0.05295	0.12787	0.34665	14669	0.03686	0.190	.....
OPLAH	8	0.99001	0.11788	0.98601	0.28372	0.11688	14669	0.03688	0.190	.....
FRG1BP	20	0.06893	NA	0.91808	0.80719	0.07692	13691	0.03688	0.190	.?...
FLJ33534	2	0.21379	0.05994	0.02697	0.37063	0.73526	14669	0.03689	0.190	.....
ERF	19	0.21978	NA	0.35165	0.33766	0.19980	13691	0.0369	0.190	.?...
OR5T2	11	0.92907	0.45155	0.59341	0.71229	0.03497	14669	0.0369	0.190	.....
IP6K1	3	0.46254	0.22278	0.74925	0.24076	0.22278	14669	0.0369	0.190	.....
POT1	7	0.16084	0.03796	0.13087	NA	0.50549	11265	0.03691	0.190	...?.
C9orf152	9	0.70929	0.32268	0.49051	0.38262	0.12687	14669	0.03695	0.190	.....
VPS37C	11	0.68931	0.43057	0.97702	0.18382	0.13287	14669	0.03698	0.190	.....
TMEM79	1	0.10589	0.50350	0.87612	0.02298	0.70829	14669	0.03699	0.190	.....
C14orf28	14	0.37962	0.29770	0.08392	NA	0.23976	11265	0.03701	0.190	...?.
A2M	12	0.44755	0.04096	0.90210	0.30170	0.28272	14669	0.03703	0.190	.....
NKX2-2	20	0.89211	NA	0.04695	0.12687	0.30470	13691	0.03703	0.190	.?...
COBL	7	0.26773	0.16084	0.71329	NA	0.16284	11265	0.03705	0.190	...?.
TMEM41A	3	0.99001	0.39660	0.28072	0.37463	0.11189	14669	0.03705	0.190	.....
TBC1D3P2	17	0.46354	NA	0.49850	0.46254	0.08591	13691	0.03705	0.190	.?...
ZNF277	7	0.21279	0.89910	0.68432	0.16983	0.22078	14669	0.03706	0.190	.....
KIF1C	17	0.88911	0.15884	0.08192	0.99101	0.08991	14669	0.03707	0.190	.....
HS6ST1	2	0.77023	0.27972	0.18581	0.49451	0.14885	14669	0.03709	0.190	.....
CEP72	5	0.20380	0.30070	0.79820	0.05894	0.54246	14669	0.0371	0.190	.....
PCP2	19	0.48452	NA	0.26274	0.05195	0.42458	13691	0.0371	0.190	.?...
CLIP3	19	0.53347	0.06593	0.49051	0.27473	0.32767	14669	0.03712	0.190	.....
TSPAN14	10	0.39361	0.90909	0.05195	0.17383	0.36763	14669	0.03713	0.190	.....
NIF3L1	2	0.75924	0.38661	0.57842	0.01299	0.55644	14669	0.03714	0.190	.....
ARID5A	2	0.24775	0.72428	0.81019	0.30170	0.14486	14669	0.03715	0.190	.....
DHX8	17	0.48851	0.14685	0.00230	0.25574	0.77423	14669	0.03715	0.190	.....
SYT6	1	0.65335	0.81219	0.72228	0.52248	0.04396	14669	0.03718	0.190	.....
IL20	1	0.06593	0.09191	0.26773	0.46254	0.53846	14669	0.0372	0.191	.....
TEX36	10	0.85914	0.63736	0.12388	0.32268	0.15185	14669	0.03724	0.191	.....
MKRN9P	12	0.82617	0.01598	0.57343	0.17982	0.40859	14669	0.03725	0.191	.....
TMEM81	1	0.27073	0.53746	0.23277	0.11588	0.46853	14669	0.03726	0.191	.....
LIMS2	2	0.80719	0.19580	0.98202	0.66933	0.04795	14669	0.03726	0.191	.....
ADAMTS10	19	0.97602	0.80719	0.53047	0.03796	0.24476	14669	0.03727	0.191	.....

LOC100507291	3	0.65135	0.26074	0.08192	0.03696	0.75824	14669	0.03729	0.191	.....
HLA-DQA1	6	0.45554	0.82617	0.02498	NA	0.17782	11265	0.03729	0.191	...?.
PSMG2	18	0.40959	0.77323	0.30070	0.36364	0.14985	14669	0.0373	0.191	.....
RUSC1	1	NA	0.90609	0.16284	0.12188	0.24076	13245	0.0373	0.191	?....
CRYM	16	0.10989	0.95205	0.18681	0.30869	0.30370	14669	0.03733	0.191	.....
LINC01192	3	0.50649	0.64436	0.98801	0.10889	0.18482	14669	0.03733	0.191	.....
CCM2L	20	0.18482	0.23277	0.77223	0.03397	0.71528	14669	0.03734	0.191	.....
TPRA1	3	0.06593	0.28372	0.62837	0.72727	0.19980	14669	0.03735	0.191	.....
CCDC130	19	0.05994	0.51848	0.82118	0.04296	0.68531	14669	0.03736	0.191	.....
TMEM18	2	0.54645	0.63736	0.69331	0.00590	0.63536	14669	0.0374	0.191	.....
CASC6	6	0.64036	0.07592	0.13886	NA	0.24276	11265	0.03744	0.191	...?.
PLEKHM1P1	17	0.05295	NA	0.44655	0.53447	0.21079	13691	0.03744	0.191	.?...
MRPL4	19	0.80519	0.78022	0.20380	0.77622	0.04396	14669	0.03746	0.191	.....
BAG5	14	0.14086	0.33566	0.88412	0.85415	0.09091	14669	0.03748	0.191	.....
LIMD1	3	0.74026	0.19081	0.17782	0.28272	0.28072	14669	0.0375	0.191	.....
LOC440028	11	0.99600	0.94006	0.07093	0.28372	0.13387	14669	0.03751	0.191	.....
LIG1	19	0.25075	0.86014	0.48452	0.42657	0.12787	14669	0.03753	0.191	.....
KSR1	17	0.18981	0.39461	0.51848	0.02098	0.80320	14669	0.03753	0.191	.....
EPM2A	6	0.15684	0.33866	0.10390	0.41459	0.41159	14669	0.03755	0.191	.....
TRAF3IP3	1	NA	0.93906	0.35465	0.60140	0.04496	13245	0.03755	0.191	?....
NAPSB	19	0.79520	0.09990	0.47652	0.33666	0.20380	14669	0.0376	0.191	.....
LINC00879	3	0.20779	0.77822	0.34066	0.44855	0.16783	14669	0.03761	0.191	.....
GPC2	7	0.27972	0.18182	0.44356	0.25375	0.36963	14669	0.03762	0.191	.....
PGM3	6	0.41359	0.29570	0.15385	0.36164	0.29670	14669	0.03763	0.191	.....
ACP2	11	0.28072	0.76923	0.99600	0.04695	0.33766	14669	0.03765	0.191	.....
CLLU1OS	12	0.31369	0.86214	0.21578	0.36164	0.18382	14669	0.03765	0.191	.....
BIRC8	19	0.27672	0.12288	0.61039	0.82318	0.13287	14669	0.03768	0.191	.....
LINC00681	8	0.50350	0.32068	0.87013	0.08392	0.31568	14669	0.03768	0.191	.....
MFSD11	17	0.58541	0.13087	0.71628	0.03996	0.55045	14669	0.03768	0.191	.....
ICAM1	19	0.64036	0.08991	0.49850	0.18282	0.35065	14669	0.03771	0.191	.....
TEDDM1	1	0.70729	0.54146	0.50150	0.50549	0.07393	14669	0.03771	0.191	.....
MROH5	8	0.47053	0.21678	0.38661	0.25674	0.29171	14669	0.03772	0.191	.....
SMC3	10	0.32667	0.25974	0.46154	0.67532	0.13586	14669	0.03772	0.191	.....
TSNAX-DISC1	1	0.75924	0.57542	0.14486	0.79920	0.06494	14669	0.03772	0.191	.....
PROS1	3	0.19880	0.45455	0.59341	0.77722	0.09890	14669	0.03772	0.191	.....
LOC339874	3	0.51349	0.40559	0.21678	0.41459	0.18981	14669	0.03773	0.191	.....
CCDC97	19	0.11489	0.12587	0.94505	0.80220	0.15385	14669	0.03774	0.191	.....
FAM69B	9	0.51349	0.98302	0.56943	0.25075	0.11089	14669	0.03775	0.191	.....
APOL5	22	0.86813	0.90509	0.30969	0.23776	0.10989	14669	0.03776	0.191	.....
SLC45A2	5	0.23876	0.67732	0.06993	0.26474	0.39461	14669	0.03776	0.191	.....
PRR29-AS1	20	NA	0.03297	NA	0.22478	NA	4382	0.03777	0.191	?.?.?
PATL2	15	0.16384	0.78222	0.26374	0.62038	0.14985	14669	0.03778	0.191	.....
TAF3	10	0.54945	0.22378	0.19081	0.18981	0.39461	14669	0.03785	0.192	.....
LRRC31	3	0.11888	0.76224	0.79121	0.54745	0.11688	14669	0.03786	0.192	.....
TREML2	6	0.37363	0.63536	0.21279	0.47053	0.16384	14669	0.03786	0.192	.....
SP2	17	0.54146	0.84615	0.87313	0.33167	0.07193	14669	0.03786	0.192	.....
GAN	16	0.79820	0.73227	0.99101	0.46054	0.03596	14669	0.03787	0.192	.....
ITLN2	1	0.15584	0.97502	0.51948	0.18781	0.25774	14669	0.03787	0.192	.....
SPAG9	17	0.32168	0.90609	0.56743	0.15085	0.21978	14669	0.03791	0.192	.....
IQCF5-AS1	3	0.23377	0.29171	0.17782	0.31768	0.39660	14669	0.03793	0.192	.....
CLEC4G	19	0.64336	NA	0.38362	0.17383	0.17782	13691	0.03794	0.192	.?...
B4GALT4	3	0.30969	0.07892	0.46653	0.05594	0.77722	14669	0.03794	0.192	.....
SGK2	20	0.34865	NA	0.60240	0.89810	0.03896	13691	0.03795	0.192	.?...
TBC1D8	2	0.25475	0.17383	0.25774	0.43656	0.32867	14669	0.03796	0.192	.....
IL12RB2	1	0.99001	0.84216	0.80819	0.23177	0.06094	14669	0.03801	0.192	.....
ANKRD20A19P	13	0.02597	0.46254	0.50150	0.19980	0.57742	14669	0.03801	0.192	.....
PTRF	17	0.22178	0.53546	0.25375	0.06993	0.60140	14669	0.03801	0.192	.....
C11orf80	11	0.33067	0.40659	0.10090	0.59341	0.22078	14669	0.03802	0.192	.....
LOC102723557	5	0.50350	0.72727	0.73027	NA	0.05195	11265	0.03804	0.192	...?.
DUOXA2	15	0.14985	NA	0.23477	0.99401	0.08691	13691	0.03808	0.192	.?...
LOC100506124	2	0.25375	0.85614	0.02498	0.25874	0.42657	14669	0.03808	0.192	.....
NR6A1	9	0.09690	0.88212	0.11688	0.57542	0.23876	14669	0.03811	0.192	.....
FDX1L	19	0.39161	0.56144	0.56044	0.15485	0.26074	14669	0.03812	0.192	.....
TLK2	17	0.00599	0.64136	0.04795	0.21279	0.97802	14669	0.03813	0.192	.....
HTR1A	5	0.06693	0.87512	0.46254	0.04496	0.66633	14669	0.03818	0.192	.....
ADPRHL1	13	0.12388	0.05694	0.65335	0.24775	0.55445	14669	0.03819	0.192	.....
CCDC65	12	0.17882	0.29870	0.66733	0.95904	0.08891	14669	0.0382	0.192	.....
FADS6	17	0.42158	0.15584	0.14985	0.25774	0.44555	14669	0.0382	0.192	.....
MYO1F	19	0.66234	0.08691	0.97403	0.09990	0.34765	14669	0.03821	0.192	.....

ENAH	1	0.19780	0.88412	0.72428	0.03796	0.45754	14669	0.03821	0.192	.....
FKBP1AP1	19	0.90709	0.03497	0.81518	0.10390	0.37762	14669	0.03821	0.192	.....
ASB16	17	0.21479	0.46254	0.88711	0.71429	0.08192	14669	0.03822	0.192	.....
NDUFA4	7	0.73227	0.83516	0.37862	0.48551	0.06494	14669	0.03823	0.192	.....
ZNF534	19	0.67033	0.01299	0.78122	0.13287	0.49251	14669	0.03825	0.192	.....
FAM86HP	3	0.64835	0.14985	0.64735	0.53546	0.11988	14669	0.03826	0.192	.....
MPHOSPH8	13	0.02997	0.94006	0.93007	0.99800	0.06693	14669	0.03826	0.192	.....
OR4A47	11	0.14685	0.61439	0.73826	0.58941	0.11988	14669	0.03827	0.192	.....
MALL	2	0.86613	0.82817	0.07293	0.04795	0.43357	14669	0.03827	0.192	.....
E2F7	12	0.81518	0.80519	0.51349	0.02298	0.35564	14669	0.03828	0.192	.....
MYBPC3	11	0.44955	0.25574	0.54346	0.15784	0.33267	14669	0.03829	0.192	.....
HADHB	2	0.02997	0.41958	0.32567	0.33067	0.51449	14669	0.03829	0.192	.....
SGF29	16	0.82218	0.33866	0.52248	0.02198	0.50150	14669	0.03833	0.192	.....
ZFR	5	0.35265	0.56244	0.77223	0.04496	0.41958	14669	0.03833	0.192	.....
CEP85L	6	0.89910	0.70030	0.46753	0.03497	0.31568	14669	0.03834	0.192	.....
MIR5700	12	0.14386	0.35964	0.86813	0.23976	0.29570	14669	0.03836	0.192	.....
HCG8	6	0.79321	0.59341	0.16583	0.50749	0.10490	14669	0.03836	0.192	.....
SST	3	0.02897	0.83716	0.01698	0.23776	0.79321	14669	0.03837	0.192	.....
ACVR1	2	0.76923	0.34865	0.33067	0.64535	0.08292	14669	0.03837	0.192	.....
RCL1	9	0.18881	0.24176	0.59740	NA	0.18681	11265	0.0384	0.192	...?.
NFKBIL1	6	0.00440	0.06593	0.23876	0.52947	0.86913	14669	0.0384	0.192	.....
PPP1R12C	19	0.22178	0.05295	0.03596	0.59241	0.56344	14669	0.03841	0.192	.....
TRIM37	17	0.28971	0.27273	0.75125	0.04496	0.57443	14669	0.03842	0.192	.....
LOC100507201	2	0.31069	0.42657	0.67932	0.06593	0.44555	14669	0.03842	0.192	.....
BTF3P11	13	0.52348	NA	0.19580	NA	0.09790	10287	0.03844	0.192	.?.?.
LCN10	9	0.23676	0.35365	0.29371	0.23077	0.39261	14669	0.03844	0.192	.....
LPA	6	0.23976	0.44855	0.05395	0.54945	0.30669	14669	0.03846	0.192	.....
KLB	4	0.08791	0.11089	0.83816	0.20180	0.53147	14669	0.03846	0.192	.....
P2RX4	12	0.54446	0.86913	0.27972	0.12288	0.25874	14669	0.03849	0.192	.....
GYPE	4	0.21578	0.14985	0.29271	0.49051	0.32567	14669	0.0385	0.192	.....
LINC01159	2	0.16184	0.36164	0.84715	0.16583	0.35465	14669	0.03851	0.192	.....
BPIFA3	20	0.35864	0.21179	0.44755	0.48452	0.20280	14669	0.03851	0.192	.....
GCHFR	15	0.75624	0.77822	0.44256	0.30370	0.09890	14669	0.03851	0.192	.....
MAP3K10	19	0.45754	0.03996	0.83417	0.11788	0.49550	14669	0.03852	0.192	.....
PEX1	7	0.75924	0.86314	0.39361	0.27872	0.10390	14669	0.03853	0.192	.....
MIR217HG	2	0.63636	0.44256	0.72428	0.43357	0.08791	14669	0.03855	0.192	.....
PIGH	14	0.71628	0.48352	0.71429	0.61239	0.05095	14669	0.03855	0.192	.....
DYNC1LI1	3	0.47353	0.88412	0.21578	0.36364	0.14685	14669	0.03856	0.192	.....
CCT2	12	0.79021	0.54945	0.28172	0.09191	0.30370	14669	0.03857	0.192	.....
TOLLIP	11	0.69930	0.39361	0.84216	NA	0.05395	11265	0.03857	0.192	...?.
TASP1	20	0.35165	0.54845	0.05295	0.04695	0.78621	14669	0.03857	0.192	.....
IQCH	15	0.35465	0.75025	0.25874	0.03297	0.58941	14669	0.03857	0.192	.....
EIF4A1	17	0.17483	0.13786	0.57542	0.37263	0.34466	14669	0.03858	0.192	.....
LOC101928150	10	0.81918	0.24975	0.41059	0.44156	0.12587	14669	0.03859	0.192	.....
ELAVL3	19	0.58342	0.71329	0.68332	0.61439	0.04795	14669	0.0386	0.192	.....
LENG1	19	0.20280	0.55544	0.76024	0.06094	0.46254	14669	0.03862	0.192	.....
GABRA1	5	0.21578	0.04995	0.45754	NA	0.31568	11265	0.03863	0.192	...?.
RNF223	20	NA	0.36763	0.46753	0.21179	0.19580	13245	0.03863	0.192	?....
WFDC5	20	0.64336	0.12388	0.73626	0.43856	0.14685	14669	0.03864	0.192	.....
SHISA8	22	0.30969	0.16583	0.13786	0.50250	0.33467	14669	0.03867	0.192	.....
DEPDC1	1	0.81019	0.28871	0.93207	0.10390	0.21578	14669	0.03869	0.192	.....
MYL6B	12	0.01598	NA	0.27373	0.26474	0.54645	13691	0.0387	0.192	.?...
BTNL8	5	0.77023	0.30170	0.00799	0.60639	0.26573	14669	0.03871	0.192	.....
TAF13	1	0.11688	0.37463	0.34965	0.55544	0.25574	14669	0.03873	0.192	.....
SNAI3	16	0.00790	0.40559	0.05095	0.28272	0.96004	14669	0.03873	0.192	.....
LOC101928131	4	0.88811	0.54645	0.11089	0.70829	0.07792	14669	0.03877	0.192	.....
GDPD5	11	0.94006	0.00130	0.81718	0.14286	0.54146	14669	0.03877	0.192	.....
TLR6	4	0.83017	0.67732	0.22078	0.70130	0.05594	14669	0.03877	0.192	.....
AATK-AS1	17	0.00999	0.65534	0.71329	0.61938	0.26673	14669	0.03878	0.192	.....
LOC389199	4	0.47952	0.50450	0.05994	0.37762	0.28172	14669	0.0388	0.192	.....
COL11A2	6	0.93806	0.76923	0.37263	NA	0.04296	11265	0.03881	0.192	...?.
HYOU1	11	0.31069	0.24875	0.89910	0.96603	0.05794	14669	0.03882	0.192	.....
OR3A4P	17	0.00390	0.96903	0.48951	0.67832	0.28472	14669	0.03882	0.192	.....
C9orf153	9	0.00999	0.19580	0.70829	NA	0.42957	11265	0.03882	0.192	...?.
CARD6	5	0.60140	0.30969	0.61339	0.09790	0.32867	14669	0.03883	0.192	.....
SH2D6	2	0.53447	0.12987	0.84016	0.15684	0.30969	14669	0.03883	0.192	.....
DNAJC8	1	0.89810	0.30070	0.32168	0.02298	0.56543	14669	0.03883	0.192	.....
CECR7	22	0.03497	NA	0.75425	0.11988	0.48651	13691	0.03883	0.192	.?...
LINC01254	18	0.90410	0.47752	0.93207	0.71029	0.02597	14669	0.03883	0.192	.....

CNR2	1	0.72527	0.14985	0.11888	0.15085	0.48452	14669	0.03887	0.192	.....
VAPB	20	0.17782	0.02997	0.70929	0.70130	0.27273	14669	0.03888	0.192	.....
LOC103611081	11	0.13187	0.62537	0.61538	0.02897	0.68831	14669	0.03892	0.193	.....
RPL18A	19	0.45554	NA	0.04895	0.76823	0.12088	13691	0.03892	0.193	.?...
C19orf12	19	0.12587	0.44555	0.79021	0.61239	0.14286	14669	0.03893	0.193	.....
BRAT1	7	0.26773	0.95405	0.06993	0.27572	0.31469	14669	0.03894	0.193	.....
MMP25	16	0.65734	0.48352	0.70230	0.30170	0.11788	14669	0.03898	0.193	.....
SNRNP27	2	0.42857	0.35564	0.88711	0.73127	0.06194	14669	0.03898	0.193	.....
SMARCE1	17	0.09890	0.00590	0.73726	0.19081	0.86214	14669	0.03898	0.193	.....
ARL14	3	0.15584	0.56743	0.45854	0.03996	0.67832	14669	0.03898	0.193	.....
NEFH	22	0.03696	0.83716	0.47453	0.08292	0.63237	14669	0.039	0.193	.....
TCTA	3	0.65035	0.20879	0.94605	0.13786	0.24076	14669	0.03904	0.193	.....
PLEK2	14	0.36264	0.27073	0.47552	0.11289	0.44855	14669	0.03905	0.193	.....
SGCA	17	0.23377	0.22577	0.38062	0.17283	0.48851	14669	0.03906	0.193	.....
SH3RF3-AS1	2	0.57143	0.51548	0.02498	0.01898	0.94705	14669	0.03908	0.193	.....
LINC00996	7	0.41359	0.86214	0.55544	0.53846	0.07293	14669	0.03909	0.193	.....
SARNP	12	0.43956	0.19580	0.55245	0.76124	0.10490	14669	0.03914	0.193	.....
MANBAL	20	0.46254	0.35864	0.19980	0.94905	0.08891	14669	0.03914	0.193	.....
OR52A5	11	NA	0.27173	NA	0.07992	NA	4382	0.03915	0.193	?.?.?
LOC101928858	5	0.17083	0.53946	0.73826	0.11289	0.39161	14669	0.03916	0.193	.....
CD96	3	0.62438	0.30569	0.66833	0.14585	0.25475	14669	0.03916	0.193	.....
LINC01467	14	0.80420	0.26873	0.70729	NA	0.06693	11265	0.03918	0.193	...?.
AKR1C1	10	0.07393	0.33067	0.84016	0.21079	0.41459	14669	0.03918	0.193	.....
TESPA1	12	0.23576	0.63836	0.00510	0.62937	0.36863	14669	0.03918	0.193	.....
GAS2L2	17	0.45055	0.63636	0.45654	0.68432	0.07193	14669	0.03918	0.193	.....
PMAIP1	18	0.11988	0.18781	0.22877	NA	0.34865	11265	0.03919	0.193	...?.
SUN2	22	0.00899	0.50050	0.42857	0.26873	0.63536	14669	0.03923	0.193	.....
LOC401463	8	0.39061	0.02298	0.17982	NA	0.39461	11265	0.03923	0.193	...?.
LOC101928461	6	0.57642	0.78721	0.41558	0.77123	0.04496	14669	0.03923	0.193	.....
FAF1	1	0.54945	0.75425	0.46354	0.20879	0.17083	14669	0.03925	0.193	.....
CCDC89	11	0.76623	0.35065	0.06993	NA	0.16084	11265	0.03926	0.193	...?.
RASSF5	1	NA	0.10190	0.34765	0.06793	0.56044	13245	0.03933	0.193	?....
PTGDS	9	0.64236	0.25874	0.74126	0.07792	0.34665	14669	0.03934	0.193	.....
TRIM39	6	0.06294	0.58442	0.97702	0.91508	0.08292	14669	0.03936	0.193	.....
CCDC79	16	0.98901	0.80519	0.18382	0.04995	0.32468	14669	0.03937	0.193	.....
PCDHB19P	5	0.95105	0.87013	0.06893	0.66034	0.06793	14669	0.03938	0.193	.....
MS4A10	11	0.73826	0.34466	0.61339	0.05994	0.35465	14669	0.03939	0.193	.....
USP33	1	0.52947	0.67832	0.03097	0.52248	0.20879	14669	0.03943	0.194	.....
ELFN1	7	0.54545	0.27872	0.44456	NA	0.11888	11265	0.03945	0.194	...?.
CDC26	9	0.50450	0.05495	0.03397	0.59640	0.42458	14669	0.03945	0.194	.....
AP4B1	1	0.09590	0.37762	0.04496	0.92208	0.28971	14669	0.03948	0.194	.....
LOC101927915	8	0.79620	0.55245	0.03497	0.46953	0.18981	14669	0.03948	0.194	.....
GCDH	19	0.93606	0.10290	0.44655	0.23377	0.24675	14669	0.03949	0.194	.....
LOC253573	3	0.05894	0.03097	0.17582	0.62138	0.63636	14669	0.0395	0.194	.....
ZNF587B	19	0.71329	0.00180	0.76424	0.03097	0.97103	14669	0.03951	0.194	.....
GPN2	1	0.68332	0.86114	0.61838	0.13786	0.15485	14669	0.03955	0.194	.....
KCNJ4	22	0.70529	0.42458	0.41359	0.32867	0.14885	14669	0.03956	0.194	.....
LASP1	17	0.79421	0.42058	0.30170	0.15684	0.25674	14669	0.03957	0.194	.....
TNFRSF14	1	0.10490	0.70629	0.73427	0.39061	0.19381	14669	0.03957	0.194	.....
HS1BP3-IT1	2	0.20080	0.36364	0.32967	0.84615	0.13786	14669	0.03959	0.194	.....
LINC00575	4	0.50250	0.21479	0.58442	0.92907	0.06693	14669	0.0396	0.194	.....
SPINK6	5	0.35964	0.20879	0.66334	0.23277	0.29870	14669	0.03962	0.194	.....
PTGR2	14	0.49351	0.34665	0.65135	NA	0.09391	11265	0.03962	0.194	...?.
IFIT1B	10	0.49051	0.37363	0.66334	0.92308	0.04795	14669	0.03963	0.194	.....
SNRPD2	20	NA	0.65734	0.93207	0.48252	0.04496	13245	0.03963	0.194	?....
SLC35G1	10	0.04895	0.59441	0.91608	NA	0.16484	11265	0.03966	0.194	...?.
LOC101926964	1	0.11489	0.52947	0.76823	0.20280	0.33067	14669	0.03968	0.194	.....
LOC101927619	2	0.68432	0.70230	0.92208	0.35664	0.06494	14669	0.03968	0.194	.....
IBSP	4	0.54545	0.68531	0.71828	0.26274	0.12388	14669	0.03973	0.194	.....
CTCFL	20	0.52048	0.25275	0.64735	0.18981	0.26773	14669	0.03974	0.194	.....
SMC4	3	0.01040	0.91309	0.41958	0.71229	0.24575	14669	0.03974	0.194	.....
SLC13A5	17	0.65834	0.15584	0.72328	NA	0.10090	11265	0.03976	0.194	...?.
DYM	18	0.09491	0.21578	0.59441	NA	NA	3253	0.03978	0.194	...??
SKOR1	15	0.45155	0.78521	0.54545	0.10490	0.26474	14669	0.03979	0.194	.....
CCDC109B	4	0.98302	0.60639	0.04895	0.18082	0.28072	14669	0.0398	0.194	.....
CACNA1D	3	0.01698	0.00820	0.71528	0.26673	0.99301	14669	0.03981	0.194	.....
NHEG1	6	0.57243	0.35265	0.62138	0.92408	0.04595	14669	0.03981	0.194	.....
TMEM155	4	0.48352	NA	0.60639	0.07493	0.28771	13691	0.03981	0.194	.?...
FAM71F2	7	0.52747	0.65534	0.63137	0.39660	0.09890	14669	0.03982	0.194	.....

ATL2	2	0.13487	0.80320	0.13986	0.19680	0.45854	14669	0.03984	0.194	.....
SDR9C7	12	0.17483	0.86314	0.05994	0.09990	0.64336	14669	0.03986	0.194	.....
PHLDB3	19	0.64635	0.72128	0.56444	0.25874	0.12388	14669	0.03987	0.194	.....
TAS2R38	20	NA	0.44456	0.53746	0.50549	0.08192	13245	0.03991	0.194	?....
LOH12CR2	12	0.01030	0.21578	0.61439	0.29770	0.67233	14669	0.03992	0.194	.....
ITPR1	3	NA	0.84815	0.24575	0.93706	0.03197	13245	0.03993	0.194	?....
LOC553103	5	0.40160	0.03297	0.42158	0.36763	0.40559	14669	0.03996	0.195	.....
DUSP1	5	0.80919	0.72028	0.90709	NA	0.02997	11265	0.03998	0.195	...?.
DNAJB6	7	0.15285	0.75724	0.48452	0.44955	0.17782	14669	0.04	0.195	.....
TCAF1	7	0.78122	0.57542	0.13886	0.63037	0.09491	14669	0.04001	0.195	.....
PLSCR1	3	0.96603	0.02697	0.25275	0.11988	0.54745	14669	0.04001	0.195	.....
CSRP2BP	20	0.38561	0.10889	0.78721	0.06893	0.56244	14669	0.04002	0.195	.....
NICN1	3	0.27373	0.17982	0.84416	0.15784	0.38761	14669	0.04002	0.195	.....
LOC101928035	16	0.89510	0.91708	0.02797	NA	0.10390	11265	0.04006	0.195	...?.
GAD1	2	0.10889	0.05295	0.50549	0.27173	0.62238	14669	0.04007	0.195	.....
LOC102723448	3	0.97103	0.17882	0.10490	0.67532	0.13586	14669	0.04007	0.195	.....
PITPNA-AS1	17	0.82118	0.67433	0.09291	0.94605	0.05195	14669	0.04011	0.195	.....
VTN	17	0.90410	0.49351	0.77023	0.20380	0.11788	14669	0.04012	0.195	.....
C1D	2	0.24076	0.16683	0.27173	0.44456	0.34565	14669	0.04013	0.195	.....
PIWIL1	12	0.69730	0.88012	0.38761	NA	0.05295	11265	0.04013	0.195	...?.
AKIRIN2	6	0.67632	0.45155	0.10689	NA	0.14186	11265	0.04015	0.195	...?.
SLC16A8	22	0.45255	0.01598	0.20979	0.83017	0.28472	14669	0.04016	0.195	.....
SESN2	1	0.27273	0.21179	0.49850	0.04595	0.72228	14669	0.04024	0.195	.....
ALDH9A1	1	0.34366	0.96503	0.79021	0.03097	0.38561	14669	0.04027	0.195	.....
STK32C	10	0.49151	0.16084	0.70030	0.36963	0.19880	14669	0.04027	0.195	.....
WASF1	6	0.03796	0.36863	0.42058	0.54246	0.35465	14669	0.04034	0.195	.....
ERICH5	8	0.55844	0.14086	0.05694	0.47752	0.35265	14669	0.04035	0.195	.....
IVNS1ABP	1	0.10789	0.22677	0.93806	0.59540	0.19281	14669	0.04036	0.195	.....
GRHPR	9	0.29970	0.51648	0.03297	NA	0.28372	11265	0.04036	0.195	...?.
NR2F1	5	0.25574	NA	0.35764	0.22278	0.26873	13691	0.04038	0.196	.?...
CCDC144B	17	0.22977	NA	0.87013	NA	0.07792	10287	0.0404	0.196	.?.?.
P3H3	12	0.03696	0.95604	0.87512	0.05994	0.55045	14669	0.04041	0.196	.....
PEX13	2	0.35065	0.81319	0.94006	0.09391	0.23776	14669	0.04042	0.196	.....
ALDH18A1	10	0.57243	0.63037	0.40759	0.16084	0.23676	14669	0.04042	0.196	.....
OVGP1	1	0.42158	0.01898	0.76024	0.08591	0.69231	14669	0.04043	0.196	.....
NUDT16L1	16	0.76823	NA	0.85015	0.27872	0.07393	13691	0.04043	0.196	.?...
LOC101927653	12	0.93207	0.94406	0.54446	0.67532	0.02498	14669	0.04044	0.196	.....
ATXN10	22	0.47053	0.63237	0.79021	0.12987	0.21978	14669	0.04047	0.196	.....
AGBL3	7	0.01898	0.23776	0.49151	NA	0.42158	11265	0.04048	0.196	...?.
MCTP1	5	0.06394	0.44555	0.03097	NA	0.49750	11265	0.0405	0.196	...?.
LINC01080	13	0.11788	0.65634	0.24975	0.06094	0.71828	14669	0.0405	0.196	.....
MIS18BP1	14	0.15584	0.30270	0.07493	0.30070	0.57642	14669	0.0405	0.196	.....
TSG1	6	0.28871	0.63736	0.15784	NA	0.17483	11265	0.0405	0.196	...?.
LENG8	19	0.94406	0.53047	0.57742	0.01898	0.41658	14669	0.04051	0.196	.....
PTMS	12	0.55345	NA	0.06394	0.35564	0.22178	13691	0.04054	0.196	.?...
PTPRB	12	0.42757	0.20180	0.49550	0.47153	0.19381	14669	0.04055	0.196	.....
PFKFB4	3	0.83217	0.33866	0.75724	0.70030	0.04595	14669	0.04056	0.196	.....
EXOSC10	1	0.86713	0.08292	0.56643	0.78322	0.08392	14669	0.04057	0.196	.....
ATXN7L1	7	0.28372	0.04595	0.89810	0.05495	0.74226	14669	0.04058	0.196	.....
PRRT3	3	0.04895	0.52947	0.47552	0.13087	0.61439	14669	0.04058	0.196	.....
HLA-A	6	0.77722	0.15784	0.24975	NA	0.14985	11265	0.0406	0.196	...?.
FKBP3	14	0.76523	0.91908	0.15984	0.28272	0.14685	14669	0.04061	0.196	.....
PDCD6	5	0.53846	0.12288	0.50549	NA	0.15784	11265	0.04062	0.196	...?.
RNASEL	1	0.03896	0.26474	0.27672	0.91708	0.27672	14669	0.04063	0.196	.....
NGB	14	0.52048	0.26873	0.69131	0.11389	0.34066	14669	0.04064	0.196	.....
MRS2P2	12	0.41459	0.21079	0.80919	0.04396	0.55045	14669	0.04064	0.196	.....
UBE2D1	10	0.16284	0.44156	0.93906	0.49950	0.14785	14669	0.04068	0.196	.....
SRRT	7	0.27173	0.12288	0.70130	0.19580	0.42458	14669	0.04071	0.196	.....
LINC01387	18	0.40959	0.75025	0.29870	0.30669	0.19181	14669	0.04071	0.196	.....
NOC3L	10	0.99900	0.53147	0.68531	NA	0.03796	11265	0.04072	0.196	...?.
SPINK7	5	0.14086	0.75924	0.40160	0.31169	0.26773	14669	0.04072	0.196	.....
TRY2P	7	0.55744	0.93706	0.80619	0.04795	0.26074	14669	0.04074	0.196	.....
ZNF250	8	0.65035	0.68332	0.41259	NA	0.06793	11265	0.04075	0.196	...?.
ANP32D	12	0.58242	0.30569	0.76224	0.83417	0.05195	14669	0.04077	0.196	.....
CSTL1	20	0.71628	0.27872	0.04196	0.19980	0.46054	14669	0.04077	0.196	.....
MCM4	8	0.11189	0.33966	0.41858	0.11389	0.63836	14669	0.04079	0.196	.....
CHST4	16	0.81019	0.01050	0.23177	0.07592	0.82118	14669	0.04079	0.196	.....
LINC00482	17	0.76424	NA	0.70230	0.28671	0.08392	13691	0.04081	0.196	.?...
BRMS1L	14	0.38162	0.67832	0.51848	NA	0.08891	11265	0.04081	0.196	...?.

GTSF1	12	0.36563	0.84915	0.89011	0.36164	0.09291	14669	0.04081	0.196	.....
FIZ1	19	0.26174	0.96104	0.30869	0.42557	0.15385	14669	0.04083	0.196	.....
CLLU1	12	0.40260	0.82318	0.41758	0.40260	0.12687	14669	0.04084	0.196	.....
SPATA5L1	15	0.31269	0.07193	0.17383	0.36563	0.49650	14669	0.04084	0.196	.....
DSC1	18	0.16284	0.72028	0.54146	0.09291	0.44755	14669	0.04085	0.196	.....
UQCRC2	16	0.42857	0.41958	0.03197	0.27872	0.45554	14669	0.04086	0.196	.....
ARGLU1	13	0.80819	0.21479	0.01199	0.19580	0.56943	14669	0.04089	0.196	.....
CACNB4	2	0.36464	0.69031	0.66733	0.34066	0.13786	14669	0.04091	0.196	.....
PSMD12	17	0.19181	0.76024	0.92507	0.19281	0.21878	14669	0.04092	0.196	.....
SCARF2	22	0.78721	0.58142	0.44655	0.43357	0.08891	14669	0.04093	0.196	.....
UOX	1	0.02697	0.12987	0.46354	0.43956	0.57443	14669	0.04094	0.196	.....
TRIP10	19	0.36663	0.22677	0.18182	NA	0.23576	11265	0.04095	0.196	...?.
TP53BP2	1	0.45854	0.14386	0.72228	0.14985	0.37463	14669	0.04097	0.196	.....
NUDT16	3	0.37163	0.52747	0.54346	0.17483	0.27772	14669	0.04098	0.196	.....
TOP1	20	0.47053	0.13487	0.93606	0.32268	0.20480	14669	0.04098	0.196	.....
SYTL3	6	0.10490	0.55345	0.21878	0.23177	0.48751	14669	0.04099	0.196	.....
AFG3L1P	16	0.76224	0.78821	0.34965	0.28272	0.12388	14669	0.041	0.196	.....
OR10J3	1	0.25674	0.80519	0.64136	0.46054	0.11688	14669	0.041	0.196	.....
S1PR5	19	0.36863	NA	0.17083	0.34266	0.21978	13691	0.04102	0.196	.?...
HULC	6	0.32967	0.55145	0.87712	0.04096	0.44555	14669	0.04104	0.196	.....
NEU3	11	0.14785	0.01240	0.60340	NA	0.44156	11265	0.04104	0.196	...?.
OR1A2	17	0.28272	0.89610	0.79421	0.89810	0.03596	14669	0.04106	0.196	.....
GULP1	2	0.66533	0.94605	0.14086	0.20480	0.21079	14669	0.04109	0.196	.....
HLA-F	6	NA	0.91608	0.30969	0.14386	0.19181	13245	0.04113	0.196	?....
ZFP37	9	0.55744	0.26573	0.49151	0.13087	0.35864	14669	0.04115	0.196	.....
PSPH	7	0.74525	0.44855	0.06893	0.80519	0.10789	14669	0.04115	0.196	.....
NAMA	9	0.03596	0.64336	0.40959	0.46953	0.32867	14669	0.04116	0.196	.....
ING2	4	0.43856	0.89910	0.13886	0.67433	0.10190	14669	0.04116	0.196	.....
SAMD14	17	0.00018	0.11489	0.13586	0.95704	0.91109	14669	0.04117	0.196	.....
SUMO2	17	0.51548	0.10989	0.55644	0.12987	0.45255	14669	0.04117	0.196	.....
MTA3	2	0.16184	0.08691	0.87812	0.28272	0.40959	14669	0.04118	0.196	.....
LOC101927787	1	0.01598	0.66134	0.43556	0.79820	0.24076	14669	0.04118	0.196	.....
CLCN2	3	0.42957	0.26374	0.64535	0.05295	0.53047	14669	0.04121	0.196	.....
LOC100379224	19	0.60340	0.25475	0.55744	0.28871	0.20679	14669	0.04122	0.196	.....
KANSL1L	20	NA	0.69431	0.37263	0.02897	0.43956	13245	0.04124	0.196	?....
JOSD2	19	0.11588	0.77822	0.16184	0.33367	0.36064	14669	0.04127	0.196	.....
ERH	14	0.08292	0.12787	0.23477	NA	0.44156	11265	0.04128	0.196	...?.
PHYHD1	9	0.27273	0.95504	0.66234	0.11788	0.26374	14669	0.04128	0.196	.....
LOC101927575	9	0.15285	0.15884	0.55844	NA	0.25674	11265	0.04128	0.196	...?.
C10orf35	10	0.27972	0.52148	0.67532	NA	0.10889	11265	0.04131	0.196	...?.
THUMPD3	3	0.88911	0.02597	0.50649	0.02198	0.83017	14669	0.04131	0.196	.....
SV2C	5	0.71429	0.42857	0.57542	NA	0.07093	11265	0.04131	0.196	...?.
LDHAL6A	11	0.44555	0.21678	0.73027	0.47153	0.15385	14669	0.04131	0.196	.....
LOC101929371	20	0.83217	0.44356	0.71628	NA	0.05195	11265	0.04134	0.197	...?.
ANTXRL	20	NA	0.66533	0.09391	NA	0.11788	9841	0.04136	0.197	?..?.
NANOS3	19	0.48951	NA	0.06793	0.05395	0.60040	13691	0.04138	0.197	.?...
C16orf47	16	0.10190	0.69231	0.81518	NA	0.12987	11265	0.04138	0.197	...?.
HIVEP1	6	NA	0.85315	0.87612	0.01798	0.33467	13245	0.04139	0.197	?....
GABRA4	4	0.27273	0.21778	0.04995	0.56344	0.39461	14669	0.04139	0.197	.....
ZCCHC24	10	0.35764	0.97502	0.33666	NA	0.08791	11265	0.0414	0.197	...?.
BSN	3	0.45155	0.33367	0.97203	0.28472	0.16284	14669	0.04141	0.197	.....
WNT3A	1	0.16683	0.54545	0.61738	0.85115	0.09291	14669	0.04142	0.197	.....
SMPD4	2	0.96404	NA	0.15285	0.10689	0.25874	13691	0.04147	0.197	.?...
DLX6	7	0.35764	0.34765	0.22278	0.45255	0.24775	14669	0.04147	0.197	.....
KREMEN1	22	0.21678	0.98002	0.33966	0.55644	0.12488	14669	0.04148	0.197	.....
SPC24	19	0.70929	0.72128	0.49550	0.39161	0.08991	14669	0.0415	0.197	.....
TCHH	1	0.65834	0.29371	0.04496	0.32667	0.36164	14669	0.0415	0.197	.....
OR6C68	12	0.99700	0.80919	0.22478	NA	0.05295	11265	0.04152	0.197	...?.
CRLF1	19	0.10490	0.69031	0.02398	0.46853	0.46753	14669	0.04155	0.197	.....
CDH3	16	0.56643	0.06893	0.07493	0.46254	0.41159	14669	0.04155	0.197	.....
OR13A1	10	0.61638	0.70929	0.02897	NA	0.17283	11265	0.04157	0.197	...?.
TLK1	2	0.81319	0.87013	0.16084	0.32867	0.13187	14669	0.04158	0.197	.....
SACM1L	3	0.68931	0.01499	0.15984	0.59840	0.34865	14669	0.04159	0.197	.....
KIF2B	17	0.91109	0.15085	0.47153	0.64735	0.09391	14669	0.0416	0.197	.....
ANKRD16	10	0.29670	0.44555	0.11688	0.04496	0.81618	14669	0.0416	0.197	.....
PCAT5	10	0.62438	0.08791	0.07592	NA	0.30070	11265	0.0416	0.197	...?.
GPR62	3	0.20979	0.58442	NA	0.00899	0.80020	13818	0.04161	0.197	..?..
LINC01505	9	0.57842	0.17682	0.92907	0.06593	0.41059	14669	0.04162	0.197	.....
STAC3	12	0.74625	0.45654	0.44256	0.21878	0.19081	14669	0.04163	0.197	.....

RASSF9	12	0.80120	0.29970	0.34565	0.79620	0.06993	14669	0.04163	0.197	.....
GS1-279B7.1	1	0.20080	NA	0.44955	0.14186	0.35764	13691	0.04163	0.197	.?...
HPR	16	0.91908	0.01000	0.25874	0.63536	0.25574	14669	0.04164	0.197	.....
PWRN3	15	0.08691	0.85215	0.09091	0.23676	0.51748	14669	0.04165	0.197	.....
LINC01522	20	0.03896	0.99800	0.30869	0.47952	0.27473	14669	0.04166	0.197	.....
PTP4A3	8	0.34166	0.21778	0.83716	0.89910	0.07592	14669	0.04167	0.197	.....
COL28A1	7	0.20380	0.67832	0.24276	NA	0.17582	11265	0.04168	0.197	...?.
TSG101	11	0.36464	0.53147	0.37263	0.14186	0.36464	14669	0.04171	0.197	.....
AUNIP	1	0.99201	0.63137	0.56144	0.15085	0.14486	14669	0.04171	0.197	.....
RABGAP1L	1	0.01399	0.12488	0.80120	0.11688	0.93706	14669	0.04172	0.197	.....
CHURC1-FNTB	14	0.73526	0.53946	0.88312	0.15784	0.15285	14669	0.04174	0.197	.....
LOC101928523	9	NA	0.42358	NA	0.05994	NA	4382	0.04174	0.197	?.?.?
TYMP	22	0.09391	0.73726	0.57642	0.04795	0.64535	14669	0.04175	0.197	.....
YTHDF1	20	0.61938	0.54945	0.38462	0.09690	0.33167	14669	0.04177	0.197	.....
SNN	16	0.37063	0.40759	0.36164	0.10589	0.46154	14669	0.04179	0.197	.....
COPS2	15	0.77922	0.45554	0.12987	0.44955	0.16484	14669	0.04179	0.197	.....
LALBA	12	NA	NA	0.96903	0.57942	0.03197	12267	0.0418	0.197	??...
FAM136A	2	0.18382	0.56244	0.88611	0.17083	0.29271	14669	0.0418	0.197	.....
FAM26F	6	0.50949	0.02997	0.16084	NA	0.35764	11265	0.04183	0.197	...?.
ABLIM2	4	0.45754	0.24276	0.82817	0.10290	NA	6657	0.04183	0.197	....?
LINC01349	1	0.63337	0.68432	0.63337	0.11289	0.22178	14669	0.04184	0.197	.....
HTR6	1	0.51748	0.31069	0.41259	0.18581	0.31868	14669	0.04184	0.197	.....
LOC101927450	9	0.12587	0.28571	0.49251	0.19780	0.50150	14669	0.04186	0.197	.....
SLFN14	17	0.20180	0.51049	0.98501	0.03896	0.52647	14669	0.04187	0.197	.....
KIF14	1	0.62038	0.12388	0.93407	0.09790	0.36763	14669	0.04188	0.197	.....
CCDC170	6	NA	0.12587	0.41558	0.31668	0.24476	13245	0.0419	0.197	?....
UQCRC1	3	0.89610	0.48452	0.59441	0.49850	0.06593	14669	0.0419	0.197	.....
ABHD5	3	0.23477	0.34665	0.35964	0.25375	0.37862	14669	0.04192	0.197	.....
PATE1	11	0.48951	0.62038	0.15684	0.48651	0.16983	14669	0.04196	0.197	.....
LOC284648	1	NA	NA	NA	0.04196	NA	3404	0.04196	0.197	???.?
MVD	16	0.02997	0.66733	0.05195	0.16084	0.88911	14669	0.04196	0.197	.....
LOC105369635	20	NA	NA	NA	0.04196	NA	3404	0.04196	0.197	???.?
UGT3A1	5	0.85614	0.20879	0.61139	0.67732	0.07193	14669	0.04197	0.197	.....
FSCB	14	0.37263	0.43357	0.28372	NA	0.15784	11265	0.042	0.197	...?.
DAAM1	14	0.17283	0.71129	0.80320	0.02797	0.59341	14669	0.042	0.197	.....
LOC101928372	1	0.22777	0.81918	0.56044	0.25574	0.21778	14669	0.04202	0.197	.....
LINC00398	13	0.22577	NA	0.55145	0.43756	0.14785	13691	0.04202	0.197	.?...
LINC00460	13	0.95904	NA	0.13287	0.45554	0.09690	13691	0.04203	0.197	.?...
PAPLN	14	0.10190	0.15684	0.05894	0.79620	0.43357	14669	0.04203	0.197	.....
CYP4Z2P	1	0.82318	0.36464	0.57343	0.40360	0.10689	14669	0.04203	0.197	.....
ERBB2	17	0.54845	0.01598	0.72228	0.27473	0.40759	14669	0.04207	0.197	.....
LRRC58	3	0.03397	0.14086	0.15684	0.72028	0.51748	14669	0.04208	0.197	.....
OSER1-AS1	20	0.59041	0.35864	0.31469	0.37463	0.19481	14669	0.04208	0.197	.....
PSMC4	19	0.01499	0.64136	0.21578	0.66134	0.38262	14669	0.04209	0.197	.....
LZIC	1	0.36663	0.66134	0.77423	0.25175	0.16883	14669	0.0421	0.197	.....
LOC101927636	4	0.16084	0.41658	0.10490	0.07193	0.86214	14669	0.04211	0.197	.....
FCMR	1	0.08591	0.02697	0.49750	0.41758	0.61439	14669	0.04211	0.197	.....
NOSIP	19	0.59141	0.80519	0.53147	0.57343	0.06194	14669	0.04211	0.197	.....
ROCK1P1	18	0.28571	0.56144	0.35065	0.34965	0.23676	14669	0.04213	0.197	.....
TRIM64	11	0.39760	NA	0.95405	NA	0.05395	10287	0.04214	0.197	.?.?.
CRIP3	6	0.74226	0.87812	0.61139	0.13986	0.15285	14669	0.04214	0.197	.....
APOBR	16	0.80719	NA	0.28971	0.07393	0.29670	13691	0.04215	0.197	.?...
SPTB	14	0.91109	0.32667	0.34066	0.15085	0.26573	14669	0.04215	0.197	.....
PCDHB10	5	0.86613	0.39560	0.18981	0.47752	0.13586	14669	0.04215	0.197	.....
VSX2	14	0.81618	0.39461	0.40859	0.11489	0.29071	14669	0.04219	0.197	.....
VPS53	17	0.24975	0.85814	0.03796	0.77822	0.17982	14669	0.0422	0.197	.....
HGFAC	4	0.19381	0.28072	0.33866	0.53546	0.26773	14669	0.04221	0.197	.....
SYCE2	19	0.43756	0.72128	0.83516	0.15385	0.19381	14669	0.04221	0.197	.....
CTD-2194D22.4	5	0.26573	0.44256	0.47652	0.54945	0.16484	14669	0.04222	0.197	.....
NLRP11	19	0.11089	0.88611	0.31469	0.03097	0.77023	14669	0.04225	0.197	.....
CDH26	20	0.70829	0.05095	0.13287	0.73826	0.22977	14669	0.04225	0.197	.....
DUSP10	1	0.27173	0.46454	0.85115	0.23177	0.23477	14669	0.04229	0.197	.....
C15orf52	15	0.20180	0.08492	0.72428	0.62138	0.24176	14669	0.0423	0.197	.....
ATF6	1	0.90410	0.18482	0.38561	0.12288	0.34466	14669	0.0423	0.197	.....
NELFB	20	NA	NA	0.35165	NA	0.06693	8863	0.0423	0.197	??.?.
LNX1	4	0.52847	0.73626	0.77023	0.84915	0.03197	14669	0.04231	0.197	.....
NEURL2	20	0.41758	0.99500	0.15684	0.34466	0.18482	14669	0.04231	0.197	.....
PRDX6	1	0.28771	0.52947	0.54945	0.07992	0.45754	14669	0.04232	0.197	.....
NCOR1	17	0.82517	0.46653	0.27473	0.43856	0.12388	14669	0.04233	0.197	.....

LOC554223	6	0.33167	0.62937	0.06094	0.53447	0.24875	14669	0.04235	0.197 .....
EEF1A2	20	0.11988	0.42358	0.73027	0.72827	0.14086	14669	0.04236	0.197 .....
CIDEC	3	0.01598	0.93107	0.32168	0.07792	0.81918	14669	0.04237	0.197 .....
LOC105371335	16	0.76923	0.04995	0.15385	0.03097	0.94705	14669	0.04241	0.197 .....
C16orf82	16	0.18581	NA	0.25175	0.76024	0.12787	13691	0.04242	0.197 .?...
LINC00557	13	0.96903	0.55245	0.79421	NA	0.03596	11265	0.04243	0.197 ...?.
RSPO4	20	0.87912	0.27173	0.83516	0.74126	0.04396	14669	0.04244	0.197 .....
BMP8B	1	0.10190	0.81918	0.86913	0.89810	0.06593	14669	0.04245	0.197 .....
RDH5	12	0.59740	0.40360	0.85115	0.27972	0.14286	14669	0.04246	0.197 .....
FAM45A	10	0.96404	0.86513	0.93407	0.00999	0.33666	14669	0.04247	0.197 .....
ZNF442	19	0.08791	0.84515	0.20679	0.99600	0.12288	14669	0.04248	0.197 .....
LINC00867	10	0.28771	0.45155	0.97203	0.71828	0.07393	14669	0.0425	0.197 .....
ADAMTS15	11	0.66633	0.81818	0.50649	0.06693	0.28372	14669	0.04251	0.197 .....
LOC644554	19	0.56943	0.02098	0.79221	0.66034	0.18482	14669	0.04251	0.197 .....
CYP4X1	1	0.56444	0.64136	0.57642	0.41159	0.10490	14669	0.04253	0.197 .....
PRSS50	3	0.42258	0.79221	0.55345	0.18781	0.20679	14669	0.04255	0.197 .....
CRCP	7	0.94206	0.72328	0.97902	NA	0.02498	11265	0.04256	0.197 ...?.
IYD	6	0.04795	0.72727	0.12088	0.42957	0.44256	14669	0.04257	0.197 .....
BORA	13	0.28871	0.71429	0.51449	NA	0.10589	11265	0.04257	0.197 ...?.
M1AP	2	0.23377	0.30470	0.29670	0.44755	0.29471	14669	0.0426	0.197 .....
CTSH	15	0.45355	0.17483	0.28172	0.41359	0.28472	14669	0.0426	0.197 .....
TMPRSS3	21	0.02298	0.82018	0.59041	NA	0.23377	11265	0.04261	0.197 ...?.
CEP83-AS1	12	0.28472	0.58042	0.12088	0.16983	0.48452	14669	0.04261	0.197 .....
SCG3	15	0.49351	0.25275	0.17782	0.80020	0.14685	14669	0.04263	0.197 .....
ZNF251	8	0.73826	NA	0.88611	NA	0.03497	10287	0.04271	0.198 .?.?.
THEMIS2	1	0.23277	NA	0.40559	0.09391	0.43357	13691	0.04271	0.198 .?...
ANGEL1	14	0.50549	0.43856	0.08691	NA	0.19580	11265	0.04272	0.198 ...?.
POLD3	11	0.59840	0.59041	0.12987	NA	0.13387	11265	0.04273	0.198 ...?.
LINC01016	6	0.19381	0.87612	0.25974	NA	0.15185	11265	0.04274	0.198 ...?.
CCR1	3	0.10689	0.63237	0.17682	0.46953	0.32567	14669	0.04277	0.198 .....
FLAD1	1	0.10589	0.14386	0.71029	0.06693	0.81518	14669	0.04277	0.198 .....
GSN	9	0.22178	0.30669	0.76324	0.85315	0.09890	14669	0.04277	0.198 .....
ACSS1	20	0.58242	0.80919	0.05894	0.55644	0.15385	14669	0.04278	0.198 .....
SEMA4A	1	0.22078	0.45055	0.38462	0.97203	0.09790	14669	0.04278	0.198 .....
STEAP3	2	0.28771	0.52647	0.79820	0.00280	0.96803	14669	0.04278	0.198 .....
DEFB133	6	0.91209	0.62737	0.06693	0.04096	0.55145	14669	0.04279	0.198 .....
LINC00950	9	0.19181	0.70629	0.54246	0.62637	0.12288	14669	0.04283	0.198 .....
MYOT	5	0.11688	0.26374	0.89810	NA	0.19081	11265	0.04284	0.198 ...?.
NPW	20	NA	NA	0.09690	0.10889	0.35764	12267	0.04285	0.198 ??...
SULT1A2	16	0.89211	0.19680	0.44555	0.02298	0.61638	14669	0.04288	0.198 .....
CHMP2A	19	0.95804	NA	0.70030	0.17582	0.10589	13691	0.04288	0.198 .?...
FAM155A	13	0.06593	0.52647	0.36763	NA	NA	3253	0.04289	0.198 ...??
AMPD3	11	0.66434	0.06993	0.28971	0.81119	0.15485	14669	0.0429	0.198 .....
ZDHHC23	3	0.29970	0.39061	0.95105	0.02398	0.60939	14669	0.0429	0.198 .....
FAM131C	1	0.49151	0.03596	0.55944	0.24875	0.43856	14669	0.04291	0.198 .....
ZMIZ1	10	0.56444	0.19081	0.31968	NA	0.16883	11265	0.04294	0.198 ...?.
MIA	19	0.61538	0.45255	0.79920	0.16484	0.20180	14669	0.04295	0.198 .....
NDUFS1	2	0.12887	0.58442	0.81219	0.07293	0.50849	14669	0.04296	0.198 .....
ZNF844	19	0.14885	0.97003	0.30769	0.68332	0.12887	14669	0.04297	0.198 .....
ARHGAP35	19	0.70030	0.31568	0.38861	0.01698	0.69730	14669	0.04297	0.198 .....
LINC00707	10	0.55245	0.05794	0.64436	0.05495	0.67133	14669	0.04297	0.198 .....
RLBP1	15	0.19181	0.41459	0.36464	1.00000	0.10789	14669	0.04298	0.198 ...0.
ZNF461	19	0.46853	0.99600	0.33566	0.71728	0.05994	14669	0.04299	0.198 .....
YWHAEP7	17	0.30370	NA	0.70929	0.43457	0.11389	13691	0.043	0.198 .?...
RNPC3	1	0.82118	0.46653	0.79221	0.87213	0.02797	14669	0.04301	0.198 .....
EGF	4	0.81419	0.38961	0.36863	0.22478	0.20979	14669	0.04302	0.198 .....
YKT6	7	0.66034	0.19680	0.05994	0.67932	0.22278	14669	0.04303	0.198 .....
HSPC324	9	0.02897	0.09091	0.48651	0.21578	0.84715	14669	0.04304	0.198 .....
CNIH3	1	NA	0.65235	0.30769	0.35664	0.13087	13245	0.04305	0.198 ?....
LOC101929441	1	0.09391	NA	0.78921	NA	0.13387	10287	0.04307	0.198 .?.?.
RNMT	18	0.13387	0.51748	0.88911	0.33966	0.22577	14669	0.04308	0.198 .....
FOXP2	7	0.30669	0.18482	0.03796	NA	0.40759	11265	0.04308	0.198 ...?.
ARPC2	20	NA	0.72627	0.48252	0.14985	0.18981	13245	0.04308	0.198 ?....
MXRA7	17	0.75624	0.03596	0.18182	0.24775	0.49351	14669	0.0431	0.198 .....
APELA	4	0.92607	0.80819	0.66334	NA	0.03297	11265	0.04312	0.198 ...?.
ZBTB34	9	0.82218	0.52847	0.66833	NA	0.05195	11265	0.04313	0.198 ...?.
EBF3	10	0.00217	0.91908	0.46454	NA	0.40559	11265	0.04313	0.198 ...?.
WWC2-AS2	4	0.79321	NA	0.25275	0.44256	0.09690	13691	0.04313	0.198 .?...
LOC101929567	2	0.03497	0.45654	0.17383	0.27872	0.66134	14669	0.04314	0.198 .....

NBPF22P	5	0.08292	1.00000	0.55145	NA	0.14086	11265	0.04316	0.198	.0.?.
GUCY2D	17	0.35465	0.85614	0.09790	0.08392	0.53746	14669	0.04316	0.198	.....
OR13J1	9	0.16084	0.41059	0.79820	0.45055	0.19980	14669	0.04318	0.198	.....
OR2A2	7	0.16583	0.46354	0.03996	0.85115	0.26973	14669	0.04319	0.198	.....
ZNF629	16	NA	NA	0.12587	0.13487	NA	4255	0.04319	0.198	??..?
TLR2	4	0.24775	0.44356	0.00990	0.95604	0.26973	14669	0.04323	0.198	.....
RPS19	19	0.77822	NA	0.50350	0.18781	0.14785	13691	0.04324	0.198	.?...
ZNF252P-AS1	8	0.29071	0.13387	0.31369	NA	0.27173	11265	0.04326	0.198	...?.
LOC100506497	7	0.86414	0.78921	0.39161	NA	0.05095	11265	0.04326	0.198	...?.
CAMSAP3	19	0.51249	0.15085	0.24176	0.14785	0.52747	14669	0.04327	0.198	.....
MMRN2	10	0.10190	0.72328	0.31269	0.71429	0.17582	14669	0.04327	0.198	.....
UNC13D	17	0.28671	0.27772	0.55145	0.05894	0.63037	14669	0.04327	0.198	.....
ZNF718	4	0.86014	NA	0.28971	0.04695	0.35365	13691	0.04328	0.198	.?...
CHCHD3	7	0.29471	0.84216	0.74925	NA	0.07493	11265	0.04328	0.198	...?.
BPIFA4P	20	0.28072	0.88611	0.23676	0.37463	0.20579	14669	0.0433	0.198	.....
APOPT1	14	0.10190	0.56943	0.63536	0.92607	0.10290	14669	0.0433	0.198	.....
ARHGEF4	2	0.48851	0.79620	0.41159	0.79121	0.05594	14669	0.04334	0.198	.....
STON2	14	0.38861	0.50849	0.73427	NA	0.09091	11265	0.04334	0.198	...?.
ZNF8	19	0.73127	0.08192	0.07992	NA	0.28372	11265	0.04338	0.198	...?.
CLEC1A	12	0.41558	0.04895	0.87612	0.26174	0.35764	14669	0.04338	0.198	.....
DHPS	19	0.86713	0.03097	0.74226	0.34466	0.23576	14669	0.0434	0.198	.....
PDC	1	0.83317	0.37463	0.97303	0.10290	0.20779	14669	0.04342	0.198	.....
NFIB	9	1.00000	0.73926	0.34965	0.05295	0.29371	14669	0.04343	0.198	0....
TAF4B	18	0.42258	0.85514	0.81818	0.08591	0.25574	14669	0.04343	0.198	.....
GIMD1	4	0.87213	0.50250	0.47453	0.03696	0.40260	14669	0.04348	0.198	.....
FAM183BP	7	0.36264	NA	0.65734	0.26374	0.16683	13691	0.04349	0.198	.?...
DDI2	1	0.67632	0.76224	0.01299	0.09790	0.57443	14669	0.04351	0.198	.....
TAGLN3	3	0.81518	0.38561	0.23976	0.11389	0.35964	14669	0.04353	0.199	.....
TTLL10	1	0.21379	0.10789	0.28571	0.11588	0.78521	14669	0.04355	0.199	.....
FBP1	9	0.17083	0.19281	0.26174	0.66933	0.29171	14669	0.04356	0.199	.....
LOC100505625	5	0.63037	0.21279	0.66034	0.14486	0.31768	14669	0.04356	0.199	.....
ADAMTS12	5	NA	0.73227	0.99900	0.06793	0.19880	13245	0.04358	0.199	?....
LINC01599	14	0.71229	0.59041	0.23377	0.00630	0.82717	14669	0.04359	0.199	.....
PCNX2	1	0.84416	0.75425	0.84116	0.04296	0.24875	14669	0.04361	0.199	.....
C5orf56	5	0.65335	0.12587	0.39560	0.25475	0.32368	14669	0.04362	0.199	.....
TOP2A	17	0.38162	0.50949	0.51049	0.24476	0.24675	14669	0.04362	0.199	.....
MOCOS	18	0.18981	0.46553	0.98801	0.83417	0.07493	14669	0.04363	0.199	.....
TIMM23B	10	0.06593	0.97003	0.05594	NA	0.32667	11265	0.04365	0.199	...?.
FNDC3B	3	0.35964	0.98002	0.46054	0.21778	0.19680	14669	0.04367	0.199	.....
AHCTF1	1	0.76823	0.53646	0.04895	0.23576	0.32967	14669	0.04367	0.199	.....
KTN1	14	0.72827	0.09191	0.47952	0.10290	0.48352	14669	0.04367	0.199	.....
HIST1H4H	6	0.64436	1.00000	0.31469	0.11988	0.23277	14669	0.04369	0.199	.0...
ABCC2	10	0.56643	0.61838	0.26474	0.99201	0.05195	14669	0.04369	0.199	.....
BMP5	6	0.15884	0.23177	0.65135	NA	0.21778	11265	0.04371	0.199	...?.
PLEKHM2	1	0.46154	0.77722	0.06394	0.28871	0.30569	14669	0.04372	0.199	.....
SNCAIP	5	0.33566	0.66134	0.98302	NA	0.06793	11265	0.04372	0.199	...?.
POP4	19	0.24076	0.18881	0.99600	0.85015	0.09890	14669	0.04374	0.199	.....
MPZL3	11	0.49351	0.40360	0.22178	NA	0.15784	11265	0.04375	0.199	...?.
PRSS27	20	NA	NA	0.52348	0.05295	NA	4255	0.04376	0.199	??..?
PRKCG	19	0.22078	NA	0.27273	0.34266	0.25275	13691	0.04376	0.199	.?...
FOXK2	17	0.94406	0.74026	0.10889	NA	0.08492	11265	0.04378	0.199	...?.
LOC101927513	8	0.42757	0.10390	0.63237	NA	0.18282	11265	0.04378	0.199	...?.
CALB2	16	0.84316	0.39061	0.10989	0.35165	0.22378	14669	0.04379	0.199	.....
FKBP2	11	0.42058	NA	0.76424	0.27572	0.13686	13691	0.0438	0.199	.?...
STAM-AS1	10	0.60739	0.17782	0.67632	0.22478	0.26573	14669	0.04381	0.199	.....
TMEM140	7	0.02098	0.17782	0.66533	NA	0.42058	11265	0.04381	0.199	...?.
PTGDR2	11	0.49550	0.04096	0.87712	0.82917	0.12488	14669	0.04386	0.199	.....
LINC01498	12	0.73127	0.16384	0.79321	0.06194	0.42757	14669	0.04388	0.199	.....
ABLIM1	10	0.58142	0.50949	0.89910	0.00640	0.65634	14669	0.04391	0.199	.....
PAH	12	0.70729	0.65135	0.11888	0.22078	0.26973	14669	0.04392	0.199	.....
HOXB6	17	0.17782	0.30669	0.51848	0.36264	0.31868	14669	0.04393	0.199	.....
NPIPB8	20	NA	NA	NA	0.04396	NA	3404	0.04396	0.199	???.?
LOC101929460	6	0.32068	0.83616	0.79321	NA	0.06993	11265	0.04396	0.199	...?.
MIR4277	5	NA	0.04396	NA	NA	NA	978	0.04396	0.199	?.???
ACOX3	4	0.65435	0.30270	0.34366	0.57942	0.13187	14669	0.04398	0.199	.....
KPNA3	13	0.19181	0.65934	0.43457	NA	0.15285	11265	0.04398	0.199	...?.
FARS2	6	0.97303	0.53546	0.90909	NA	0.03397	11265	0.04398	0.199	...?.
GPC6	13	0.18581	0.44555	0.15884	NA	NA	3253	0.04398	0.199	...??
LINC00989	4	0.87413	0.18382	0.18581	0.05994	0.59141	14669	0.04399	0.199	.....

SOX5	12	0.22178	0.56444	0.08192	NA	NA	3253	0.04399	0.199	...??
PKMYT1	16	0.00380	0.19680	0.36863	0.25475	0.98501	14669	0.044	0.199	.....
LOC101927358	9	0.82817	0.56144	0.60939	NA	0.05395	11265	0.044	0.199	...?.
CAST	5	0.51249	0.35165	0.50150	0.13786	0.34865	14669	0.04402	0.199	.....
GNGT1	7	0.96603	0.35564	0.29570	NA	0.08791	11265	0.04402	0.199	...?.
GRM8	7	0.07293	0.25974	0.68232	NA	NA	3253	0.04403	0.199	...??
DTNBP1	6	0.85814	0.45155	0.66533	0.08891	0.25574	14669	0.04404	0.199	.....
ATP5S	14	0.33966	0.41558	0.20879	0.61738	0.19281	14669	0.04406	0.199	.....
COPE	19	0.55644	0.40759	0.73127	0.02198	0.56044	14669	0.04406	0.199	.....
SPAG4	20	0.53646	0.65734	0.78521	0.11988	0.22777	14669	0.04408	0.199	.....
MAPK14	6	0.74326	0.87512	0.10589	0.02597	0.58042	14669	0.04409	0.199	.....
NUDT16P1	3	0.57243	0.37762	0.47552	0.21878	0.25275	14669	0.04409	0.199	.....
TBX20	7	0.32667	0.38861	0.24875	NA	0.19481	11265	0.0441	0.199	...?.
COPS7A	12	0.04296	0.19281	0.09291	NA	0.59540	11265	0.04411	0.199	...?.
TDH	8	0.53447	0.11489	0.79421	0.33566	0.23077	14669	0.04412	0.199	.....
DLX5	7	0.79421	0.68132	0.88112	0.11089	0.16683	14669	0.04412	0.199	.....
HSD17B3	9	0.67133	0.78422	0.13986	NA	0.10290	11265	0.04414	0.199	...?.
UBTD2	5	0.96803	0.27972	0.60340	0.66134	0.06094	14669	0.04415	0.199	.....
LPAR2	19	0.71828	0.53546	0.07892	0.08292	0.51449	14669	0.04415	0.199	.....
LINC01170	5	0.61439	0.26973	0.19880	NA	0.17083	11265	0.04417	0.199	...?.
SLC4A5	2	0.12787	0.25874	0.34366	0.24775	0.53247	14669	0.0442	0.199	.....
LINC00602	6	0.25175	0.24076	0.24476	0.21778	0.51748	14669	0.0442	0.199	.....
MOSPD3	7	0.06494	0.72627	0.26274	0.58042	0.27273	14669	0.04421	0.199	.....
KLF7	2	0.03297	0.93706	0.36264	0.05594	0.80020	14669	0.04423	0.199	.....
ZBTB22	6	0.72727	0.85614	0.25874	0.24076	0.16783	14669	0.04423	0.199	.....
PPP1R10	6	0.28472	0.79920	0.15784	0.44755	0.21978	14669	0.04423	0.199	.....
LINC00624	1	0.56044	0.51848	0.60739	0.04895	0.42358	14669	0.04424	0.199	.....
AKT2	19	0.94605	0.01998	0.88611	0.10989	0.42857	14669	0.04427	0.199	.....
IFT80	3	0.02098	0.95405	0.39760	0.49850	0.31169	14669	0.04427	0.199	.....
TPSD1	20	NA	0.12388	0.39461	0.33566	0.25275	13245	0.04427	0.199	?....
RTCB	22	0.15385	0.29970	0.55944	0.61838	0.21379	14669	0.04428	0.199	.....
DRAM1	12	0.49650	0.16583	0.52747	0.61039	0.15784	14669	0.04431	0.199	.....
FUT7	9	0.15584	0.40859	0.50150	0.80519	0.14585	14669	0.04432	0.199	.....
CAPS	19	0.63337	0.99500	0.93107	0.03896	0.25275	14669	0.04434	0.199	.....
IFNLR1	1	0.94605	0.95205	0.20779	0.03197	0.38861	14669	0.04439	0.199	.....
C1QTNF2	5	0.96104	0.02797	0.41259	0.45754	0.23776	14669	0.04439	0.199	.....
PEA15	1	0.90709	0.67632	0.48751	0.01898	0.44156	14669	0.04441	0.199	.....
USP12	13	0.95804	0.28871	0.00560	NA	0.27672	11265	0.04442	0.199	...?.
CD300LG	17	0.41259	0.30769	0.52448	0.19680	0.33267	14669	0.04446	0.199	.....
PLEKHA5	12	0.99600	0.94605	0.05594	NA	0.08292	11265	0.04449	0.199	...?.
KLK14	19	0.85814	0.89710	0.95305	0.34266	0.04995	14669	0.04452	0.200	.....
SPNS3	17	0.92807	0.43157	0.23976	0.05794	0.43157	14669	0.04453	0.200	.....
LOC101928820	6	0.12587	0.04496	0.22178	0.77123	0.43257	14669	0.04454	0.200	.....
UFC1	1	0.33167	0.50150	0.31269	0.33766	0.26274	14669	0.04456	0.200	.....
AMHR2	12	0.32368	0.42857	0.60240	0.05794	0.53546	14669	0.04456	0.200	.....
BAIAP3	16	0.11588	0.74725	0.86513	0.12488	NA	6657	0.04456	0.200	....?
LOC101927244	1	0.20679	0.14186	0.01598	0.70230	0.52348	14669	0.04459	0.200	.....
MIR17HG	13	0.44755	0.98302	0.22577	0.19780	0.24775	14669	0.04459	0.200	.....
LSM14A	19	0.94605	0.97802	0.24875	0.26773	0.11389	14669	0.04461	0.200	.....
GMPPB	3	0.47153	0.12687	0.78222	0.35764	0.23377	14669	0.04462	0.200	.....
FRMD3	9	0.47652	0.44356	0.36563	NA	0.12887	11265	0.04462	0.200	...?.
KCNAB2	1	0.84216	0.37163	0.23776	0.52947	0.12687	14669	0.04462	0.200	.....
C10orf2	10	0.42058	0.10989	0.36863	0.62637	0.22677	14669	0.04463	0.200	.....
LRRFIP1	20	NA	0.09890	0.11988	0.06194	0.77822	13245	0.04464	0.200	?....
KLC3	19	0.41359	0.54745	0.96304	0.01598	0.56444	14669	0.04467	0.200	.....
PRDM4	12	0.03397	0.72028	0.33966	0.62238	0.28671	14669	0.04467	0.200	.....
ARHGDIG	16	0.99001	NA	0.90310	0.13786	0.10689	13691	0.04468	0.200	.?...
TRIM41	5	0.37662	NA	0.06394	0.05794	0.68032	13691	0.04468	0.200	.?...
PGAM5	12	0.22577	0.16983	0.68232	NA	0.20879	11265	0.04468	0.200	...?.
OR52B4	11	0.03397	0.79021	0.44356	NA	0.25275	11265	0.0447	0.200	...?.
CPLX3	15	0.06394	0.45255	0.92308	NA	0.19181	11265	0.04471	0.200	...?.
ANKRD18DP	3	0.10090	NA	0.46454	0.91708	0.10390	13691	0.04471	0.200	.?...
GNG13	20	NA	NA	0.91009	0.05395	0.23377	12267	0.04472	0.200	??...
LINC00970	1	0.44955	0.56244	0.59740	0.05794	0.43157	14669	0.04475	0.200	.....
LINC00901	3	0.16384	0.83716	0.28472	0.06793	0.60440	14669	0.04477	0.200	.....
ZNF71	19	0.98202	0.09590	0.31069	0.02498	0.74026	14669	0.04485	0.200	.....
N4BP3	5	0.18382	0.96503	0.56643	0.60739	0.10589	14669	0.04486	0.200	.....
PDE6G	17	0.06294	0.74126	0.22677	0.18082	0.58342	14669	0.04488	0.200	.....
OPRL1	20	0.03397	0.34665	0.95405	0.89411	0.16583	14669	0.04488	0.200	.....

SERPINB5	18	0.52647	0.12887	0.28871	0.11489	0.58741	14669	0.04491	0.200	.....
HLA-DMA	6	0.36364	0.41958	0.16983	0.29870	0.35564	14669	0.04492	0.200	.....
LOC101930114	1	0.23277	0.44056	0.45155	0.90410	0.10589	14669	0.04493	0.200	.....
HN1	17	0.36064	NA	0.68731	0.02298	0.53347	13691	0.04493	0.200	.?...
TMEM133	11	0.52947	0.68731	0.69630	0.28272	0.13886	14669	0.04495	0.200	.....
RASL10B	17	0.33966	0.07892	0.69231	0.37463	0.32268	14669	0.04496	0.200	.....
RALGAPA1	14	0.70030	0.75824	0.82118	NA	0.04196	11265	0.04497	0.200	...?.
SECISBP2	9	0.43357	0.87512	0.20679	0.80719	0.07892	14669	0.045	0.200	.....
PLA2G4D	15	0.88511	0.53846	0.72827	0.30170	0.09890	14669	0.045	0.200	.....
ALPK3	15	0.18082	0.62837	0.19281	NA	0.22278	11265	0.04501	0.200	...?.
RIPK4	21	0.93806	0.07692	0.04895	0.82617	0.20180	14669	0.04503	0.200	.....
LIX1	5	0.06294	0.47952	0.29970	NA	0.31369	11265	0.04504	0.200	...?.
LINC00587	9	0.71928	0.09391	0.59740	0.32268	0.24675	14669	0.04505	0.200	.....
ADGRA1	10	0.34965	0.93307	0.50549	0.86513	0.04995	14669	0.04506	0.200	.....
NTRK2	9	0.21479	0.75824	0.84116	NA	0.09291	11265	0.04508	0.200	...?.
ZFAND1	8	0.00799	0.16783	0.98402	0.67732	0.40460	14669	0.0451	0.200	.....
CD1C	1	0.43457	NA	0.65335	0.16983	0.21179	13691	0.0451	0.200	.?...
LIN52	14	0.16583	0.93606	0.41958	0.87612	0.08292	14669	0.04513	0.200	.....
NEU1	6	0.22478	0.95604	0.08791	0.38262	0.29171	14669	0.04514	0.200	.....
STK10	5	0.01698	0.78422	0.63836	0.28671	0.44655	14669	0.04514	0.200	.....
MYNN	3	0.49151	0.30869	0.97203	0.12288	0.29471	14669	0.04515	0.200	.....
ABI2	2	0.64635	0.88012	0.25774	0.22877	0.18981	14669	0.04515	0.200	.....
BTNL10	1	0.20579	0.24476	0.65634	0.17183	0.46254	14669	0.04519	0.200	.....
LOC392364	9	0.12987	0.22478	0.35664	NA	0.31169	11265	0.04521	0.200	...?.
CNOT10	3	0.59341	0.64535	0.72927	0.10789	0.24476	14669	0.04521	0.200	.....
LOC101927765	1	0.14386	NA	0.08392	0.90809	0.17283	13691	0.04521	0.200	.?...
ELDR	7	0.47053	0.69530	0.31768	NA	0.10789	11265	0.04523	0.200	...?.
APEH	3	0.28272	0.92707	0.63237	0.22278	0.20280	14669	0.04527	0.201	.....
RNF213	17	0.35465	0.75824	0.45155	NA	0.10290	11265	0.04527	0.201	...?.
HEMGN	9	0.63936	NA	0.81518	0.08991	0.21578	13691	0.04529	0.201	.?...
SMAD2	18	0.06194	0.48651	0.10090	NA	0.41858	11265	0.04531	0.201	...?.
SMARCAL1	20	NA	0.99401	0.37363	0.53946	0.05994	13245	0.04531	0.201	?....
RRN3	16	0.66334	0.28771	0.29471	0.55744	0.15285	14669	0.04532	0.201	.....
LOC100507373	19	0.46853	NA	0.02098	0.17083	0.50549	13691	0.04533	0.201	.?...
TMEM89	3	0.46254	0.02098	0.90310	0.18881	0.48651	14669	0.04533	0.201	.....
ZNF430	19	0.80819	0.17183	0.52547	0.08492	0.42458	14669	0.04535	0.201	.....
LYSMD3	5	0.50150	0.06793	0.15385	0.83117	0.23676	14669	0.04536	0.201	.....
CA3	8	0.51349	0.73427	0.08791	0.09391	0.50549	14669	0.04536	0.201	.....
MYO18B	22	0.38462	0.89211	0.05095	0.05594	0.68032	14669	0.04536	0.201	.....
LOC101929353	4	0.33866	0.61339	0.65634	0.02697	0.58841	14669	0.04537	0.201	.....
CCDC115	2	0.15185	0.70529	0.75724	0.08092	0.46354	14669	0.04538	0.201	.....
LINC01044	13	0.42258	0.71129	0.92507	0.29770	0.12587	14669	0.0454	0.201	.....
LOC284395	19	0.87113	0.69930	0.61239	0.66234	0.03996	14669	0.04541	0.201	.....
TAAR2	6	0.46454	0.47453	0.38062	0.22478	0.28372	14669	0.04542	0.201	.....
INPP5K	17	0.98901	0.63437	0.48551	0.30270	0.10190	14669	0.04545	0.201	.....
GPR155	2	0.87313	0.83916	0.18781	0.47153	0.09491	14669	0.04545	0.201	.....
VCP	9	0.64336	0.05495	0.51149	0.84915	0.13287	14669	0.04547	0.201	.....
APPL2	12	0.08991	0.44855	0.52148	0.84715	0.16384	14669	0.04547	0.201	.....
LOC286178	8	0.99301	0.98501	0.22877	NA	0.04895	11265	0.0455	0.201	...?.
DBX1	11	0.02997	0.86014	0.87413	0.28771	0.32468	14669	0.04551	0.201	.....
TIA1	2	NA	0.71029	0.97003	0.03996	0.27572	13245	0.04555	0.201	?....
ZBTB42	14	0.01199	0.92308	0.78022	0.30769	0.39161	14669	0.04556	0.201	.....
LOC101929147	1	0.51848	0.21878	0.77822	0.42258	0.16484	14669	0.04557	0.201	.....
LOC100507277	14	0.83916	NA	0.03197	0.19980	0.32068	13691	0.04559	0.201	.?...
HEATR5B	2	NA	0.30270	0.42058	0.37263	0.17183	13245	0.04562	0.201	?....
HOXB7	17	0.18382	0.20280	0.45355	0.48851	0.31568	14669	0.04564	0.201	.....
LAMB2	3	0.18681	0.80020	0.41958	0.07592	0.52248	14669	0.04564	0.201	.....
LOC101928682	16	0.36464	0.82517	0.68631	0.96703	0.03696	14669	0.04567	0.201	.....
NR3C1	5	0.03996	0.10589	0.81518	0.43357	0.49251	14669	0.04569	0.201	.....
FAIM2	12	0.42757	0.15684	0.66633	0.24276	0.33067	14669	0.04569	0.201	.....
MYT1L-AS1	2	0.84116	0.84915	0.25974	0.33367	0.11988	14669	0.0457	0.201	.....
OR52B6	11	0.40360	0.96603	0.76124	0.00350	0.73826	14669	0.04571	0.201	.....
USP2-AS1	11	0.11089	0.69131	0.40759	0.21279	0.41758	14669	0.04572	0.201	.....
ARHGDIB	12	0.64935	0.51149	0.13287	NA	0.14486	11265	0.04575	0.201	...?.
ABCB8	7	0.89011	0.52048	0.04296	0.26274	0.30270	14669	0.04575	0.201	.....
POLR1D	13	0.40859	0.47652	0.57542	0.39560	0.17083	14669	0.04576	0.201	.....
LOC101928751	1	0.04695	0.08392	0.01898	0.52647	0.93307	14669	0.04577	0.201	.....
ZBTB24	6	0.30869	0.32068	0.94406	0.40160	0.17483	14669	0.04579	0.201	.....
ARL4D	17	0.63636	NA	0.08591	0.13686	0.37762	13691	0.04579	0.201	.?...

LINC01538	18	0.90010	0.91009	0.02298	NA	0.12488	11265	0.04582	0.201	...?.
LMF1	16	0.56743	0.81319	0.00210	NA	0.29770	11265	0.04587	0.201	...?.
PATZ1	22	0.34865	0.74426	0.84515	0.60040	0.07393	14669	0.0459	0.202	.....
KLRF1	12	0.83117	0.03796	0.12188	0.65934	0.27373	14669	0.04592	0.202	.....
RFXANK	19	0.37363	0.79920	0.54845	0.69630	0.07393	14669	0.04592	0.202	.....
WSB1	17	0.93107	0.93007	0.70330	0.17083	0.10589	14669	0.04593	0.202	.....
OR52B2	11	NA	NA	NA	0.04595	NA	3404	0.04595	0.202	???.?
LOC100996455	11	0.73726	NA	0.83916	0.45954	0.05495	13691	0.04595	0.202	.?...
CCND2	12	0.29171	0.81319	0.78721	NA	0.07992	11265	0.04599	0.202	...?.
HSD3BP4	1	0.58541	0.24975	0.56344	0.38062	0.19081	14669	0.04599	0.202	.....
OR8H2	11	0.73826	0.56444	0.82018	0.83117	0.03197	14669	0.04599	0.202	.....
LOC100652758	5	0.95804	0.70030	0.98901	0.49850	0.03596	14669	0.04602	0.202	.....
VMAC	19	0.35964	0.45255	0.57642	0.06394	0.50849	14669	0.04604	0.202	.....
LINC00667	18	0.20579	0.85315	0.41459	0.27872	0.25874	14669	0.04605	0.202	.....
IFNK	9	0.36164	0.38861	0.84216	0.28671	0.20979	14669	0.04606	0.202	.....
CMA1	14	0.46254	0.90210	0.59441	0.03497	0.42458	14669	0.04606	0.202	.....
OR11A1	6	0.07393	0.78322	0.57143	0.53946	0.20579	14669	0.04606	0.202	.....
SLC4A1AP	2	0.72328	0.40260	0.49650	0.41359	0.13287	14669	0.04607	0.202	.....
C1orf35	1	0.00470	0.57542	0.71928	0.48252	0.46154	14669	0.0461	0.202	.....
CDKL3	5	0.30270	0.55245	0.30569	NA	0.16484	11265	0.04611	0.202	...?.
LOC101927766	5	0.50549	0.74825	0.89111	0.43956	0.07892	14669	0.04614	0.202	.....
ARF3	12	0.17882	0.94605	0.62537	0.52947	0.12388	14669	0.04615	0.202	.....
DENND2D	1	0.05095	0.35165	0.29670	0.12887	0.82617	14669	0.04617	0.202	.....
OR5D14	11	0.46953	0.35365	0.18182	0.48452	0.23876	14669	0.04617	0.202	.....
TAF1A	1	0.11988	0.14785	0.05095	0.35065	0.77023	14669	0.04617	0.202	.....
LOC285629	5	0.69730	0.17582	0.15385	NA	0.20679	11265	0.04617	0.202	...?.
NRTN	19	0.38661	0.06394	0.96503	0.13087	0.49251	14669	0.04619	0.202	.....
SIX5	19	0.96903	0.27073	0.51149	0.60539	0.08092	14669	0.04622	0.202	.....
LINC00173	20	NA	NA	0.04296	0.22378	NA	4255	0.04623	0.202	??..?
ATP4A	19	0.37762	0.58042	0.23776	0.62238	0.15385	14669	0.04624	0.202	.....
SLC13A3	20	0.57742	0.69630	0.36863	0.24775	0.20080	14669	0.04625	0.202	.....
TMEM151B	6	0.92008	0.04296	0.51648	0.63536	0.15385	14669	0.04631	0.202	.....
TNFRSF25	1	0.79321	0.79221	0.99001	0.10989	0.14785	14669	0.04633	0.202	.....
ZNF184	6	0.34166	0.73726	0.75524	0.31868	0.15385	14669	0.04634	0.202	.....
DLG5	10	0.34965	0.31568	0.93207	NA	0.11189	11265	0.04634	0.202	...?.
KBTBD6	13	0.22478	0.46454	0.78122	0.42957	0.18581	14669	0.04634	0.202	.....
B4GALNT1	12	0.47053	0.47752	0.05594	0.46853	0.29471	14669	0.04635	0.202	.....
TMPRSS9	19	0.39860	0.07493	0.35664	0.44555	0.35764	14669	0.04635	0.202	.....
WDR41	5	0.24176	0.90609	0.88112	0.44755	0.10589	14669	0.04637	0.202	.....
MRPL23	11	0.42857	0.16983	0.15085	0.30569	0.45554	14669	0.04637	0.202	.....
BICD1	12	0.09191	0.55045	0.66733	NA	0.19281	11265	0.04641	0.202	...?.
TMEM165	4	0.85714	0.97403	0.54645	0.79520	0.02498	14669	0.04642	0.202	.....
PRELID3A	18	0.84515	0.32268	0.73427	0.44655	0.09690	14669	0.04643	0.202	.....
SGK223	8	0.03896	0.09890	0.34266	0.16284	0.97702	14669	0.04643	0.202	.....
PTPRK	6	NA	0.08991	0.29570	NA	0.20280	9841	0.04645	0.202	?..?.
LOC100507487	4	0.53247	0.28072	0.54745	0.60240	0.12887	14669	0.04646	0.202	.....
MMP17	12	0.59740	0.70230	0.06593	NA	0.15984	11265	0.04646	0.202	...?.
IFT172	2	0.11888	0.36064	0.42258	0.54945	0.28671	14669	0.0465	0.202	.....
LOC101929454	5	0.94306	0.22278	0.51149	NA	0.09091	11265	0.0465	0.202	...?.
SRPRB	3	0.00530	0.65534	0.07093	0.83716	0.52248	14669	0.04652	0.202	.....
AGXT	20	NA	0.32967	0.08991	0.10589	0.54046	13245	0.04653	0.202	?....
HDGFRP2	19	0.78322	0.96404	0.44555	0.16084	0.16184	14669	0.04654	0.202	.....
OR52I2	11	0.46054	0.53746	0.44555	0.06793	0.46653	14669	0.04655	0.202	.....
NT5C1B-RDH14	2	0.00230	0.37862	0.24875	0.76523	0.59640	14669	0.04657	0.202	.....
MCL1	1	0.44156	0.62737	0.32967	0.31369	0.22078	14669	0.04657	0.202	.....
FAM163A	1	0.11988	0.48452	0.59640	0.26973	0.36763	14669	0.04658	0.202	.....
DYNC1LI2	16	0.95005	0.79421	0.31469	0.05794	0.31169	14669	0.04658	0.202	.....
ATL3	11	0.56943	NA	0.39461	0.83916	0.05095	13691	0.04658	0.202	.?...
RNF41	12	0.04396	NA	0.79620	0.50150	0.21878	13691	0.04659	0.202	.?...
KRT7	12	0.85115	0.02997	0.03397	0.84316	0.30070	14669	0.04661	0.202	.....
EPT1	2	0.17982	0.66034	0.31568	0.08791	0.59341	14669	0.04662	0.202	.....
BBOF1	14	0.15285	1.00000	0.65834	NA	0.10689	11265	0.04664	0.202	.0.?.
LCLAT1	2	0.81618	0.16084	NA	NA	0.09291	10414	0.04665	0.202	..??.
LINC01338	5	0.52647	0.14885	0.42757	NA	0.18382	11265	0.04665	0.202	...?.
SLC35G2	3	0.39061	0.98901	0.13786	0.89011	0.07992	14669	0.04668	0.202	.....
ZNF675	19	0.15584	0.42657	0.21379	0.82418	0.20579	14669	0.0467	0.202	.....
CWF19L1	10	0.51049	0.26174	0.50450	0.22278	0.31269	14669	0.0467	0.202	.....
LOC101927354	7	0.36963	0.74426	0.67233	NA	0.08392	11265	0.0467	0.202	...?.
RIF1	2	0.95005	0.91508	0.68731	0.03696	0.25574	14669	0.04671	0.202	.....

IQSEC3	12	0.04396	0.18781	0.62637	NA	0.37363	11265	0.04676	0.202	...?.
LOC400867	21	0.86613	0.47153	0.36963	0.11489	0.29471	14669	0.04677	0.202	.....
CBLN2	18	0.37962	NA	0.06294	NA	0.20180	10287	0.04678	0.202	.?.?.
CYP46A1	14	0.49750	0.73027	0.19381	0.09890	0.43257	14669	0.04678	0.202	.....
LOC101929696	5	0.24476	0.54446	0.59441	0.43457	0.19181	14669	0.04679	0.202	.....
TMEM117	12	0.17982	0.75924	0.97602	NA	0.09391	11265	0.04679	0.202	...?.
SNX7	1	0.30370	0.46354	0.18182	0.85614	0.14785	14669	0.04679	0.202	.....
TMEM263	12	0.06793	0.99101	0.94805	0.71828	0.09690	14669	0.04681	0.202	.....
PGAP3	17	0.83816	0.06893	0.63536	0.30569	0.25175	14669	0.04682	0.202	.....
SPOP	17	0.59740	0.67832	0.62837	0.03297	0.43856	14669	0.04682	0.202	.....
ZNF148	3	0.01099	0.94905	0.12787	0.23576	0.74426	14669	0.04683	0.202	.....
DLX1	2	0.40759	0.07592	0.81319	0.04795	0.72328	14669	0.04685	0.202	.....
ZNF880	19	0.19880	0.92807	0.02597	0.18881	0.59341	14669	0.04686	0.202	.....
OR11H6	14	0.31169	0.18482	0.68032	0.18282	0.42557	14669	0.04687	0.202	.....
PLA2G16	11	0.44056	0.84316	0.55844	0.78621	0.05395	14669	0.04689	0.202	.....
NCBP2	3	0.66833	0.87912	0.15884	0.76324	0.07193	14669	0.0469	0.202	.....
L3MBTL3	6	0.17782	0.45155	0.19580	NA	0.26973	11265	0.0469	0.202	...?.
LPGAT1	1	0.05095	0.50150	0.64535	0.58941	0.25874	14669	0.0469	0.202	.....
GNB4	3	0.01340	0.60240	0.96104	0.32667	0.41858	14669	0.04691	0.202	.....
APRT	16	0.04595	0.44256	0.76424	NA	0.25075	11265	0.04693	0.203	...?.
TULP3	12	0.46753	0.68631	0.10789	0.86114	0.10889	14669	0.04693	0.203	.....
GPR45	2	NA	NA	NA	0.04695	NA	3404	0.04695	0.203	???.?
RNF149	2	0.67333	0.71429	0.04895	0.40759	0.22977	14669	0.04696	0.203	.....
CDKN2C	1	0.60040	0.37163	0.44655	0.45455	0.15584	14669	0.047	0.203	.....
C9orf142	9	NA	0.58442	0.49151	0.06793	NA	5233	0.04702	0.203	?...?
MEF2A	15	0.08392	0.66134	0.79620	0.08791	0.54246	14669	0.04703	0.203	.....
LOC101929066	8	0.03996	0.44256	0.30470	NA	0.37562	11265	0.04704	0.203	...?.
PNPLA5	22	0.25874	0.39161	0.13886	0.40659	0.37463	14669	0.04709	0.203	.....
OR3A2	17	0.05994	0.04096	0.40460	0.96204	NA	6657	0.04709	0.203	....?
LOC100129924	1	0.88611	0.87712	0.35465	0.23676	0.13387	14669	0.04711	0.203	.....
HBBP1	11	0.33666	0.06394	0.34166	0.08192	0.84915	14669	0.04711	0.203	.....
SERPINC1	1	0.28372	0.04895	0.54745	0.69730	0.27273	14669	0.04711	0.203	.....
IFFO1	12	0.68032	0.69930	0.01099	0.15884	0.52847	14669	0.04712	0.203	.....
CAMK1	3	0.35265	0.65135	0.46653	0.53946	0.13387	14669	0.04713	0.203	.....
FBXL14	12	0.43357	NA	0.19580	0.17682	0.33566	13691	0.04714	0.203	.?...
CD163	12	0.39660	0.29570	0.77822	0.04496	0.57243	14669	0.04714	0.203	.....
NET1	10	0.02597	0.94805	0.01299	0.79021	0.45854	14669	0.04716	0.203	.....
GPLD1	6	0.35265	0.30769	0.39161	NA	0.18482	11265	0.04716	0.203	...?.
PTCHD4	6	0.38362	0.46853	0.19580	NA	0.18981	11265	0.04718	0.203	...?.
GNAS	20	0.76823	0.89710	0.33866	0.39560	0.09890	14669	0.0472	0.203	.....
KRTAP10-9	21	0.31169	0.49650	0.12388	0.69630	0.20579	14669	0.0472	0.203	.....
ATP2A1	16	0.39660	0.62537	0.20979	0.07592	0.55445	14669	0.04725	0.203	.....
HADH	4	0.85415	0.70030	0.40759	0.10589	0.25075	14669	0.04727	0.203	.....
LINC01346	1	0.74625	0.08192	0.24975	0.04895	0.77822	14669	0.04727	0.203	.....
HSD3B2	1	0.83217	0.70829	0.05694	0.51149	0.15684	14669	0.04728	0.203	.....
CCT6P1	7	0.86214	0.16583	0.95904	0.66234	0.06793	14669	0.04729	0.203	.....
CRISP1	6	0.02098	0.18681	0.15085	0.30769	0.90709	14669	0.04729	0.203	.....
LGALS17A	19	0.72128	0.28571	0.75724	0.13686	0.27572	14669	0.04732	0.203	.....
TRIM35	8	0.86414	0.52747	0.02398	NA	0.18581	11265	0.04733	0.203	...?.
ZBTB11-AS1	3	0.44755	0.40959	0.10889	0.68132	0.19880	14669	0.04735	0.203	.....
AHR	7	0.04995	0.42957	0.65035	NA	0.27073	11265	0.04735	0.203	...?.
DISC1	1	0.52747	0.85215	0.15385	NA	0.11988	11265	0.04735	0.203	...?.
OSR1	2	0.25974	NA	0.05495	0.27473	0.43556	13691	0.04736	0.203	.?...
JADE1	4	0.09990	0.12587	0.77023	0.30270	0.49151	14669	0.04736	0.203	.....
GOLGA4	3	0.32567	0.38062	0.07792	0.56144	0.31269	14669	0.04736	0.203	.....
ARC	8	0.23177	NA	0.19281	0.24076	0.36863	13691	0.04736	0.203	.?...
CCDC64B	16	0.15884	0.18182	0.45155	0.44755	0.37762	14669	0.04737	0.203	.....
MSL2	3	0.36264	0.62737	0.59840	NA	0.10390	11265	0.04737	0.203	...?.
PNO1	2	0.34366	0.72527	0.63437	0.52348	0.11189	14669	0.04741	0.203	.....
TNFSF13B	13	0.17582	0.06094	0.49550	0.49650	0.42857	14669	0.04743	0.203	.....
SSR4P1	21	0.29471	0.04895	0.42657	0.55844	0.36164	14669	0.04743	0.203	.....
BCL2L12	19	0.77822	NA	0.44855	0.34366	0.10789	13691	0.0475	0.203	.?...
SLC39A9	14	0.07592	0.11189	0.18282	NA	0.54046	11265	0.0475	0.203	...?.
LOC645553	19	0.73127	0.21778	0.20779	0.92108	0.10290	14669	0.04751	0.203	.....
ID2-AS1	2	0.51548	NA	0.67333	0.46354	0.08891	13691	0.04751	0.203	.?...
PTCRA	6	0.01299	0.36464	0.87313	NA	0.35065	11265	0.04755	0.203	...?.
ADTRP	6	0.15385	0.14885	0.56643	NA	0.29071	11265	0.04759	0.203	...?.
MLST8	16	0.49850	NA	0.44056	0.16883	0.24775	13691	0.04761	0.203	.?...
MIIP	1	0.84216	0.19980	0.57343	0.70430	0.08591	14669	0.04762	0.203	.....

LINC00313	21	0.06294	0.80220	0.27473	0.66533	0.24176	14669	0.04763	0.203	.....
RALB	2	0.51049	0.51349	0.35564	0.32567	0.21778	14669	0.04764	0.203	.....
COL22A1	8	0.63736	0.28272	0.19680	NA	0.17582	11265	0.04764	0.203	...?.
MTUS2	13	0.06194	0.80919	0.23077	NA	NA	3253	0.04766	0.203	...??
SKINT1L	1	0.94805	0.88212	0.18082	0.00390	0.77722	14669	0.04766	0.203	.....
CD38	4	0.12288	0.63337	0.59441	0.13786	0.47053	14669	0.04768	0.203	.....
CDH17	8	0.38861	0.88711	0.75225	NA	0.06693	11265	0.04768	0.203	...?.
CYP2C19	10	0.01099	0.25674	0.47852	NA	0.51648	11265	0.04768	0.203	...?.
HIGD1A	3	0.42458	0.37562	0.58941	0.08591	0.46054	14669	0.04768	0.203	.....
CNBD2	20	0.77123	0.72028	0.72727	0.25275	0.11588	14669	0.0477	0.203	.....
EDN1	6	0.49850	0.99600	0.50450	0.00223	0.85614	14669	0.04771	0.203	.....
NFS1	20	0.80819	0.48751	0.36264	0.70829	0.07293	14669	0.04773	0.203	.....
LINC01335	5	0.97403	0.32667	0.02298	0.51149	0.24875	14669	0.04773	0.203	.....
MMP15	16	0.13387	0.05095	0.08791	0.89810	0.46853	14669	0.04776	0.203	.....
ORM2	9	0.71828	0.58242	0.11888	0.55045	0.15085	14669	0.04778	0.203	.....
ABCC8	11	0.51648	0.85215	0.74825	NA	0.05694	11265	0.04778	0.203	...?.
LOC645967	6	0.18282	0.31469	0.35764	0.26773	0.45754	14669	0.0478	0.203	.....
TRAIP	3	0.43357	0.82218	0.77023	0.19481	0.18382	14669	0.0478	0.203	.....
GUCY2C	12	0.16484	0.54046	0.98901	NA	0.12288	11265	0.0478	0.203	...?.
KLHDC10	7	0.86414	0.24276	0.41059	0.12288	0.35564	14669	0.04782	0.203	.....
SMARCA5	4	0.19780	0.23177	0.88212	0.22278	NA	6657	0.04784	0.203	....?
FOLH1	11	0.82817	0.21079	0.40260	0.40959	0.18082	14669	0.04786	0.203	.....
LOC494127	9	0.84815	0.50949	0.50749	NA	0.06893	11265	0.04787	0.203	...?.
C17orf51	17	0.60539	0.30070	0.01898	0.50749	0.36763	14669	0.04789	0.203	.....
GIPR	19	0.28272	0.92707	0.92308	0.58641	0.07093	14669	0.04789	0.203	.....
LINC00207	22	0.84116	NA	0.07692	0.75624	0.08492	13691	0.04789	0.203	.?...
ADORA1	1	0.44955	0.73227	0.08292	0.29371	0.32268	14669	0.0479	0.203	.....
ZNF117	7	0.94905	0.08591	0.86913	NA	0.09590	11265	0.0479	0.203	...?.
YPEL2	17	0.50749	0.65734	0.14885	0.41259	0.21578	14669	0.0479	0.203	.....
TRIP13	5	0.07393	0.55245	0.93407	0.02198	0.84515	14669	0.04793	0.203	.....
LRP1	12	0.25375	0.36464	0.32967	0.24875	0.41858	14669	0.04794	0.203	.....
C15orf53	15	0.77922	0.05794	0.08392	0.83716	0.23077	14669	0.04794	0.203	.....
PGM5P4-AS1	2	0.04795	NA	NA	NA	NA	1424	0.04795	0.203	.????
CYB5R2	11	0.99401	0.97003	0.52248	NA	0.03397	11265	0.04795	0.203	...?.
ARFGAP3	22	0.25475	0.39660	0.37063	0.10789	0.57542	14669	0.04795	0.203	.....
GTDC1	2	NA	0.54046	0.21978	0.55345	0.12288	13245	0.04796	0.203	?....
KRT4	12	0.53846	0.57343	0.25974	0.66234	0.11688	14669	0.04797	0.203	.....
TPPP3	16	0.45455	0.15485	0.75325	0.68332	0.13487	14669	0.04798	0.203	.....
ZNF576	19	0.98202	0.12088	0.22178	0.36563	0.25574	14669	0.04798	0.203	.....
RHBDD3	22	0.03896	0.43956	0.10689	0.18781	0.88012	14669	0.04803	0.204	.....
SMPDL3A	6	0.73227	0.02398	0.35664	0.53247	0.30070	14669	0.04803	0.204	.....
HOTAIR	12	0.25774	0.98601	0.69530	0.28871	0.16983	14669	0.04803	0.204	.....
FAM182A	20	0.82018	0.20480	0.07692	0.21479	0.45255	14669	0.04804	0.204	.....
C10orf120	10	0.49550	0.52947	0.81419	0.44555	0.11089	14669	0.04804	0.204	.....
LECT2	5	0.79221	0.60440	0.48152	0.62238	0.06693	14669	0.04808	0.204	.....
44447	20	NA	NA	NA	NA	NA	14669	0.04809	0.204	.....
PTPN22	1	0.23477	0.98302	0.01998	0.68831	0.25175	14669	0.04811	0.204	.....
HADHA	2	0.02797	0.62837	0.29970	0.39660	0.49151	14669	0.04813	0.204	.....
MDM4	1	0.13586	0.54545	0.09391	0.86913	0.23776	14669	0.04814	0.204	.....
PACRGL	4	0.50450	0.66434	0.33467	0.26573	0.22977	14669	0.04815	0.204	.....
TRIM61	4	0.47353	0.40859	0.68831	0.19181	0.27073	14669	0.04815	0.204	.....
TP53RK	20	0.89211	0.33866	0.10889	0.73926	0.12288	14669	0.04815	0.204	.....
KRTAP8-1	21	0.13087	NA	0.08791	0.54046	0.32168	13691	0.04816	0.204	.?...
ABHD13	13	0.61638	0.76923	0.52048	0.94905	0.03497	14669	0.04817	0.204	.....
LOC100506422	9	0.48452	0.80320	0.15984	NA	0.13287	11265	0.04818	0.204	...?.
GHRLOS	3	0.53147	0.12987	0.04695	0.51548	0.41858	14669	0.04819	0.204	.....
TMEM64	8	0.57842	0.00170	0.92408	NA	0.32667	11265	0.0482	0.204	...?.
SLC28A1	15	0.10889	0.47952	0.84715	NA	0.17483	11265	0.0482	0.204	...?.
LZTR1	22	0.46853	0.88012	0.39860	0.64535	0.08192	14669	0.04821	0.204	.....
RUNDC3A-AS1	17	0.96204	0.58541	0.03596	0.07393	0.53946	14669	0.04824	0.204	.....
ENDOV	17	0.31369	0.83417	0.19281	0.13786	0.43357	14669	0.04825	0.204	.....
CUL7	6	0.15085	0.83916	0.78222	0.09291	0.41758	14669	0.04826	0.204	.....
ODAM	4	0.09291	0.09590	0.96603	0.45754	0.37662	14669	0.04827	0.204	.....
NR1I2	3	0.13387	0.39660	0.15984	0.39061	0.46753	14669	0.04827	0.204	.....
MFN1	3	0.00490	0.77423	0.81119	0.76224	0.26673	14669	0.04829	0.204	.....
MARCKS	6	0.72028	0.37163	0.59141	0.18681	0.24076	14669	0.04833	0.204	.....
CHCHD2	7	0.95305	0.30569	0.58541	0.91808	0.04096	14669	0.04834	0.204	.....
LOC101929288	20	0.02597	NA	0.97902	0.28272	0.35065	13691	0.04835	0.204	.?...
LOC100129931	4	0.52647	0.73127	0.03297	0.19580	0.45554	14669	0.04835	0.204	.....

PGAM2	7	0.13287	0.50749	0.42258	0.09790	0.64136	14669	0.04837	0.204	.....
OCLN	20	NA	0.21479	0.50050	NA	0.12488	9841	0.0484	0.204	?..?.
SYNE4	19	0.27173	NA	0.52647	0.00830	0.85015	13691	0.04848	0.204	.?...
LFNG	7	0.59640	0.45954	0.17483	0.57043	0.16883	14669	0.04849	0.204	.....
TBX18-AS1	6	0.94006	0.45255	0.25275	0.77023	0.06993	14669	0.0485	0.204	.....
LOC151174	20	NA	0.39860	0.00560	0.35265	0.46354	13245	0.0485	0.204	?....
TCERG1	5	0.28571	0.88312	0.15085	0.63337	0.16484	14669	0.04851	0.204	.....
VWA3B	2	0.65135	0.71029	0.55844	0.16284	0.21179	14669	0.04852	0.204	.....
JAKMIP2-AS1	5	0.63237	0.06494	0.63836	0.24176	0.36464	14669	0.04853	0.204	.....
TAF1D	11	0.87612	0.58841	0.28272	NA	0.08292	11265	0.04855	0.204	...?.
PADI1	1	0.91808	0.27373	0.71329	0.02198	0.52847	14669	0.04855	0.204	.....
AQP9	15	0.46454	0.90709	0.55944	NA	0.07193	11265	0.04855	0.204	...?.
ATP13A2	1	0.61738	0.65235	0.15784	0.13187	0.39061	14669	0.04856	0.204	.....
CNRIP1	2	0.80220	0.01430	0.60839	0.56044	0.24575	14669	0.04858	0.204	.....
PRSS16	6	0.05395	0.18482	0.79820	0.58242	0.34166	14669	0.0486	0.204	.....
PIP5K1A	1	0.71229	1.00000	0.90909	0.05295	0.22478	14669	0.04863	0.204	.0...
ART5	11	0.10290	NA	0.31269	0.18282	0.48851	13691	0.04863	0.204	.?...
SLC35E2B	20	NA	NA	0.19381	NA	0.09890	8863	0.04865	0.204	??.?.
DDIT3	12	0.05095	NA	0.49051	0.10490	0.63636	13691	0.04865	0.204	.?...
C5AR2	19	0.67233	NA	0.51948	0.09590	0.26973	13691	0.04866	0.204	.?...
LINC00378	13	0.19181	0.53946	0.11189	0.20180	0.58042	14669	0.04867	0.204	.....
PCDHGA2	5	0.91209	0.07293	0.27273	0.28971	0.34366	14669	0.04867	0.204	.....
RSPO3	6	0.48052	0.46953	0.06893	0.80819	0.17183	14669	0.04868	0.204	.....
NPAS3	14	0.57742	0.36064	0.57143	NA	0.10989	11265	0.0487	0.204	...?.
ACADL	20	NA	0.85814	0.45355	0.06494	0.30569	13245	0.04874	0.204	?....
ADCK4	19	0.97502	0.11788	0.11788	0.14885	0.51149	14669	0.04875	0.204	.....
METTL11B	1	0.68032	0.06294	0.13886	0.04196	0.97203	14669	0.04875	0.204	.....
IL17C	16	0.03097	NA	0.28172	NA	0.33067	10287	0.04875	0.204	.?.?.
MICAL1	6	0.47552	0.13886	0.98901	0.36763	0.20879	14669	0.04876	0.204	.....
FGF3	11	0.31768	0.59041	0.68032	0.66533	0.10090	14669	0.0488	0.205	.....
CD4	12	0.75225	0.77522	0.56743	0.38062	0.09391	14669	0.04882	0.205	.....
ZNF101	19	0.26873	0.02498	0.83816	0.37063	0.44356	14669	0.04882	0.205	.....
PRDM12	9	0.52148	0.91808	0.23876	0.19181	0.26074	14669	0.04884	0.205	.....
MYL6	12	0.03197	NA	0.19281	0.26673	0.60140	13691	0.04886	0.205	.?...
CMKLR1	12	0.24476	0.40060	0.09690	0.10190	0.78422	14669	0.04888	0.205	.....
MDK	11	0.15085	0.70629	0.15085	0.18581	0.55045	14669	0.04889	0.205	.....
UROC1	3	0.95305	0.30669	0.33067	0.32867	0.18382	14669	0.04891	0.205	.....
PSME3	17	0.00880	NA	NA	0.51548	NA	4828	0.04897	0.205	.??.?
LOC101927131	16	0.57143	0.65834	0.34765	0.31169	0.19181	14669	0.04899	0.205	.....
FBXO34	14	0.97203	0.95205	0.58741	0.12088	0.14985	14669	0.049	0.205	.....
OSTC	4	0.24476	0.43057	0.19780	0.47053	0.31369	14669	0.04903	0.205	.....
PIEZO2	18	0.49151	0.13187	0.77023	0.19780	0.36164	14669	0.04903	0.205	.....
NAA38	17	0.01499	0.18082	0.55045	0.21578	0.86613	14669	0.04905	0.205	.....
AIM1	6	0.07193	0.60639	0.05894	0.56244	0.46454	14669	0.04905	0.205	.....
DHX30	3	0.71828	0.06693	0.61239	0.14386	0.45155	14669	0.04907	0.205	.....
SELT	3	0.10390	0.15784	0.63636	0.20280	0.61738	14669	0.04907	0.205	.....
GGNBP2	17	0.82318	0.19680	0.28571	0.70929	0.12687	14669	0.04909	0.205	.....
NRXN2	11	0.79221	0.02398	0.38561	0.65435	0.23477	14669	0.0491	0.205	.....
SIM2	21	0.47453	0.67532	0.78422	0.18981	0.20380	14669	0.0491	0.205	.....
CCS	11	0.40260	0.09391	0.89211	0.34565	0.28671	14669	0.04911	0.205	.....
WDFY4	10	0.87013	0.47253	0.46454	NA	0.07592	11265	0.04911	0.205	...?.
CTNND1	11	0.10290	0.38761	0.86114	0.38162	0.29570	14669	0.04911	0.205	.....
DISC1FP1	11	NA	0.35664	0.05794	NA	NA	1829	0.04913	0.205	?..??
SEMG1	20	0.56743	0.46154	0.69131	0.32967	0.16184	14669	0.04914	0.205	.....
SLC26A6	3	0.66833	0.05994	0.96404	0.31069	0.25075	14669	0.04915	0.205	.....
FOXA2	20	0.06893	0.13686	0.13886	0.49051	0.65734	14669	0.04915	0.205	.....
HSPB7	1	0.63636	0.03297	0.60040	0.17582	0.50649	14669	0.04916	0.205	.....
CDC42BPG	11	NA	0.49950	0.36763	0.44256	0.13387	13245	0.04918	0.205	?....
LOC283045	10	0.52747	0.04995	0.16983	NA	0.35065	11265	0.04922	0.205	...?.
GOT2	16	0.29071	0.67932	0.13886	0.51149	0.24575	14669	0.04924	0.205	.....
SERPINI2	3	0.73626	0.34066	0.04296	0.24575	0.44156	14669	0.04925	0.205	.....
FITM2	20	0.21079	0.15085	0.03097	0.75524	0.46853	14669	0.04926	0.205	.....
C2orf48	2	NA	0.81019	0.66134	0.74126	0.03696	13245	0.04927	0.205	?....
ATG14	14	0.98901	0.82318	0.57443	0.09590	0.19081	14669	0.04928	0.205	.....
NOTCH1	9	0.73127	0.62038	0.03996	0.06494	0.63836	14669	0.04928	0.205	.....
HARS	5	0.06494	0.20779	0.05295	0.98501	0.42458	14669	0.04934	0.205	.....
KIAA1191	5	0.60440	0.50649	0.54146	0.42957	0.13487	14669	0.04935	0.205	.....
SYCP1	1	0.40759	0.16284	0.87812	0.03996	0.66434	14669	0.04935	0.205	.....
SLC25A35	17	0.12687	0.43357	0.73327	NA	0.19281	11265	0.04936	0.205	...?.

FNTA	8	0.63337	0.30370	0.89211	0.06693	0.38961	14669	0.04939	0.205	.....
LINCMD1	6	0.95604	0.70829	0.59940	NA	0.04496	11265	0.04939	0.205	...?.
PLEKHB1	11	0.26074	0.21978	0.90110	0.49251	0.20480	14669	0.04942	0.205	.....
LINC00955	4	0.89411	NA	0.82018	0.03796	0.27772	13691	0.04944	0.205	.?...
FAM172BP	3	0.68032	0.17582	0.03696	0.35065	0.46953	14669	0.04947	0.205	.....
DONSON	21	0.85614	0.15085	0.44356	0.76024	0.10290	14669	0.04949	0.205	.....
MGST2	4	0.15684	0.97203	0.72727	0.73227	0.08691	14669	0.04951	0.205	.....
LOC102723427	7	0.11289	0.95604	0.37263	NA	0.17682	11265	0.04953	0.205	...?.
POLE4	2	0.04795	0.66633	0.01199	0.54845	0.64336	14669	0.04953	0.205	.....
GIPC2	1	0.88012	0.81618	0.80420	0.05794	0.23177	14669	0.04954	0.205	.....
GADD45B	20	NA	NA	0.18482	0.57842	0.10190	12267	0.04954	0.205	??...
C9orf173-AS1	20	NA	NA	0.46154	NA	0.06793	8863	0.04956	0.205	??.?.
ZACN	17	0.54545	NA	0.73427	0.15584	0.20180	13691	0.04956	0.205	.?...
FAM161B	14	0.68631	0.27772	0.34166	NA	0.14186	11265	0.04958	0.205	...?.
VAX1	10	0.52048	NA	0.89111	0.06993	0.28472	13691	0.04959	0.205	.?...
NPR2	9	0.16583	0.56144	0.55844	0.54845	0.19780	14669	0.04959	0.205	.....
CXCL12	10	0.83816	0.46753	0.91908	0.09590	0.23077	14669	0.0496	0.205	.....
CHD3	17	0.00999	0.49750	0.92108	0.04695	0.99500	14669	0.0496	0.205	.....
ITGB2	21	0.56244	0.08292	0.49351	0.52148	0.24376	14669	0.0496	0.205	.....
ZNF506	19	0.74825	0.09391	0.32567	0.05095	0.73127	14669	0.04961	0.205	.....
UTP4	16	0.18581	0.92008	0.14685	0.25075	0.39960	14669	0.04961	0.205	.....
MSANTD3-TMEF	9	0.47153	0.42458	0.03397	0.10989	0.73327	14669	0.04962	0.205	.....
PDIA5	3	0.87912	0.57443	0.52348	0.75724	0.04695	14669	0.04962	0.205	.....
LRRC37A6P	10	0.09690	0.29870	0.23377	NA	0.38062	11265	0.04963	0.205	...?.
EFNA5	5	0.35465	0.01060	0.58142	0.45554	0.47952	14669	0.04964	0.205	.....
C1orf94	1	0.19381	0.71129	0.23776	0.16883	0.48551	14669	0.04964	0.205	.....
OR6Y1	1	0.34665	0.20879	0.65634	0.82917	0.12488	14669	0.04964	0.205	.....
MYOCD	17	0.72128	0.36064	0.49750	0.82218	0.06893	14669	0.04964	0.205	.....
GBGT1	9	0.81419	0.31469	0.36264	0.19680	0.28571	14669	0.04966	0.205	.....
KLHDC8A	1	0.92507	0.82218	0.70529	0.26673	0.09091	14669	0.04966	0.205	.....
P2RX1	17	0.48651	0.87712	0.02198	0.61339	0.21778	14669	0.04968	0.205	.....
C16orf71	16	0.66034	0.70729	0.20679	0.09590	0.39960	14669	0.0497	0.205	.....
HLA-J	6	0.95504	0.67632	0.30370	0.25774	0.15784	14669	0.04975	0.206	.....
ARF1	1	0.11189	0.16983	0.91009	0.85315	0.17782	14669	0.04976	0.206	.....
HOXA11-AS	7	0.15485	NA	0.84615	0.37962	0.18382	13691	0.04976	0.206	.?...
TRRAP	7	0.04895	0.70230	0.31269	0.26973	0.52048	14669	0.04977	0.206	.....
NPY2R	4	0.17782	0.06793	0.31069	0.50749	0.49251	14669	0.04978	0.206	.....
PLD1	3	0.12887	0.48152	0.89610	0.98302	0.08691	14669	0.04979	0.206	.....
BHLHE22	8	0.38062	0.91109	0.43856	NA	0.09590	11265	0.04979	0.206	...?.
FMO2	1	0.18082	0.98302	0.58042	0.93906	0.06394	14669	0.04981	0.206	.....
PVT1	8	0.42458	0.39061	0.97303	0.14685	0.28871	14669	0.04982	0.206	.....
C1orf198	1	0.91908	0.81518	0.13686	0.01998	0.59740	14669	0.04983	0.206	.....
C2orf69	2	0.32368	0.32567	0.00899	0.32068	0.69231	14669	0.04984	0.206	.....
ANKRD1	10	0.40460	0.44356	0.08791	0.04595	0.85914	14669	0.04986	0.206	.....
RABGEF1	7	0.90609	0.45255	0.68531	NA	0.05994	11265	0.04986	0.206	...?.
MPP6	7	0.94106	0.17582	0.71129	0.19181	0.23876	14669	0.04986	0.206	.....
PLLP	16	0.54046	0.72328	0.80919	0.05994	0.33367	14669	0.04988	0.206	.....
MED30	8	0.03297	0.64036	0.28272	0.30569	0.56643	14669	0.04989	0.206	.....
FCRL4	1	0.26474	0.48052	0.69431	0.28272	0.26773	14669	0.04989	0.206	.....
SCNN1A	12	0.22877	0.44256	0.15884	NA	0.27572	11265	0.04992	0.206	...?.
PSMC6	14	0.43856	0.39660	0.04995	NA	0.28771	11265	0.04992	0.206	...?.
MSMB	10	0.69630	0.66134	0.80619	NA	0.05395	11265	0.04992	0.206	...?.
FECH	18	0.53846	0.41558	0.29071	NA	0.15085	11265	0.04994	0.206	...?.
SNX29P1	20	NA	NA	0.04995	NA	NA	851	0.04995	0.206	??.??
SSBP3	1	0.04396	0.26074	0.56444	0.79121	0.29371	14669	0.04995	0.206	.....
LOC100996263	1	0.05594	0.38761	0.16983	0.80120	0.35065	14669	0.04996	0.206	.....
NOP10	15	0.34366	0.45155	0.13087	0.96004	0.14286	14669	0.04996	0.206	.....
USP4	3	0.44456	0.52048	0.51449	0.78122	0.08691	14669	0.04998	0.206	.....
FCRL5	1	0.21179	0.39061	0.88312	0.43157	0.20579	14669	0.04999	0.206	.....

Table S3. *FMNL2* x factor associated with LOAD.

Gene	factor	ROSMAP	WHICAP African-Americans	WHICAP Whites	Caribbean Hispanics	NACC	Meta-analysis
		p	p	p	p	p	P
FMNL2	PC1+PC2	0.010	0.623	0.157	0.089	0.044	3.47E-04
FMNL2	Hypertension	0.016	0.869	0.057	0.137	0.058	7.53E-04
FMNL2	Bmi	0.096	0.491	0.051	0.284	0.07	2.51E-03
FMNL2	Heart	0.145	0.098	0.945	0.132	0.787	1.44E-02
FMNL2	Diabetes	0.268	0.488	0.131	0.189	0.21	6.82E-02

Table S4. Top SNP x CVRF interactions (p<0.05) in *FMNL2* associated with LOAD

							ROSMAP	WHICAP African-Americans	WHICAP Whites	Caribbean Hispanics	NACC	Meta-analysis			
snp	chr	bp	A1	A2	MinFreq	MaxFreq	p	p	p	p	p	b	SE	p	Direction
rs57223657	2	153403659	t	g	0.0449	0.0722	0.0738	NA	0.3833	0.0148	0.0389	0.168	0.0471	0.000361	+?+++
rs6760139	2	153388019	g	c	0.5135	0.7246	0.5498	0.9096	0.9002	0.0003	0.0157	0.0752	0.0211	0.000363	-++++
rs11892232	2	153358329	a	g	0.2766	0.3472	0.4620	0.2084	0.2843	0.0069	0.0120	0.0704	0.0222	0.001535	-+-++
rs11893683	2	153359260	t	g	0.2769	0.3472	0.4678	0.2139	0.2843	0.0074	0.0117	0.0703	0.0223	0.001578	-+-++
rs72864990	2	153371385	a	g	0.0454	0.0712	0.1072	NA	0.7846	0.0065	0.1288	0.1486	0.0478	0.001875	+?+++
rs2881336	2	153351548	t	c	0.284	0.391	0.4612	0.1978	0.2655	0.0042	0.0210	0.0671	0.0217	0.002003	-+-++
rs10173398	2	153354994	g	a	0.6516	0.7053	0.4538	0.7147	0.2843	0.0015	0.0110	-0.0677	0.0221	0.002168	+++--
rs10172230	2	153398476	c	a	0.2777	0.4246	0.6638	0.4457	0.4393	0.1325	0.0180	0.0629	0.021	0.002748	+++++
rs10165830	2	153399629	t	c	0.3849	0.4235	0.7782	0.6016	0.3983	0.1376	0.0132	0.0621	0.0208	0.002832	+++++
rs12617686	2	153351829	t	c	0.3065	0.3916	0.4625	0.9898	0.2655	0.0020	0.0207	0.0635	0.0217	0.003425	-+-++
rs1155779	2	153405594	g	a	0.3989	0.499	0.2927	0.3373	0.1950	0.0000	0.3713	0.0601	0.0206	0.003501	-++++
rs2463	2	153505376	c	t	0.0134	0.0424	0.0578	NA	0.9381	NA	0.0142	0.2561	0.0909	0.004831	+-+-+
rs4471845	2	153334144	g	t	0.7082	0.7505	0.0837	0.9105	0.2234	0.0029	0.0121	-0.0636	0.0231	0.00594	+++--
rs6737006	2	153368175	t	a	0.2926	0.3901	0.1952	0.5209	0.2444	0.0047	0.0245	0.0598	0.0219	0.006176	-+-++
rs2346204	2	153332159	c	a	0.7071	0.7484	0.1205	0.9506	0.2568	0.0038	0.0134	-0.0632	0.0231	0.006178	+++--
rs10166703	2	153323026	a	g	0.2523	0.2944	0.1146	0.9877	0.2487	0.0031	0.0157	0.0631	0.0231	0.006246	-+-++
rs4613221	2	153334141	g	a	0.6991	0.7507	0.0830	0.9540	0.2234	0.0035	0.0121	-0.0629	0.023	0.006327	+++--
rs75795961	2	153388742	g	a	0.9432	0.9784	0.0878	0.6546	0.6124	0.9938	0.0128	0.1288	0.0477	0.006887	+++++
rs9288103	2	153323912	c	g	0.7072	0.7477	0.1146	0.9352	0.2526	0.0037	0.0152	-0.0622	0.0231	0.007002	+++--
rs2346181	2	153388779	g	a	0.5885	0.8998	0.0212	0.5105	0.1608	0.0157	0.3252	0.0759	0.0284	0.007482	+-+++
rs6434078	2	153368897	c	t	0.5047	0.8717	0.0096	0.2690	0.1188	0.0190	0.9029	0.0694	0.026	0.007527	+++++
rs79368258	2	153388743	t	c	0.9432	0.9779	0.1049	0.7051	0.6124	0.9938	0.0128	0.1271	0.0476	0.007588	+++++
rs6736639	2	153367861	g	a	0.2918	0.3901	0.1952	0.4388	0.2444	0.0081	0.0255	0.0582	0.0218	0.007639	-+-++
rs11892859	2	153337038	a	g	0.6944	0.7477	0.1084	0.7912	0.2596	0.0044	0.0135	-0.061	0.023	0.007977	+++--
rs10195380	2	153342552	c	g	0.2852	0.3844	0.3341	0.8121	0.3273	0.0059	0.0307	0.0576	0.0218	0.008138	-+-++
rs7590079	2	153369079	g	a	0.2902	0.3906	0.2073	0.4971	0.2428	0.0140	0.0188	0.0578	0.022	0.008595	-+-++
rs75239367	2	153282016	g	t	0.0161	0.0259	0.9223	NA	0.6372	0.0330	0.0424	0.1898	0.0727	0.008997	+--++
rs10432494	2	153315522	a	g	0.2456	0.2973	0.0428	0.8859	0.1729	0.0031	0.0178	0.0598	0.0232	0.01	-+-++
rs6434059	2	153349165	c	t	0.3356	0.4489	0.1332	0.8523	0.1450	0.1188	0.0022	0.0528	0.0213	0.01298	-+-++
rs6434061	2	153349283	a	g	0.3358	0.4489	0.1261	0.8523	0.1450	0.1188	0.0022	0.0527	0.0213	0.01326	-+-++
rs6434060	2	153349236	a	c	0.336	0.4489	0.1257	0.8523	0.1450	0.1188	0.0022	0.0527	0.0213	0.01328	-+-++
rs59965317	2	153332134	g	t	0.7685	0.9148	0.1504	0.9035	0.2306	0.0600	0.2012	0.08	0.0323	0.0133	+-+++
rs7580023	2	153334971	c	g	0.8036	0.9528	0.0345	0.4919	0.4004	0.0000	0.0655	-0.0702	0.0284	0.01336	+++--
rs6434062	2	153349338	c	a	0.3358	0.4489	0.1261	0.8523	0.1344	0.1188	0.0022	0.0526	0.0213	0.01346	-+-++
rs12614608	2	153331546	a	g	0.2787	0.3306	0.1123	0.9933	0.2730	0.0062	0.0270	0.055	0.0223	0.01382	-+-++
rs16831171	2	153333501	a	t	0.8036	0.9528	0.0312	0.4753	0.4004	0.0001	0.0678	-0.0693	0.0284	0.01459	+++--
rs57173254	2	153434711	c	t	0.5419	0.8721	0.3349	0.0150	0.3730	NA	0.1909	0.0803	0.033	0.01501	+++-+
rs893331	2	153394396	t	c	0.3503	0.4152	0.2057	0.5283	0.2592	0.3689	0.0978	0.0502	0.0208	0.01583	+++++
rs7591606	2	153373875	c	t	0.7374	0.8971	0.0652	0.4143	0.2455	0.0059	0.5626	0.0714	0.0296	0.01585	+-+++
rs7425826	2	153338538	a	g	0.669	0.7335	0.0965	0.8500	0.2763	0.0059	0.0270	-0.054	0.0224	0.01598	+++--
rs2346183	2	153389732	a	g	0.7613	0.8631	0.0253	0.1436	0.2972	0.0082	0.0279	0.0605	0.0251	0.01616	-+-++
rs11892222	2	153436469	t	a	0.5413	0.8723	0.3026	0.0216	0.3856	NA	0.1909	0.0792	0.033	0.01651	+++-+
rs10180234	2	153387379	a	g	0.5925	0.8985	0.0327	0.3747	0.1672	0.0163	0.4600	0.0679	0.0284	0.01659	+-+++
rs6717982	2	153388190	g	a	0.5869	0.8985	0.0327	0.4083	0.1672	0.0178	0.4600	0.0677	0.0283	0.01668	+-+++
rs13010632	2	153257756	t	c	0.7543	0.79	0.2305	0.2211	0.1146	0.0110	0.0099	-0.0586	0.0245	0.01696	+++--
rs13029407	2	153257866	c	t	0.7543	0.79	0.2305	0.2211	0.1146	0.0110	0.0099	-0.0586	0.0245	0.01696	+++--
rs10207275	2	153387764	g	c	0.5867	0.8985	0.0327	0.3809	0.1672	0.0177	0.4600	0.0673	0.0283	0.01738	+-+++
rs2068886	2	153339823	t	c	0.6692	0.7201	0.0965	0.9084	0.2831	0.0074	0.0281	-0.0531	0.0223	0.01755	+++--
rs6720532	2	153387852	c	t	0.5867	0.8985	0.0327	0.3809	0.1672	0.0184	0.4600	0.067	0.0283	0.01782	+-+++
rs2577182	2	153393747	t	c	0.3447	0.3968	0.1633	0.3596	0.3407	0.2741	0.1804	0.0492	0.0209	0.01868	+++++
rs11686482	2	153377839	g	c	0.7607	0.8655	0.0182	0.1774	0.3493	0.0079	0.0299	0.059	0.0252	0.01913	-+-++
rs7596408	2	153473435	a	g	0.3465	0.4929	0.7051	0.4695	0.6580	0.0077	0.1832	0.0489	0.0209	0.01913	-+-++
rs16823809	2	153439057	c	g	0.539	0.8734	0.3036	0.0248	0.3931	NA	0.2001	0.0775	0.0332	0.01953	+++?+
rs34047327	2	153273911	g	c	0.7821	0.8227	0.1891	0.7372	0.4320	0.0066	0.0475	-0.0609	0.0261	0.01958	+++--
rs76719918	2	153492611	t	c	0.877	0.9538	0.2678	NA	0.7362	0.0072	0.0587	0.0828	0.0355	0.01958	-?+++
rs16823813	2	153439698	c	t	0.5385	0.8738	0.2816	0.0262	0.3894	NA	0.2055	0.0773	0.0332	0.01986	+++-+
rs58383962	2	153439145	c	t	0.5385	0.8734	0.3036	0.0261	0.3931	NA	0.2001	0.0773	0.0332	0.01988	+++-+
rs9288108	2	153386833	a	c	0.3262	0.4146	0.3949	0.7429	0.3880	0.2159	0.1054	0.0483	0.0207	0.01993	+++++
rs2678305	2	153403715	t	c	0.6132	0.7988	0.3758	0.9379	0.3335	0.0153	0.4129	0.0553	0.0239	0.0207	+++++
rs77136812	2	153398709	t	c	0.9583	0.9812	0.4332	0.6447	0.9671	0.9549	0.0153	0.1297	0.0563	0.02125	+++++
rs12693405	2	153430606	g	a	0.2191	0.3105	0.7613	0.0045	0.5225	NA	0.0781	0.0641	0.0279	0.02148	-++-+
rs113166685	2	153424318	a	t	0.9748	0.9864	0.0991	NA	0.8768	NA	0.0502	0.1811	0.079	0.02184	+?-?+
rs3811576	2	153282544	g	t	0.7732	0.8864	0.7105	0.0510	0.2616	0.0063	0.0365	-0.0618	0.0269	0.0219	+++--
rs113829840	2	153477160	c	g	0.9561	0.9884	0.4266	NA	0.3373	0.7510	0.0386	0.1375	0.06	0.02198	+?+++
rs76762063	2	153403933	t	c	0.9236	0.9671	0.7645	0.7972	0.5594	0.1599	0.0465	0.1219	0.0534	0.02235	+-+++
rs35776654	2	153468107	c	t	0.8586	0.9484	0.8451	NA	0.2707	0.2155	0.0680	0.0758	0.0334	0.02303	+-+++
rs4664112	2	153362927	c	t	0.3611	0.4561	0.1131	0.1983	0.1443	0.3253	0.0041	0.0474	0.0209	0.02374	-+-++
rs2304556	2	153473794	g	t	0.3078	0.4354	0.6299	0.5218	0.4041	0.2584	0.0434	0.0476	0.0212	0.02508	++-++
rs73968029	2	153331545	c	a	0.8602	0.9148	0.1530	0.7846	0.2306	0.1408	0.2012	0.0755	0.0338	0.0254	+-+++
rs12474927	2	153391582	a	g	0.7622	0.9348	0.0302	0.0481	0.3439	0.0260	0.0263	0.0577	0.0258	0.0254	-+-++
rs16831307	2	153390922	g	a	0.2862	0.3908	0.1255	0.9015	0.3272	0.2951	0.1594	0.0472	0.0211	0.02544	+++++
rs7592283	2	153344389	c	t	0.3638	0.5697	0.1345	0.7516	0.1568	0.1934	0.0042	0.0465	0.0208	0.02566	-+-++
rs55914172	2	153392034	a	g	0.7622	0.9348	0.0302	0.0481	0.3439	0.0278	0.0263	0.0574	0.0258	0.02615	-+-++
rs10180542	2	153327452	g	c	0.8427	0.9148	0.1530	0.7544	0.2306	0.1342	0.2023	0.0744	0.0335	0.02639	+-+++
rs66839581	2	153469039	t	c	0.0484	0.1599	0.3371	0.3277	0.5517	NA	0.0377	0.0778	0.0351	0.02644	++-?+
rs16831169	2	153331699	a	g	0.7902	0.9148	0.1530	0.9455	0.2306	0.1687	0.2012	0.0724	0.0327	0.02654	+-+++
rs11689419	2	153467816	a	g	0.1126	0.1623	0.3986	NA	0.9676	NA	0.0336	0.0796	0.0361	0.02735	+?-?+
rs6739525	2	153332898	a	t	0.0171	0.0261	0.8843	NA	0.4525	0.0551	0.0659	0.1578	0.0717	0.02782	-?-++
rs6751576	2	153251742	g	a	0.6402	0.7558	0.3184	0.2056	0.2383	0.2790	0.0010	-0.0501	0.0229	0.02872	+++--
rs10193625	2	153314204	c	t	0.2744	0.4325	0.0420	0.8180	0.1606	0.0108	0.0364	0.0483	0.0221	0.02886	-+-++
rs12622731	2	153383345	a	t	0.265	0.3918	0.3140	0.3804	0.4084	0.2582	0.1961	0.0463	0.0212	0.02886	+++++
rs62180830	2	153385021	c	t	0.7607	0.9335	0.0202	0.0480	0.3857	0.0259	0.0279	0.0564	0.0258	0.02906	-+-++
rs7595822	2	153381503	g	a	0.604	0.8998	0.0469	0.3225	0.1603	0.0150	0.6162	0.062	0.0284	0.02908	+-+++
rs11904088	2	153381728	a	g	0.3681	0.4218	0.5245	0.8389	0.4826	0.2413	0.1068	0.0449	0.0207	0.02997	+++++
rs12619679	2	153379006	t	c	0.3684	0.4218	0.5237	0.8351	0.4826	0.2375	0.1100	0.0448	0.0207	0.03035	+++++
rs7606592	2	153347813	t	a	0.3846	0.6353	0.1214	0.9664	0.1587	0.2115	0.0034	-0.0451	0.0208	0.03046	+++--

rs62180831	2	153386258	t	c	0.7607	0.9335	0.0202	0.0478	0.3353	0.0321	0.0264	0.0555	0.0258	0.03162	-+-++
rs6708667	2	153250565	g	a	0.641	0.7558	0.3184	0.2354	0.2367	0.3343	0.0010	-0.0493	0.0229	0.03168	+++--
rs2678301	2	153399594	a	g	0.7666	0.8605	0.0164	0.4733	0.3718	0.0192	0.0252	0.0542	0.0253	0.03193	-+-++
rs10209069	2	153384255	c	t	0.2786	0.3919	0.3284	0.4238	0.3834	0.2303	0.2276	0.0452	0.0211	0.03243	+++++
rs2459776	2	153404900	a	t	0.6151	0.7989	0.3858	0.9368	0.3546	0.0127	0.5444	0.0509	0.0239	0.03267	+-+++
rs62180832	2	153386761	t	c	0.7607	0.9331	0.0202	0.0474	0.3353	0.0321	0.0283	0.0549	0.0258	0.03336	-+-++
rs7421149	2	153380304	c	g	0.6041	0.8998	0.0469	0.3258	0.1603	0.0149	0.6863	0.0603	0.0284	0.03389	+-+++
rs6757782	2	153278499	a	c	0.7353	0.8335	0.6912	0.1463	0.4232	0.0494	0.0225	-0.0538	0.0254	0.03407	+++--
rs4664116	2	153430993	t	g	0.1371	0.3093	0.7613	0.0177	0.5302	NA	0.0781	0.0594	0.0281	0.03437	-++?+
rs11904617	2	153359258	t	c	0.7511	0.913	0.1103	0.0673	0.2974	0.8047	0.2502	0.0668	0.0316	0.03447	+++++
rs12478681	2	153482128	a	g	0.1171	0.1532	0.4611	0.4813	0.7909	0.9858	0.0210	0.063	0.0298	0.0345	+++-+
rs12623070	2	153318339	t	c	0.5687	0.7248	0.0419	0.9754	0.1626	0.0156	0.0308	-0.0466	0.0221	0.03488	+++--
rs16831174	2	153333513	g	a	0.8215	0.9148	0.1433	0.5396	0.2306	0.1491	0.2004	0.0698	0.0331	0.03497	+-+++
rs10931154	2	153388043	t	c	0.2693	0.3863	0.1424	0.7217	0.3447	0.3597	0.2007	0.0444	0.0211	0.03584	+++++
rs12467702	2	153398723	t	g	0.7657	0.9347	0.0142	0.0581	0.3675	0.0391	0.0248	0.0547	0.0261	0.03612	-+-++
rs12989943	2	153429985	c	g	0.1367	0.3093	0.7799	0.0195	0.5302	NA	0.0824	0.0588	0.0281	0.03625	-++?+
rs10168692	2	153371191	g	c	0.268	0.3901	0.3171	0.3876	0.4256	0.3016	0.2082	0.0446	0.0213	0.03626	+++++
rs1837105	2	153418270	c	t	0.2185	0.3106	0.7444	0.0716	0.4873	NA	0.0711	0.0582	0.0278	0.03627	-++-+
rs7586195	2	153367873	t	a	0.756	0.9148	0.0869	0.1156	0.2147	0.7643	0.2501	0.0662	0.0316	0.03634	+++++
rs62181862	2	153400547	a	g	0.7644	0.839	0.0148	0.4481	0.3738	0.0212	0.0307	0.0523	0.025	0.03669	-+-++
rs10194603	2	153339304	t	c	0.8383	0.9148	0.1471	0.5185	0.2306	0.1569	0.2077	0.0697	0.0334	0.03687	+-+++
rs727602	2	153502888	t	a	0.691	0.924	0.3369	0.0951	0.2657	NA	0.2572	0.0796	0.0384	0.03809	+++-+
rs11696004	2	153279726	a	g	0.772	0.877	0.7179	0.0260	0.2616	0.0148	0.0404	-0.0553	0.0267	0.03855	+++--
rs77908057	2	153409486	a	t	0.9676	0.9847	0.9801	NA	0.3850	0.4243	0.0668	0.1349	0.0655	0.03947	+?+++
rs2678303	2	153403533	t	c	0.6439	0.7997	0.4519	0.5146	0.3299	0.0190	0.3983	0.0495	0.0241	0.03962	+-+++
rs6434098	2	153415376	t	g	0.142	0.3092	0.7042	0.0390	0.5318	NA	0.0745	0.0575	0.028	0.04021	-++?+
rs10173899	2	153355512	a	g	0.8123	0.9148	0.1130	0.9723	0.2306	0.2417	0.2641	0.0675	0.0329	0.0404	+-+++
rs7582984	2	153367453	g	a	0.7559	0.9142	0.0905	0.1005	0.2147	0.8630	0.2510	0.0643	0.0316	0.04179	+++++
rs2678304	2	153403545	t	c	0.6444	0.7994	0.4212	0.5206	0.3342	0.0186	0.4306	0.0489	0.0241	0.04199	+-+++
rs7594969	2	153367447	t	g	0.7562	0.9145	0.0943	0.1017	0.2147	0.8630	0.2510	0.0641	0.0316	0.04233	+++++
rs60446845	2	153258618	g	a	0.0124	0.0204	0.0581	NA	0.5137	0.8256	0.0496	0.1615	0.0797	0.04271	+--++
rs934750	2	153404391	c	t	0.8317	0.8698	0.6712	0.8697	0.6219	0.2947	0.0789	0.0601	0.0297	0.04319	+-+++
rs2577176	2	153402873	g	a	0.6229	0.7999	0.4253	0.2769	0.3370	0.0109	0.4396	0.0483	0.0239	0.04354	+-+++
rs1545134	2	153403242	c	t	0.6499	0.7994	0.4212	0.5115	0.3342	0.0215	0.4220	0.0485	0.0241	0.04438	+-+++
rs10497102	2	153380844	a	g	0.9388	0.9596	0.2343	0.6696	0.5823	0.9552	0.0374	0.0904	0.045	0.0444	+-+-+
rs7587450	2	153276774	c	t	0.7368	0.8319	0.7169	0.1247	0.3837	0.0594	0.0262	-0.051	0.0254	0.04475	+++--
rs2577185	2	153389851	g	t	0.8907	0.9442	0.1666	0.2781	0.5531	0.7441	0.0208	0.0812	0.0405	0.04523	+-+++
rs74866949	2	153380071	c	t	0.9385	0.9596	0.2551	0.6669	0.5823	0.9552	0.0374	0.0897	0.0449	0.04601	+-+-+
rs35066174	2	153415244	t	c	0.7519	0.829	0.0960	NA	0.4025	NA	0.0050	0.0588	0.0296	0.04667	-?-?+
rs6434114	2	153458809	a	t	0.0425	0.0572	0.1167	NA	0.2797	NA	0.0682	-0.109	0.0548	0.04679	-?+?-
rs77936147	2	153422159	c	t	0.9682	0.9854	0.8067	NA	0.1847	0.5696	0.0918	0.1318	0.0665	0.0474	+-+++
rs17400191	2	153463568	t	c	0.7451	0.8813	0.0313	0.3277	0.3819	0.2385	0.0096	0.0511	0.0258	0.0475	-+-++
rs7607506	2	153446104	g	a	0.1837	0.3185	0.4423	0.0749	0.5756	NA	0.0570	0.0545	0.0275	0.0477	-++-+
rs6739300	2	153397674	t	c	0.8626	0.9583	0.1535	0.5138	0.2954	0.0008	0.2264	0.0814	0.0415	0.04959	++++-

Table S5. Association of *FMNL2* expression in individuals with both clinical and pathological AD compared to healthy individuals ante and post-mortem

Cohort	Brain region	n	b	SE	p
ROSMAP	Dorsolateral prefrontal cortex (DLPFC)	532	1.64	0.305	7.89E-08
	Posterior cingulate cortex (PCC)	318	2.178	0.494	1.05E-05
	Anterior caudate (AC)	333	1.491	0.519	0.004

Table S6. Individual model results for mediation analyses

Model					Mediated effect		Direct effect		Proportion mediated		Total effect	
**Predictor**	→	**Mediator**	→	**Outcome**	**Coef. [95% CI]**	**Sig.**	**Coef. [95% CI]**	**Sig.**	**Coef. [95% CI]**	**Sig.**	**Coef. [95% CI]**	**Sig.**
FMNL2	→	Amyloid	→	AD	0.010 [0.0002, 0.07]	<2e-16	0.004 [-0.024, 0.02]	0.322	0.740 [0.415, 1.48]	0.002	0.014 [0.0003, 0.05]	0.002
FMNL2	→	Tau	→	AD	0.012 [0.0005, 0.07]	<2e-16	0.006 [-0.025, 0.02]	0.22	0.660 [0.373, 1.41]	0.01	0.018 [0.001, 0.05]	0.01
Amyloid	→	*FMNL2*	→	Infarcts (gcc)	0.003 [0.001, 0.010]	0.002	0.008 [-0.010, 0.020]	0.404	0.290 [-2.568, 3.24]	0.206	0.011 [-0.007, 0.020]	0.204
Tau	→	*FMNL2*	→	Infarcts (gcc)	0.003 [0.001,0.01]	<2e-16	0.002 [-0.015,0.01]	0.8	0.562 [-3.887,5.18]	0.51	0.005 [-0.012,0.02]	0.51

Note: Effects are reported from causal mediation analyses using nonparametric bootstrapped confidence intervals with 1000 resamples. gcc: Gross chronic infarctions in cortex;

Table S7. Top SNP ($p<0.05$) in FMNL2 associated with *FMNL2* expression

snp	chr	bp	A1	A2	MinFreq	MaxFreq	b	SE	p
rs1023754	2	153460454	t	c	0.5038	0.6421	0.071	0.025	0.004
rs71417234	2	153500983	a	g	0.9676	0.9831	0.174	0.073	0.017
rs111959109	2	153445907	a	g	0.0294	0.0564	-0.117	0.049	0.018
rs6744391	2	153334614	c	t	0.4066	0.8131	-0.046	0.021	0.025
rs77136812	2	153398709	t	c	0.9583	0.9812	0.118	0.053	0.027
rs34069841	2	153488297	a	g	0.943	0.9751	0.142	0.065	0.029
rs17328224	2	153456903	c	g	0.145	0.2485	0.053	0.024	0.03
rs34536210	2	153481832	t	c	0.0243	0.0564	-0.141	0.065	0.03
rs34635042	2	153484075	t	c	0.9441	0.9757	0.141	0.065	0.03
rs78926321	2	153347966	a	g	0.9654	0.9829	-0.122	0.058	0.035
rs140710129	2	153467818	a	c	0.9084	0.9299	0.082	0.039	0.036
rs116727388	2	153406206	a	t	0.9805	0.9922	-0.242	0.118	0.041
rs893330	2	153460315	t	c	0.0545	0.1089	0.074	0.037	0.043
rs10931150	2	153369892	a	g	0.3543	0.5172	-0.045	0.023	0.047

Table S8. SNPs associated with hypomethylation of several CPG sites in and around the *FMNL2* gene

snp	chr	bp	A1	A2	featureName	featurePositionStart	b	se	t	p
rs4664586	2	153324071	T	C	cg04308657	153362242	-0.19624	0.042157	-4.65504	4.08E-06
rs4664586	2	153324071	T	C	cg04681845	153283485	-0.20722	0.04206	-4.92672	1.11E-06
rs4664586	2	153324071	T	C	cg05027533	153290479	-0.18076	0.042285	-4.27474	2.26E-05
rs4664586	2	153324071	T	C	cg05696136	153318682	-0.21129	0.042023	-5.0281	6.75E-07
rs4664586	2	153324071	T	C	cg05868491	153339703	-0.03895	0.042961	-0.90655	0.36505
rs4664586	2	153324071	T	C	cg05935184	153350189	0.017474	0.042987	0.406493	0.684541
rs4664586	2	153324071	T	C	cg09169117	153302353	0.015666	0.042988	0.364416	0.71569
rs4664586	2	153324071	T	C	cg11172396	153295007	0.112277	0.042722	2.628109	0.00883
rs4664586	2	153324071	T	C	cg11786365	153305731	-0.04598	0.042948	-1.0705	0.284872
rs4664586	2	153324071	T	C	cg11994229	153310258	-0.15303	0.042487	-3.60183	0.000345
rs4664586	2	153324071	T	C	cg15571231	153277513	-0.05914	0.042918	-1.37799	0.168776
rs4664586	2	153324071	T	C	cg15705810	153357492	-0.08393	0.042842	-1.95915	0.050608
rs4664586	2	153324071	T	C	cg18686547	153326947	0.043618	0.042952	1.015505	0.310319
rs4664586	2	153324071	T	C	cg19402624	153278845	0.078163	0.042862	1.823595	0.068765
rs4664586	2	153324071	T	C	cg20171711	153307345	-0.03339	0.042969	-0.77702	0.437487
rs4664586	2	153324071	T	C	cg24749089	153322823	0.121807	0.042673	2.854416	0.004477
rs4664586	2	153324071	T	C	cg26204803	153364045	0.005862	0.042993	0.136341	0.891602
rs4664586	2	153324071	T	C	cg26207245	153347526	-0.04365	0.042952	-1.01625	0.309965
rs4664586	2	153324071	T	C	cg26676845	153280733	0.031211	0.042972	0.7263	0.467969

Table S9. Gliovascular interactions in dIHCs for kdrl:GFP and S100b in control and amyloid-injected brains with DAPI counterstain, related to Figure 3F.

Blood vessel - glia interaction surface area normalized to vasculature surface	Mean,.Intensities.#1	Sum,.Intensities.#1	Mean,.Intensities.#2	Sum,.Intensities.#2	Mean,.Intensities.#3	Sum,.Intensities.#3	Mean,.Intensities.#4	Sum,.Intensities.#4	Volume,.Volume.(µm³)	VoxelCount,.Volume	Surface.Area.(µm²)	
Ab42 3dpi_1.1_i_Apo_Ab42 3dpi_1.1_i_Apo.czi (default)__GFP+S100.xlsx	0.086834102	0.004434154	0.443061858	0.009159616	0.07380749	0.005648962	0.271461638	0.042348069	0.009332778	0.009332778	0.026051503	Amyloid 3 dpi
Ab42 3dpi_1.1_ii_Apo_Ab42 3dpi_1.1_ii_Apo.czi (default)__GFP+S100.xlsx	0.164033028	0.020628289	0.558144691	0.023006644	0.147329339	0.03228766	0.18070114	0.068944411	0.031726534	0.031726534	0.064893658	
Ab42 3dpi_1.1_iii_Apo_Ab42 3dpi_1.1_iii_Apo.czi (default)__GFP+S100.xlsx	0.202211205	0.027779933	0.603450425	0.033058445	0.169379253	0.043295744	0.083960151	0.04866598	0.036852025	0.036852025	0.073589008	
Ab42 3dpi_1.1_iv_Apo_Ab42 3dpi_1.1_iv_Apo.czi (default)__GFP+S100.xlsx	0.109740613	0.020986582	0.356431798	0.027824661	0.159042474	0.057778343	0.106200047	0.074066863	0.033451094	0.033451094	0.052037138	
A3_1.5_13_Apo_13_Apo.czi (default)__GFP+S100.xlsx	0.156637342	0.004876942	0.481126017	0.011115178	0.310434218	0.054419461			0.014685406	0.014685406	0.030320765	
A3_1.6_14_Apo_14_Apo.czi (default)__GFP+S100.xlsx	0.309583078	0.009089825	1.236549992	0.013936778	0.48568379	0.066311683			0.018837889	0.018837889	0.044618852	
A3_1.7_16_Apo_16_Apo.czi (default)__GFP+S100.xlsx	0.107071069	0.002946024	0.34220295	0.004393987	0.201037353	0.019585909			0.004339521	0.004339521	0.011564707	
Ab42 3dpi_2.1_i_Apo_Ab42 3dpi_2.1_i_Apo.czi (default)__GFP+S100.xlsx	0.193338443	0.011314585	0.710238571	0.012247588	0.308915804	0.019176116	0.462894081	0.071206485	0.01905886	0.01905886	0.040969146	
Ab42 3dpi_2.1_ii_Apo_Ab42 3dpi_2.1_ii_Apo.czi (default)__GFP+S100.xlsx	0.3424694	0.024281364	0.803663931	0.014201208	0.363110871	0.03953662	0.459465844	0.086594267	0.033304241	0.033304241	0.06891165	
Ab42 3dpi_2.1_iii_Apo_Ab42 3dpi_2.1_iii_Apo.czi (default)__GFP+S100.xlsx	0.122392263	0.014421966	0.507862378	0.01614048	0.108060956	0.022878483	0.078052725	0.035123387	0.018386213	0.018386213	0.031992533	
Ab42 3dpi_2.1_iv_Apo_Ab42 3dpi_2.1_iv_Apo.czi (default)__GFP+S100.xlsx	0.118638346	0.004834457	0.394866228	0.005337859	0.170286999	0.02109528	0.275209173	0.037363432	0.011511256	0.011511256	0.027536291	
A3_2.5_17_Apo_17_Apo.czi (default)__GFP+S100.xlsx	0.111132839	0.020220381	0.435242807	0.015406157	0.348259691	0.071474469			0.017219381	0.017219381	0.0389339	
A3_2.6_18_Apo_18_Apo.czi (default)__GFP+S100.xlsx	0.066617672	0.019692476	0.253568846	0.01870734	0.121604277	0.077452154			0.025099781	0.025099781	0.042099079	
A3_2.7_19_Apo_19_Apo.czi (default)__GFP+S100.xlsx	0.047190284	0.00146335	0.167916754	0.001295826	0.289509028	0.015982272			0.002924605	0.002924605	0.008667884	
Ab42 3dpi_3.1_i_Apo_Ab42 3dpi_3.1_i_Apo.czi (default)__GFP+S100.xlsx	0.084837213	0.011958093	0.357562079	0.014445119	0.115683058	0.022633324	0.211549412	0.06328144	0.015557071	0.015557071	0.046839463	
Ab42 3dpi_3.1_ii_Apo_Ab42 3dpi_3.1_ii_Apo.czi (default)__GFP+S100.xlsx	0.018305507	0.001823089	0.120506636	0.005727352	0.022616643	0.003819515	0.04625544	0.019308642	0.003965561	0.003965561	0.010055108	
Ab42 3dpi_3.1_iii_Apo_Ab42 3dpi_3.1_iii_Apo.czi (default)__GFP+S100.xlsx	0.02973586	0.024540858	0.094423787	0.036272368	0.027862077	0.042071249	0.025509823	0.097240569	0.034798143	0.034798143	0.043068297	
Ab42 3dpi_3.1_iv_Apo_Ab42 3dpi_3.1_iv_Apo.czi (default)__GFP+S100.xlsx	0.034813419	0.019391121	0.15047466	0.023350663	0.039704817	0.028182556	0.054424907	0.066315847	0.028366769	0.028366769	0.045787855	
A3_3.5_21_Apo_21_Apo.czi (default)__GFP+S100.xlsx	0.077758743	0.005277493	0.248744606	0.006406988	0.355366135	0.042102247			0.011616085	0.011616085	0.025366492	
A3_3.6_23_Apo_23_Apo.czi (default)__GFP+S100.xlsx	0.098114204	0.013366272	0.206498958	0.012140552	0.120759957	0.063160885			0.019799095	0.019799095	0.04326603	
A3_3.7_24_Apo_24_Apo.czi (default)__GFP+S100.xlsx	0.225871513	0.015966145	0.780040368	0.016398827	0.473558959	0.07084576			0.023331429	0.023331429	0.052405065	
Ab42 7dpi_1.1_i_Apo_Ab42 7dpi_1.1_i_Apo.czi (default)__GFP+S100.xlsx	0.040237735	0.030043306	0.203591192	0.045468152	0.038720697	0.034412829	0.043014845	0.094288118	0.040871068	0.040871068	0.056322778	Amyloid 7 dpi
Ab42 7dpi_1.1_ii_Apo_Ab42 7dpi_1.1_ii_Apo.czi (default)__GFP+S100.xlsx	0.042651266	0.027905355	0.213339511	0.044301946	0.052842863	0.038477735	0.056230862	0.094960988	0.039123039	0.039123039	0.051992428	
Ab42 7dpi_1.1_iii_Apo_Ab42 7dpi_1.1_iii_Apo.czi (default)__GFP+S100.xlsx	0.019759174	0.014822605	0.085629167	0.026524941	0.027497935	0.026291205	0.023335776	0.057471007	0.021070743	0.021070743	0.026153051	
Ab42 7dpi_1.1_iv_Apo_Ab42 7dpi_1.1_iv_Apo.czi (default)__GFP+S100.xlsx	0.119682126	0.022334669	0.45509649	0.027237403	0.121270166	0.028945028	0.163590819	0.082966841	0.029227557	0.029227557	0.059049263	
A7_1.5_26_Apo_26_Apo.czi (default)__GFP+S100.xlsx	0.049348935	0.015483728	0.284755802	0.023896771	0.086147653	0.065989426			0.02198594	0.02198594	0.036571435	
A7_1.6_27_Apo_27_Apo.czi (default)__GFP+S100.xlsx	0.107292356	0.015658081	0.363707452	0.017560331	0.176252257	0.057173403			0.021325534	0.021325534	0.037765476	
A7_1.7_28_Apo_28_Apo.czi (default)__GFP+S100.xlsx	0.046726675	0.00454719	0.226041976	0.006663666	0.239474094	0.029108302			0.007868866	0.007868866	0.018404633	
Ab42 7dpi_2.1_i_Apo_Ab42 7dpi_2.1_i_Apo.czi (default)__GFP+S100.xlsx	0.027174973	0.022254814	0.154322338	0.0298689	0.026527897	0.016182564	0.029946073	0.07329576	0.030307224	0.030307224	0.058009463	
Ab42 7dpi_2.1_ii_Apo_Ab42 7dpi_2.1_ii_Apo.czi (default)__GFP+S100.xlsx	0.069030914	0.021846249	0.248899183	0.021783315	0.049285775	0.019271974	0.060730641	0.03424813	0.018526076	0.018526076	0.028308861	
Ab42 7dpi_2.1_iii_Apo_Ab42 7dpi_2.1_iii_Apo.czi (default)__GFP+S100.xlsx	0.136655302	0.011004912	0.307210124	0.010751763	0.090226087	0.014357105	0.267157651	0.0407759	0.015334176	0.015334176	0.031219838	
Ab42 7dpi_2.1_iv_Apo_Ab42 7dpi_2.1_iv_Apo.czi (default)__GFP+S100.xlsx	0.086465734	0.005657301	0.305690859	0.011322613	0.107882088	0.012967496	0.319959405	0.042454419	0.013986114	0.013986114	0.027981316	
A7_2.5_29_Apo_29_Apo.czi (default)__GFP+S100.xlsx	0.124507183	0.020599463	0.303428555	0.021462869	0.163543495	0.090601496			0.023417997	0.023417997	0.056428082	
A7_2.6_31_Apo_31_Apo.czi (default)__GFP+S100.xlsx	0.086042777	0.026088499	0.334222203	0.048196978	0.139926742	0.132050013			0.044822932	0.044822932	0.06867077	
A7_2.7_32_Apo_32_Apo.czi (default)__GFP+S100.xlsx	0.157045435	0.016275857	0.306059966	0.011985028	0.128017226	0.0562857			0.020517405	0.020517405	0.046487289	
Ab42 7dpi_3.1_i_Apo_Ab42 7dpi_3.1_i_Apo.czi (default)__GFP+S100.xlsx	0.103558193	0.012056046	0.361886416	0.014066079	0.083659449	0.023169494	0.202576928	0.089841777	0.023804329	0.023804329	0.050777294	
Ab42 7dpi_3.1_ii_Apo_Ab42 7dpi_3.1_ii_Apo.czi (default)__GFP+S100.xlsx	0.030927304	0.01094063	0.240354182	0.018478478	0.052211956	0.021918773	0.096570889	0.063880868	0.021686007	0.021686007	0.038499968	
Ab42 7dpi_3.1_iii_Apo_Ab42 7dpi_3.1_iii_Apo.czi (default)__GFP+S100.xlsx	0.12801105	0.014758767	0.60207403	0.017862303	0.060425576	0.022593355	0.178952496	0.076962658	0.020922319	0.020922319	0.041102161	
Ab42 7dpi_3.1_iv_Apo_Ab42 7dpi_3.1_iv_Apo.czi (default)__GFP+S100.xlsx	0.014369124	0.019033093	0.108039737	0.030744919	0.021712199	0.023592844	0.028703409	0.056275766	0.024196782	0.024196782	0.027945662	
A7_3.5_33_Apo_33_Apo.czi (default)__GFP+S100.xlsx	0.059017219	0.008555898	0.192436377	0.020225462	0.060273141	0.055944418			0.021372152	0.021372152	0.036404427	
A7_3.6_34_Apo_34_Apo.czi (default)__GFP+S100.xlsx	0.107394038	0.009316136	0.313552758	0.011460134	0.211486154	0.04207614			0.016276939	0.016276939	0.036971424	
A7_3.7_35_Apo_35_Apo.czi (default)__GFP+S100.xlsx	0.18834814	0.017409071	0.768612094	0.024721458	0.452996884	0.085254562			0.03596823	0.03596823	0.065272302	
Ctrl PBS_1.1_i_Apo_Ctrl PBS_1.1_i_Apo.czi (default)__GFP+S100.xlsx	0.105738803	0.019597487	0.256883976	0.026412035	0.094115798	0.027769813	0.222664749	0.105819679	0.024425229	0.024425229	0.043320134	Control
Ctrl PBS_1.1_ii_Apo_Ctrl PBS_1.1_ii_Apo.czi (default)__GFP+S100.xlsx	0.04107815	0.020346601	0.157137324	0.02974632	0.068479528	0.072872927	0.056184776	0.126629008	0.040835558	0.040835558	0.068064426	
Ctrl PBS_1.1_iii_Apo_Ctrl PBS_1.1_iii_Apo.czi (default)__GFP+S100.xlsx	0.149361065	0.020917865	0.739262953	0.024878623	0.158253715	0.029922747	0.236643926	0.0821485	0.031527028	0.031527028	0.073747569	
Ctrl PBS_1.1_iv_Apo_Ctrl PBS_1.1_iv_Apo.czi (default)__GFP+S100.xlsx	0.224214422	0.018679444	0.781744081	0.025444899	0.230067624	0.028604603	0.359156841	0.067119621	0.030416569	0.030416569	0.064390061	
C_1.5_Apo_1_Apo.czi (default)__GFP+S100.xlsx	0.333616515	0.015150754	0.961618215	0.017316195	0.542016662	0.08115015			0.033260292	0.033260292	0.07472614	
C_1.6_Apo_2_Apo.czi (default)__GFP+S100.xlsx	0.104408132	0.034208828	0.437673474	0.038989565	0.152684182	0.11394659			0.048057893	0.048057893	0.088281994	
C_1.7_Apo_4_Apo.czi (default)__GFP+S100.xlsx	0.103538105	0.030259128	0.428358156	0.040882252	0.143916363	0.094070121			0.041931696	0.041931696	0.077861248	
Ctrl PBS_2.1_i_Apo_Ctrl PBS_2.1_i_Apo.czi (default)__GFP+S100.xlsx	0.012285275	0.017975131	0.091948409	0.035205934	0.018359423	0.023099655	0.020174955	0.054943698	0.028587071	0.028587071	0.033672093	
Ctrl PBS_2.1_ii_Apo_Ctrl PBS_2.1_ii_Apo.czi (default)__GFP+S100.xlsx	0.029065598	0.014013191	0.190294382	0.022784949	0.05124623	0.017295346	0.074738341	0.060146968	0.023089906	0.023089906	0.033405822	
Ctrl PBS_2.1_iii_Apo_Ctrl PBS_2.1_iii_Apo.czi (default)__GFP+S100.xlsx	0.080273775	0.021652259	0.376888	0.022695261	0.0571221	0.018188928	0.23909529	0.056577765	0.028566391	0.028566391	0.059813107	
Ctrl PBS_2.1_iv_Apo_Ctrl PBS_2.1_iv_Apo.czi (default)__GFP+S100.xlsx	0.061773194	0.013041362	0.219278203	0.01743097	0.045834865	0.015322658	0.180265086	0.065129218	0.021546226	0.021546226	0.040395873	
C_2.5_Apo_5_Apo.czi (default)__GFP+S100.xlsx	0.050362576	0.015643776	0.205418451	0.020549888	0.058357266	0.051305358			0.021259006	0.021259006	0.032104223	
C_2.6_Apo_7_Apo.czi (default)__GFP+S100.xlsx	0.061720845	0.02096708	0.306023804	0.0332066	0.103327275	0.083896528			0.040789164	0.040789164	0.070914093	
C_2.7_Apo_8_Apo.czi (default)__GFP+S100.xlsx	0.102005793	0.047899077	0.518008902	0.053735608	0.129654174	0.144145414			0.058010577	0.058010577	0.09044062	
Ctrl PBS_3.1_i_Apo_Ctrl PBS_3.1_i_Apo.czi (default)__GFP+S100.xlsx	0.132589181	0.035931174	0.307243352	0.015186091	0.102874008	0.026435213	0.155530123	0.065369834	0.02960862	0.02960862	0.056639284	
Ctrl PBS_3.1_ii_Apo_Ctrl PBS_3.1_ii_Apo.czi (default)__GFP+S100.xlsx	0.077731763	0.008077849	0.381633978	0.013572895	0.074063287	0.022395798	0.120021216	0.04571787	0.017491622	0.017491622	0.035810411	
Ctrl PBS_3.1_iii_Apo_Ctrl PBS_3.1_iii_Apo.czi (default)__GFP+S100.xlsx	0.211541949	0.017689858	0.606372386	0.017895386	0.178352891	0.026319205	0.342519337	0.074402011	0.02956156	0.02956156	0.055361256	
Ctrl PBS_3.1_iv_Apo_Ctrl PBS_3.1_iv_Apo.czi (default)__GFP+S100.xlsx	0.116494259	0.03087046	0.243264632	0.043368068	0.147948242	0.038947347	0.085538732	0.061964936	0.03441121	0.03441121	0.033696991	
C_3.5_Apo_10_Apo.czi (default)__GFP+S100.xlsx	0.112707916	0.011857699	0.378074863	0.014424684	0.27298818	0.071494973			0.021871419	0.021871419	0.04496173	
C_3.6_Apo_11_Apo.czi (default)__GFP+S100.xlsx	0.036900117	0.023343023	0.121493041	0.026367573	0.030545173	0.059276417			0.029777941	0.029777941	0.040456181	
C_3.7_Apo_12_Apo.czi (default)__GFP+S100.xlsx	0.071418247	0.023632543	0.274950645	0.034646444	0.095455275	0.103450357			0.045852571	0.045852571	0.085743538	

Table S10. Gliovascular interactions in dIHCs for kdrl:GFP and S100b in amyloid injected brains that were co-injected with control morpholino and fmnl2 morpholino with DAPI counterstain, related to Figure 3J.

**Blood vessel - glia interaction surface area normalized to vasculature surface**	Mean,.Intensities.#1	Sum,.Intensities.#1	Mean,.Intensities.#2	Sum,.Intensities.#2	Mean,.Intensities.#3	Sum,.Intensities.#3	Mean,.Intensities.#4	Sum,.Intensities.#4	Volume,.Volume.(µm³)	VoxelCount,.Volume	Surface.Area.(µm²)
A11_Apo_A11_Apo.czi (default)__GFP+S100.xlsx	0.514749858	0.009997571	1.064577801	0.020454632	0.508450278	0.067086628			0.029943592	0.029943592	0.092717931
A12_Apo_A12_Apo.czi (default)__GFP+S100.xlsx	0.793270153	0.049598467	1.362972121	0.039891773	0.604248557	0.165577784			0.071837668	0.071837668	0.169062685
A13_Apo_A13_Apo.czi (default)__GFP+S100.xlsx	0.488578154	0.023748799	0.651589934	0.030674198	0.715366975	0.163678659			0.06922367	0.06922367	0.181120982
A14_Apo_A14_Apo.czi (default)__GFP+S100.xlsx	0.81660795	0.024548251	0.945002973	0.047498176	0.303118096	0.067644873			0.049911129	0.049911129	0.126828704
A15_Apo_A15_Apo.czi (default)__GFP+S100.xlsx	0.372756677	0.053780296	0.483815506	0.080781948	0.231013292	0.177892502			0.119302984	0.119302984	0.233629142
A16_Apo_A16_Apo.czi (default)__GFP+S100.xlsx	0.301083938	0.027615878	0.457496823	0.041823854	0.142706207	0.077357521			0.051512625	0.051512625	0.104625128
A21_Apo_A21_Apo.czi (default)__GFP+S100.xlsx	1.051295275	0.035901631	1.635338204	0.044579381	0.352545919	0.067474729			0.054241873	0.054241873	0.147605714
A22_Apo_A22_Apo.czi (default)__GFP+S100.xlsx	1.132211596	0.046294096	1.843910178	0.041561645	0.769527847	0.074996785			0.055966967	0.055966967	0.174000669
A23_Apo_A23_Apo.czi (default)__GFP+S100.xlsx	1.176247719	0.064535584	1.327857822	0.064029631	0.390536405	0.096982645			0.073762737	0.073762737	0.201250816
A24_Apo_A24_Apo.czi (default)__GFP+S100.xlsx	0.37657592	0.078453097	0.57819422	0.090300118	0.310222926	0.172667087			0.127725293	0.127725293	0.246204924
A25_Apo_A25_Apo.czi (default)__GFP+S100.xlsx	0.981213266	0.053499689	1.267484137	0.036501644	0.523816982	0.122503807			0.059847907	0.059847907	0.157333869
A26_Apo_A26_Apo.czi (default)__GFP+S100.xlsx	0.302655741	0.070411115	0.511263189	0.079445318	0.2162141	0.1478484			0.108579422	0.108579422	0.184832578
A31_Apo_A31_Apo.czi (default)__GFP+S100.xlsx	1.581284977	0.031667524	2.357334368	0.042862504	0.827177577	0.11989141			0.060758777	0.060758777	0.214855348
A32_Apo_A32_Apo.czi (default)__GFP+S100.xlsx	1.236553262	0.019988093	1.603940951	0.026426247	1.122969144	0.106929263			0.03964608	0.03964608	0.161410822
A33_Apo_A33_Apo.czi (default)__GFP+S100.xlsx	1.043401274	0.047547344	3.634985171	0.058369398	2.121989074	0.181216078			0.073930431	0.073930431	0.247812784
A34_Apo_A34_Apo.czi (default)__GFP+S100.xlsx	0.990656805	0.05467791	2.216059492	0.086358341	1.194608342	0.208122334			0.090570173	0.090570173	0.234151484
A35_Apo_A35_Apo.czi (default)__GFP+S100.xlsx	2.640578506	0.056301511	6.753737079	0.078202239	4.214897314	0.16598039			0.085122559	0.085122559	0.267181939
A36_Apo_A36_Apo.czi (default)__GFP+S100.xlsx	1.226633962	0.037198376	3.34040767	0.038788864	1.073840396	0.06503135			0.049395444	0.049395444	0.143433774
A41_Apo_A41_Apo.czi (default)__GFP+S100.xlsx	0.36257042	0.040452608	0.751242805	0.059252234	0.512437002	0.151830136			0.064517058	0.064517058	0.156797329
A42_Apo_A42_Apo.czi (default)__GFP+S100.xlsx	0.454704487	0.050416107	0.518903148	0.065129832	0.277186106	0.149471087			0.079415278	0.079415278	0.168774203
A43_Apo_A43_Apo.czi (default)__GFP+S100.xlsx	0.833940372	0.056609874	1.305556444	0.062854723	0.557487407	0.155100982			0.084439483	0.084439483	0.212323319
A44_Apo_A44_Apo.czi (default)__GFP+S100.xlsx	0.832004106	0.035306432	1.1228371	0.039332969	0.545238132	0.108021841			0.051058214	0.051058214	0.145356501
A45_Apo_A45_Apo.czi (default)__GFP+S100.xlsx	0.7093148	0.055410418	1.087349906	0.046796694	0.686559048	0.133535931			0.063855612	0.063855612	0.145848414
A46_Apo_A46_Apo.czi (default)__GFP+S100.xlsx	0.297774171	0.026407599	0.721359817	0.033057657	0.562715101	0.099650011			0.035876348	0.035876348	0.10005313
M11_Apo_M11_Apo.czi (default)__GFP+S100.xlsx	1.218664732	0.068664335	2.703787221	0.06644406	1.457112517	0.148635279			0.070692686	0.070692686	0.208177861
M12_Apo_M12_Apo.czi (default)__GFP+S100.xlsx	1.286021583	0.057679037	2.58772611	0.050364719	1.044300858	0.160477376			0.074353348	0.074353348	0.201027938
M13_Apo_M13_Apo.czi (default)__GFP+S100.xlsx	1.019089157	0.055439214	2.214060015	0.062196779	0.675746104	0.12204773			0.081463145	0.081463145	0.220897361
M14_Apo_M14_Apo.czi (default)__GFP+S100.xlsx	0.773055115	0.064077536	1.555107797	0.052562511	0.622158707	0.183639512			0.095496397	0.095496397	0.280632494
M15_Apo_M15_Apo.czi (default)__GFP+S100.xlsx	1.877632529	0.11691662	2.334377866	0.096506104	1.03549668	0.243197315			0.116369768	0.116369768	0.303020055
M16_Apo_M16_Apo.czi (default)__GFP+S100.xlsx	0.796820995	0.045925905	1.312668387	0.058641516	0.591201063	0.152882747			0.07279861	0.07279861	0.206922468
M21_Apo_M21_Apo.czi (default)__GFP+S100.xlsx	0.766192536	0.120736433	1.359536189	0.102105496	0.463712231	0.19279878			0.13380675	0.13380675	0.313377994
M22_Apo_M22_Apo.czi (default)__GFP+S100.xlsx	0.774502101	0.061284613	1.348501694	0.059647145	0.635548452	0.185105751			0.08422512	0.08422512	0.230282077
M23_Apo_M23_Apo.czi (default)__GFP+S100.xlsx	0.538037132	0.041150457	0.839176257	0.040872802	0.441027278	0.078966364			0.05814818	0.05814818	0.152981429
M24_Apo_M24_Apo.czi (default)__GFP+S100.xlsx	0.521250282	0.116656824	0.600777204	0.124410589	0.399650385	0.223011319			0.160874679	0.160874679	0.316568774
M25_Apo_M25_Apo.czi (default)__GFP+S100.xlsx	0.464739388	0.065540288	0.754412577	0.045480545	0.187523747	0.133835241			0.090064999	0.090064999	0.169180415
M26_Apo_M26_Apo.czi (default)__GFP+S100.xlsx	2.841215157	0.075258194	4.435722606	0.056346607	1.831220926	0.119006195			0.068924088	0.068924088	0.189295932
M31_Apo_M31_Apo.czi (default)__GFP+S100.xlsx	0.368425516	0.094227666	0.639541896	0.054640705	0.203659483	0.156588741			0.104660669	0.104660669	0.193935183
M32_Apo_M32_Apo.czi (default)__GFP+S100.xlsx	0.479770871	0.092687885	0.666000046	0.079487155	0.220397039	0.190786913			0.10491495	0.10491495	0.216183632
M33_Apo_M33_Apo.czi (default)__GFP+S100.xlsx	0.527836395	0.065203976	0.820896005	0.061188764	0.560415427	0.220146588			0.126354353	0.126354353	0.271078471
M34_Apo_M34_Apo.czi (default)__GFP+S100.xlsx	0.54035	0.084474441	0.664207625	0.102345641	0.498246023	0.293283506			0.177584976	0.177584976	0.356822219
M35_Apo_M35_Apo.czi (default)__GFP+S100.xlsx	0.606040854	0.133447354	0.574609273	0.096317495	0.20233987	0.190830595			0.127199704	0.127199704	0.25089673
M36_Apo_M36_Apo.czi (default)__GFP+S100.xlsx	0.431334506	0.030683581	0.626107449	0.041183695	0.271840047	0.123185534			0.067368283	0.067368283	0.155426144
M41_Apo_M41_Apo.czi (default)__GFP+S100.xlsx	0.296500361	0.046960406	0.304227258	0.031628584	0.102846376	0.059614763			0.046547209	0.046547209	0.088560079
M42_Apo_M42_Apo.czi (default)__GFP+S100.xlsx	0.245927626	0.060334202	0.324303466	0.065302855	0.138655486	0.108975333			0.087176564	0.087176564	0.159325928
M43_Apo_M43_Apo.czi (default)__GFP+S100.xlsx	0.246753774	0.104596328	0.337605308	0.129850179	0.251879758	0.305758747			0.197167017	0.197167017	0.312545335
M44_Apo_M44_Apo.czi (default)__GFP+S100.xlsx	0.80363517	0.188673678	0.984008743	0.210358803	1.027193946	0.428018729			0.267580607	0.267580607	0.496624602
M45_Apo_M45_Apo.czi (default)__GFP+S100.xlsx	0.874115781	0.029743285	1.386711132	0.030283254	0.780537276	0.108862617			0.045459401	0.045459401	0.136933191
M46_Apo_M46_Apo.czi (default)__GFP+S100.xlsx	0.969653239	0.089219779	1.157608646	0.081788867	0.766418108	0.192061139			0.124470994	0.124470994	0.291405152

Table S11. Quantification of activated microglia, related to Figure 4G.

Activated microglia: l-plastin staining

	**CoMO**	**CoMO+Ab42**	**MO+Ab42**
	48	62	38
	32	54	55
	42	69	49
	38	66	50
	35	71	51
fish 1	32	75	45
	39	48	32
	42	59	37
	38	56	43
	44	67	52
	36	69	39
fish 2	31	69	43
	55	70	52
	53	78	59
	48	63	46
	45	76	62
	51	77	44
fish 3	46	72	51
	58	64	46
	52	82	54
	56	79	39
	49	70	44
	43	85	53
fish 4	49	81	46

Table S12. Measurements of the blood vessel length in larval zebrafish after fmnl2 knockdown, related to Supplementary Figure 6F

Length of intersegmental blood vessels		
**uninjected**	**control mo**	**fmnl2a/b mo**
149.215	97.862	120.341
148.449	96.509	100.449
139.463	93.648	111.521
163.487	173.254	143.031
154.019	145.086	140.057
157.003	180.361	118.038
130.115	97.082	104.89
136.132	134	95.776
135.945	139.23	105.119
88.6	139.291	80.623
97.719	149.566	86.006
94.868	150.479	108.853
152.013	133.825	125.16
134.134	126.178	143.6
142.172	139.058	142.948
140.228	124.193	114.057
146.168	111.83	126.4
123.794	126.649	128.222
	105.891	107.787
	86.371	92.661
	100.21	96.385
	146.373	126.696
	117.273	132.774
	132.136	120.204
	122.25	156.978
	123.762	151.42
	121.433	158.382
	107.49	141.598
	98.595	123.102
	106.963	112.018

Table S13. Automated image analyses results for FMNL2 expression levels normalized to astroglial surface, related to Figure 6G

Group	Fmnl2 total intensity (arbitrary unit)	Total area of GFAP (um2)	Fmnl2 GFAP overlap (um2)	Ratio	Intensity normalized
Control	1753675	60125.17797	9178.494609	0.152656423	2.677097528
	1182617	54640.37798	8546.553246	0.156414607	1.849785732
	1178666	52175.22828	8947.920183	0.17149748	2.021382491
	1001222	32272.12903	5538.854392	0.171629656	1.718393871
	1195875	63643.27094	14871.06431	0.233662791	2.7943149
	655478	53701.69958	10627.90523	0.19790631	1.297232326
Pooled AD patients	1346500	49133.30518	11380.62011	0.231627408	3.118863045
	1559875	58475.18731	11125.10203	0.19025338	2.967714911
	1188491	27939.57689	7906.488104	0.282985248	3.363254207
	1810502	34986.73557	11356.6008	0.324597326	5.876841074
	1551495	43848.11779	11660.55565	0.265930586	4.125899744
	1173543	50679.56211	13349.62043	0.263412308	3.091256704
	1759011	64903.077	14538.52234	0.224003591	3.940247812
	1296815	80531.3483	19045.02259	0.236492037	3.066864208
	1210092	63577.78504	14929.51699	0.234822855	2.841572582
	1007282	18230.69413	7993.606048	0.438469649	4.416625845
	1206527	19047.60758	7661.852686	0.402247508	4.853224794
	588158	9681.486558	4203.379362	0.43416673	2.553586357
	1174261	22507.64252	7680.823175	0.341254006	4.007212703
	1583227	33282.66078	10147.51583	0.30488896	4.827084333
	1565249	49994.21054	10392.89476	0.207881966	3.253870389
	1429877	37548.462	10048.44609	0.267612721	3.826532751
	1916020	47173.12457	12468.04119	0.264303908	5.064115745
	831081	55115.99021	12209.21885	0.221518634	1.840999277
	1520989	73778.87686	19127.11664	0.259249225	3.943152193
	1417098	54534.83443	15638.69748	0.286765288	4.063745156
	1158493	76927.94397	15548.74841	0.202120941	2.341556951
	1311610	84741.33595	20593.03019	0.24301045	3.187349364
	1077858	67271.68051	15341.07686	0.228046583	2.458018337
	2192924	73913.87971	19164.59686	0.259282789	5.685874503
	2060334	71535.37894	16906.38145	0.236335946	4.869309847
	1907358	69204.05371	16832.81437	0.243234514	4.63935296
	1335992	72694.72319	13565.16597	0.186604548	2.493021833
	1432043	43988.10855	10256.65939	0.233168912	3.339079077
	1096877	16993.44689	6952.35942	0.409120025	4.487543459
	1554774	62708.01486	13490.86238	0.215137768	3.344906087
	1412390	47466.68469	12797.74421	0.269615295	3.808019469
	1075981	13288.55623	7435.723954	0.559558452	6.020742629
	1941036	27040.68678	12228.75402	0.452235334	8.778050642
	856242	13749.53927	6174.508531	0.449070213	3.845127776
	922620	34943.86148	8107.685958	0.232020321	2.140665886
	1312630	20102.00273	8178.909635	0.406870387	5.340702763
	984656	14314.30865	7535.985387	0.526465201	5.183871193
	1290425	36170.447	8562.284074	0.236720438	3.054699718
…opathy patients	754608	15902.34376	6730.635876	0.423248043	3.193863593
	1240793	24214.4002	8636.919719	0.356685264	4.425725783
	1833039	51966.79377	16667.10576	0.320726074	5.879034025
	1842074	21789.28702	6387.661807	0.293156073	5.400151792
	946799	24859.11481	8609.580051	0.346334941	3.279095754

Pooled primary age-related Ta	1165717	36127.08512	11242.54535	0.311194366	3.627645629
	820524	22555.34557	7412.535208	0.328637625	2.69655059
	542313	32260.6739	6001.356806	0.186027013	1.008848676
	1321839	38915.00473	9644.735019	0.247841034	3.276059448
	1476311	47958.63041	10871.13681	0.226677383	3.346463132
	1323996	36091.48315	10138.37829	0.280907777	3.719207727
	1303592	51857.74001	17602.27377	0.339433878	4.424832873
	1748854	36369.7538	14236.18333	0.391429192	6.845525076
	1611519	51199.79573	16553.08905	0.32330381	5.210102331

Table S14. Post-mortem human brain demographics, related to Figure 6J.

**Age (Years)**	**Gender**	**Neuropathological diagnosis**
89+	Woman	Control
84	Woman	Alzheimer disease (AD) neuropathologic changes
78	Man	Alzheimer disease (AD) neuropathologic changes
50	Woman	Alzheimer disease (AD) neuropathologic changes
85	Woman	Alzheimer disease (AD) neuropathologic changes
73	Man	Alzheimer disease (AD) neuropathologic changes
89+	Man	Alzheimer disease (AD) neuropathologic changes
89+	Woman	Primary age-related tauopathy
73	Woman	Primary age-related tauopathy

Table S15. Reagents

Materials				
**Peptide**				
**Synthetic peptide**	**Peptide sequence**			
TR-Aβ42 (From Bhattarai et al., 2016)	GWTLNSAGYLLGKINLKALAALAKKIL - DAEFRHDSGYEVHHQKLVFFAEDVGSNKGAIIGLMVGGVVIA	Coupled with Trans-Cell Penetrating peptide		
**Antibodies**				
**Primary Antibodies**	**Company**	**Catalog Number**	**Dilution**	**Species**
anti-S100	Dako	Z0311	1:500	rabbit IgG
anti-GFP	Abcam	ab13970	1:2000	chicken IgY
anti-ß-amyloid (4G8)	Biolegend (previously Covance)	SIG-39220	1:2000	mouse mAb IgG2b
anti-FMNL2	Invitrogen	PA5-52148	1:250	rabbit IgG
anti-FMNL2	Abnova	H00114793-M01	1:250	mouse IgG1
anti-ZO-1 (ZO1-1A12)	Invitrogen	33-9100	1:500	mouse mAb IgG1
anti-ß-amyloid (D54D2))	Cell Signaling	8243	1:500	rabbit mAb IgG
anti-GFAP	ThermoFisher / Invitrogen	OPA1-06100	1:250	rabbit IgG
anti-CD31	BD Pharma	555444	1:100	Mouse IgG1
anti-GFAP	Thermo Scientific	PA5-18598	1:500	Goat IgG
anti-4G8 (anti-β-amyloid)	Biolegend	800701	1:2000	Mouse IgG2b
**Secondary Antibodies**	**Company**		**Dilution**	
Goat anti-mouse IgG1 Alexa 488, 555	Molecular Probes, Thermo Fischer Scientific		1:500	
Goat anti-mouse IgG2b Alexa 555	Molecular Probes, Thermo Fischer Scientific		1:500	
Goat anti-rabbit IgG Alexa 555, 647	Molecular Probes, Thermo Fischer Scientific		1:500	
Goat anti-chicken IgY Alexa 488	Molecular Probes, Thermo Fischer Scientific		1:500	
**Other**				
**Counter Staining**	**Company**	**Catalog Number**	**Concentration**	
DAPI	Invitrogen	D21490	0.1mg/ml	
Alexa Fluor™ 488 Phalloidin	Invitrogen	A12379	1 unit	
**Morpholino for fmnl2**				
fmnl2a morpholino Oligo Sequence	5’-GGTTTTGTGATTTACCTGGTAGTTC-3’	from Wakayama et al. 2015, Dev Cell		
fmnl2b morpholino Oligo Sequence	5’-GAAGGGAAAGTAGACCCACCATGAA-3	from Wakayama et al. 2015, Dev Cell		
control morpholino Oligo Sequence	5'-CCTCTTACCTCAGTTACAATTTATA-3'	vivo standard control, from Gene Tools		
